# Cross-Feedings, Competition, and Positive and Negative Synergies in a Four-Species Synthetic Community for Anaerobic Degradation of Cellulose to Methane

**DOI:** 10.1128/mbio.03189-22

**Published:** 2023-02-27

**Authors:** Dongyu Wang, Kristopher A. Hunt, Pieter Candry, Xuanyu Tao, Neil Q. Wofford, Jizhong Zhou, Michael J. McInerney, David A. Stahl, Ralph S. Tanner, Aifen Zhou, Mari Winkler, Chongle Pan

**Affiliations:** a Department of Microbiology and Plant Biology, University of Oklahoma, Norman, Oklahoma, USA; b Department of Civil and Environmental Engineering, University of Washington, Seattle, Washington, USA; c Institute for Environmental Genomics, University of Oklahoma, Norman, Oklahoma, USA; d School of Civil Engineering and Environmental Sciences, University of Oklahoma, Norman, Oklahoma, USA; e Earth and Environmental Sciences, Lawrence Berkeley National Laboratory, Berkeley, California, USA; f School of Computer Science, University of Oklahoma, Norman, Oklahoma, USA; Shanghai Jiao Tong University; Rutgers, The State University of New Jersey

**Keywords:** synthetic community, anaerobic metabolism, microbial interactions, sulfate perturbation

## Abstract

Complex interactions exist among microorganisms in a community to carry out ecological processes and adapt to changing environments. Here, we constructed a quad-culture consisting of a cellulolytic bacterium (Ruminiclostridium cellulolyticum), a hydrogenotrophic methanogen (Methanospirillum hungatei), an acetoclastic methanogen (Methanosaeta concilii), and a sulfate-reducing bacterium (Desulfovibrio vulgaris). The four microorganisms in the quad-culture cooperated via cross-feeding to produce methane using cellulose as the only carbon source and electron donor. The community metabolism of the quad-culture was compared with those of the R. cellulolyticum-containing tri-cultures, bi-cultures, and mono-culture. Methane production was higher in the quad-culture than the sum of the increases in the tri-cultures, which was attributed to a positive synergy of four species. In contrast, cellulose degradation by the quad-culture was lower than the additive effects of the tri-cultures which represented a negative synergy. The community metabolism of the quad-culture was compared between a control condition and a treatment condition with sulfate addition using metaproteomics and metabolic profiling. Sulfate addition enhanced sulfate reduction and decreased methane and CO_2_ productions. The cross-feeding fluxes in the quad-culture in the two conditions were modeled using a community stoichiometric model. Sulfate addition strengthened metabolic handoffs from R. cellulolyticum to M. concilii and D. vulgaris and intensified substrate competition between M. hungatei and D. vulgaris. Overall, this study uncovered emergent properties of higher-order microbial interactions using a four-species synthetic community.

## INTRODUCTION

Complex anaerobic microbial communities can degrade cellulose to methane, CO_2_, and other products in many anoxic ecosystems, including wetlands (1), rumen gut (2), waste treatment systems (3), and biogas digesters (4). In these communities, cellulolytic bacteria metabolize cellulosic materials primarily to CO_2_, hydrogen (H_2_), and a variety of organic acids such as acetate, lactate, formate, and succinate (5). Then, H_2_ and CO_2_ are converted to CH_4_ by hydrogenotrophic methanogens, and acetate is metabolized to CH_4_ by acetoclastic methanogens. When sulfate is available, sulfate-reducing bacteria (SRB) may oxidize acetate and lactate to CO_2_ and reduce sulfate to H_2_S. When sulfate is limited, some SRB grow as syntrophic oxidizers, fermenting lactate to acetate, H_2_, and CO_2_ (6). The overall methane and CO_2_ emissions are determined by the nutrient and redox potential of their local environments that eventually drive the interactions among these functional guilds (7–9). Increased sulfate availability caused by, for example, intrusion of sulfate-laden seawater to estuarine wetlands (10), will likely enable SRB to produce more H_2_S, utilizing H_2_ and/or organic acids such as acetate and lactate as electron donors. The competition from SRB for H_2_ and acetate may reduce methane production by hydrogenotrophic and acetoclastic methanogens (7, 11). Characterization of these microbial interactions will provide insights into community metabolism under changing environmental conditions and allow modeling of metabolic flux rates among community members.

It is a challenge to mechanistically study complex microbial interactions in natural communities. Synthetic communities provide a complementary reductionist system to characterizing microbial interactions from microbiological, ecological, and biochemical perspectives. Synthetic communities can be directly manipulated, and the response of individual community members can be identified (12, 13). Previous synthetic community studies have investigated mutualistic interactions between Methanococcus maripaludis and Desulfovibrio vulgaris in bi-cultures (9), cooperative interactions between Geobacter metallireducens and Geobacter sulfurreducens (14), cross-feeding between Escherichia coli and Rhodopseudomonas palustris (15), and competitive interaction in a synthetic community of Pseudomonas aeruginosa and Agrobacterium tumefaciens (16). Rationally designed synthetic communities are experimentally more tractable and enable identifying interactions between key functional guilds that are challenging to assess in environmental communities.

In this study, we developed a simplified, yet functionally complete, synthetic community to study the microbial interactions and carbon exchanges during anaerobic carbon degradation. Our synthetic microbial community was composed of the major functional guilds (cellulolytic fermenter, sulfate reducer, hydrogenotrophic methanogen, and acetoclastic methanogen) that mediate the anaerobic conversion of cellulosic biomass to CH_4_ and CO_2_. The choice of a sulfate-reducing bacterium (Desulfovibrio vulgaris Hildenborough) introduced metabolic versatility and enabled investigations into the community response to sulfate intrusion. We characterized the biochemical and physiological response of each microorganism in the synthetic community, investigated the ecological and metabolic functions, and modeled the interspecies interactions governing carbon exchange among these four organisms. This study provides a foundation for the development of mechanistic metabolic models of anaerobic degradation of cellulose to methane, CO_2_, and H_2_S.

## RESULTS

### Overview of the experimental design.

**FIG 1:**
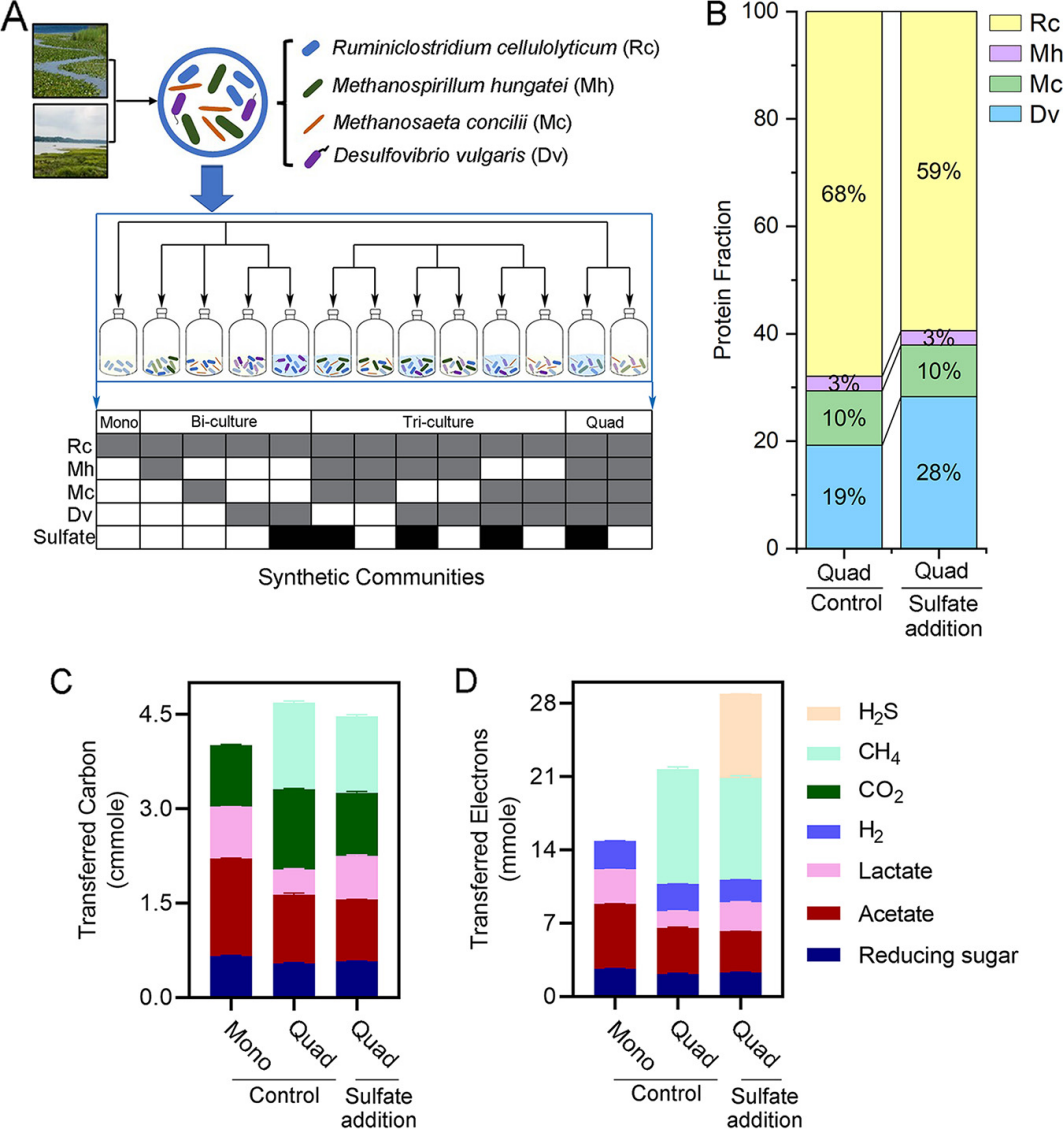
Perturbation of the synthetic communities by sulfate addition. (A) Schematics of the synthetic community assembly. (B) Proteome composition of the quad-cultures in the control and sulfate addition conditions. (C and D) Distribution of the fermentation products in R. cellulolyticum mono-culture and the quad-cultures by carbon molarity (C) and electron molarity (D). Rc, Ruminiclostridium cellulolyticum; Mh, Methanospirillum hungatei; Mc, Methanosaeta concilii; Dv, Desulfovibrio vulgaris.

A four-species synthetic community consisting of a cellulolytic bacterium (Ruminiclostridium cellulolyticum), a hydrogenotrophic methanogen (Methanospirillum hungatei), an acetoclastic methanogen (Methanosaeta concilii), and a sulfate-reducing bacterium (Desulfovibrio vulgaris) was constructed. To unravel how each species and their interactions contribute to the community metabolism, the growth of this quad-culture was compared with those of a mono-culture, three bi-cultures, and three tri-cultures (Fig. 1A; Table 1). All cultures were grown anaerobically in triplicate using the same medium with cellulose as the sole carbon source. To simulate the increased sulfate availability from seawater intrusion into a wetland soil community, the D. vulgaris-containing cultures received a daily sulfate addition after the first day of cultivation. The growth status of these multispecies cultures was measured over a week by daily analysis of substrate consumption and product accumulation. The quad-cultures were analyzed with metaproteomics at the end of experiment to characterize the community structure and metabolic activities. A stoichiometric model was built to analyze the cross-feeding fluxes through the four species (Fig. 1A; see Table S1 and Table S2 in the supplemental material).

**TABLE 1:** Metabolites of the synthetic communities

Species and metabolite	Mono-culture	Bi-cultures				Tri-cultures						Quad-cultures	
Community assembly													
R. cellulolyticum	Yes	Yes	Yes	Yes	Yes	Yes	Yes	Yes	Yes	Yes	Yes	Yes	Yes
M. hungatei	No	No	Yes	No	No	Yes	No	Yes	Yes	No	Yes	Yes	Yes
M. concilii	No	Yes	No	No	No	Yes	Yes	No	Yes	Yes	No	Yes	Yes
D. vulgaris	No	No	No	Yes	Yes	No	Yes	Yes	No	Yes	Yes	Yes	Yes
Growth condition													
Sulfate addition	No	No	No	No	Yes	No	No	No	Yes	Yes	Yes	No	Yes
Substrate													
Cellulose (mg)	184.39 ± 0.54	197.77 ± 2.23	198.17 ± 2.60	203.73 ± 0.77	205.43 ± 0.31	207.11 ± 1.00	200.98 ± 1.27	200.67 ± 4.09	201.46 ± 0.97	200.49 ± 1.26	197.87 ± 0.47	210.02 ± 2.10	205.44 ± 1.63
Products in gas phase													
CH_4_ (mmol)	0	0.18 ± 0.003	0.42 ± 0.007	0	0	0.62 ± 0.02	0.16 ± 0.01	0.89 ± 0.02	0.53 ± 0.003	0.14 ± 0.001	0.74 ± 0.007	1.37 ± 0.03	1.21 ± 0.03
CO_2_ (mmol)	0.98 ± 0.01	1.08 ± 0.03	0.99 ± 0.03	1.04 ± 0.03	0.92 ± 0.003	1.14 ± 0.01	1.11 ± 0.02	1.22 ± 0.03	1.15 ± 0.005	0.99 ± 0.03	0.97 ± 0.005	1.28 ± 0.007	1.00 ± 0.02
H_2_ (mmol)	1.36 ± 0.02	1.47 ± 0.03	1.23 ± 0.01	1.52 ± 0.003	1.21 ± 0.002	1.42 ± 0.03	1.30 ± 0.01	1.32 ± 0.03	1.27 ± 0.01	1.09 ± 0.01	1.04 ± 0.01	1 0.30 ± 0.02	1.07 ± 0.01
H_2_S (mmol)^*a*^	0	0	0	0	1.17 ± 0.01	0	0	0	0	1.17 ± 0.01	1.17 ± 0.01	0	1.17 ± 0.01
Products in liquid phase													
Acetate (mmol)	0.78 ± 0.002	0.48 ± 0.01	0.78 ± 0.02	0.65 ± 0.006	0.59 ± 0.03	0.60 ± 0.02	0.52 ± 0.01	0.71 ± 0.02	0.59 ± 0.01	0.44 ± 0.002	0 0.62 ± 0.005	0.55 ± 0.01	0.49 ± 0.001
Lactate (mmol)	0.27 ± 0.001	0.29 ± 0.005	0.29 ± 0.005	0.13 ± 0.005	0.26 ± 0.006	0.29 ± 0.02	0.15 ± 0.009	0.14 ± 0.001	0.30 ± 0.004	0.27 ± 0.006	0.28 ± 0.00 5	0.13 ± 0.004	0.23 ± 0.005
Glucose (μmol)	0.35 ± 0.002	0.19 ± 0.02	0.23 ± 0.01	1.51 ± 0.12	1.25 ± 0.03	0.64 ± 0.004	4.51 ± 0.08	2.89 ± 0.13	0.21 ± 0.01	4.40 ± 0.12	2.13 ± 0.09	1.6 6 ± 0.11	1.15 ± 0.03
Cellobiose (μmol)	2.34 ± 0.02	7.30 ± 1.75	3.71 ± 0.16	9.83 ± 0.24	6.14 ± 0.04	3.55 ± 0.47	15.24 ± 0.01	6.45 ± 0.06	4.08 ± 0.09	13.93 ± 0.06	3.10 ± 0.02	1.70 ± 0.20	2.33 ± 0.11

a H_2_S accumulation inferred from the sulfate consumption. Zero indicates negligible sulfate consumption.

10.1128/mbio.03189-22.3 TABLE S1 General concept of the stoichiometric model under control condition. Download Table S1, PDF file, 0.2 MB. Copyright © 2023 Wang et al. 2023 Wang et al. https://creativecommons.org/licenses/by/4.0/ This content is distributed under the terms of the Creative Commons Attribution 4.0 International license.

10.1128/mbio.03189-22.4 TABLE S2 General concept of the stochiometric model under sulfate addition condition. Download Table S2, PDF file, 0.3 MB. Copyright © 2023 Wang et al. 2023 Wang et al. https://creativecommons.org/licenses/by/4.0/ This content is distributed under the terms of the Creative Commons Attribution 4.0 International license.

Metaproteomics analyses measured the relative proteome abundances of the organisms in the quad-cultures (Fig. 1B) (17, 18). A total of 3,736 proteins were identified from the four microbial species (Table S4). R. cellulolyticum was dominant in the quad-cultures as the producer of the growth substrates for the other three microorganisms. Sulfate addition reduced the relative proteome abundance of R. cellulolyticum, on average, from 68% to 59% (*P* = 0.047), while it increased that of D. vulgaris, on average, from 19% to 28% (*P* = 0.0004). The relative proteome abundances of M. concilii and M. hungatei remained at 10% and 3%, respectively, in both the control condition and the sulfate addition condition. The fermentation products of the synthetic communities were tracked by carbon molarity (Fig. 1C) and electron molarity (Fig. 1D). The mono-culture of R. cellulolyticum hydrolyzed cellulose to reducing sugars and fermented these sugars to acetate, lactate, ethanol, H_2_, and CO_2_. The quad-cultures transferred 1.37 ± 0.03 (29%) mmol of carbon (Fig. 1C) and 10.99 ± 0.21 (51%) mmol of electrons (Fig. 1D) to methane in the control condition. The quad-cultures transferred 9.36 ± 0.04 (43%) mmol of electrons (Fig. 1C) to H_2_S in the sulfate addition condition. Sulfate addition reduced the electrons and carbon transferred to methane by 1.28 ± 0.02 mmol and 0.16 ± 0.02 mmol, respectively, compared to the control condition.

10.1128/mbio.03189-22.6 TABLE S4 Metaproteomics results. Download Table S4, PDF file, 0.8 MB. Copyright © 2023 Wang et al. 2023 Wang et al. https://creativecommons.org/licenses/by/4.0/ This content is distributed under the terms of the Creative Commons Attribution 4.0 International license.

### Metabolism of cellulolytic bacterium Ruminiclostridium cellulolyticum.

**FIG 2:**
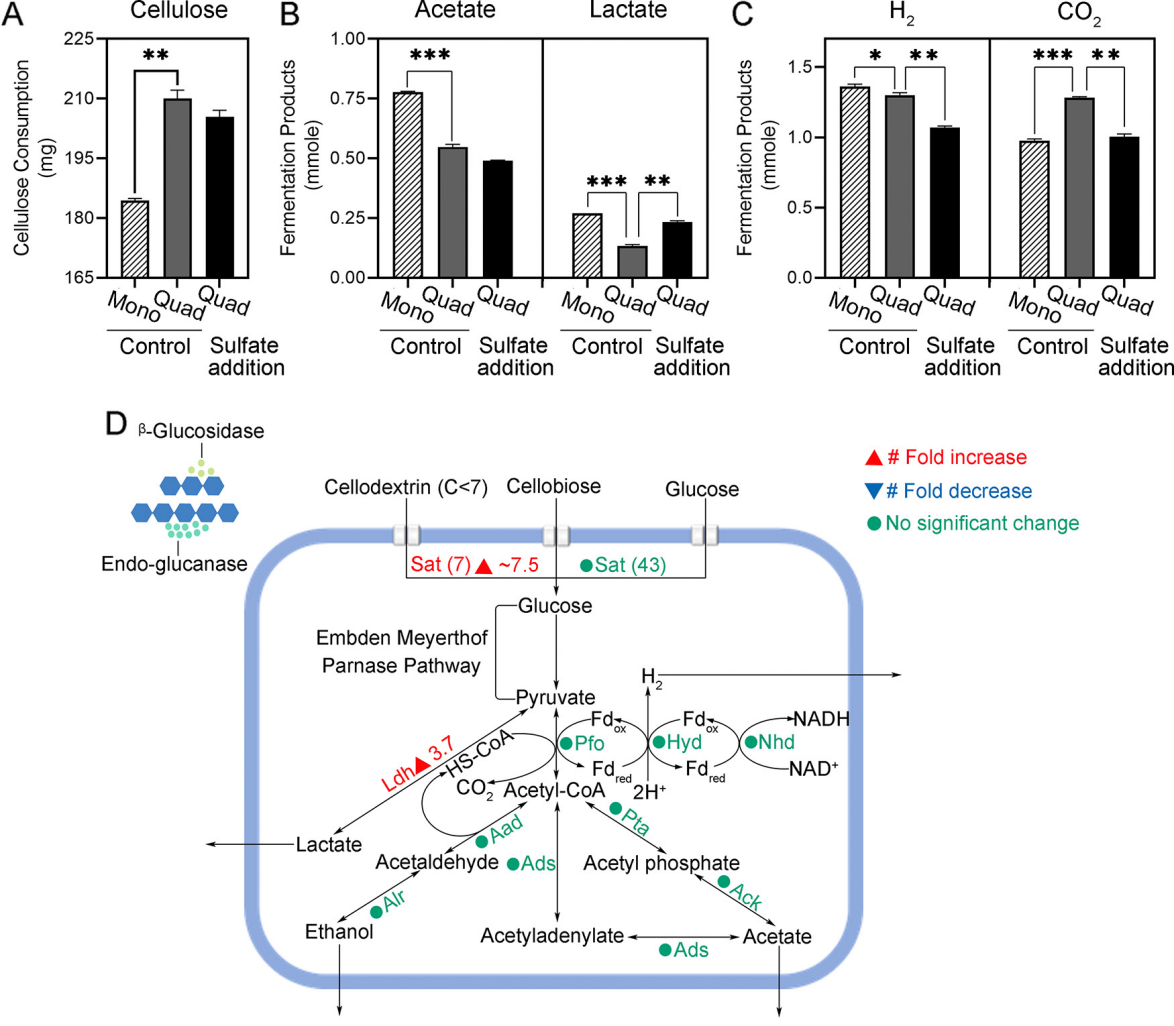
Metabolism of R. cellulolyticum. (A) Cellulose consumption. (B) Accumulation of acetate and lactate in the liquid phase. (C) Accumulation of H_2_ and CO_2_ in the gas phase. Significant differences (*, *P* < 0.05; **, *P* < 0.01; ***, *P* < 0.001) indicate targeted biological hypotheses. (D) Protein expression profile of the major carbon metabolism pathways in R. cellulolyticum. Enzymes are color-coded based on their protein abundance changes induced by sulfate addition. Unidentified enzymes are not labeled. Sat, sugar-related ABC transporter; Pfo, pyruvate flavodoxin/ferredoxin oxidoreductase; Hyd, hydrogenase, Fe only; Nhd, flavin oxidoreductase/NADH oxidase; Ldh, l-lactate dehydrogenase; Aad, acetaldehyde dehydrogenase; Alr, alcohol dehydrogenase; Ads, AMP-dependent synthetase; Ack, acetate kinase; Pta, phosphate acetyltransferase.

Cellulose was the only carbon source provided to all the synthetic communities. After 2 days of incubation, all cultures began to rapidly consume cellulose (Fig. S1). Over 7 days of incubation, the R. cellulolyticum mono-culture degraded 184.39 ± 0.54 mg of cellulose. Cellulose degradation increased significantly when R. cellulolyticum was cultivated with the other three species (*P* = 0.0028) (Table 1). The highest cellulose degradation was achieved by the quad-culture (210.02 ± 2.10 mg) (Fig. 2A).

10.1128/mbio.03189-22.1 FIG S1 Cellulose consumption by the synthetic communities in the control condition. Rc, Ruminiclostridium cellulolyticum; Mc, Methanosaeta concilii; Mh, Methanospirillum hungatei; Dv, Desulfovibrio vulgaris. Gray filling indicates the microorganisms contained in the corresponding cultures. Download FIG S1, TIF file, 0.2 MB. Copyright © 2023 Wang et al. 2023 Wang et al. https://creativecommons.org/licenses/by/4.0/ This content is distributed under the terms of the Creative Commons Attribution 4.0 International license.

Metaproteomic analysis of the quad-culture identified 64 R. cellulolyticum cellulase and 50 R. cellulolyticum ABC transporters related to uptake of monosaccharides, disaccharides, and oligosaccharides. Glucose and cellobiose produced by cellulose hydrolysis were detected at micromolar concentrations in the medium (Table 1). In the R. cellulolyticum mono-culture acetate and lactate accumulated in the medium (Fig. 2B), and H_2_ and CO_2_ accumulated in the headspace (Fig. 2C). These fermentation products from R. cellulolyticum can be used by other microorganisms as carbon and/or energy sources in multispecies cultures. Compared with the R. cellulolyticum mono-culture 29.5% less acetate (*P* = 0.0006), 51.9% less lactate (*P* = 0.0002), 4.4% less H_2_ (*P* = 0.027), and 30.6% more CO_2_ (*P* < 0.0001) accumulated in the quad-culture control condition without sulfate addition (Fig. 2C).

Metaproteomics identified key enzymes in the fermentative pathways in R. cellulolyticum, including hydrogenase (Hyd) for H_2_ generation, pyruvate flavodoxin/ferredoxin oxidoreductase (Pfo) for CO_2_ production, L-lactate dehydrogenase (Ldh) for lactate generation, and phosphotransacetylase (Pta), AMP-forming acetyl-CoA synthetase (Ads), and acetate kinase (Ack) for acetate generation (Fig. 2D). The protein abundance of L-lactate dehydrogenase from R. cellulolyticum in the quad-culture increased by 3.7-fold in the sulfate addition condition compared to the control condition (*q* = 0.04). This may have contributed to the 1.8-fold higher lactate accumulation in the quad-cultures in the sulfate addition condition than the control condition (*P* = 0.003) (Fig. 2B).

### Metabolism of hydrogenotrophic methanogen Methanospirillum hungatei and acetoclastic methanogen Methanosaeta concilii.

**FIG 3:**
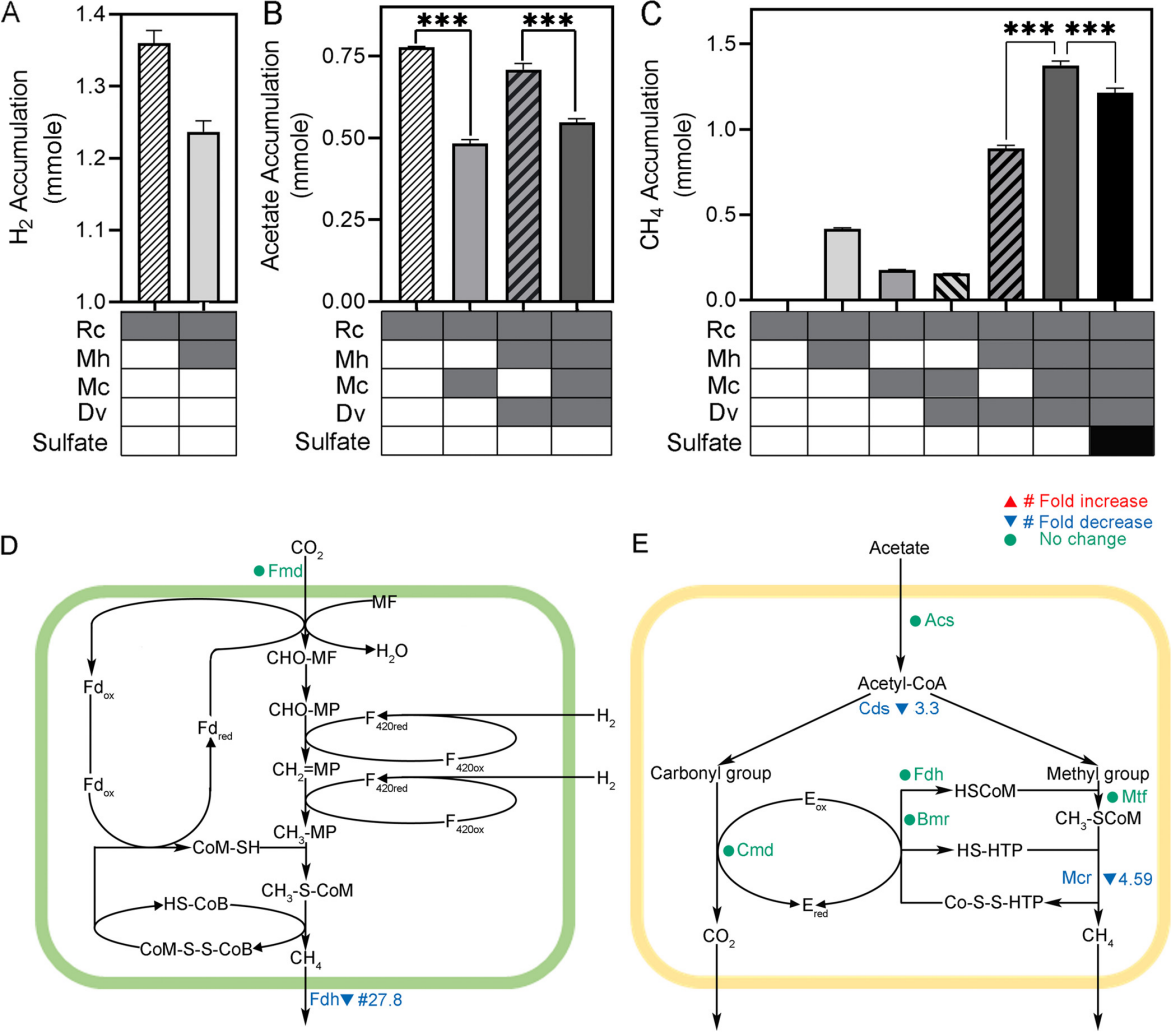
Metabolism of Methanospirillum hungatei and Methanosaeta concilii. (A) H_2_ accumulation. (B) Acetate accumulation. (C) CH_4_ accumulation. The table on the *x* axis marks the species included in each culture and the presence or absence of sulfate addition. Significant differences (***, *P* < 0.001) indicate targeted biological hypotheses. (D) Hydrogenotrophic methanogenesis in M. hungatei. (E) Acetoclastic methanogenesis in M. concilii. Enzymes are color-coded based on their protein abundance changes induced by sulfate addition. Unidentified enzymes are not labeled. Fmd, formylmethanofuran dehydrogenase; Fdh, formate dehydrogenase; Acs, acetyl-coenzyme A synthetase; Cds, CO dehydrogenase/acetyl-CoA synthase; Cmd, carbon monoxide dehydrogenase CoM; Mtf, tetrahydromethanopterin *S*-methyltransferase; Mcr, methyl-coenzyme M reductase; Bmr, CoB-CoM reductase.

M. hungatei was selected as a model hydrogenotrophic methanogen. The accumulation of H_2_ in the bi-culture of R. cellulolyticum and M. hungatei over 7 days (1.23 ± 0.01 mmol) was approximately 0.13 mmol lower than in the R. cellulolyticum mono-culture (1.36 ± 0.02 mmol) (Fig. 3A). Methane (0.42 ± 0.007 mmol) accumulated in the bi-culture of R. cellulolyticum and M. hungatei. Since M. hungatei needs to consume 4 H_2_ per CH_4_ generated, approximately 2.91 mmol of H_2_ [(0.42 mmole CH4 × 4) + 1.23 mmole H_2_] should be produced by R. cellulolyticum in the bi-culture, which was more than twice that in the R. cellulolyticum mono-culture (1.36 ± 0.02 mmol). This indicates that M. hungatei promoted H_2_ production by R. cellulolyticum through the alleviation of product inhibition. The methane production increased from 0.16 ± 0.01 mmol in the tri-culture without M. hungatei to 1.37 ± 0.03 mmol in the quad-culture with M. hungatei (Fig. 3C). Two key enzymes in hydrogenotrophic methanogenesis, formylmethanofuran dehydrogenase (Fmd) and formate dehydrogenase (Fdh), were identified in the quad-cultures by metaproteomics (Fig. 3D). This indicated active hydrogenotrophic methanogenesis by M. hungatei in the multispecies cultures.

M. concilii was added as a model acetoclastic methanogen. The net accumulation of acetate in the multispecies cultures containing M. concilii increased in the first 5 or 6 days and then decreased or leveled off in the last 1 or 2 days, which suggested a lag in acetate consumption (Fig. S2). In the cultures lacking M. concilii, the amount of acetate kept increasing during the 7-day growth. The accumulative amount of acetate in the bi-culture of M. concilii and R. cellulolyticum (0.48 ± 0.01 mmol) was significantly lower than in the mono-culture of R. cellulolyticum alone (0.78 ± 0.002 mmol) (*P* = 0.0006). The accumulation of acetate in the quad-culture (0.55 ± 0.01 mmol) was also significantly lower than that in the tri-culture that lacked M. concilii (0.71 ± 0.02 mmol) (*P* = 0.0005) (Fig. 3B). Approximately 0.48 mmol more methane accumulated in the quad-culture (1.37 ± 0.03 mmol) than in the tri-culture (0.89 ± 0.02 mmol) without M. concilii (Fig. 3C) (*P* < 0.0001). In the M. concilii acetoclastic methanogenesis pathway, acetyl-coenzyme A synthetase (Acs), CO dehydrogenase/acetyl-CoA synthase (Cds), tetrahydromethanopterin *S*-methyltransferase, methyl-coenzyme A reductase (Mtf), and CoB-CoM reductase (Mcr) were identified by metaproteomics (Fig. 3E). This indicated active acetoclastic methanogenesis by M. concilii in the multispecies cultures.

10.1128/mbio.03189-22.2 FIG S2 Acetate accumulation over 7 days of cultivation in the control condition. Rc, Ruminiclostridium cellulolyticum; Mc, Methanosaeta concilii; Mh, Methanospirillum hungatei; Dv, Desulfovibrio vulgaris. Gray filling indicates the microorganisms contained in the corresponding cultures. Download FIG S2, TIF file, 0.7 MB. Copyright © 2023 Wang et al. 2023 Wang et al. https://creativecommons.org/licenses/by/4.0/ This content is distributed under the terms of the Creative Commons Attribution 4.0 International license.

Sulfate addition reduced quad-culture methane production by 11.7% (*P* < 0.0001) (Fig. 3C). The protein abundance of formate dehydrogenase, involved in the hydrogenotrophic methanogenesis in M. hungatei, significantly decreased by 27.8-fold (*q* < 0.0001) (Fig. 3D). In addition, sulfate addition significantly decreased the protein abundances of enzymes involved in acetoclastic methanogenesis. Specifically, the protein abundances of CO dehydrogenase/acetyl-CoA synthase (*q* = 0.03) and methyl-coenzyme M reductase (*q* = 0.001) significantly decreased by at least 4-fold (Fig. 3E). These decreases indicated that the sulfate addition suppressed both hydrogenotrophic methanogenesis and acetoclastic methanogenesis in the quad-culture.

### Metabolism of the sulfate-reducing bacterium Desulfovibrio vulgaris.

**FIG 4:**
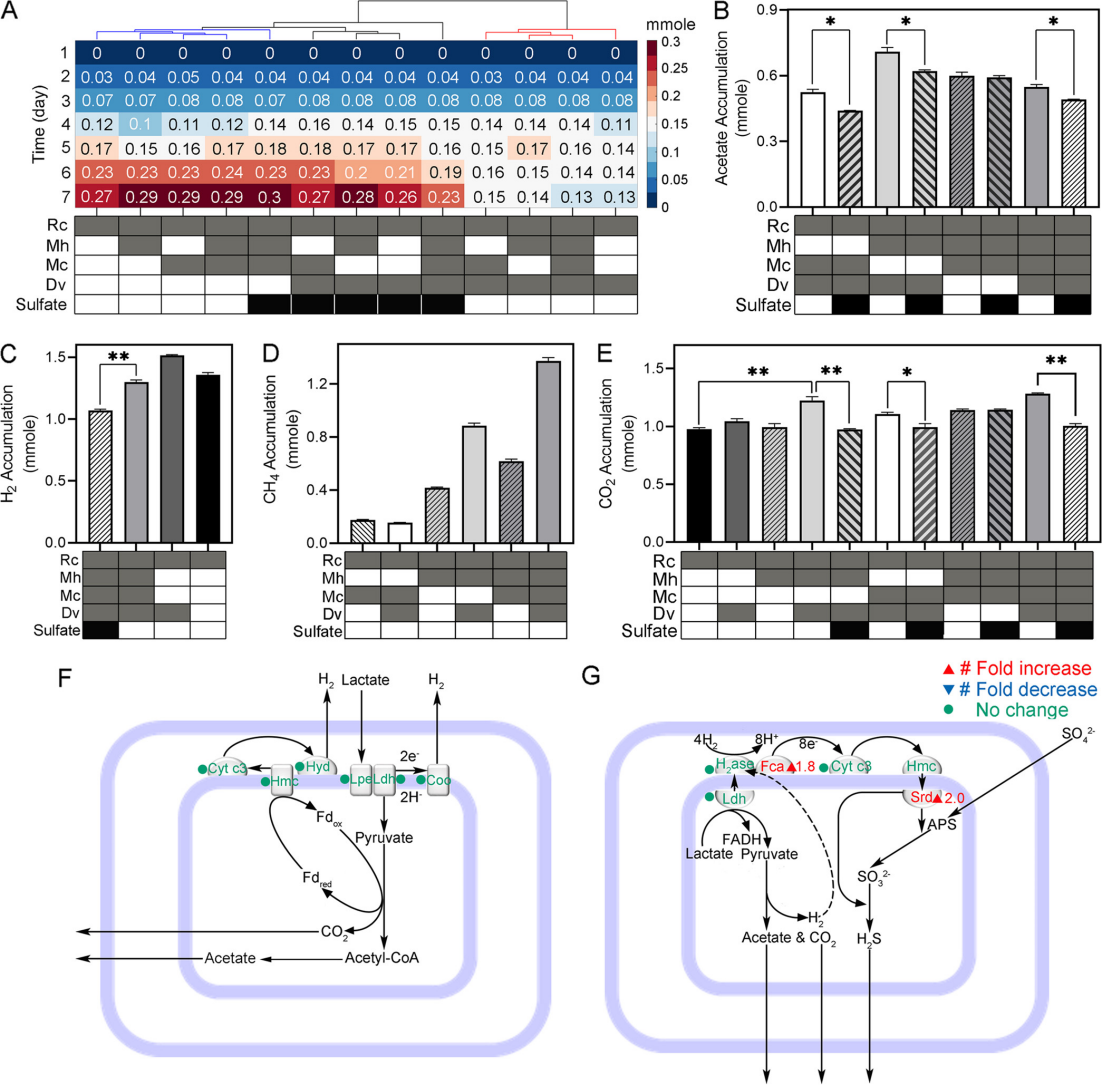
Metabolism of D. vulgaris. (A) Lactate accumulation. A column represents daily lactate accumulation in a culture over 7 days of cultivation. The cultures with sulfate additions were clustered together. (B) Acetate accumulation. (C) H_2_ accumulation. (D) CH_4_ accumulation. (E) CO_2_ accumulation. Significant differences (*, *P* < 0.05; **, *P* < 0.01) indicate targeted biological hypotheses. (F) Hydrogenic lactate fermentation in D. vulgaris. (G) Sulfidogenic lactate oxidation and sulfidogenic hydrogenic oxidation in D. vulgaris. Cyt c_3_, cytochrome *c*_3_; Hmc, high-molecular cytochrome; Hdy, hydrogenase; Lpe, l-lactate permease; Coo, membrane-bound Coo hydrogenase; H_2_ase, hydrogenase; Fca, FeS cluster assembly ATPase; Ldh, lactate dehydrogenase; Srd, sulfite reductase. The table on the *x* axis marks the species included in each culture and the presence or absence of sulfate addition.

D. vulgaris can ferment lactate to acetate, H_2_, and CO_2_ when sulfate is limited (Fig. 4F) or reduce sulfate with lactate or H_2_ as electron donors (Fig. 4G) (19). In the control condition, while lactate in the cultures without D. vulgaris accumulated continuously over 7 days, lactate in the cultures with D. vulgaris increased in the first 3 days and then decreased slightly from day 4 onward. Over 7 days, 0.13 ± 0.005 mmol lactate accumulated in the bi-culture of R. cellulolyticum and D. vulgaris, which was 51.9% lower than that in the mono-culture (0.27 ± 0.001 mmol) (*P* = 0.0005). The lactate accumulation by the tri-culture that lacked D. vulgaris (0.29 ± 0.02 mmol) was also higher than that in the quad-culture that included D. vulgaris (0.13 ± 0.004 mmol) (*P* = 0.002) (Fig. 4A). Metaproteomics identified cytochrome *c*_3_ (*c*_3_), high-molecular-weight cytochrome (Hmc), periplasmic [NiFe] hydrogenase (Hyd), membrane-bound Coo hydrogenase (Coo), lactate dehydrogenase (Ldh) and L-lactate permease (Lpe) in the hydrogenic lactate oxidation pathway of D. vulgaris (Fig. 4F). In the mono-culture of R. cellulolyticum, 1.36 ± 0.02 mmol H_2_ accumulated at the end of the cultivation, while 1.52 ± 0.003 mmol H_2_ accumulated in the bi-culture of R. cellulolyticum and D. vulgaris. These results suggest that D. vulgaris fermented lactate produced by R. cellulolyticum to H_2_.

The addition of D. vulgaris to the bi-culture of R. cellulolyticum and M. concilii did not significantly increase the methane production. But the addition of D. vulgaris to the bi-culture of R. cellulolyticum and M. hungatei significantly increased the methane production by ~112% (from 0.42 ± 0.007 mmol to 0.89 ± 0.02 mmol) (Fig. 4D). D. vulgaris enhanced hydrogenotrophic methanogenesis, likely by producing additional hydrogen from lactate fermentation (9).

We found that the synergy between hydrogenotrophic and acetoclastic methanogenesis depended on the presence of D. vulgaris. Methane produced by the tri-culture of R. cellulolyticum, M. concilii, and M. hungatei (0.62 ± 0.02 mmol) was close to the sum (0.60 ± 0.01 mmol) of the methane produced by the bi-culture of R. cellulolyticum and M. concilii (0.18 ± 0.003 mmol) and the bi-culture of R. cellulolyticum and M. hungatei (0.42 ± 0.007 mmol). However, the methane produced by the quad-culture (1.37 ± 0.03 mmol) was ~30% higher than the sum (1.05 ± 0.02 mmol) of the methane produced by the tri-culture of R. cellulolyticum, D. vulgaris, and M. concilii (0.16 ± 0.001 mmol) and the tri-culture of R. cellulolyticum, D. vulgaris, and M. hungatei (0.89 ± 0.02 mmol) (Fig. 4D; Table 1). From another perspective to compare, the addition of D. vulgaris to the tri-culture of R. cellulolyticum, M. concilii, and M. hungatei increased total methane production by 0.75 mmol. This was 67% more than the additive increase of adding D. vulgaris to either a R. cellulolyticum-M. hungatei bi-culture (0.47 mmol increase) or to a R. cellulolyticum-M. concilii bi-culture (0.02 mmol decrease) (Fig. 4D). Both ways of comparison showed a positive synergistic relationship among methanogenesis and fermentation via D. vulgaris.

The mono-culture of R. cellulolyticum produced 0.98 ± 0.01 mmol of CO_2_. Coculturing R. cellulolyticum with D. vulgaris did not significantly increase their CO_2_ production (1.04 ± 0.03 mmol). Adding M. hungatei to R. cellulolyticum did not significantly change the CO_2_ production in their bi-culture (0.99 ± 0.03 mmol) either. However, combining both D. vulgaris and M. hungatei with R. cellulolyticum significantly increased CO_2_ production (1.22 ± 0.03 mmol). Further, adding M. concilii to this tri-culture did not result in a significant increase of CO_2_ production by the quad-culture (1.28 ± 0.007 mmol) (Fig. 4E). These results suggested a positive synergy between M. hungatei and D. vulgaris on CO_2_ production.

Sulfate addition enhanced sulfate reduction by D. vulgaris. At the end of cultivation, the quad-culture consumed 1.17 ± 0.01 mmol of sulfate in the sulfate addition condition (Table 1). Sulfate addition increased the relative abundance of sulfite reductase by 2.0-fold (*q* = 0.003) (Fig. 4G). H_2_ accumulation significantly decreased in the sulfate addition condition (1.07 ± 0.02 mmol) compared with the control condition (1.30 ± 0.02 mmol) (*P* = 0.003) (Fig. 4C). Sulfate reduction promoted sulfidogenic lactate oxidation as evidenced by a 1.8-fold increase of the relative abundance of FeS cluster assembly ATPase (Fca) (*q* = 0.009) in the sulfate addition condition (Fig. 4G). However, lactate accumulated in the first 3 days and then slightly decreased in the following 4 days in the control condition, but lactate continuously accumulated over the 7 days in the sulfate addition condition (Fig. 4A). The increased accumulation of lactate in the sulfate addition condition, despite the enhanced D. vulgaris sulfidogenic lactate oxidation, can be attributed to the increased R. cellulolyticum lactate fermentation stimulated by sulfate addition as described above (Fig. 2D).

Sulfate addition significantly decreased the CO_2_ accumulation in the two tri-cultures containing D. vulgaris and the quad-culture, but not in the tri-culture without D. vulgaris (Fig. 4E; Table 1). Sulfate addition also significantly reduced the acetate accumulation in the two tri-cultures containing D. vulgaris and the quad-culture, but not in the tri-culture without D. vulgaris (Fig. 4B; Table 1). These observations can be attributed to the suppression of the D. vulgaris fermentation of lactate to acetate and CO_2_ by the sulfate addition.

### Modeling of carbon and energy flow through the synthetic community.

**FIG 5:**
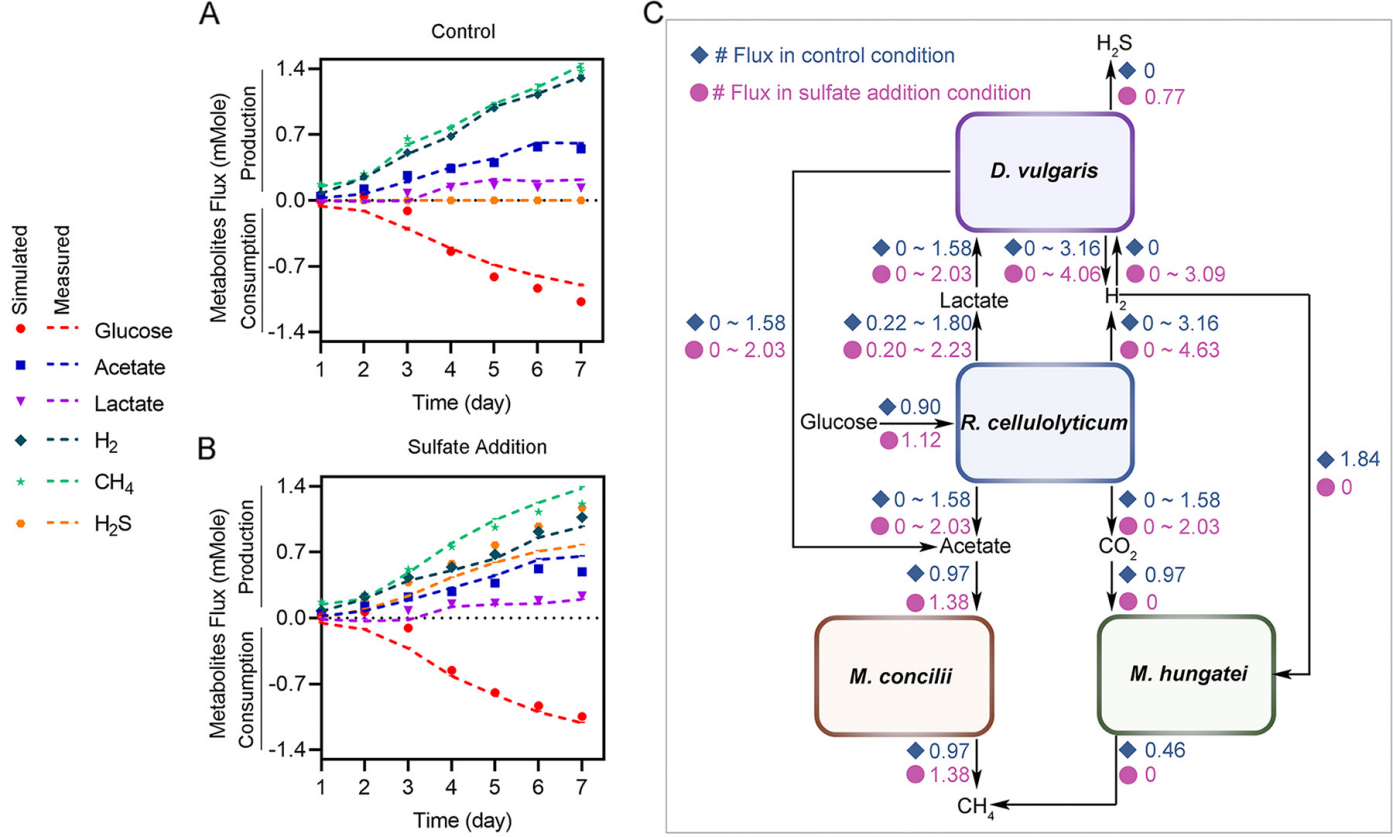
Community stoichiometric modeling. (A) Model fitting in the control condition. (B) Model fitting in sulfate addition condition. (C) Cross-feeding fluxes among the four species under the control condition and sulfate addition conditions.

A stoichiometric model was constructed to simulate fluxes through the primary energy production pathways in the four organisms (Tables S1 and S2). For simplicity, extracellular cellulose hydrolysis was omitted, and R. cellulolyticum was considered to ferment glucose. The overall model has seven reactions, including two for R. cellulolyticum, three for D. vulgaris, and one for each methanogen (Tables S1 and S2). Reactions that were not expected to be active were removed based on either genetic or nutrient availability in the system (i.e., sulfidogenic reactions were removed in the absence of sulfate, and acetoclastic methanogenesis was removed in the absence of M. concilii). The contributions of each reaction were fitted to the accumulative millimoles of glucose, acetate, lactate, H_2_, CH_4_, and H_2_S produced or consumed over the 7 days of incubation. To access redundancy of metabolic pathways in this stoichiometric model, each reaction pathway was sequentially excluded before fitting, and the impact on model fit was evaluated. These simulations identified two and three possible flux distributions through the quad-culture in the control and sulfate addition conditions (Tables S1 to Table S3) with equal goodness of fit to the experimental data. The correlation coefficients, *R*^2^, exceeded 0.9 in both conditions. The normalized root mean square errors (NRMSEs) were 0.11 ± 0.006 in the control condition and 0.25 ± 0.01 in the sulfate addition condition. The consistency between the experimental data and the modeling results indicated a reliable estimation of the carbon and energy flows (Fig. 5A and B).

R. cellulolyticum in the cultures was modeled by lactate fermentation and hydrogenic acetogenesis (Table S1). Sulfate addition stimulated the production of acetate and lactate in R. cellulolyticum. D. vulgaris was modeled to have three metabolic possibilities, lactate oxidation to acetate with proton reduction to hydrogen (hydrogenic lactate oxidation) (Fig. 4F), lactate oxidation to acetate with sulfate reduction to sulfide (sulfidogenic lactate oxidation) (Fig. 4G), and hydrogen oxidation to protons with sulfate reduction to sulfide (sulfidogenic hydrogen oxidation) (Fig. 4G). As expected, sulfate addition increased the estimated flux through sulfate reduction from 0 to 3.09 ± 0.03 mmol (*P* < 0.0001). The estimated flux of carbon and electrons through D. vulgaris was poorly constrained due to redundant pathways. However, the flux did increase within related possible flux distributions. For example, the maximum flux of hydrogen production by D. vulgaris increased from 3.16 ± 0.02 to 4.06 ± 0.02 mmol with the addition of sulfate, which agrees with the increased relative abundance of the D. vulgaris proteome in the community metaproteome (Fig. 1B). M. hungatei and M. concilii were represented by hydrogenotrophic methanogenesis and acetoclastic methanogenesis, respectively. Compared to the control condition, the addition of sulfate halted hydrogenotrophic methanogenesis in M. hungatei and increased the flux through acetoclastic methanogenesis in M. concilii by 1.42-fold (*P* < 0.0001).

The model estimated the cross-feeding fluxes from R. cellulolyticum to the other microorganisms (Fig. 5C). The fluxes of acetate and CO_2_ from R. cellulolyticum to M. concilii and M. hungatei were each 0.97 mmol in the control condition. In the sulfate addition condition, the flux of H_2_ consumption by M. hungatei fully stopped, suggesting that substrate competition for H_2_ by D. vulgaris was the main cause of the lower methane yields in the sulfate-amended quad-culture. Indeed, the H_2_ consumption flux of D. vulgaris increased from 0 in the control condition to 3.09 mmol under the sulfate addition condition. For comparison, sulfate addition increased H_2_ production by R. cellulolyticum from 3.16 to 4.63 mmol. Overall, this analysis demonstrates that sulfate addition deeply restructures carbon fluxes and metabolic hand-offs through the four-species synthetic community, ultimately affecting overall community metabolism.

## DISCUSSION

Synthetic communities have been shown to be a powerful approach to studying the interactions among major microbial guilds in the degradation of cellulosic biomass to methane and CO_2_. A bi-culture of D. vulgaris and Methanococcus maripaludis showed the requirement of hydrogen transfer for their syntrophic growth coupled with methane production (20). Cellulose degradation by Clostridium cellulovorans was enhanced by coculturing with Methanosarcina barkeri or Methanosarcina mazei (21). Coculturing R. cellulolyticum with D. vulgaris was also shown to accelerate cellulose degradation (22). However, these bi-culture can only reveal binary interactions between two organisms. By using a four-species synthetic community and comparing that to its constitutive parts, we investigated tertiary interactions (i.e., interactions of interactions) among four microorganisms involved in methanogenesis from cellulose. A tertiary interaction has positive synergy if the effect of combining three or more organisms on a phenotype of the community is greater than the addictive effects of combining two of these organisms separately. In contrast, a tertiary interaction has negative synergy when the effect of combining three or more organisms on a phenotype is less than the sum of the separate effects of combining the subsets. Here, we demonstrated that the bi-culture of R. cellulolyticum with any of the other 3 species increased cellulose degradation relative to the R. cellulolyticum mono-culture. This provides a baseline to determine the type of tertiary interactions. The quad-cultures further improved the cellulose degradation over any of the bi-cultures, but the increase of cellulose degradation by the quad-culture over the mono-culture (25.63 mg) was less than the sum of the increases by the 3 bi-cultures over the mono-culture (13.38 mg, 13.78 mg, and 19.34 mg). Such a negative synergy suggests that the putative beneficial effects of pH stabilization and cellulose colonization (21) provided by the methanogens and D. vulgaris may be antagonistic for cellulose degradation by R. cellulolyticum (23). The methane production by the tri-culture of R. cellulolyticum, M. concilii, and M. hungatei (0.62 ± 0.02 mmol) was approximately equal to the combined methane production by R. cellulolyticum-M. concilii bi-culture (0.18 ± 0.003 mmol) and R. cellulolyticum-M. hungatei bi-culture (0.42 ± 0.007 mmol), while the methane production of the quad-culture (1.37 ± 0.03 mmol) was higher than the combined methane production by the R. cellulolyticum, M. hungatei, and D. vulgaris tri-culture (0.89 ± 0.02 mmol) and the R. cellulolyticum, M. concilii, and D. vulgaris tri-culture (0.16 ± 0.001 mmol), hence indicating a positive synergy. Mechanistically, it may be that D. vulgaris enhances methanogenesis by producing the substrates CO_2_, acetate, and H_2_ and removing lactate (24, 25). The two methanogens can enhance each other’s methanogenesis by also consuming the substrates. The whole of these positively synergistic effects was greater than the sum of the individual effects on methanogenesis.

Synthetic communities can be modeled to estimate their intracellular metabolic fluxes and cross-feeding fluxes, which can be used to infer competitive and cooperative interactions among the organisms. Genome-scale flux balance analysis (FBA) models of individual organisms have been combined to construct FBA models of their cocultures (25–27). However, genome-scale FBA is vastly underparameterized and requires strong assumptions on the objective function for optimization of the fluxes in an organism (28), This is further complicated in a synthetic community with different organisms possibly requiring divergent objective functions to maximize the total fitness of the community. In addition, reliable FBA models require labor-intensive curation of the genome-scale metabolic networks of all members of a synthetic community. As a result, the previous studies focused on the synthetic communities of microorganisms with preexisting FBA models (29). Alternatively, a kinetic model of anaerobic digestion, ADM1 (30), models the major metabolic processes in the degradation of complex biomass (e.g., cellulose) to methane and CO_2_. However, the lack of knowledge of kinetic parameters in a study would limit the prediction accuracy of the ADM1 modeling (31). Here, we constructed a simple stochiometric model for synthetic communities. Similar to ADM1, the stochiometric modeling approach considers only the major metabolic processes of the organisms, but it enables quantifying metabolic fluxes without knowledge of substrate concentrations or kinetic parameters. Unlike a genome-scale FBA model, our simple stoichiometric model can be fitted just using the experimental data without assuming any objective function (32). These complementary modeling approaches could be combined to build a multiscale model for synthetic communities. Specifically, the organism-level boxes in a community-level stoichiometric model (Fig. 5) can be expanded to individual genome-scale FBA models for selected organisms of interest. The estimated fluxes by the multiscale flux balancing analyses can then be integrated with a parameterized kinetic model for each of the organisms to predict synthetic community dynamics and its responses to disturbances.

The stoichiometric model and metaproteomics analysis characterized the metabolic activities of microorganisms in two different ways, so we compared the fluxes through the modeled reactions with the protein abundances of marker enzymes in those reactions (see Table S3 in the supplemental material). The stoichiometric model showed that sulfate addition zeroed out the flux through hydrogenotrophic methanogenesis. This change was also reflected in the significantly decreased abundance of M. hungatei formate dehydrogenase, a marker enzyme for hydrogenotrophic methanogenesis. However, sulfate addition significantly increased the flux of acetoclastic methanogenesis in M. concilii, while the relative abundance of M. concilii methyl-coenzyme M reductase significantly decreased. In addition to the protein abundance of the corresponding enzyme, a reaction may also be regulated by a variety of other mechanisms, such as posttranslational modifications and allosteric regulations (17). Compared with the control condition, the accumulation of CH_4_ was reduced by only 12% in the sulfate addition condition, while the flux of hydrogenotrophic methanogenesis decreased to 0 mmol, so a 1.42-fold increase in acetoclastic methanogenesis flux should be reasonable.

10.1128/mbio.03189-22.5 TABLE S3 Comparison of stoichiometric fluxes and marker protein abundances. Download Table S3, PDF file, 0.3 MB. Copyright © 2023 Wang et al. 2023 Wang et al. https://creativecommons.org/licenses/by/4.0/ This content is distributed under the terms of the Creative Commons Attribution 4.0 International license.

The quad-culture and its model were used to study the effects of increased sulfate availability on the microbial communities. Such a perturbation may occur in estuarine wetland soil communities as a result of more prevalent sulfate-laden seawater intrusion from rising seawater levels. The impact of seawater intrusion has been studied using natural wetland ecosystems (33, 34), demonstrating that seawater intrusion reduced the CH_4_ and CO_2_ fluxes from the wetland. However, it was not clear how microbial interactions responded to seawater intrusion because of the great complexity of natural communities. In this study, we showed that sulfate addition reduced CH_4_ and CO_2_ emissions, consistent with results obtained with natural ecosystems (33, 35). The main reason for decreased CH_4_ production was the sharp decrease in H_2_ available for the hydrogenotrophic methanogen. The competition for H_2_ use between methanogens and sulfate reducers has been proposed to have a thermodynamic basis, as hydrogenotrophic sulfate reduction is more energetically favorable than hydrogenotrophic methanogenesis (36). Therefore, such competitions exist in reactor systems and environments with transient sulfate (37, 38). In addition, possible interactions can be estimated by modeling what is consumed and produced by the individual species. Our result demonstrated that sulfate addition increased both the production of acetate and lactate by R. cellulolyticum and their consumption by M. concilii and D. vulgaris. This suggested that sulfate addition intensified the carbon transfer from R. cellulolyticum to M. concilii and D. vulgaris. Although the flux of H_2_ production by R. cellulolyticum increased, the flux of H_2_ consumed to produce H_2_S in D. vulgaris increased even more. This, in turn, further intensified the competitive interactions between M. hungatei and D. vulgaris for H_2_ as the substrate in the presence of sulfate.

Many syntrophic communities have been shown to develop around the exchange of H_2_ and organic acids (9, 39). In this study, our stoichiometric analysis was unable to constrain the possible exchange of lactate or H_2_ between R. cellulolyticum and D. vulgaris, even in the absence of sulfate (Table S3). However, limited changes in hydrogenase abundance were observed in both R. cellulolyticum and D. vulgaris, indicating a likely exchange of lactate or some other oxidizable carbon intermediate. While the oxidation of lactate to acetate and production of H_2_ is expected to be unfavorable under the H_2_ partial pressures studied, alternative organic compounds could be intermediates, including pyruvate or alanine, which have been demonstrated to produce H_2_ in sulfate-reducing bacterium at high H_2_ partial pressures (9). Furthermore, D. vulgaris was observed in both the sulfate-replete and -deplete conditions, indicating growth under both conditions and, therefore, at least a partial exchange of lactate or another metabolite to support D. vulgaris growth. In the presence of sulfate, the lactate dehydrogenase in R. cellulolyticum also increased, indicating a possible increased participation of the lactate exchange in the flux distributions (Table S3). While D. vulgaris should outcompete M. hungatei for H_2_ in the sulfate addition condition, the relative total protein abundance of M. hungatei remained at 3% in both conditions. This could be explained if D. vulgaris had a long lag phase in the quad-culture and M. hungatei entered the stationary phase before being outcompeted by D. vulgaris. Alternatively, M. hungatei might employ direct electron transfer using its electrogenic pili (40) or utilize other compounds, such as formate (41), as the electron donor.

We anticipate that this four-species community, as well as the future construction of additional multispecies synthetic communities, introduces the level of complexity needed to more realistically model carbon cycling in natural anaerobic communities. By combining metabolic profiling, metaproteomics, and modeling, we observed many community behaviors as designed, especially the adaptive cross-feeding fluxes and the shifting substrate competitions under changing environments. More interestingly, the multispecies cultures also revealed some emergent properties of microbial communities, including synergistic epistasis and antagonistic epistasis of tertiary interactions. By quantifying the expected competitive/cooperative behaviors and revealing the emergent interaction properties, the synthetic communities may pave the way toward more accurate modeling of natural communities.

## MATERIALS AND METHODS

### Strains.

The strains used in this study included Ruminiclostridium cellulolyticum H10 (ATCC 35319), Desulfovibrio vulgaris Hildenborough (ATCC 29579), Methanospirillum hungatei JF1 (txid323259), and Methanosaeta concilii (txid990316). Cultures of R. cellulolyticum H10 and D. vulgaris Hildenborough were stored in 17%(vol/vol) glycerol at −80°C, while the methanogens were maintained in a defined medium, which is referred to as RST (42) here, at room temperature.

### Cultures, growth conditions, and experimental design.

Four strains were revived by respective media and conditions commonly published in previous studies. Briefly, modified VM medium supplemented with 2.0g/L yeast extract and LS4D medium were used for R. cellulolyticum H10 and Desulfovibrio vulgaris Hildenborough, respectively, and both were cultured at 34°C (43, 44). M. hungatei JF1 and M. concilii were grown in RST medium with H_2_/CO_2_ (80:20) in the headspace and 50 mM acetate at 37°C (45). M. concilii required about 5 weeks before noticeable growth and acclimation of acetate occurred. Subsequently, active inocula (optical density at 600 nm [OD_600_] = 0.5) were transferred to the modified VM medium with 10 g/L cellulose as the sole carbon source for experiments (44).

All fermentation experiments were carried out in 160 mL serum bottles with 20 mL of modified VM medium containing 10 g/L cellulose; the chamber gas contained 3% H_2_ and 97% N_2_. The modified VM medium contained 1.25 mg/L of FeSO_4_·6H_2_O, which introduced approximately 0.14 μmol sulfate to the control condition. Revived cells were counted by flow cytometry; the number of inoculated cells per species was about 5 × 10^7^ for all mixed cultures. All cultures were incubated at 34°C with shaking at 200 rpm for 7 days and sampled each day.

During the sampling, 2 mL of well-shaken culture liquid was sampled, centrifuged at 14,000 rpm for 15 min to separate the cells and cellulose from the supernatant, and stored at −80°C for later analysis. We collected 5 mL gas phase products in vacuumed Labco exetainer glass vials (catalog no. 837w) for later analysis.

After sampling, 2 mL fresh medium with 10 g/L cellulose was added to the cultures, pH was adjusted to 7.3, and produced gases were vented to restore serum bottle headspace to atmospheric pressure. To mimic seawater intrusion, sulfate-treated experiments received 2 mL of 100 mM K_2_SO_4_ solution every day from the first day of cultivation, and the control group received the same volume of sterile water.

### Chemical analysis.

Cellulose in the medium was measured by the phenol-sulfuric acid method as described previously (46). Headspace pressure was measured daily with a pressure gauge (Cole Parmer; catalog no. SK-68900-24) before sampling liquid and gas phases. Gas composition (H_2_, CO_2_, and CH_4_) was analyzed from the stored Labco vacuum containers with a gas chromatograph (GC-8A; Shimadzu, Japan). The absolute gas composition in each bottle was calculated with the ideal gas law. The remaining sulfate in the medium was measured by ion chromatography (IC) and used to estimate H_2_S production in the cultures. The fermentation products (lactate and acetate) in liquid phase and soluble sugars (cellobiose and glucose) were analyzed by high-performance liquid chromatography (HPLC) as reported previously (47).

### Protein identification and quantification.

Samples from quad-cultures were selected for global metaproteomic analysis. Protein extraction was carried out according to a previously reported method (48, 49). Briefly, harvested cell pellets were washed twice with Nanopure water, cells were suspended in lysis buffer (containing 10 mM Tris-HCl, 1% SDS, and 0.1 M dithiothreitol [DTT]), and incubated at 60°С for 1 h, and then the supernatant was collected after centrifugation. Proteins were then precipitated by trichloroacetic acid overnight at 4°C and pelleted by centrifugation. Protein pellets were washed with ice-cold acetone three times and resuspended in guanidine buffer. Protein concentrations were quantified by bicinchoninic acid assay (50). Twenty-milligram samples of proteins were processed using filter-aided sample preparation and digested using trypsin-LysC mixture. Each sample was analyzed using two-dimensional liquid chromatography-tandem mass spectrometry (2D-LC-MS/MS) on an Orbitrap Fusion Tribrid mass spectrometer (Thermo Fisher Scientific, USA) at the IDeA National Resource for Quantitative Proteomics. Tryptic peptides were separated into 46 fractions on a 100- by 1.0-mm Acquity BEH C_18_ column (Waters) with a 50-min gradient from 99:1 to 60:40 buffer ratio under basic pH conditions and then consolidated into 8 superfractions. Each superfraction was then separated by reverse phase XSelect CSH C_18_ 2.5-μm resin (Waters) on an in-line 120- by 0.075-mm column using an UltiMate 3000 RSLCnano system (Thermo Fisher Scientific, USA). Peptides were eluted using a 60-min gradient from 98:2 to 65:35 buffer ratio. Eluted peptides were ionized by electrospray (2.4 kV) followed by mass spectrometric analysis on an Orbitrap Fusion Tribrid mass spectrometer (Thermo Fisher Scientific, USA). MS data were acquired using a Fourier transform MS (FTMS) analyzer in profile mode at a resolution of 240,000 over a range of 375 to 1,500 *m/z*. Following high-energy collisional dissociation (HCD) activation, MS/MS data were acquired using the ion trap analyzer in centroid mode and normal mass range with normalized collision energy of 28 to 31% depending on charge state and precursor selection range. Mass spectrometry spectra were searched using Sipros Ensemble against a matched protein database constructed from the genomes of the four microorganisms making up the synthetic community (51, 52). Raw search results were filtered to achieve a 1% false-discovery rate (FDR) at the peptide level, estimated by the target-decoy approach. Peptide identifications are assigned to proteins or protein groups following the parsimonious rule (51). A protein group included multiple proteins sharing a set of common peptides, at least one of which cannot be attributed to any other protein or protein group. A minimum of one unique peptide was required for each identified protein or protein group. To avoid ambiguity in data analysis, only proteins were used for biological analysis, while protein groups were not evaluated. Intensity-based label-free quantification for protein was performed using ProRata (53, 54). The protein abundance was represented by the total peak height of all quantified unique peptides from a protein (17).

### Construction of the stoichiometric model.

A set of overall reactions representing the documented metabolisms of the individual species were fit into the daily measured amounts of metabolites in the constructed synthetic communities (Tables S1 and S2). The metabolism of R. cellulolyticum was modeled to consume glucose to eliminate artifacts from cellulase production. The overall metabolism of R. cellulolyticum was modeled as lactate fermentation, hydrogenic acetogenesis. The low observed ethanol in ethanol/acetate fermentations, accounting for less than 0.36% of the carbon flux, led us to remove this pathway from analysis. D. vulgaris was modeled to oxidize lactate to acetate, coupled with reduction of protons or sulfate, or oxidize H_2_ coupled to sulfate reduction. The methanogens were represented by hydrogenotrophic methanogenesis and acetoclastic methanogenesis. The code used for the fitting can be found at GitHub (https://github.com/thepanlab/Community_stoichiometric_model). The resulting fits indicate the relative contribution of each microorganism to the overall observed transformations and provide a basis for quantifying metabolic contributions. Deviations between the inferred metabolic transformations and the measured transformations indicate uncertainty in our understanding of the metabolic transformations or limitations in analytical capacity.

### Statistical analysis.

Metabolic measurement data were compared between different conditions using Student’s *t* test. The differences in protein abundance in metaproteome were analyzed by the DeSeq R package (55), and the *P* values were adjusted to *q* values using the Benjamini-Hochberg method for multiple-comparison correction (55). Proteins with *q* values of <0.05 were regarded as statistically significant.

### Data availability.

Proteomic data are available at the ProteomeXchange Consortium via the Proteomics Identification Database (PRIDE) partner repository with the data set identifier PXD035759.
