# Supplementary material for: Cross-Feedings, Competition, and Positive and Negative Synergies in a Four-Species Synthetic Community for Anaerobic Degradation of Cellulose to Methane
Source: mBio. 2023 Feb 27;14(2):e03189-22. doi: 10.1128/mbio.03189-22 (PMC10128006; doi:10.1128/mbio.03189-22)
Supplement: TABLE S1 [file mbio.03189-22-s0003.pdf]

Measured data		Overall catabolic reaction stoichiometries							Fit of measurements	
		R. cellulolyticum		*D. vulgaris*			*M. hungatei*	*M. concilii*	Sum of contributions	
Metabolites	mmoles accumulated	Lactate fermentation	Hydrogenic acetogenesis	Hydogenic lactate oxidation	Sulfidogenic lactate oxidation	Sulfidogenic hydrogen oxidation	Hydrogenotrophic methanogenesis	Acetotrophic methanogenesis	Excluding Hydrogenic acetogenesis	Excluding Hydogenic lactate oxidation
Glucose	-1.17	-1	-1	0	0	0	0	0	-0.90	-0.90
Lactate	0.13	2	0	-1	-1	0	0	0	0.22	0.22
Acetate	0.55	0	2	1	1	0	0	-1	0.62	0.62
Hydrogen	1.30	0	4	2	0	-2	-4	0	1.31	1.31
Carbon Dioxide	1.28	0	2	1	1	0	-1	1	2.07	2.07
Methane	1.37	0	0	0	0	0	1	1	1.42	1.42
Sulfide	0.00	0	0	0	0.5	0.5	0	0	0.00	0.00
									$\sum(x_{measured} - x_{fit})^2$	
Flux through each reaction	Scenario 1	0.90	0.00	1.57	0.00	0.00	0.46	0.96	0.71	
	Scenario 2	0.11	0.79	0.00	0.00	0.00	0.46	0.96		0.71
