# Supplementary material for: Cross-Feedings, Competition, and Positive and Negative Synergies in a Four-Species Synthetic Community for Anaerobic Degradation of Cellulose to Methane
Source: mBio. 2023 Feb 27;14(2):e03189-22. doi: 10.1128/mbio.03189-22 (PMC10128006; doi:10.1128/mbio.03189-22)
Supplement: TABLE S2 [file mbio.03189-22-s0004.pdf]

Measured data

Metabolites	mmoles accumulated
Glucose	-1.14
Lactate	0.23
Acetate	0.49
Hydrogen	1.07
Carbon Dioxide	1.00
Methane	1.21
Sulfide	1.17

Overall catabolic reaction stoichiometries

	R. cellulolyticum		*D. vulgaris*			*M. hungatei*	*M. concilii*
	Lactate fermentation	Hydrogenic acetogenesis	Hydogenic lactate oxidation	Sulfidogenic lactate oxidation	Sulfidogenic hydrogen oxidation	Hydrogenotrophic methanogenesis	Acetotrophic methanogenesis
Glucose	-1	-1	0	0	0	0	0
Lactate	2	0	-1	-1	0	0	0
Acetate	0	2	1	1	0	0	-1
Hydrogen	0	4	2	0	-2	-4	0
Carbon Dioxide	0	2	1	1	0	-1	1
Methane	0	0	0	0	0	1	1
Sulfide	0	0	0	0.5	0.5	0	0
Flux through each reaction – Scenario 1	1.12	0.00	2.03	0.00	1.55	0.00	1.38
Flux through each reaction – Scenario 2	0.87	0.24	0.00	1.55	0.00	0.00	1.38
Flux through each reaction – Scenario 3	0.09	1.02	0	0	1.55	0	1.38

Fit of measurements

	Sum of contributions		
	Excluding lactate fermentation	Excluding sulfidogenic lactate oxidation	Excluding hydrogenic acetogenesis
Glucose	-1.11	-0.87	-0.098
Lactate	2.03	0.00	0
Acetate	0.00	0.24	1.02
Hydrogen	0.00	1.55	0
Carbon Dioxide	1.55	0.00	1.55
Methane	0.00	0.00	0
Sulfide	1.38	1.38	1.38
$\sum(x_{measured} - x_{fit})^2$	6.44	2.93	4.38
