# Supplementary material for: Cross-Feedings, Competition, and Positive and Negative Synergies in a Four-Species Synthetic Community for Anaerobic Degradation of Cellulose to Methane
Source: mBio. 2023 Feb 27;14(2):e03189-22. doi: 10.1128/mbio.03189-22 (PMC10128006; doi:10.1128/mbio.03189-22)
Supplement: TABLE S4 [file mbio.03189-22-s0006.pdf]

ProteinID	Annotation	Peak Height						*q*- value	Fold Change	Change
		Control condition			Sulfate addition condition					
		Rep_1	Rep_2	Rep_3	Rep_1	Rep_2	Rep_3			
B8HZS3	RUMCH Uncharacterized protein	124711875	30218161	80568312	33285952	66003537	38336788	0.5467	1.7	Null
B8HZS6	RUMCH Redox-active disulfide protein 2	12213831	885857	6935197	5874367	228254	321313	0.3932	3.1	Null
B8HZS7	RUMCH Protein tyrosine phosphatase	4136943	11053394	0	0	0	1975132	0.6115	7.7	Null
B8HZT0	RUMCH Nickel-dependent hydrogenase large subunit	0	0	123300	242827	595593	0	0.5377	6.8	Null
B8HZT2	RUMCH Cupin 2 conserved barrel domain protein	33186283	31461937	34178910	30052778	22659369	13998578	0.4142	1.5	Null
B8HZT4	RUMCH Basic membrane lipoprotein	0	0	0	293463	239213	0	0.2245	NA	Null
B8HZT8	RUMCH Isochorismatase hydrolase	0	78462	0	0	0	0	NA	NA	Null
B8HZU2	RUMCH Iron-containing alcohol dehydrogenase	0	0	0	71017	0	0	NA	NA	Null
B8HZU4	RUMCH Uncharacterized protein	285073	0	0	48528	40704	70275	0.8463	1.8	Null
B8HZU6	RUMCH DUF43 domain-containing protein	6255948	1172268	2506247	8906238	7207942	3840000	0.4912	2.0	Null
B8HZU7	RUMCH GCN5-related N-acetyltransferase	0	1897389	0	0	921347	0	NA	2.1	Null
B8HZV0	RUMCH dTDP-glucose 4,6-dehydratase	507380	187790	0	3055301	1716194	313726	0.2575	7.3	Null
B8HZV1	RUMCH S-adenosylmethionine decarboxylase proenzyme	14159282	12906658	24239523	11588043	10260437	440884	0.4624	2.3	Null
B8HZV2	RUMCH Zn-dependent hydrolase of the beta-lactamase fold protein	83015756	249207153	418712961	360868143	238302136	211974594	0.8427	1.1	Null
B8HZV3	RUMCH Methyladenine glycosylase	0	266094	0	410643	232868	0	0.8206	2.4	Null
B8HZV4	RUMCH Mtd	30438870146	54650212966	25125491425	20011369593	9323941518	21966447307	0.2917	2.1	Null
B8HZV6	RUMCH Kelch repeat-containing protein	11901967021	14502228099	12711374039	14578890097	9900606035	10090945120	0.8824	1.1	Null
B8HZV7	RUMCH Methionine--tRNA ligase	10672396	21964155	10220438	10960971	11517782	9164657	0.7043	1.4	Null
B8HZV9	RUMCH Uncharacterized protein	3023995	0	725318	14749805	14136378	9369216	0.1838	10.2	Null
B8HZW0	RUMCH Cellulase	4978365655	5721727516	4014061178	3411214945	3134794932	4173920330	0.6546	1.4	Null
B8HZW1	RUMCH 4Fe-4S ferredoxin iron-sulfur binding domain protein	265189	389387	274937	2529733	3968145	133073	0.0611	7.1	Null
B8HZW2	RUMCH Transcriptional regulator, PadR-like family	186994	0	0	0	0	0	NA	NA	Null
B8HZW3	RUMCH Uncharacterized protein	1004549	0	522979	0	214910	203901	0.6304	3.6	Null
B8HZW4	RUMCH Tail Collar domain protein	11735222	19659466	25483872	11387766	4767068	4921153	0.0752	2.7	Null
B8HZW5	RUMCH Tail Collar domain protein	668881	2090042	1288158	1024217	916946	0	0.7087	2.1	Null
B8HZW6	RUMCH NADH:flavin oxidoreductase/NADH oxidase	4252045	16705439	7729780	4953456	2534086	5292029	0.3312	2.2	Null
B8HZW7	RUMCH DUF1858 domain-containing protein	1924546	3662402	10418072	3210144	3261295	2627179	0.5812	1.8	Null
B8HZW8	RUMCH Uncharacterized protein	0	0	0	0	2788942	0	NA	NA	Null
B8HZW9	RUMCH Glycoside hydrolase family 18	1700476	4402877	133455	1486651	3059726	5153069	0.6875	1.6	Null
B8HZX4	RUMCH Uncharacterized protein	3284564	4149002	4233760	2989644	1895801	1864778	0.2083	1.7	Null
B8HZX5	RUMCH FAD:protein FMN transferase	0	0	0	236098	227296	209059	0.0049	NA	Increase
B8HZX7	RUMCH Biosynthetic arginine decarboxylase	4755030	7773723	5560004	9683137	5366414	4398776	0.8463	1.1	Null
B8HZY3	RUMCH Oxidoreductase domain protein	5387552	9608290	21164695	7250804	7356478	4070736	0.3812	1.9	Null
B8HZY4	RUMCH Copper amine oxidase domain protein	32686919	37947599	52759217	64670698	72028808	69572599	0.1121	1.7	Null
B8HZY6	RUMCH Saccharopine dehydrogenase	6119255	4632002	5286929	11260288	7597901	4592325	0.4791	1.5	Null
B8HZY7	RUMCH Carboxynorspermidine/carboxyspermidine decarboxylase	1651759	640454	929267	0	0	0	0.0000	NA	Decrease
B8HZY8	RUMCH Uncharacterized protein	159110	298393	124994	0	0	0	0.0200	NA	Decrease
B8HZZ0	RUMCH Extracellular solute-binding protein family 1	17104971889	18868368682	25039749112	28207387441	18608486760	17085987531	0.8395	1.0	Null
B8HZZ2	RUMCH Binding-protein-dependent transport systems inner membrane cc	0	0	0	134250	0	0	NA	NA	Null
B8HZZ3	RUMCH Extracellular solute-binding protein family 1	320734	1984820	756243	1179273	1073104	400763	0.9658	1.2	Null
B8HZZ4	RUMCH Putative sensor with HAMP domain	176150	1759763	937834	0	148280	200433	0.2285	8.2	Null
B8HZZ5	RUMCH Stage 0 sporulation protein A homolog	0	44171	114771	114811	210294	69606	0.5467	2.5	Null
B8HZZ6	RUMCH Glycoside hydrolase family 3 domain protein	3310812	21230282	3102640	2603765	1819123	687820	0.0779	5.4	Null
B8HZZ7	RUMCH Methylated-DNA--protein-cysteine methyltransferase	8942791	187342	85527074	218010	264400	0	0.0013	196.2	Decrease
B8HZZ8	RUMCH Uncharacterized protein	194237	0	419371	0	85110	54124	0.6048	4.4	Null
B8HZZ9	RUMCH Radical SAM domain protein	0	438094	0	664916	502582	0	0.7930	2.7	Null
B8I000	RUMCH Transcriptional regulator, ArsR family	42064	0	0	0	0	0	NA	NA	Null
B8I001	RUMCH FAD-dependent pyridine nucleotide-disulphide oxidoreductase	9655521	31300104	24351773	18694866	66591855	25431340	0.4080	1.7	Null
B8I003	RUMCH Copper amine oxidase domain protein	83423860	43490537	15782064	5416361	16153394	28891897	0.3325	2.8	Null
B8I004	RUMCH Undecaprenyl-diphosphatase	0	0	0	0	2129058	0	NA	NA	Null
B8I006	RUMCH Methyltransferase type 11	1560478	802244	0	8824450	18235433	3412167	0.1165	12.9	Null
B8I007	RUMCH Plasmid maintenance system antidote protein, XRE family	127500868	95783831	95634333	122330978	66975369	37639038	0.6089	1.4	Null
B8I008	RUMCH ABC transporter related	27342152	196649224	84630885	28296539	24777703	123246140	0.7969	1.8	Null
B8I009	RUMCH Uncharacterized protein	1133625	1393390	1104297	2180058	896446	2187181	0.5045	1.4	Null
B8I010	RUMCH Stage 0 sporulation protein A homolog	308400	0	636954	202935	2882322	1443366	0.3308	4.8	Null
B8I011	RUMCH Histidine kinase	0	0	0	0	0	72331	NA	NA	Null
B8I012	RUMCH PfkB domain protein	21224097	11082443	8721553	14054640	10536116	12324984	0.9167	1.1	Null
B8I013	RUMCH Periplasmic solute binding protein	62337479	244004620	146790919	286664402	77016828	237683954	0.6538	1.3	Null
B8I014	RUMCH Stage 0 sporulation protein A homolog	0	0	0	94246	242300	264292	0.0144	NA	Increase
B8I015	RUMCH Histidine kinase	0	0	0	55944	0	68081	NA	NA	Null
B8I018	RUMCH ABC transporter related	0	240142	444281	611318	594562	1194251	0.3244	3.5	Null
B8I019	RUMCH Transposase IS3/IS911 family protein	0	0	0	45793	48727	0	NA	NA	Null
B8I021	RUMCH Pyruvate flavodoxin/ferredoxin oxidoreductase domain protein	3861561648	5290352683	2646379627	4057835786	4675588893	3602952518	0.8698	1.0	Null
B8I024	RUMCH Stage 0 sporulation protein A homolog	0	0	0	157309	0	0	NA	NA	Null
B8I025	RUMCH Rubrerythrin	1006282	2852029	5769608	935321	765714	1433007	0.2316	3.1	Null
B8I027	RUMCH Uncharacterized protein	123677518	129181219	170414637	28696575	23417490	27551798	0.0000	5.3	Decrease
B8I030	RUMCH Short-chain dehydrogenase/reductase SDR	213910034	105676102	96075410	33536584	32210108	20710938	0.0042	4.8	Decrease
B8I032	RUMCH Transcriptional regulator, PadR-like family	0	0	3231936	2471739	0	2038438	NA	1.4	Null
B8I034	RUMCH ABC transporter related	35927185	183879624	41807671	20026070	6316134	20428490	0.0576	5.6	Null
B8I035	RUMCH ABC-type Na+ efflux pump permease component-like protein	5595348	7706377	3238486	15137704	12251091	4333055	0.3199	1.9	Null
B8I036	RUMCH Mannose-6-phosphate isomerase	322418	3619538	10958069	5139519	2135284	2156369	0.8174	1.6	Null
B8I037	RUMCH ABC transporter related	0	2228329	2940164	8372834	160171	6773965	0.5554	3.0	Null
B8I039	RUMCH Cobalamin (Vitamin B12) biosynthesis CbiM protein	21669700	28120367	40264541	31105617	30155433	27069438	0.9007	1.0	Null
B8I041	RUMCH Cobalt ABC transporter, ATPase subunit	116022313	1249650	380103	13086211	3050405	0	NA	7.3	Null
B8I042	RUMCH Uncharacterized protein	2400085	1312728	1534676	2135543	1830999	2570204	0.7341	1.2	Null
B8I043	RUMCH Uncharacterized protein	2386681	1202415	2775017	1945136	3146126	3456481	0.6330	1.3	Null
B8I045	RUMCH Aminotransferase class V	35908774	70874074	122873069	303983594	397497971	254071960	0.0018	4.2	Increase
B8I046	RUMCH RNA binding S1 domain protein	25922492	73314578	43067284	27838263	16749325	20565257	0.1984	2.2	Null
B8I047	RUMCH Septum formation initiator	20362640	36138057	23551133	23261	429621	0	0.0000	176.8	Decrease
B8I049	RUMCH Sporulation protein YabP	18123749	14087010	51818947	67758843	57409869	79811811	0.1432	2.4	Null
B8I050	RUMCH RNA-binding S4 domain protein	113939796	16333241	43403794	1046841	9311766	13382329	0.0870	7.3	Null
B8I051	RUMCH Histone family protein DNA-binding protein	134999948	60488598	212348763	47110821	43073734	5339814	0.1058	4.3	Null
B8I052	RUMCH MazG family protein	7526817	13698213	25770868	4904329	2379630	7730107	0.1949	3.1	Null
B8I053	RUMCH Single-stranded DNA-binding protein	1338904	257999	0	0	335625	702691	0.8997	1.5	Null
B8I054	RUMCH Transcriptional regulator, AbrB family	115040215	135311267	135218564	59273039	50335028	54577523	0.0061	2.3	Decrease
B8I055	RUMCH PpiC-type peptidyl-prolyl cis-trans isomerase	20483932793	59303219499	36835479436	9188244957	8696019993	6966042383	0.0004	4.7	Decrease
B8I056	RUMCH Transcription-repair-coupling factor	2524648	1551670	2344917	2200446	3478864	4332890	0.4359	1.6	Null
B8I057	RUMCH Peptidyl-tRNA hydrolase	6697907	149907163	75380941	67105417	77424301	57716842	0.9962	1.1	Null
B8I058	RUMCH Ribose-phosphate pyrophosphokinase	41883328	36367203	25539727	115429951	53539885	59089010	0.1464	2.2	Null
B8I059	RUMCH UDP-N-acetylglucosamine pyrophosphorylase / glucosamine-1-ph	33619240	11931772	6901636	11560046	5939818	5556748	0.3084	2.3	Null
B8I060	RUMCH Putative septation protein SpoVG	4200577	4216390	8546786	3641956	3404456	3374184	0.4339	1.6	Null
B8I061	RUMCH Pur operon repressor	3616118	4736722	5801458	10875444	3520771	109622	0.9962	1.0	Null
B8I062	RUMCH UDP-N-acetylmuramate--L-alanine ligase	6592112	504269	1231529	7315697	1718547	1037560	0.9728	1.2	Null
B8I063	RUMCH 3-hydroxyacyl-[acyl-carrier-protein] dehydratase FabZ	3344324	0	0	1282118	28498485	30834312	0.2650	18.1	Null
B8I065	RUMCH Rod-binding domain-containing protein	0	85325	0	0	0	0	NA	NA	Null
B8I066	RUMCH Flagellar basal body protein	1206518	1310721	522153	695054	653212	0	0.6359	2.3	Null
B8I067	RUMCH Flagellar basal body protein	0	800703	0	676706	1931180	171576	0.6002	3.5	Null
B8I068	RUMCH Cell shape-determining protein MreB	333281559	49449132	80853469	132310012	119526659	53706415	0.6623	1.5	Null
B8I069	RUMCH Small GTP-binding protein	2829015	984309	3484620	3610992	1635648	2400905	0.9428	1.0	Null
B8I070	RUMCH Biotin and thiamin synthesis associated	6966671	11169897	8043690	2712753	3887573	2630126	0.0105	2.8	Decrease
B8I071	RUMCH Uncharacterized protein	0	0	0	127893	61715	0	NA	NA	Null
B8I074	RUMCH Sodium/hydrogen exchanger	503072	0	0	0	0	0	0.2316	NA	Null
B8I075	RUMCH TrkA-C domain protein	0	808593	325645	4881909	5812010	3564100	0.0404	12.6	Increase
B8I076	RUMCH Resolvase domain protein	0	0	0	0	154759	0	NA	NA	Null
B8I080	RUMCH Transcriptional regulator, XRE family	1856739	9312526	16446862	7087884	8760421	4200485	0.8496	1.4	Null
B8I081	RUMCH Stage 0 sporulation protein A homolog	109474	0	207993	98109	724195	409190	0.3922	3.9	Null
B8I085	RUMCH CRISPR-associated endonuclease Cas9	16640663	8815523	4802184	8323479	6672865	4604190	0.5796	1.5	Null
B8I087	RUMCH Uncharacterized protein	6724963845	4873732612	11538201266	21278769975	2466240199	1244405787	0.9730	1.1	Null

B8I089	RUMCH Uncharacterized protein	0	0	0	114750	0	0	NA	NA	Null
B8I091	RUMCH Peptidase M23	0	0	6643425	0	0	227381	NA	29.2	Null
B8I095	RUMCH Transcriptional regulator, AbrB family	332243	110846	111780	0	0	0	0.0274	NA	Decrease
B8I096	RUMCH PKD domain-containing protein	0	0	0	221370	86699	0	0.2925	NA	Null
B8I097	RUMCH S-layer domain protein	812802948	108156113	133930653	156306328	149976150	139400821	NA	2.4	Null
B8I0A0	RUMCH Uncharacterized protein	0	0	0	22640	0	0	NA	NA	Null
B8I0A6	RUMCH Uncharacterized protein	553271	239774	532529	312172	108325	189173	0.3931	2.2	Null
B8I0B2	RUMCH Uncharacterized protein	0	365297	0	0	93453	0	0.7889	3.9	Null
B8I0B9	RUMCH Uncharacterized protein	0	158230	566341	993261	72735	0	0.9006	1.5	Null
B8I0C0	RUMCH Uncharacterized protein	290699	385868	1162528	2193451	1420589	473980	0.3252	2.2	Null
B8I0C7	RUMCH Ankyrin	0	300437	280494	0	0	92030	0.6655	6.3	Null
B8I0C9	RUMCH Cobyrinic acid ac-diamide synthase	0	0	171259	138197	0	0	0.9743	1.2	Null
B8I0E2	RUMCH Helicase domain protein	0	15625	5633445	0	0	0	NA	NA	Null
B8I0E4	RUMCH Putative transcriptional regulator, TetR family	0	425293	374911	321418	0	336150	0.9972	1.2	Null
B8I0E5	RUMCH Glycosyltransferase, MGT family	0	882995	0	254036	0	0	NA	3.5	Null
B8I0E6	RUMCH Uncharacterized protein	1552816182	1245265658	3117597620	3166163864	2354672557	896689662	0.8990	1.1	Null
B8I0E7	RUMCH Seryl-tRNA synthetase	28800442	51951637	10924059	795001	1463529	5644586	0.0144	11.6	Decrease
B8I0E8	RUMCH Metal dependent phophohydrolase	926535	1867367	2846739	1021479	795252	246199	0.2055	2.7	Null
B8I0E9	RUMCH Aminotransferase class I and II	234086	0	0	0	815516	211232	0.7087	4.4	Null
B8I0F0	RUMCH Cysteine synthase	1003142069	971536463	1761009207	674050928	705177614	1681085678	0.9455	1.2	Null
B8I0F1	RUMCH DNA/pantothenate metabolism flavoprotein domain protein	0	0	688517	705114	147060	40278	0.9265	1.3	Null
B8I0F2	RUMCH Flavoprotein	3776083	4616985	7159637	7381488	5086157	7457390	0.5217	1.3	Null
B8I0F3	RUMCH Radical SAM domain protein	4987814	3298368	3905256	3397973	3083496	2319371	0.5869	1.4	Null
B8I0F4	RUMCH bPH	2285011	3690375	5267973	2451956	1751240	3227827	0.6590	1.5	Null
B8I0F5	RUMCH PfkB domain protein	532248	0	0	1654657	4040230	192759	0.3222	11.1	Null
B8I0F6	RUMCH 2-dehydro-3-deoxyphosphogluconate aldolase/4-hydroxy-2-oxogl	0	0	0	117966	207522	609808	0.0041	NA	Increase
B8I0F9	RUMCH Uncharacterized protein Ccel	396087652	724846176	183755282	1199256491	1752269063	1170559859	0.0533	3.2	Increase
B8I0G0	RUMCH Peptidase S8 and S53 subtilisin kexin sedolisin	3000968311	833387885	190397749	34287842	25507054	27652725	0.0000	46.0	Decrease
B8I0G1	RUMCH Uncharacterized protein	2288482946	1888148976	1289029638	1040543739	1375457521	640587642	0.3130	1.8	Null
B8I0G2	RUMCH Aldehyde-alcohol dehydrogenase	56601450482	90118891528	102331349476	99946886290	98933127158	81481496517	0.6002	1.1	Null
B8I0G3	RUMCH Methionine synthase B12-binding module cap domain protein	156354933	141386967	176583510	96928854	46378654	57035005	0.0645	2.4	Null
B8I0G5	RUMCH Ferredoxin	337254	796404	765666	1673048	1246703	437975	0.3911	1.8	Null
B8I0G6	RUMCH ABC transporter related	0	359741	0	103756	0	61188	0.8799	2.2	Null
B8I0G8	RUMCH Periplasmic binding protein	292955127	230823701	47304459	47111756	85797129	19901595	0.1492	3.7	Null
B8I0G9	RUMCH Phosphopentomutase	15178849	16552289	23094306	3247277	5078754	13986851	0.3527	2.5	Null
B8I0H0	RUMCH CapA domain protein	126280	1435921	548818	532035	1333206	1607120	0.5557	1.6	Null
B8I0H1	RUMCH D-3-phosphoglycerate dehydrogenase	303947379	336026117	240341387	817655497	932109665	446710840	0.0273	2.5	Increase
B8I0H2	RUMCH Transcriptional regulator, MarR family	0	0	0	0	602087	0	0.2184	NA	Null
B8I0H3	RUMCH ABC transporter related	53075268	83047476	58074326	87236728	67340177	38808812	0.9470	1.0	Null
B8I0H4	RUMCH ABC transporter related	0	2842605	2874231	4507439	23741859	10283762	0.2217	6.7	Null
B8I0H5	RUMCH Uncharacterized protein	21029097	29871471	42447732	5462934	2522345	3519787	0.0000	8.1	Decrease
B8I0I1	RUMCH Valine--tRNA ligase	62292081	40582184	20214320	14671862	21303394	23051668	0.3294	2.1	Null
B8I0I2	RUMCH Glutamine-dependent NAD(+) synthetase	349033	3015246	369451	2310772	2023682	1297763	0.6546	1.5	Null
B8I0I3	RUMCH Diadenylate cyclase	2963204	5118538	16467185	5260780	6580160	1987793	0.6002	1.8	Null
B8I0I4	RUMCH YbbR family protein	53908887	114362272	65690444	91132182	70420385	64868310	0.9419	1.0	Null
B8I0I5	RUMCH FAD dependent oxidoreductase	30396034	7443924	11061172	10953154	12974758	14597686	0.8262	1.3	Null
B8I0I6	RUMCH Phosphoglucosamine mutase	88096091	208923758	84352025	25184769	27012965	29744675	0.0031	4.7	Decrease
B8I0I7	RUMCH Glutamine--fructose-6-phosphate aminotransferase [isomerizing]	555292	4422280	954707	1998378	109406	2790518	0.9940	1.2	Null
B8I0I9	RUMCH Cellulase	1012288453	1311746113	1173626278	2224419247	2074149443	1081016747	0.2467	1.5	Null
B8I0J2	RUMCH Uncharacterized protein	0	0	0	128871	0	36000	NA	NA	Null
B8I0J3	RUMCH Thioesterase superfamily protein	649811	955138	0	719978	55668	0	0.8009	2.1	Null
B8I0J5	RUMCH Condensation domain protein	0	392708	86092	0	0	0	0.2633	NA	Null
B8I0J6	RUMCH Short-chain dehydrogenase/reductase SDR	2024818	1631927	1874146	1003787	1113082	611084	0.1821	2.0	Null
B8I0J7	RUMCH Beta-ketoacyl-[acyl-carrier-protein] synthase III	1802359	6971858	8587870	2544166	2671877	3308054	0.4322	2.0	Null
B8I0K0	RUMCH Phenylacetate-coenzyme A ligase	452310	836724	274448	717522	738878	995967	0.4772	1.6	Null
B8I0K1	RUMCH Integrase catalytic region	605607	957409	6153010	1229938	1090427	316224	0.3521	2.9	Null
B8I0K2	RUMCH Alpha-L-arabinofuranosidase domain protein	2182734	2993538	3182734	2027316	10545342	396528	0.7237	1.6	Null
B8I0K3	RUMCH Transcriptional regulator, ArsR family	0	0	0	92558	171559	345650	0.0150	NA	Increase
B8I0K4	RUMCH Periplasmic binding protein/LacI transcriptional regulator	2113955	2462825	1949055	5245902	3774247	4013570	0.0423	2.0	Increase
B8I0K5	RUMCH ABC transporter related	0	0	0	0	196786	0	NA	NA	Null
B8I0L0	RUMCH Cellulase	16298286	11171688	14072049	123251702	91612934	127883560	0.0000	8.3	Increase
B8I0L1	RUMCH Beta-xylanase	104480296	136561798	40181493	151659914	312195441	477173036	0.0939	3.3	Null
B8I0L2	RUMCH Cellulase	1984356	3968123	8651137	10163544	14806421	7480495	0.1877	2.2	Null
B8I0L3	RUMCH Cellulase	73612095	49418037	112023757	132320255	92207310	57687920	0.7602	1.2	Null
B8I0L4	RUMCH Cellulase	3792003	4654054	7961824	15829441	22214205	10028668	0.0152	2.9	Increase
B8I0L5	RUMCH Carbohydrate binding family 6	75885868	40403090	84613688	28135509	129229343	110364338	0.7087	1.3	Null
B8I0L6	RUMCH Cellulase	28499357	26618189	45212086	33103658	49328504	22798273	0.8764	1.0	Null
B8I0L8	RUMCH Alpha-galactosidase	18003427	2719124	12261601	11090680	5062077	451233	0.5637	2.0	Null
B8I0M0	RUMCH Carbohydrate binding family 6	12266421	7995740	6388039	12875991	10718610	6444501	0.8775	1.1	Null
B8I0M1	RUMCH Carbohydrate binding family 6	12138511	17051241	3748148	178579518	16550479	10214445	NA	6.2	Null
B8I0M2	RUMCH Cellulase	18219456	8803568	9027582	15517321	7445286	4145268	0.7227	1.3	Null
B8I0M3	RUMCH Cellulase	30116529	19867781	50800931	45813948	72914948	25346728	0.5815	1.4	Null
B8I0M4	RUMCH Uncharacterized protein	0	0	0	0	0	141974	NA	NA	Null
B8I0M6	RUMCH Cellulase	13006142	9112385	9847682	9959272	3781504	4549353	0.3697	1.7	Null
B8I0M7	RUMCH Pectinesterase	1214241	4213969	732818	826765	1777174	1433117	0.7930	1.5	Null
B8I0M8	RUMCH ABC transporter related	317469	420251	1075986	250454	152088	0	0.3007	4.5	Null
B8I0M9	RUMCH ABC transporter related	275087	269181	0	186080	137160	71833	0.8784	1.4	Null
B8I0N0	RUMCH Glucanase	1138478502	2620556824	1919727448	1339883514	1928235675	1973100944	0.9609	1.1	Null
B8I0N2	RUMCH Stage 0 sporulation protein A homolog	0	0	0	0	93706	0	NA	NA	Null
B8I0N3	RUMCH Extracellular solute-binding protein family 1	22371090	44649805	37046805	39331377	25044980	24700612	0.8866	1.2	Null
B8I0N6	RUMCH Alpha-L-arabinofuranosidase domain protein	487285	324661	0	1151172	808489	467188	0.5337	3.0	Null
B8I0N8	RUMCH Glycoside hydrolase family 43	0	0	0	0	282423	0	0.2962	NA	Null
B8I0N9	RUMCH Glycoside hydrolase family 8	0	126363	0	352194	1541682	399377	0.1035	18.1	Null
B8I0P1	RUMCH Putative esterase	456864	13028002	21768939	13278978	4992503	10809994	0.9790	1.2	Null
B8I0P2	RUMCH Uncharacterized protein	0	0	0	0	131055	87729	NA	NA	Null
B8I0P4	RUMCH GntR domain protein	0	0	143370	268105	333044	366314	0.2978	6.7	Null
B8I0P5	RUMCH Dihydroxy-acid and 6-phosphogluconate dehydratase	184196	2611206	4564949	828512	8495120	1202564	0.7647	1.4	Null
B8I0Q0	RUMCH ABC transporter ATP-binding protein	0	463331	490211	332683	462555	390758	0.8496	1.2	Null
B8I0Q2	RUMCH Precorrin-2 C20-methyltransferase	205447	288768	0	621259	323127	268482	0.6050	2.5	Null
B8I0Q3	RUMCH Precorrin-4 C11-methyltransferase	172566	636227	658995	653855	1359700	488972	0.4529	1.7	Null
B8I0Q8	RUMCH Porphobilinogen deaminase	2663498	1293251	2188257	4063662	3214854	2589196	0.4138	1.6	Null
B8I0Q9	RUMCH Uroporphyrinogen III methylase	1155862	1265092	1531204	1721652	1903821	1887513	0.3384	1.4	Null
B8I0R0	RUMCH Delta-aminolevulinic acid dehydratase	3556207	4410383	4218918	1362878	1246048	246440	0.0308	4.3	Decrease
B8I0R1	RUMCH Glutamate-1-semialdehyde 2,1-aminomutase	0	1154458	0	2622799	2680497	2241015	0.3500	6.5	Null
B8I0R2	RUMCH Cobalamin (Vitamin B12) biosynthesis CbiX protein	497387	4590017	1604378	9002898	10271772	6836996	0.0574	3.9	Null
B8I0R4	RUMCH Cobyrinate a,c-diamide synthase	919931	764561	643840	3981561	1332546	1298652	0.1215	2.8	Null
B8I0R5	RUMCH Cobyric acid synthase	0	202525	111050	0	133083	150323	0.9721	1.1	Null
B8I0R6	RUMCH Precorrin-8X methylmutase CbiC/CobH	0	136864	0	217958	264837	255909	0.3893	5.4	Null
B8I0R9	RUMCH S-methyl-5-thioribose kinase	3021324	4743951	3172396	5175783	2970516	4011624	0.7596	1.1	Null
B8I0S0	RUMCH Methylthioribose-1-phosphate isomerase	10651375	9636862	10699934	10627829	8025452	8666525	0.8881	1.1	Null
B8I0S1	RUMCH Class II aldolase/adducin family protein	0	34010095	3354422	20318034	8245298	9155156	0.9593	1.0	Null
B8I0S4	RUMCH Uncharacterized protein	587273	4432172	390959	1265379	202726	217667	0.3750	3.2	Null
B8I0S8	RUMCH Cellulase	2395828238	3158952812	2364194146	2856390370	2251127760	1428400958	0.7727	1.2	Null
B8I0S9	RUMCH Transposase IS3/IS911 family protein	637747	9403823	882202	3078631	0	0	NA	3.5	Null
B8I0T2	RUMCH Spore coat protein CotH	0	317583	699314	204550	408244	109600	0.9255	1.4	Null
B8I0T3	RUMCH VTC domain-containing protein	135270	1127966	1926479	1255554	722137	390025	0.8782	1.3	Null
B8I0T5	RUMCH Phosphoesterase PA-phosphatase related	0	183482	0	0	0	0	NA	NA	Null
B8I0T8	RUMCH Thymidylate synthase	2892893	3251185	3333361	1191913	2589732	642886	0.2886	2.1	Null
B8I0T9	RUMCH Dihydrofolate reductase	824973	2581332	245874	1938074	229771	453187	0.8481	1.4	Null
B8I0U0	RUMCH Translation initiation factor IF-3	67308209	34881897	80315284	422548	24129178	242695	0.2035	7.4	Null
B8I0U1	RUMCH Sulfatase	53060862	43652597	48721975	25003313	20642106	17909854	0.0288	2.3	Decrease
B8I0U2	RUMCH Uncharacterized protein	1626110	0	2122859	3777079	4279244	6033183	0.4137	3.8	Null

B8I0U3	RUMCH Tetratricopeptide TPR	24872643	17592067	20655463	27888994	27791777	81128916	0.2565	2.2	Null
B8I0U5	RUMCH Uncharacterized protein	6161913	3186506	2715724	3867681	5071368	5339866	0.8256	1.2	Null
B8I0U6	RUMCH Uncharacterized protein	0	0	0	117870	0	0	NA	NA	Null
B8I0U7	RUMCH Anthranilate synthase component 1	59989359	111898032	184581086	149365127	58365021	90880996	0.9202	1.2	Null
B8I0U8	RUMCH Glutamine amidotransferase of anthranilate synthase	9381444	2917186	2581060	15502109	15619125	16299806	0.1500	3.2	Null
B8I0U9	RUMCH Anthranilate phosphoribosyltransferase	7265068	10752272	19653958	54607049	21227608	13384125	0.1985	2.4	Null
B8I0V0	RUMCH Indole-3-glycerol phosphate synthase	5069784	7890974	11501503	12500866	7118911	6592149	0.8308	1.1	Null
B8I0V1	RUMCH N-(5'-phosphoribosyl)anthranilate isomerase	530436	1571939	714704	2377068	2015887	336561	0.5660	1.7	Null
B8I0V2	RUMCH Tryptophan synthase beta chain	81206775	163422356	33451584	43061687	66868604	118089427	0.9532	1.2	Null
B8I0V3	RUMCH Tryptophan synthase alpha chain	54021203	11801445	36949282	80264581	53900310	79707756	0.3471	2.1	Null
B8I0V4	RUMCH Aldo/keto reductase	1212845	4108239	918772	746138	1793988	3276982	0.9468	1.1	Null
B8I0V5	RUMCH Hexokinase	12641488	15836752	14903727	26204013	24856028	26744894	0.0346	1.8	Increase
B8I0V6	RUMCH Uncharacterized protein	0	0	0	1638880	0	0	NA	NA	Null
B8I0V7	RUMCH Cell wall hydrolase/autolysin	2004762	1468235	743049	616684	1446376	1828383	0.9943	1.1	Null
B8I0V8	RUMCH Alcohol dehydrogenase GroES domain protein	427418	0	15321	163626	224301	0	0.9265	1.1	Null
B8I0V9	RUMCH Ribokinase	0	0	0	133213	179101	208725	0.0170	NA	Increase
B8I0W0	RUMCH Methyl-accepting chemotaxis sensory transducer	2541124923	2819707797	1968765131	1627913152	2040962151	2021926732	0.7493	1.3	Null
B8I0W1	RUMCH RelA/SpoT domain protein	2082781	1759953	0	4325215	4072805	2258048	0.5833	2.8	Null
B8I0W3	RUMCH ABC transporter related	0	0	0	0	456560	0	0.2468	NA	Null
B8I0W4	RUMCH Uracil-DNA glycosylase superfamily	0	201516	139974	128720	178124	161428	0.8106	1.4	Null
B8I0W6	RUMCH Uncharacterized protein	30384889764	83739464073	22638867663	12833181279	16383391467	28724714210	0.3489	2.4	Null
B8I0W8	RUMCH Methyl-accepting chemotaxis sensory transducer	997275	3201963	3218008	3445164	8169945	2491159	0.3086	1.9	Null
B8I0W9	RUMCH Uncharacterized protein	1302464	1941514	938276	702013	662245	1082524	0.5295	1.7	Null
B8I0X1	RUMCH UDP-glucose 4-epimerase	0	5511180	6466698	1972012	2603907	1596527	0.8195	1.9	Null
B8I0X2	RUMCH Galactokinase	1194662	1400079	1611172	2119581	2683996	2110131	0.1557	1.6	Null
B8I0X3	RUMCH Uncharacterized protein	1868787	2930290	18312263	3176615	4773874	2177345	0.4982	2.3	Null
B8I0X9	RUMCH Extracellular solute-binding protein family 1	6332603	8045383	11731858	1492930	11790749	3552153	0.7533	1.6	Null
B8I0Y0	RUMCH Sugar-binding domain protein	290020	1091961	1069420	9128318	2486411	5011029	0.0023	6.8	Increase
B8I0Y1	RUMCH Putative sensor with HAMP domain	0	243496	0	0	0	0	NA	NA	Null
B8I0Y3	RUMCH Aldo/keto reductase	339393	633748	748793	502045	820886	277846	0.9988	1.1	Null
B8I0Y4	RUMCH Uncharacterized protein	5963948	4840354	11358371	1509089	511994	83401	0.0081	10.5	Decrease
B8I0Y5	RUMCH AMP-dependent synthetase and ligase	1274469	5965676	1968754	4595738	7943663	7338954	0.2371	2.2	Null
B8I0Y7	RUMCH Uncharacterized protein	0	0	0	11134973	0	0	NA	NA	Null
B8I0Y8	RUMCH Orn/DAP/Arg decarboxylase 2	72909446	7538347	12348852	26261388	46904788	16493143	NA	1.0	Null
B8I0Y9	RUMCH Phosphopantetheine-binding	0	6473900	15531250	12219086	978164	892708	0.8801	1.6	Null
B8I0Z0	RUMCH ATP-grasp domain-containing protein	139815603	65002181	145602525	165074013	99300992	117868693	0.8795	1.1	Null
B8I0Z1	RUMCH Uncharacterized protein	42431655	12670025	26543671	33507645	34346174	74614087	0.4870	1.7	Null
B8I0Z2	RUMCH Aminotransferase class-III	42113947	26048061	21166889	59067238	51856149	20441961	0.6244	1.5	Null
B8I0Z3	RUMCH Uncharacterized protein	0	0	2142166	722832	485863	2386618	NA	1.7	Null
B8I0Z5	RUMCH ABC transporter related	679920453	3356863595	1516200489	1623300522	1929705295	2488618683	0.8029	1.1	Null
B8I0Z6	RUMCH Transcription activator effector binding	49135906230	20900495824	27364950509	3902347100	5062122715	8526500427	0.0114	5.6	Decrease
B8I0Z7	RUMCH Transcriptional regulator, AraC family	1935408	3961445	731275	2322816	3738533	1091018	0.9190	1.1	Null
B8I0Z8	RUMCH Excinuclease ABC, A subunit	13386179	24505596	12916678	38988014	31981205	17614053	0.2355	1.7	Null
B8I101	RUMCH Uncharacterized protein	344058	396192	3340238	3010270	0	98872	NA	1.3	Null
B8I103	RUMCH Uncharacterized protein	0	0	0	0	362442	0	0.2715	NA	Null
B8I105	RUMCH Uncharacterized protein	0	118673	0	0	0	197856	0.8688	1.7	Null
B8I107	RUMCH Uncharacterized protein	0	360247	0	0	0	0	0.2917	NA	Null
B8I109	RUMCH Uncharacterized protein	0	0	0	0	178688	0	NA	NA	Null
B8I110	RUMCH Uncharacterized protein	1119968	765493	0	0	102490	595021	0.7765	2.7	Null
B8I111	RUMCH Uncharacterized protein	283734	304273	397454	133628	119341	121090	0.1977	2.6	Null
B8I112	RUMCH Uncharacterized protein	12746928	9921060	7491589	16902240	5573190	6610022	0.9692	1.0	Null
B8I114	RUMCH Lj965 prophage protein	0	0	0	0	265539	0	0.3000	NA	Null
B8I117	RUMCH Uncharacterized protein	5195791	9792613	11379109	6380200	6484813	11412138	0.9560	1.1	Null
B8I118	RUMCH Uncharacterized protein	0	0	0	0	19823	2905723	NA	NA	Null
B8I120	RUMCH Uncharacterized protein	1255984	0	0	15643857	0	0	NA	12.5	Null
B8I121	RUMCH Uncharacterized protein	482498	0	250107	169700	0	0	0.6540	4.3	Null
B8I122	RUMCH Uncharacterized protein	877654	3859633	1662185	1300299	419214	652047	0.2609	2.7	Null
B8I123	RUMCH Uncharacterized protein	1079508	62657962	34339324	0	411922	10825243	0.3901	8.7	Null
B8I124	RUMCH Putative phage major capsid protein	24580572	6939897	22546271	13131086	137311	17591732	0.7816	1.8	Null
B8I125	RUMCH Minor structural GP20 protein	7525296	16192338	12840790	3904749	13269075	6174508	0.6592	1.6	Null
B8I126	RUMCH Uncharacterized protein	0	2482050	0	348595	0	0	NA	7.1	Null
B8I128	RUMCH Uncharacterized protein	1785270	3997509	633318	3892809	474846	2053012	0.9708	1.0	Null
B8I129	RUMCH Putative phage minor capsid protein	546691	856480	237357	997976	857533	532520	0.6288	1.5	Null
B8I130	RUMCH Putative phage terminase, large subunit	0	0	0	0	0	573730	0.2063	NA	Null
B8I131	RUMCH Terminase small subunit	4897301	44885673	59081247	4174725	2612878	3345291	0.0038	10.7	Decrease
B8I133	RUMCH RNA polymerase, sigma-24 subunit, ECF subfamily	0	19595	186300	38566	70662	86190	0.9633	1.1	Null
B8I134	RUMCH Phage protein	0	0	1318293	0	0	0	NA	NA	Null
B8I136	RUMCH Uncharacterized protein	176366	0	0	0	50208	0	0.7607	3.5	Null
B8I139	RUMCH ParB-like partition protein	707831	915058	110832	1585071	1910793	600824	0.3931	2.4	Null
B8I140	RUMCH Type III restriction protein res subunit	135726	0	0	8856575	90914	138892	NA	66.9	Null
B8I141	RUMCH Phage protein	3711669	25703002	10308472	542834	773203	3317209	0.0525	8.6	Decrease
B8I142	RUMCH Phage protein	5147752	1725526	4219176	8550320	6574446	7112553	0.2886	2.0	Null
B8I143	RUMCH SMC domain protein	73935763	145596640	38065491	12270283	15967889	13173911	0.0021	6.2	Decrease
B8I145	RUMCH Transcriptional regulator, AbrB family	191429419	186530667	225388030	17365510	16394670	546853	0.0021	17.6	Decrease
B8I150	RUMCH Transcriptional regulator, XRE family	3245458	3225119	1785777	4633116	6717116	5158091	0.1901	2.0	Null
B8I152	RUMCH RNA polymerase, sigma-24 subunit, ECF subfamily	1647331	0	1515875	1283319	703461	348133	0.8764	1.4	Null
B8I153	RUMCH RNA methyltransferase, TrmH family, group 3	154557436	239252492	183696299	97831136	77714323	55641088	0.0062	2.5	Decrease
B8I154	RUMCH Mini-ribonuclease 3	557416	621467	0	2259979	0	3233179	0.5337	4.7	Null
B8I156	RUMCH NLP/P60 protein	1038333	139811	58520	1957090	657654	2671493	0.2794	4.3	Null
B8I157	RUMCH Cysteine--tRNA ligase	47229479	87469187	133446202	75271026	67341023	58606446	0.7556	1.3	Null
B8I158	RUMCH Serine acetyltransferase	0	588623	3493815	1050640	983600	1837895	0.9585	1.1	Null
B8I159	RUMCH Uncharacterized protein	0	402146	253891	0	0	0	0.2083	NA	Null
B8I160	RUMCH Uncharacterized protein	1187381	0	2438971	7093537	2546154	0	0.7591	2.7	Null
B8I162	RUMCH 8-oxoguanine DNA glycosylase domain protein	3876658	252685	0	505206	510542	0	0.5690	4.1	Null
B8I163	RUMCH Uncharacterized protein	5642404	37962169	42453204	15853731	7844968	6421875	0.2562	2.9	Null
B8I164	RUMCH Homoserine O-acetyltransferase	2744667	1571111	4428853	4045380	35337118	36381958	0.0096	8.7	Increase
B8I165	RUMCH O-acetylhomoserine/O-acetylserine sulfhydrylase	591697	12747642	18666266	7966372	4310775	4711945	0.6966	1.9	Null
B8I166	RUMCH Substrate-binding region of ABC-type glycine betaine transport sy	657935	392284	594093	516893	651727	728795	0.8104	1.2	Null
B8I167	RUMCH Glycine betaine/L-proline ABC transporter, ATPase subunit	0	199792	0	161863	177405	173962	0.6757	2.6	Null
B8I168	RUMCH 50S ribosomal protein L35	102040329	215481961	753795912	143974554	93649256	28414378	0.1455	4.0	Null
B8I169	RUMCH 50S ribosomal protein L20	885289865	678460300	650213281	24982552	208479465	23587781	0.0196	8.6	Decrease
B8I170	RUMCH DNA polymerase IV	0	0	0	108854	0	132549	0.3045	NA	Null
B8I172	RUMCH Phospholipid/glycerol acyltransferase	16576833	17943058	23554096	25929306	15926049	19775370	0.8112	1.1	Null
B8I173	RUMCH Radical SAM domain protein	252883	1654700	935208	1861288	949169	195401	0.9359	1.1	Null
B8I174	RUMCH DUF2344 domain-containing protein	584546	0	592641	65173	0	784988	0.9591	1.4	Null
B8I175	RUMCH Ribonuclease, Rne/Rng family	1362912	0	0	994872	921029	266053	NA	1.6	Null
B8I176	RUMCH 50S ribosomal protein L21	1632240301	2434142707	618368895	1173845951	165229173	918303331	0.5122	2.1	Null
B8I177	RUMCH Uncharacterized protein	0	484925	790729	0	301841	645716	0.9908	1.3	Null
B8I178	RUMCH 50S ribosomal protein L27	785572666	1217824009	1023672965	164988965	149254541	173011160	0.0000	6.2	Decrease
B8I179	RUMCH GTPase Obg	17229624	23894557	47856071	13062250	16601776	32940343	0.8307	1.4	Null
B8I180	RUMCH CRM domain-containing protein	3551881	3544791	23264717	7746000	12270099	9571794	0.9412	1.0	Null
B8I182	RUMCH Uncharacterized protein	3871828	0	245161	601525	5337679	4520014	NA	2.5	Null
B8I183	RUMCH 4Fe-4S ferredoxin iron-sulfur binding domain protein	0	425170	0	0	0	244764	0.9585	1.7	Null
B8I184	RUMCH GTP cyclohydrolase 1	0	0	0	0	257387	444750	0.1476	NA	Null
B8I185	RUMCH Metal dependent phosphohydrolase	35373469	8337740	1993995	52431	139345	71614	0.0000	173.5	Decrease
B8I186	RUMCH Dihydropteroate synthase	1125735	3599729	2706282	1716697	3673622	9533511	0.3283	2.0	Null
B8I189	RUMCH ATP:corrinoid adenosyltransferase BtuR/CobO/CobP	500959	451681	953875	0	86539	0	0.0792	22.0	Null
B8I190	RUMCH Glycogen synthase	3899481	3766767	5095338	162978	119081	0	0.0000	45.2	Decrease
B8I192	RUMCH Uncharacterized protein	379190515	1782000526	1524882189	18688022	16558258	11142442	0.0000	79.5	Decrease
B8I193	RUMCH Uncharacterized protein	1692443	28381516	19703271	21778654	3387203	32333372	0.8318	1.2	Null
B8I194	RUMCH Protein serine/threonine phosphatase	13468518	38914270	29395200	8318743	17045828	2671679	0.2171	2.9	Null
B8I195	RUMCH Propeptide PepSY amd peptidase M4	2343492859	2534274930	1822031266	3255459422	2101262557	2017029283	0.8101	1.1	Null

B8I196	RUMCH Stress responsive alpha-beta barrel domain protein	0	519604	0	69948	99982	520698	0.8698	1.3	Null
B8I197	RUMCH Polysaccharide deacetylase	3980913	14658388	6646922	4992187	5748821	22912543	0.6623	1.3	Null
B8I198	RUMCH Uncharacterized protein	8949027	39977545	8692668	4924918	2900997	0	0.2588	7.4	Null
B8I1A0	RUMCH Argininosuccinate lyase	14269044	1970827	13538007	14296656	12952192	1013646	0.9539	1.1	Null
B8I1A1	RUMCH Argininosuccinate synthase	1143144248	1483966330	819490733	722530280	826601984	445117400	0.2991	1.7	Null
B8I1A2	RUMCH Crossover junction endodeoxyribonuclease RuvC	0	313882	550576	1560310	1301091	721683	0.2855	4.1	Null
B8I1A3	RUMCH Holliday junction ATP-dependent DNA helicase RuvA	0	0	0	2018889	1422584	439537	0.0000	NA	Increase
B8I1A4	RUMCH Holliday junction ATP-dependent DNA helicase RuvB	4472761	11618375	71719492	11604216	7293753	14377544	0.4666	2.6	Null
B8I1A5	RUMCH Epoxyqueuosine reductase QueH	3749433	5824543	7877727	2337270	2340719	489812	0.0895	3.4	Null
B8I1A6	RUMCH Cell wall hydrolase/autolysin	1115952357	928857710	853570622	516940929	436905479	633824803	0.2855	1.8	Null
B8I1A7	RUMCH Uncharacterized protein	51434510	131283565	229158008	160666630	187678209	214833890	0.5363	1.4	Null
B8I1A9	RUMCH dITP/XTP pyrophosphatase	1288961	2504112	992294	0	638958	506435	0.4176	4.2	Null
B8I1B0	RUMCH Phosphoesterase	0	0	800254	657250	14728	0	NA	1.2	Null
B8I1B3	RUMCH Cell wall hydrolase, SleB	125249	0	0	0	0	0	NA	NA	Null
B8I1B4	RUMCH 5'-methylthioadenosine/S-adenosylhomocysteine nucleosidase	6594297	9365432	11544045	22369119	27289415	20298694	0.0016	2.5	Increase
B8I1B5	RUMCH 4Fe-4S ferredoxin iron-sulfur binding domain protein	0	699199	903443	146523	0	0	0.4405	10.9	Null
B8I1B6	RUMCH Transcriptional regulator, Crp/Fnr family	0	0	0	542310	86818	0	0.2083	NA	Null
B8I1B7	RUMCH Band 7 protein	75425777	121785974	143980874	57493246	38700782	47295550	0.0383	2.4	Decrease
B8I1B8	RUMCH RHH	457031	0	133142	0	119296	0	0.6442	4.9	Null
B8I1B9	RUMCH Probable nicotinate-nucleotide adenylyltransferase	471741	0	0	487974	0	0	0.9719	1.0	Null
B8I1C0	RUMCH Metal dependent phosphohydrolase	0	3972427	2605877	1783237	138762	531536	0.6799	2.7	Null
B8I1C1	RUMCH LytR	94425768	76877619	56281317	21483599	41565690	31409274	0.1557	2.4	Null
B8I1C3	RUMCH ABC transporter related	17000144	23390286	14754775	84458418	41217514	17218666	0.1485	2.6	Null
B8I1C4	RUMCH Uncharacterized protein	0	809554	0	1314550	0	1056992	NA	2.9	Null
B8I1C5	RUMCH AMP-dependent synthetase and ligase	0	419605	1409876	272759	0	0	0.5732	6.7	Null
B8I1C6	RUMCH Serine-type D-Ala-D-Ala carboxypeptidase	2358140	8279129	4116311	8027872	1139193	1431312	0.8132	1.4	Null
B8I1C7	RUMCH Competence protein ComEA helix-hairpin-helix repeat protein	1203350	1025264	1419285	0	0	0	0.0000	NA	Decrease
B8I1C8	RUMCH Threonine--tRNA ligase	0	176781	227115	4694037	2512067	1582390	0.0035	21.8	Increase
B8I1C9	RUMCH Threonine--tRNA ligase	61731905	199682555	323087279	33578061	51283907	43629488	0.0300	4.5	Decrease
B8I1D1	RUMCH Uncharacterized protein	20772630	42271531	6665869	9789035	7144428	24745374	0.7464	1.7	Null
B8I1D2	RUMCH S-layer domain protein	101844065	67282427	204058341	258764750	177669407	144805928	0.4297	1.6	Null
B8I1D5	RUMCH Uncharacterized protein	0	80514	73101	0	0	0	NA	NA	Null
B8I1D8	RUMCH Band 7 protein	8691819873	20492119505	13046625680	9517888988	9142186985	7267093603	0.3600	1.6	Null
B8I1D9	RUMCH Methyl-accepting chemotaxis sensory transducer	5279996	24071924	15342639	8386700	7045029	22565074	0.9874	1.2	Null
B8I1E0	RUMCH ABC transporter related	0	472634	0	0	858689	1571170	0.5732	5.1	Null
B8I1E1	RUMCH ABC transporter related	588648	0	0	164422	260808	0	0.9070	1.4	Null
B8I1E2	RUMCH ABC transporter related	473172	0	682291	0	258806	0	0.6791	4.5	Null
B8I1E3	RUMCH Uncharacterized protein	0	0	0	0	0	50289	NA	NA	Null
B8I1E4	RUMCH Uncharacterized protein	0	0	0	571910	633712	83036	0.0018	NA	Increase
B8I1E5	RUMCH Indolepyruvate oxidoreductase subunit IorA	10258657	14866494	13776587	9968407	7445364	6847076	0.2098	1.6	Null
B8I1E6	RUMCH Pyruvate ferredoxin/flavodoxin oxidoreductase	887543	13846729	0	0	153886	7533356	0.9255	1.9	Null
B8I1E7	RUMCH Methylglyoxal synthase	0	172414	225120	1340902	1344668	0	0.3806	6.8	Null
B8I1E8	RUMCH Coenzyme F390 synthetase	91954952	70222158	74059017	140270935	109563338	26972315	0.8754	1.2	Null
B8I1E9	RUMCH Uncharacterized protein	0	601547	0	0	0	0	0.2401	NA	Null
B8I1F0	RUMCH FMN-binding domain protein	43690	5458338	585299	4824764	316864	375159	0.9902	1.1	Null
B8I1F2	RUMCH DUF4097 domain-containing protein	286831394	104101529	126460688	129980935	36437115	89507113	0.3944	2.0	Null
B8I1F3	RUMCH Uncharacterized protein	25677299	16922062	18889580	14919127	15301828	15631813	0.6671	1.3	Null
B8I1F4	RUMCH DNA topoisomerase (ATP-hydrolyzing)	0	0	0	113156	195749	238599	0.0155	NA	Increase
B8I1F5	RUMCH DNA topoisomerase (ATP-hydrolyzing)	356612	9874456	2015066	3870532	2919540	2107615	0.8975	1.4	Null
B8I1F8	RUMCH Peptidyl-prolyl cis-trans isomerase	1495791286	2586318249	5271027505	959445132	673758825	740711889	0.0151	3.9	Decrease
B8I1F9	RUMCH FAD dependent oxidoreductase	29865309	54925675	49993972	103747354	80018004	37767496	0.2767	1.6	Null
B8I1G1	RUMCH Xanthine phosphoribosyltransferase	5093607	10508990	8383320	27980593	9302933	18043970	0.1117	2.3	Null
B8I1G2	RUMCH Basic membrane lipoprotein	151646841	196351486	192940914	317138031	217548090	230693329	0.2130	1.4	Null
B8I1G3	RUMCH ABC transporter related	2698031	2165957	1497217	7810000	6489836	3358829	0.0627	2.8	Null
B8I1G7	RUMCH Tryptophan--tRNA ligase	106902835	72356223	51841191	23548558	24763926	10356657	0.0147	3.9	Decrease
B8I1G9	RUMCH UvrABC system protein B	1070944	36579715	1232110	4646974	5360374	12033181	NA	1.8	Null
B8I1H1	RUMCH LrgB family protein	0	0	0	769737	689623	0	0.0628	NA	Null
B8I1H2	RUMCH Uncharacterized protein	0	0	0	2996337	0	251072	0.0264	NA	Increase
B8I1H3	RUMCH Phosphoglucomutase/phosphomannomutase alpha/beta/alpha do	28826320	85586601	16336406	148488771	140128261	32717557	0.2846	2.5	Null
B8I1H5	RUMCH Metal dependent phosphohydrolase	0	0	0	0	0	340477	0.2613	NA	Null
B8I1H7	RUMCH TM2 domain containing protein	451033	853994	56660	318499	659884	62041	0.8820	1.3	Null
B8I1H8	RUMCH Antifreeze protein type I	2945552	2933932	438863	3013869	2859140	1674362	0.8782	1.2	Null
B8I1H9	RUMCH Cyclic nucleotide-binding protein	0	0	0	1161676	2185086	2739455	0.0000	NA	Increase
B8I1I0	RUMCH Hydroxylamine reductase	0	0	0	0	29866	0	NA	NA	Null
B8I1I2	RUMCH SCP-like extracellular	287442	0	0	0	0	0	0.2862	NA	Null
B8I1I4	RUMCH Transcriptional regulator, AraC family	0	1169321	0	0	784390	0	NA	1.5	Null
B8I1I5	RUMCH ABC transporter related	0	0	0	107424	0	0	NA	NA	Null
B8I1I6	RUMCH ABC transporter related	0	0	0	132372	0	0	NA	NA	Null
B8I1I7	RUMCH Aldose 1-epimerase	467180604	2319850217	2817659657	960813278	448827907	461337900	0.1913	3.0	Null
B8I1I9	RUMCH TrkA-C domain protein	14829837	8317624	17889428	6663124	9810625	11553256	0.6777	1.5	Null
B8I1J0	RUMCH Uncharacterized protein	764016839	1048676070	1542857130	631860150	623106406	1178767270	0.8009	1.4	Null
B8I1J1	RUMCH Iron-containing alcohol dehydrogenase	25933434	60995356	88024613	14758587	36654283	26397155	0.3084	2.2	Null
B8I1J2	RUMCH Transcriptional regulator, MerR family	2439366	3414468	2007243	581860	0	0	0.1944	13.5	Null
B8I1J3	RUMCH Uncharacterized protein	1526556	952223	1361175	4519244	3778767	6919293	0.0084	4.0	Increase
B8I1J5	RUMCH Uncharacterized protein	0	0	187970	0	186789	0	0.9809	1.0	Null
B8I1J7	RUMCH Small acid-soluble spore protein alpha/beta type	22459840	61109757	45523794	73335501	9487382	16753057	0.8508	1.3	Null
B8I1J9	RUMCH Uncharacterized protein	0	1984843	0	0	0	0	NA	NA	Null
B8I1K1	RUMCH Uncharacterized protein	1076704	4677285	3299268	17211237	28389977	17409017	0.0002	7.0	Increase
B8I1K3	RUMCH Uncharacterized protein	430518	755698	1300426	5035154	610191	511455	0.3931	2.5	Null
B8I1K5	RUMCH ABC transporter related	48702263	4759234	2640602	3126580	3566820	5743931	NA	4.5	Null
B8I1K8	RUMCH Uncharacterized protein	2715848	2362598	3469230	1117418	1020952	0	0.3614	4.0	Null
B8I1K9	RUMCH MscS Mechanosensitive ion channel	3499503	2533368	2985052	3244487	980361	1794979	0.6078	1.5	Null
B8I1L0	RUMCH Peptidase C26	0	920363	0	3380813	1507403	970946	0.3752	6.4	Null
B8I1L5	RUMCH Putative PAS/PAC sensor protein	0	187373	0	0	0	419679	0.8195	2.2	Null
B8I1L6	RUMCH Methionine aminopeptidase	11183731	31933740	9000218	10224269	12909697	8960334	0.5955	1.6	Null
B8I1L7	RUMCH Hydrogenase accessory protein HypB	19978943	10766579	25546439	9671709	8466480	4887828	0.1251	2.4	Null
B8I1M0	RUMCH 4Fe-4S ferredoxin iron-sulfur binding domain protein	395118650	457316880	287041007	3343978	5260392	0	0.0005	132.4	Decrease
B8I1M1	RUMCH NADH-ubiquinone oxidoreductase chain 49kDa	74159762	23775096	8116378	31137697	34305362	11390742	0.7576	1.4	Null
B8I1M2	RUMCH NADH dehydrogenase (Ubiquinone) 30 kDa subunit	0	0	0	4247557	1381820	275208	0.0000	NA	Increase
B8I1M3	RUMCH NADH ubiquinone oxidoreductase 20 kDa subunit	29862689	26970786	29885064	12619829	6489412	5466028	0.0035	3.5	Decrease
B8I1M4	RUMCH Respiratory-chain NADH dehydrogenase subunit 1	0	0	0	0	153241	70677	NA	NA	Null
B8I1M5	RUMCH NADH/Ubiquinone/plastoquinone (Complex I)	0	528293	0	2152065	0	0	NA	4.1	Null
B8I1M7	RUMCH Release factor glutamine methyltransferase	941296	4412533	454399	11008640	0	29525405	0.2855	7.0	Null
B8I1M8	RUMCH Uncharacterized protein	0	894741	738116	45126	90697	0	0.2855	12.0	Null
B8I1M9	RUMCH 50S ribosomal protein L31	2867670	3036593	674539	0	0	173380	0.0532	37.9	Decrease
B8I1N0	RUMCH Transcription termination factor Rho	14383917456	25946271573	25399813659	9016885133	7416534809	9795845867	0.0353	2.5	Decrease
B8I1N1	RUMCH Glyoxalase/bleomycin resistance protein/dioxygenase	0	0	0	0	0	74072	NA	NA	Null
B8I1N3	RUMCH Uncharacterized protein	467258	2445086	655736	826503	742464	1019899	0.8649	1.4	Null
B8I1N4	RUMCH Purine nucleoside phosphorylase DeoD-type	4694737	2442860	2180531	31663922	20495471	5851297	0.0126	6.2	Increase
B8I1N5	RUMCH Sulfatase	23232687	85056299	87372498	44519668	28567283	10392888	0.2925	2.3	Null
B8I1N6	RUMCH Histidine kinase	0	0	0	1040285	375519	0	0.0711	NA	Null
B8I1N7	RUMCH Arginine deiminase	0	216232	0	0	0	0	NA	NA	Null
B8I1P1	RUMCH Beta-lactamase domain protein	0	0	0	121380	98036	156283	0.0469	NA	Increase
B8I1P2	RUMCH UDP-N-acetylglucosamine 1-carboxyvinyltransferase	1414421	6633566	4071293	15956763	17627103	11065804	0.0141	3.7	Increase
B8I1P3	RUMCH YycI domain-containing protein	244777	93288	1571139	707798	1814307	1414124	0.4912	2.1	Null
B8I1P4	RUMCH Uncharacterized protein	0	249870	359899	958589	877945	498516	0.2962	3.8	Null
B8I1P5	RUMCH Uncharacterized protein	271219138	131040278	354016849	209977126	110651161	120026758	0.4330	1.7	Null
B8I1P6	RUMCH Histidine kinase	0	0	143047	2262298	1893122	815029	0.0192	34.7	Increase
B8I1P7	RUMCH Stage 0 sporulation protein A homolog	9270081	7129309	46851636	21048168	45914901	31922486	0.5740	1.6	Null
B8I1P8	RUMCH Uncharacterized protein	2295926677	2131348274	3264555690	2603643655	2158314023	1991693892	0.8923	1.1	Null
B8I1P9	RUMCH Outer membrane protein-like protein	288701863	261823739	358254528	184137019	246503943	588050607	0.8009	1.1	Null
B8I1Q0	RUMCH S-layer domain protein	1236743559	1730791711	403886411	2090987278	1314272265	2784032305	0.3899	1.8	Null

B8I1Q1	RUMCH Uncharacterized protein	3134859303971	3203709571786	3411374105062	4792150602969	5077157602432	5380609691874	0.1696	1.6	Null
B8I1Q2	RUMCH Uncharacterized protein	6272608	8196695	3157813	6483435	19481316	17238039	0.1968	2.5	Null
B8I1Q3	RUMCH Glucose-1-phosphate adenylyltransferase, GlgD subunit	1705757	4137787	1569496	2904032	4710403	819851	0.8698	1.1	Null
B8I1Q5	RUMCH Glutamate racemase	0	0	0	31653	395090	64815	0.0656	NA	Null
B8I1Q7	RUMCH Uncharacterized protein	87674007	21615787	21624909	20491344	44734923	42399425	0.8698	1.2	Null
B8I1Q8	RUMCH PP-loop domain protein	5147381	2542251	13997787	4154997	7029006	6244440	0.9148	1.2	Null
B8I1Q9	RUMCH Ig-like, group 2	5674799520	9570966138	11629946084	4502835180	5277548271	5268730441	0.2734	1.8	Null
B8I1R0	RUMCH UTP--glucose-1-phosphate uridylyltransferase	142453602	353264285	377038951	1303083145	996066519	815383681	0.0010	3.6	Increase
B8I1R2	RUMCH Uncharacterized protein	0	348055	455693	0	0	96387	0.6002	8.3	Null
B8I1R3	RUMCH Glycoside hydrolase family 3 domain protein	10881250	8581070	15609443	5506480	3330544	10882218	0.5713	1.8	Null
B8I1R4	RUMCH GumN family protein	5699807	5626814	4128940	7702297	2060580	6794151	0.8950	1.1	Null
B8I1R5	RUMCH Glycosyltransferase 36	995932	1129958	1101540	1053159	523812	810311	0.7087	1.4	Null
B8I1R7	RUMCH ABC transporter related	10086260	0	0	0	0	0	NA	NA	Null
B8I1R8	RUMCH Transketolase	1077774430	749906941	975459824	660567315	1056010291	550008295	0.7950	1.2	Null
B8I1S0	RUMCH Transcriptional regulator, XRE family	4409611	3349609	5162121	1597494	2272241	1609025	0.0883	2.4	Null
B8I1S1	RUMCH DUF4173 domain-containing protein	0	685257	0	0	0	439326	0.9766	1.6	Null
B8I1S3	RUMCH DUF815 domain-containing protein	720963	611345	370678	582278	0	0	0.6540	2.9	Null
B8I1S4	RUMCH Peptidase M14 carboxypeptidase A	0	99532	0	0	0	0	NA	NA	Null
B8I1S6	RUMCH DNA helicase	0	721962	172918	0	313903	668486	0.9162	1.1	Null
B8I1S8	RUMCH D-isomer specific 2-hydroxyacid dehydrogenase NAD-binding	2118890	1590710	1123949	5294405	4447054	2579691	0.0943	2.5	Null
B8I1S9	RUMCH Glycoside hydrolase family 4	333989	0	546038	46499	320932	93320	0.7953	1.9	Null
B8I1T0	RUMCH Nitroreductase	2687772	0	0	0	0	0	NA	NA	Null
B8I1T1	RUMCH Transcriptional regulator, AraC family	154286	357584	354880	122319	266620	113349	0.6484	1.7	Null
B8I1T2	RUMCH Xylose isomerase	11053422646	7091723870	2936168831	7162531299	6631364846	7531059857	0.9769	1.0	Null
B8I1T3	RUMCH ROK family protein	0	341202	297513	1534049	841840	1291058	0.1658	5.7	Null
B8I1T4	RUMCH Xylulose kinase	116436855	71810581	188641054	184238407	240118099	99891461	0.5945	1.4	Null
B8I1T5	RUMCH Uncharacterized protein	1291033	549657	717690	852549	1238838	0	0.9089	1.2	Null
B8I1T6	RUMCH (R)-citramalate synthase	113510635	116938245	171295033	1147440434	468804510	558027804	0.0001	5.4	Increase
B8I1T7	RUMCH 2-isopropylmalate synthase	7574780726	3332708552	6253687597	5485312907	4101022622	3147836015	0.6757	1.3	Null
B8I1T8	RUMCH Ketol-acid reductoisomerase (NADP(+))	4462926489	9989375056	10282407701	6265169366	5878298894	3860148997	0.4606	1.5	Null
B8I1T9	RUMCH Acetolactate synthase	50922988	158850267	167829839	816215410	1005667474	527105264	0.0001	6.2	Increase
B8I1U0	RUMCH Acetolactate synthase	55154683	61798568	147557236	155005067	97040811	127208246	0.4601	1.4	Null
B8I1U1	RUMCH Glycoside hydrolase family 43	843290	2583038	795271	4085931	6571412	1164821	0.2083	2.8	Null
B8I1U3	RUMCH tRNA-specific adenosine deaminase	0	1456136	590993	194921	280046	505622	0.8104	2.1	Null
B8I1U4	RUMCH GDP-mannose 4,6-dehydratase	462703	366739	0	1067755	846643	193650	0.6351	2.5	Null
B8I1U5	RUMCH GDP-L-fucose synthase	1176188	32075	466088	706890	534905	305131	0.9366	1.1	Null
B8I1U6	RUMCH DivIVA family protein	152763292	126319536	365786399	131833526	70082623	211470916	0.7310	1.6	Null
B8I1U7	RUMCH Uncharacterized protein	851544	296898	568089	509363	58726	128106	0.3979	2.5	Null
B8I1V0	RUMCH Peptidase S8 and S53 subtilisin kexin sedolisin	0	228079	0	0	51302	109405	0.9810	1.4	Null
B8I1V2	RUMCH Transketolase domain protein	0	180738	400399	734354	592580	278627	0.4763	2.8	Null
B8I1V7	RUMCH Leucine--tRNA ligase	4146966	17818424	31259255	12654698	3915724	6995259	0.4267	2.3	Null
B8I1W2	RUMCH Peptide chain release factor 3	20406951	8927138	4606445	22854841	17287764	24832417	0.4472	1.9	Null
B8I1W3	RUMCH AAA ATPase	1260458	2561509	3068367	7660058	5499054	2020113	0.1965	2.2	Null
B8I1W5	RUMCH RNA methyltransferase, TrmH family, group 3	448058	459059	724854	2024605	1714448	1851343	0.0014	3.4	Increase
B8I1W6	RUMCH Transcriptional regulator, LacI family	5751308	5025364	8958331	7372038	9187056	6861640	0.6540	1.2	Null
B8I1W7	RUMCH Glycosyltransferase 36	130895947	219761956	91867777	180778307	198757915	57805707	0.9825	1.0	Null
B8I1W9	RUMCH Uncharacterized protein	105507654	372643957	340840914	310173348	132736672	130093829	0.7375	1.4	Null
B8I1X0	RUMCH Phenylalanine--tRNA ligase alpha subunit	58914987	547696322	560237048	208319288	138252662	96996687	0.3148	2.6	Null
B8I1X1	RUMCH Phenylalanine--tRNA ligase beta subunit	55103545	78525047	41651757	129530556	215833322	74260158	0.1124	2.4	Null
B8I1X2	RUMCH Small acid-soluble spore protein alpha/beta type	2889434	962303	3007757	0	268774	1626952	0.5582	3.6	Null
B8I1X3	RUMCH Glucose-6-phosphate isomerase	2971723	6977447	5349861	7877435	5517641	8745937	0.3599	1.4	Null
B8I1X4	RUMCH Uncharacterized protein	1363213	4233674	2364669	1996530	1246380	993897	0.3642	1.9	Null
B8I1X5	RUMCH FolC bifunctional protein	137937	887520	205063	863017	677294	1050872	0.3381	2.1	Null
B8I1X6	RUMCH Tetratricopeptide TPR	43262817	72176286	60713849	47572152	50881606	30245966	0.5482	1.4	Null
B8I1X7	RUMCH Patatin	7285467	14123112	3514360	153302633	7796264	14601466	0.0863	7.0	Null
B8I1X8	RUMCH Threonine synthase	20863438	15334352	12838988	13571344	60046834	7377533	0.6297	1.7	Null
B8I1Y0	RUMCH Homoserine dehydrogenase	57399194	105219910	109133077	105561552	148249415	103828140	0.3965	1.3	Null
B8I1Y2	RUMCH Uncharacterized protein	0	332683	229647	208857	181592	179766	0.9412	1.0	Null
B8I1Y3	RUMCH TrpR like protein, YerC/YecD	852946	0	0	724986	703461	1177095	NA	3.1	Null
B8I1Y5	RUMCH Protein translocase subunit SecA	1887186725	4402361254	15605092146	1911328806	1239325052	1427418431	0.0672	4.8	Null
B8I1Y7	RUMCH DNA polymerase III subunit alpha	117711	755396	1411401	926994	691107	23127919	NA	10.8	Null
B8I1Y8	RUMCH Transcription attenuation protein MtrB	382984907	256911499	258676232	115466316	68200494	100005076	0.0235	3.2	Decrease
B8I1Y9	RUMCH Uncharacterized protein	4258742	0	400722	1274685	1400696	433914	0.8305	1.5	Null
B8I1Z0	RUMCH Thioesterase superfamily protein	1431950	0	0	238750	334791	244592	NA	1.8	Null
B8I1Z2	RUMCH Uncharacterized protein	0	0	86598	108557	131274	0	0.8089	2.8	Null
B8I1Z3	RUMCH Heat shock protein DnaJ domain protein	8699475	16018656	14152964	2268752	9116841	3939167	0.2451	2.5	Null
B8I1Z6	RUMCH Transcriptional regulator, XRE family	2129497	7859074	1658206	14794912	21432819	11521604	0.0313	4.1	Increase
B8I1Z7	RUMCH AMP-dependent synthetase and ligase	980164	6445355	4034862	2019422	2578743	5979599	0.9320	1.1	Null
B8I1Z8	RUMCH Endonuclease MutS2	76117344	10572990	10202562	2649603	7860596	7221707	NA	5.5	Null
B8I1Z9	RUMCH 50S ribosomal subunit assembly factor BipA	37789001	62721712	71766587	154418992	289050309	235988041	0.0006	3.9	Increase
B8I200	RUMCH Endolytic murein transglycosylase	2509521	2823219	13861038	9605513	7408423	7047437	0.7464	1.3	Null
B8I201	RUMCH tRNA 5-hydroxyuridine methyltransferase	0	0	0	0	158904	0	NA	NA	Null
B8I202	RUMCH Peptidase U32	1421591	2155939	630587	998157	576380	1696973	0.8927	1.3	Null
B8I203	RUMCH Uncharacterized protein	4326569053	6620315682	6460359440	1225288899	1544183869	1032900521	0.0000	4.6	Decrease
B8I204	RUMCH Metal dependent phosphohydrolase	0	444712	0	389806	395976	0	0.8698	1.8	Null
B8I205	RUMCH CRISPR-associated protein Cas5 family	1827682	5008481	1310963	2648695	2309592	6501049	0.6140	1.4	Null
B8I212	RUMCH Transposase IS3/IS911 family protein	384055	1545314	2157701	0	0	0	0.0000	NA	Decrease
B8I213	RUMCH Penicillin-binding protein transpeptidase	339723	0	188894	587999	1087874	140669	0.5126	3.4	Null
B8I214	RUMCH Peptidase M15B and M15C DD-carboxypeptidase VanY/endolysin	10737470	21756565	29395395	11139918	17853994	42326132	0.7578	1.2	Null
B8I215	RUMCH Uncharacterized protein	0	212805	0	0	218818	0	0.9723	1.0	Null
B8I218	RUMCH Type 3a cellulose-binding domain protein	24781940691	26335369620	22527803948	32046735662	18952874200	19445768761	0.9979	1.0	Null
B8I219	RUMCH Uncharacterized protein	326749644	108068238	37087604	46252724	269401369	184460563	0.9723	1.1	Null
B8I220	RUMCH Ankyrin	167271722	382866487	373360453	88140705	130536323	37253951	0.0342	3.6	Decrease
B8I222	RUMCH SMI1	3845440	35401775	259033186	11957683	4757255	8681128	0.0501	11.7	Decrease
B8I223	RUMCH SMI1	0	659257	484458	1523000	0	0	0.9448	1.3	Null
B8I224	RUMCH Uncharacterized protein	4846133	6552611	12837970	16719890	13010911	13784514	0.1944	1.8	Null
B8I225	RUMCH AAA ATPase central domain protein	13921065	2821105	6324152	5447615	6088384	5869861	0.7896	1.3	Null
B8I226	RUMCH Uncharacterized protein	3804270	3399054	5049901	6868115	5618641	4602311	0.3381	1.4	Null
B8I227	RUMCH Tail sheath protein	112313740	130951862	233388101	946858432	505365204	355798988	0.0039	3.8	Increase
B8I228	RUMCH Uncharacterized protein	5993091599	12228289039	12665684823	4836535441	3559879004	1108225281	0.0643	3.2	Null
B8I229	RUMCH Uncharacterized protein	48971008	159683539	42994096	98534683	118197805	120802878	0.6002	1.3	Null
B8I232	RUMCH Uncharacterized protein	3442051	33506032	21433373	1810430	563687	4162931	0.0451	8.9	Decrease
B8I233	RUMCH LysM domain-containing protein	36991817	14822902	24116904	7252235	6517519	8459189	0.0464	3.4	Decrease
B8I234	RUMCH Phage protein D	16167554	22010377	81657881	23506915	116266764	149684479	0.2925	2.4	Null
B8I235	RUMCH Rhs element Vgr protein	24797488	148346468	138394127	47626270	50024433	124127546	0.8950	1.4	Null
B8I238	RUMCH Baseplate	27841361	15532793	16761224	35094766	24900486	20350843	0.6539	1.3	Null
B8I239	RUMCH Uncharacterized protein	31197855	45923851	21818761	32411979	44175433	31161717	0.8232	1.1	Null
B8I240	RUMCH Phage tail protein	2279081	4005180	4236225	5500980	8927704	12854196	0.0604	2.6	Null
B8I241	RUMCH Peptidase S74 domain-containing protein	58323546	95923790	104330589	129539548	105931194	14493007	0.9962	1.0	Null
B8I242	RUMCH Uncharacterized protein	0	0	0	452017	1238838	315486	0.0001	NA	Increase
B8I243	RUMCH Peptidase S74 domain-containing protein	4347962	16834989	23714849	22004270	3002072	2473891	0.7261	1.6	Null
B8I244	RUMCH Uncharacterized protein	0	873908	788844	0	478463	0	0.7765	3.5	Null
B8I245	RUMCH Peptidase S74 domain-containing protein	14839448869	26351240472	30052189061	15804551414	15603616220	13102524973	0.3090	1.6	Null
B8I248	RUMCH Single-strand binding protein/Primosomal replication protein n	3593002	12233747	8864679	32348407	10922273	11704087	0.2083	2.2	Null
B8I252	RUMCH Polyprenyl synthetase	0	191587	0	0	771939	0	NA	4.0	Null
B8I253	RUMCH Primosomal protein N'	0	0	0	366491	216566	348858	0.0012	NA	Increase
B8I254	RUMCH Peptide deformylase	6526516	15703932	5534588	10898687	8519804	709022	0.8195	1.4	Null
B8I255	RUMCH Methionyl-tRNA formyltransferase	5416045	7745311	2283151	10273578	13093481	20096814	0.1134	2.8	Null
B8I256	RUMCH Uncharacterized protein	0	0	0	0	39580	0	NA	NA	Null
B8I258	RUMCH 16S rRNA m5C967 methyltransferase	0	0	0	1845700	605105	0	0.0311	NA	Increase
B8I260	RUMCH Protein serine/threonine phosphatase	0	0	0	1282962	1184589	0	0.0277	NA	Increase
B8I261	RUMCH Non-specific serine/threonine protein kinase	29537159	124866052	113467800	410863144	198290370	115010839	0.1373	2.7	Null

B8I262	RUMCH Small ribosomal subunit biogenesis GTPase RsgA	0	4072325	1364067	939149	829545	572414	0.7390	2.3	Null
B8I263	RUMCH Ribulose-phosphate 3-epimerase	0	3178943	148644	63143	128612	2551212	NA	1.2	Null
B8I264	RUMCH Thiamine diphosphokinase	317389	635909	0	540010	60129	417277	0.9605	1.1	Null
B8I265	RUMCH GCN5-related N-acetyltransferase	3255813	6928165	7945177	11725826	10358927	6721797	0.2565	1.6	Null
B8I267	RUMCH DNA	0	0	257509	0	0	0	NA	NA	Null
B8I269	RUMCH Cellulosome anchoring protein cohesin region	44825338	79293413	47825809	111704746	119057727	87577270	0.0684	1.9	Null
B8I270	RUMCH Transposase IS116/IS110/IS902 family protein	315944	0	0	163002	0	0	0.8487	1.9	Null
B8I273	RUMCH S-layer domain protein	0	0	0	280332	199381	2823906	NA	NA	Null
B8I274	RUMCH Transcriptional regulator, AraC family	0	0	0	101308	163797	0	NA	NA	Null
B8I275	RUMCH Putative outer membrane protein	88793293	234136939	575547760	170611596	310312095	183168070	0.8647	1.4	Null
B8I276	RUMCH Cellulase	12716927	16670647	7739009	35902878	42878402	74423287	0.0093	4.1	Increase
B8I277	RUMCH Cellulase	66394973	91181581	94149496	104335565	88476141	77139064	0.6746	1.1	Null
B8I278	RUMCH DUF1232 domain-containing protein	0	0	0	0	0	193888	NA	NA	Null
B8I283	RUMCH SNF2-related protein	0	2636488	4210692	0	2207619	887478	0.8481	2.2	Null
B8I284	RUMCH Putative aminopeptidase	20462826	42057173	62643503	17735539	66026001	66619513	0.7349	1.2	Null
B8I285	RUMCH Oligoendopeptidase F	27816223	59250385	42310613	123902661	184021653	63736737	0.0274	2.9	Increase
B8I286	RUMCH Uncharacterized protein	137762312	148355199	169126974	210631217	102867729	48117115	0.7893	1.3	Null
B8I288	RUMCH Carbohydrate-binding family 9	1311088339	1138460636	299192853	408620037	379439374	334150131	0.2494	2.4	Null
B8I289	RUMCH Transcriptional regulator, LacI family	0	531525	685368	486276	60115	339844	0.9470	1.4	Null
B8I290	RUMCH Uncharacterized protein	484293843	923939428	678412593	241167795	229416112	160961227	0.0004	3.3	Decrease
B8I291	RUMCH Uncharacterized protein	4866865	242086	0	0	0	1671266	NA	3.1	Null
B8I292	RUMCH L-ribulose-5-phosphate 4-epimerase	885101	934144	1973324	2463529	2195410	639386	0.6546	1.4	Null
B8I293	RUMCH Carbohydrate kinase FGGY	0	231637	430946	3096057	1718187	721892	0.1049	8.4	Null
B8I294	RUMCH L-fucose isomerase-like protein	97602824	94837994	84656091	80336850	36289590	44646611	0.3032	1.7	Null
B8I295	RUMCH Regulatory protein GntR HTH	1003185	121307	810244	308868	631024	440601	0.8166	1.4	Null
B8I297	RUMCH Uncharacterized protein	1517324	0	0	0	0	0	NA	NA	Null
B8I298	RUMCH Sugar fermentation stimulation protein homolog	0	0	0	0	52198	31585	NA	NA	Null
B8I299	RUMCH Desulfoferrodoxin	175832474	579852986	577016653	215248316	199790250	297743341	0.4573	1.9	Null
B8I2A1	RUMCH Diacylglycerol kinase catalytic region	316382	560803	0	479307	6529182	2463141	0.1251	10.8	Null
B8I2A2	RUMCH Small, acid-soluble spore protein	0	0	1656677	0	617340	0	NA	2.7	Null
B8I2A3	RUMCH Quinolinate phosphoribosyltransferase [decarboxylating]	25531569	34764727	29557970	55558911	62652417	30402340	0.1856	1.7	Null
B8I2A4	RUMCH L-aspartate oxidase	2022159	0	0	448497	1012467	1240496	0.9515	1.3	Null
B8I2A5	RUMCH Quinolinate synthase A	43521974	170106832	75975396	22206582	25918968	26287457	0.0311	3.9	Decrease
B8I2A6	RUMCH DNA gyrase subunit A	390862133	159021407	378821053	457209315	513326750	323283485	0.6104	1.4	Null
B8I2A7	RUMCH ParB-like partition protein	0	5912871	28144779	0	3619613	231482	0.4557	8.8	Null
B8I2A8	RUMCH Ribosomal RNA small subunit methyltransferase G	729363	2790927	773939	5736081	3772099	5779445	0.0407	3.6	Increase
B8I2A9	RUMCH tRNA uridine 5-carboxymethylaminomethyl modification enzyme	10751439	99263052	5697908	8970020	12169350	18107360	0.4394	2.9	Null
B8I2B0	RUMCH tRNA modification GTPase MnmE	2774246	6675554	10749901	5720985	3690260	3589423	0.6226	1.6	Null
B8I2B1	RUMCH Single-stranded nucleic acid binding R3H domain protein	36817567	128081334	184377482	73722995	135926407	56785827	0.8697	1.3	Null
B8I2B2	RUMCH 60 kDa inner membrane insertion protein	3310673	2669582	4062364	4973062	5446575	3271268	0.4573	1.4	Null
B8I2B7	RUMCH ATP-grasp domain-containing protein	0	197914	0	1136140	934270	1207223	0.0981	16.6	Null
B8I2B9	RUMCH Transposase IS3/IS911 family protein	0	0	0	277900	0	1513002	0.0446	NA	Increase
B8I2C1	RUMCH Mannosyl-glycoprotein endo-beta-N-acetylglucosamidase	160869966	68153998	174575674	15662550	57820157	10001333	0.0608	4.8	Null
B8I2C2	RUMCH Uncharacterized protein	4162367	8343902	5099413	2315763	2388449	7599171	0.8481	1.4	Null
B8I2C4	RUMCH Transcriptional regulator, XRE family	6028117	2836644	0	2027907	1363344	2335973	0.8481	1.5	Null
B8I2C6	RUMCH Uncharacterized protein	50822464	149174369	157518892	91640520	105081047	75129764	0.8132	1.3	Null
B8I2C9	RUMCH Uncharacterized protein	2284412	6426478	4310867	1448939	1861274	10816866	0.8385	1.1	Null
B8I2D1	RUMCH Zot domain-containing protein	0	882112	0	960152	765714	0	0.8496	2.0	Null
B8I2F6	RUMCH Alpha-galactosidase	209167921	243343038	207751890	158749135	293431065	213170196	0.8983	1.0	Null
B8I2G0	RUMCH Molybdenum cofactor synthesis domain protein	0	1815620	0	0	535356	264370	0.8796	2.3	Null
B8I2G1	RUMCH MOSC domain containing protein	164274	275435	0	107092	119192	65006	0.8777	1.5	Null
B8I2G2	RUMCH Cyclic pyranopterin monophosphate synthase	0	0	0	141838	106349	204007	0.0289	NA	Increase
B8I2G3	RUMCH GTP 3',8-cyclase	874869	0	0	0	0	0	NA	NA	Null
B8I2G4	RUMCH Molybdopterin-guanine dinucleotide biosynthesis protein B	0	602935	0	0	0	265524	0.9233	2.3	Null
B8I2G5	RUMCH Molybdopterin molybdenumtransferase	0	261983	193784	285930	471114	463523	0.4416	2.7	Null
B8I2G6	RUMCH Electron transfer flavoprotein alpha subunit	0	104276	0	463906	271681	171310	0.2469	8.7	Null
B8I2G7	RUMCH Electron transfer flavoprotein alpha/beta-subunit	0	518703	241794	592801	902640	763747	0.4103	3.0	Null
B8I2G8	RUMCH Transcriptional regulator, MarR family	10479751	11079711	1174360	0	3038587	3707377	0.5833	3.4	Null
B8I2H0	RUMCH 4-hydroxy-tetrahydrodipicolinate synthase	656890	9848724	255857	15604218	19695232	15411664	0.1772	4.7	Null
B8I2H1	RUMCH Aminotransferase class-III	8174823	4421380	1802638	6945440	5790010	5089763	0.8463	1.2	Null
B8I2H5	RUMCH Nitrogenase iron protein	469662	1057983	337995	508295	390108	0	0.6927	2.1	Null
B8I2H7	RUMCH Phospho-2-dehydro-3-deoxyheptonate aldolase	0	0	0	4395179	4538256	0	0.0034	NA	Increase
B8I2I4	RUMCH Acyl-CoA dehydrogenase domain protein	0	519673	749542	869103	270362	2288711	0.4959	2.7	Null
B8I2I7	RUMCH 3-oxoacyl-(Acyl-carrier-protein)-like protein	194400	0	300563	91547	0	91444	0.7497	2.7	Null
B8I2I8	RUMCH Uncharacterized protein	688758	1665880	579073	336200	336553	318119	0.1452	3.0	Null
B8I2J5	RUMCH Uncharacterized protein	16501646	115092823	12462693	11250483	12598994	14092800	0.2196	3.8	Null
B8I2J6	RUMCH Dihydroxyacetone kinase, L subunit	0	512651	0	0	308463	0	0.9463	1.7	Null
B8I2J7	RUMCH Dak kinase	0	81955	1699114	40945	0	66960	0.2855	16.5	Null
B8I2J9	RUMCH Short-chain dehydrogenase/reductase SDR	0	0	0	28528	66313	144749	0.1569	NA	Null
B8I2K0	RUMCH Periplasmic binding protein/LacI transcriptional regulator	4355205	18692388	5553506	7051447	3316692	1234458	0.3255	2.5	Null
B8I2K2	RUMCH ABC transporter related	0	0	0	0	25729	0	NA	NA	Null
B8I2K6	RUMCH Cellulase	2852470616	7070179681	10854485578	7547295410	6731137890	5871865700	0.9247	1.0	Null
B8I2K9	RUMCH 40-residue YVTN family beta-propeller repeat protein	0	0	0	84682	77953	0	NA	NA	Null
B8I2L2	RUMCH Integrase family protein	1725921	3597651	4803017	1199147	2822556	1379332	0.4394	1.9	Null
B8I2L3	RUMCH Cellulase	659223047	393547022	734335138	2254006008	2302855476	1435588405	0.0050	3.4	Increase
B8I2L4	RUMCH Probable pectate lyase C	263640755	86034479	63922883	112050237	190666138	211345556	0.8463	1.2	Null
B8I2L5	RUMCH Uncharacterized protein	3149289	700913	0	2108384	0	0	0.8496	1.8	Null
B8I2L6	RUMCH Stage 0 sporulation protein A homolog	0	0	0	123444	162059	0	0.3001	NA	Null
B8I2L7	RUMCH Histidine kinase	0	0	0	224312	172174	269159	0.0063	NA	Increase
B8I2L9	RUMCH Alkyl hydroperoxide reductase/ Thiol specific antioxidant/ Mal alle	4958287	36290666	16132657	15577520	8161390	14474572	0.7973	1.5	Null
B8I2M1	RUMCH ROK family protein	0	0	0	152268	0	0	NA	NA	Null
B8I2M7	RUMCH BFD domain protein (2Fe-2S)-binding domain protein	3181104	5173814	13445738	4862338	4054884	3496760	0.5226	1.8	Null
B8I2M8	RUMCH Beta-galactosidase	0	0	0	252806	719131	0	0.1151	NA	Null
B8I2M9	RUMCH PfkB domain protein	0	138167	176279	198725	92084	654614	0.4503	3.0	Null
B8I2N2	RUMCH Mannonate dehydratase	258728	0	0	0	0	0	0.2965	NA	Null
B8I2N8	RUMCH Serine hydroxymethyltransferase	104425631	51681788	105169381	197455284	67687519	21753221	0.9652	1.1	Null
B8I2N9	RUMCH AIR synthase related protein domain protein	6543048	19397782	43709877	29553325	31962960	23147289	0.7349	1.2	Null
B8I2P0	RUMCH Uncharacterized protein	0	5339164	0	9553792	1681705	1478849	NA	2.4	Null
B8I2P1	RUMCH Probable tRNA sulfurtransferase	0	92086	0	9068369	419420	18104140	0.0002	299.6	Increase
B8I2P2	RUMCH Aminotransferase class V	12993107	19829777	15978940	3912484	4401097	3923430	0.0000	4.0	Decrease
B8I2P3	RUMCH Uncharacterized protein	260747	217412	499853	0	0	0	0.0021	NA	Decrease
B8I2P4	RUMCH NADH ubiquinone oxidoreductase 20 kDa subunit	8474567	11156626	9051704	2607619	6017089	9113162	0.6628	1.6	Null
B8I2P5	RUMCH NADH dehydrogenase (Ubiquinone) 30 kDa subunit	1608518	242359	0	0	0	0	0.0509	NA	Decrease
B8I2P6	RUMCH NADH/Ubiquinone/plastoquinone (Complex I)	0	0	0	131184	0	0	NA	NA	Null
B8I2Q0	RUMCH Peptidoglycan-binding LysM	2704875	23504580	11204422	1372298	2334494	2108478	0.0254	6.4	Decrease
B8I2Q1	RUMCH LexA repressor	7771275	13617695	6261877	2607310	2178861	1053351	0.0022	4.7	Decrease
B8I2Q2	RUMCH RNA-binding protein Hfq	48666889	112958115	264807394	264177725	174632986	72368962	0.8042	1.2	Null
B8I2Q3	RUMCH tRNA dimethylallyltransferase	0	0	0	2376357	781730	2076421	0.0000	NA	Increase
B8I2Q4	RUMCH DNA mismatch repair protein MutL	3320785	4110987	2952585	14907801	4123161	5463989	0.1767	2.4	Null
B8I2Q5	RUMCH DNA mismatch repair protein MutS	0	671119	0	389285	244758	0	0.9972	1.1	Null
B8I2Q6	RUMCH Uncharacterized protein	5336025	280523186	335825758	10237652	6098614	5788177	0.0016	28.1	Decrease
B8I2Q7	RUMCH tRNA-2-methylthio-N(6)-dimethylallyladenosine synthase	4542367	5970123	27377749	4874986	8374204	9184270	0.7211	1.7	Null
B8I2Q9	RUMCH Thioesterase superfamily protein	68586876	26743578	97283635	102516079	42264866	126565703	0.6539	1.4	Null
B8I2R1	RUMCH Peptidase S14 ClpP	0	358522	0	63042	46915	31302	0.8104	2.5	Null
B8I2R4	RUMCH Peptidase M16 domain protein	657569	870267	4441406	69336085	53957820	6929624	0.0005	21.8	Increase
B8I2R5	RUMCH Polyribonucleotide nucleotidyltransferase	801012811	213193235	365703456	1867685575	1332877240	1122202395	0.1288	3.1	Null
B8I2R6	RUMCH 30S ribosomal protein S15	1321686	78307298	9620061	1025319	1352939	3159792	0.0331	16.1	Decrease
B8I2R7	RUMCH ATPase associated with various cellular activities AAA	85344519	62299166	89465800	206801804	242867319	88674455	0.1230	2.3	Null
B8I2R9	RUMCH Transglutaminase domain protein	2279240	4324282	0	9386797	7619687	4753035	0.4954	3.3	Null
B8I2S3	RUMCH Copper ion binding protein	5831119	3808109	3521280	2519128	1624808	3899620	0.5802	1.6	Null
B8I2S5	RUMCH Cell envelope-related transcriptional attenuator	26812543	21272506	25283009	55762172	113101508	27821162	0.1201	2.7	Null
B8I2S6	RUMCH Copper amine oxidase domain protein	154056229	121784496	77055483	122513930	113206403	116079102	0.9689	1.0	Null

B8I2S7	RUMCH Peptidase S1 and S6 chymotrypsin/Hap	388205763	353707500	470460592	433227233	334689054	340142507	0.9539	1.1	Null
B8I2S9	RUMCH Nitrilase/cyanide hydratase and apolipoprotein N-acyltransferase	610278	74802	0	6177742	2508974	2913325	0.0722	16.9	Null
B8I2T0	RUMCH UPF0210 protein Ccel	5157629	7828257	6399814	46019809	121548853	29037792	0.0000	10.1	Increase
B8I2T1	RUMCH UPF0237 protein Ccel	0	0	0	128459	225307	192879	0.0143	NA	Increase
B8I2T2	RUMCH Radical SAM domain protein	0	0	64997	419596	5942853	2114404	0.0005	130.4	Increase
B8I2T4	RUMCH UDP-N-acetylmuramoylalanine--D-glutamate ligase	18977126	6923968	7439119	6930708	4583186	4676846	0.3035	2.1	Null
B8I2T5	RUMCH MCP methyltransferase, CheR-type	1341181	1332189	756830	251487	183146	0	0.1035	7.9	Null
B8I2T6	RUMCH 4-hydroxy-3-methylbut-2-enyl diphosphate reductase	2365754893	2467648389	2330341797	7760130515	5730940655	5623012048	0.0004	2.7	Increase
B8I2T7	RUMCH Cytidylate kinase	75601159	115315109	50620363	26999810	24178211	81286661	0.6077	1.8	Null
B8I2T8	RUMCH Chorismate mutase AroH	0	934196	1354195	1125696	1288641	608230	0.8463	1.3	Null
B8I2T9	RUMCH Uncharacterized protein	1911729	0	7704003	990144	0	0	0.4772	9.7	Null
B8I2U0	RUMCH Uncharacterized protein	0	0	0	9819	0	0	NA	NA	Null
B8I2U1	RUMCH HI0933 family protein	405058	0	0	0	0	538737	0.9560	1.3	Null
B8I2U2	RUMCH Transcriptional regulator, RpiR family	17272367	12268503	16730745	26325427	30314866	17059926	0.2925	1.6	Null
B8I2U4	RUMCH Conserved carboxylase region	2311972	162822	2126378	745383	582185	938419	0.5802	2.0	Null
B8I2U5	RUMCH Biotin/lipoyl attachment domain-containing protein	73875765	18220361	124207782	232683994	127104886	138274608	0.2925	2.3	Null
B8I2U6	RUMCH Carboxyl transferase	110644496	110030464	116954942	39127573	65143840	25369091	0.0583	2.6	Null
B8I2U8	RUMCH RsgI N-terminal anti-sigma domain-containing protein	750284	0	0	0	748950	0	NA	1.0	Null
B8I2U9	RUMCH Putative rRNA methylase	5208714	9001123	4064003	17547203	17629705	22260645	0.0099	3.1	Increase
B8I2V1	RUMCH Pseudouridine synthase	1346722	1750142	2334176	2921842	1539223	1050570	0.9435	1.0	Null
B8I2V2	RUMCH Sporulation protein YtfJ	14582412	62525666	35019344	12145371	32539536	45684230	0.9698	1.2	Null
B8I2V4	RUMCH Segregation and condensation protein B	1315456	3612720	3618135	335235	344361	700204	0.0081	6.2	Decrease
B8I2V5	RUMCH Segregation and condensation protein A	0	252495	568430	888492	455107	308573	0.6481	2.0	Null
B8I2V7	RUMCH Serine-type D-Ala-D-Ala carboxypeptidase	32722507	52707622	143922872	119004019	208309049	69726627	0.4152	1.7	Null
B8I2V8	RUMCH Pyrimidine-nucleoside phosphorylase	652797	1506177	1189011	16760301	18803118	15975333	0.0000	15.4	Increase
B8I2V9	RUMCH Tyrosine recombinase XerC	548487488	683485819	570997724	586984631	334460747	257711716	0.3935	1.5	Null
B8I2W1	RUMCH NUDIX hydrolase	209607139	188000772	318886780	57368388	65458662	84980721	0.0119	3.4	Decrease
B8I2W2	RUMCH Ribonuclease H	0	475253	0	0	0	405274	0.9764	1.2	Null
B8I2W3	RUMCH Pyrroline-5-carboxylate reductase	3019882	19040729	5578865	11530806	3247054	3451903	0.7658	1.5	Null
B8I2W4	RUMCH Phenylacetate-coenzyme A ligase	11126832	233704	7282889	41559741	42973029	53786960	0.0843	7.4	Null
B8I2W5	RUMCH L-lactate permease	0	0	0	2226560	0	0	NA	NA	Null
B8I2W6	RUMCH Uncharacterized protein	37753181	24938763	46594863	48396399	43894738	49415064	0.5609	1.3	Null
B8I2W7	RUMCH RNA-metabolising metallo-beta-lactamase	7309426	6703219	4083828	27189960	9282254	17273093	0.0682	3.0	Null
B8I2W8	RUMCH Peptidase M42 family protein	23481516	64858414	201586717	138788512	149099648	125707998	0.6045	1.4	Null
B8I2W9	RUMCH Peptidase M42 family protein	7358480	37815535	13564581	8407174	17178787	11867096	0.7364	1.6	Null
B8I2X0	RUMCH Peptidase M42 family protein	0	0	0	943598	1191831	324965	0.0000	NA	Increase
B8I2X2	RUMCH Oligopeptide/dipeptide ABC transporter, ATPase subunit	8726893	38355389	19151748	22797461	13801280	33062474	0.8438	1.1	Null
B8I2X3	RUMCH Oligopeptide/dipeptide ABC transporter, ATPase subunit	4995548	6837846	7410561	6950521	12617441	13540253	0.2371	1.7	Null
B8I2X4	RUMCH Binding-protein-dependent transport systems inner membrane co	0	0	198431	0	0	0	NA	NA	Null
B8I2X5	RUMCH Binding-protein-dependent transport systems inner membrane co	0	303508	0	364466	458743	56412	0.6587	2.9	Null
B8I2X6	RUMCH Extracellular solute-binding protein family 5	4196404988	5745920622	2902787888	2203291585	2687694996	2016789793	0.2524	1.9	Null
B8I2X7	RUMCH ABC transporter related	41720436	75538458	74078799	35839548	58085559	12162680	0.4535	1.8	Null
B8I2X8	RUMCH ABC transporter related	78676370	119163901	57928827	37003319	45979919	31819369	0.1193	2.2	Null
B8I2X9	RUMCH tRNA(Met) cytidine acetate ligase	860416	251610	469138	652970	1413053	603512	0.5956	1.7	Null
B8I2Y0	RUMCH Ribonuclease J	15190401	3834116	4977876	4623586	7957676	3576784	0.6676	1.5	Null
B8I2Y1	RUMCH Glycosyl transferase group 1	0	176575	326749	0	220784	254199	0.9688	1.1	Null
B8I2Y2	RUMCH ATP-dependent helicase/nuclease subunit A	0	156522	0	240910	171946	44038	0.6791	2.9	Null
B8I2Y3	RUMCH ATP-dependent helicase/deoxyribonuclease subunit B	220731	284828	0	0	0	40461	0.4650	12.5	Null
B8I2Y6	RUMCH Amine oxidase	0	0	128129	0	0	0	NA	NA	Null
B8I2Y7	RUMCH Thioredoxin reductase	590399921	260430040	72633586	174645975	244517204	376678618	0.9247	1.2	Null
B8I2Y8	RUMCH Putative signal transduction protein with CBS domains	16563976	28196324	10681135	17289869	14190109	9600400	0.7118	1.3	Null
B8I2Y9	RUMCH Transcriptional regulator, MerR family	14187893	13119212	10208235	1300722	1081170	0	0.0431	15.8	Decrease
B8I2Z0	RUMCH Rubrerythrin family protein	109522	1634587	375015	594183	1152086	3145125	0.3846	2.3	Null
B8I2Z2	RUMCH Aspartate 1-decarboxylase	0	893294	0	704502	862554	673359	NA	2.5	Null
B8I2Z3	RUMCH Pantothenate synthetase	11071247	19803789	0	6751307	4149713	8843780	0.8796	1.6	Null
B8I2Z4	RUMCH 3-methyl-2-oxobutanoate hydroxymethyltransferase	28703814	52561077	103374242	12523027	23979195	40052520	0.3622	2.4	Null
B8I2Z5	RUMCH Uncharacterized protein	0	643803	1498110	1455688	1398181	1807091	0.5890	2.2	Null
B8I2Z6	RUMCH Glycine--tRNA ligase	41158236	8190620	7484965	18164676	2626681	5221373	0.4501	2.2	Null
B8I2Z7	RUMCH DNA-directed RNA polymerase subunit omega	19148135	9751697	14566158	6505858	7968405	11268382	0.5041	1.7	Null
B8I2Z8	RUMCH Guanylate kinase	7484155	13237598	6172101	9187662	7408127	0	0.8369	1.6	Null
B8I2Z9	RUMCH Putative regulatory protein Ccel	420138203	386613404	427460402	527542413	539271917	345637743	0.6935	1.1	Null
B8I300	RUMCH YicC domain protein	9484718	19465355	11448710	57709521	34287444	34676654	0.0025	3.1	Increase
B8I301	RUMCH Stage 0 sporulation protein A homolog	2128879	13053224	20199602	26678035	28043781	28554977	0.2316	2.4	Null
B8I302	RUMCH Ribosomal RNA small subunit methyltransferase E	0	0	0	900948	1070553	880413	0.0000	NA	Increase
B8I303	RUMCH Ribosomal protein L11 methyltransferase	715031	406521	565506	1905877	3696567	1351692	0.0213	4.1	Increase
B8I304	RUMCH Chaperone protein DnaJ	601209981	719734991	1399554116	269863846	294858804	410227532	0.1159	3.1	Null
B8I305	RUMCH Chaperone protein DnaK	16637207415	14663454347	11079500660	13898570428	10335881832	11783002530	0.8481	1.2	Null
B8I306	RUMCH Protein GrpE	95331985	307059985	471342743	48305095	49516152	25513245	0.0013	7.1	Decrease
B8I307	RUMCH Heat-inducible transcription repressor HrcA	30614393	761435	478681	10818265	34077382	44855153	0.5732	2.8	Null
B8I308	RUMCH Uncharacterized protein	0	0	0	0	92528	0	NA	NA	Null
B8I309	RUMCH Aminotransferase class IV	0	5104166	1509112	5650947	7825256	6896320	0.4624	3.1	Null
B8I310	RUMCH Metal dependent phosphohydrolase	0	1345822	0	0	0	0	NA	NA	Null
B8I311	RUMCH Deoxyguanosinetriphosphate triphosphohydrolase	2481771	3188055	3815480	2441766	2272241	1626952	0.3622	1.5	Null
B8I314	RUMCH Sulfatase	115472	1114164	0	182287	366788	619486	0.9585	1.1	Null
B8I315	RUMCH Uncharacterized protein	6785717	6118423	0	7863315	5721066	4773236	0.8799	1.4	Null
B8I316	RUMCH Cellulase	425608420	171136447	167514270	957253440	899826916	1165763403	0.0331	4.0	Increase
B8I318	RUMCH Glycosyl transferase family 2	0	535298	794372	316001	697448	847802	0.7955	1.4	Null
B8I319	RUMCH Endonuclease/exonuclease/phosphatase	1074380	1043597	1418332	1287117	1163845	1965467	0.6279	1.2	Null
B8I320	RUMCH Glycosyl transferase family 2	0	229908	0	0	0	1639613	NA	7.1	Null
B8I321	RUMCH Aminotransferase class-III	132862	2060236	787257	4011643	3972739	1850986	0.1805	3.3	Null
B8I322	RUMCH NAD-dependent epimerase/dehydratase	0	0	0	0	0	209846	NA	NA	Null
B8I325	RUMCH UbiA prenyltransferase	0	0	0	106943	78367	0	NA	NA	Null
B8I328	RUMCH Uncharacterized protein	0	0	0	0	48323	0	NA	NA	Null
B8I337	RUMCH RelA/SpoT domain protein	0	0	0	0	0	159034	NA	NA	Null
B8I340	RUMCH IS66 Orf2 family protein	0	0	0	139989	0	0	NA	NA	Null
B8I341	RUMCH Transposase IS66	3991296	3225402	3163363	1872465	698519	421305	0.0607	3.5	Null
B8I356	RUMCH Cof-like hydrolase	1863285	466314	1798458	1555394	4853876	1518073	0.4917	1.9	Null
B8I357	RUMCH Transcriptional regulator, TraR/DksA family	361340	160349	0	295426	255788	0	0.9943	1.1	Null
B8I359	RUMCH 3-dehydroquinate synthase	4002693	3308521	13680328	13061127	6931254	10175383	0.5865	1.4	Null
B8I360	RUMCH Isoleucine--tRNA ligase	43174459	31300688	29246248	42727512	243375363	52112120	0.1642	3.3	Null
B8I361	RUMCH Uncharacterized protein	0	0	0	0	0	263148	0.2877	NA	Null
B8I362	RUMCH Transcriptional regulator, GntR family with aminotransferase dom	1904117	1791519	376767	442379	1817126	901065	0.8698	1.3	Null
B8I363	RUMCH Pyridoxal 5'-phosphate synthase subunit PdxS	13465939302	6313790195	6511795891	2692392358	1467428572	1619340479	0.0079	4.5	Decrease
B8I364	RUMCH Pyridoxal 5'-phosphate synthase subunit PdxT	8331646	12109986	11501881	20662486	24269964	11920778	0.1124	1.8	Null
B8I365	RUMCH Rhomboid family protein	6015233	5056283	6637646	10768600	1938727	9047830	0.7950	1.2	Null
B8I366	RUMCH Homocysteine S-methyltransferase	46200081	66817418	113066870	359280913	402812992	196710945	0.0005	4.2	Increase
B8I367	RUMCH AMP-dependent synthetase and ligase	679403893	698854417	524624560	301610724	241132976	146010119	0.0152	2.8	Decrease
B8I368	RUMCH Stage 0 sporulation protein A homolog	13994305	37526855	9994851	17221297	31082344	7948487	0.9809	1.1	Null
B8I369	RUMCH Sensor DegS domain protein	1933095	226385	270293	1085155	1002826	302231	0.9455	1.0	Null
B8I370	RUMCH FGE-sulfatase domain-containing protein	102167668	214615186	103017835	27150101	51733028	101931367	0.3788	2.3	Null
B8I371	RUMCH Endo-1,4-beta-xylanase	12916129	13526451	3744011	5560505	3592824	7079856	0.5018	1.9	Null
B8I372	RUMCH Excinuclease ABC C subunit domain protein	435159	627610	3810662	0	0	0	0.0000	NA	Decrease
B8I373	RUMCH Cyclase family protein	0	193100	222231	0	129440	0	0.8089	3.2	Null
B8I374	RUMCH Rubrerythrin	4239311707	8036348306	5369808195	2870670989	1669194988	1563676629	0.0110	2.9	Decrease
B8I375	RUMCH Thioesterase superfamily protein	0	499953	623492	1406458	1637629	476185	0.4176	3.1	Null
B8I376	RUMCH Transcriptional regulator, XRE family	74796	0	0	0	0	0	NA	NA	Null
B8I377	RUMCH NAD-dependent protein deacetylase	32725046	22632699	63659466	64076089	25566319	38564476	0.8782	1.1	Null
B8I381	RUMCH Cysteine desulfurase family protein	0	0	0	0	0	549332	0.2094	NA	Null
B8I382	RUMCH Branched-chain-amino-acid aminotransferase	518311017	412493100	238275257	388481250	1176672098	817512153	0.2951	2.0	Null
B8I384	RUMCH H+-ATPase subunit H	450404102	715436674	184097468	57292783	52139680	90389460	0.0043	6.8	Decrease
B8I386	RUMCH Methyltransferase	0	114682	0	106699	250994	150680	0.4542	4.4	Null
B8I387	RUMCH ATP-dependent DNA helicase RecG	2231100	5065787	142145	2541092	212854	2057429	0.8463	1.5	Null

B8I388	RUMCH 50S ribosomal protein L28	74713769	61415758	83361240	63175	34178	0	0.0000	2254.6	Decrease
B8I389	RUMCH PsbP domain-containing protein	1160267	843160	0	0	0	0	0.0403	NA	Decrease
B8I390	RUMCH Diguanylate cyclase with GAF sensor	1800441	121137	0	423974	352810	0	0.7148	2.5	Null
B8I391	RUMCH Cell division protein FtsA	1795396	23218777	4180418	11798213	3720308	8829936	0.9761	1.2	Null
B8I392	RUMCH Endonuclease III	12691987	29066349	20245978	7740110	5702390	8303726	0.0379	2.9	Decrease
B8I393	RUMCH Glycogen synthase	12352198	13008572	8060065	13474872	22724366	15745113	0.3560	1.6	Null
B8I394	RUMCH Metal dependent phosphohydrolase	0	204170	0	486488	487026	191736	0.3696	5.7	Null
B8I395	RUMCH Peptidase S1 and S6 chymotrypsin/Hap	1131337522	3988588879	1340542102	752434946	568884770	1526222954	0.4157	2.3	Null
B8I396	RUMCH Histidine kinase	1498424	1694237	1039292	1313910	608805	866023	0.5747	1.5	Null
B8I397	RUMCH Stage 0 sporulation protein A homolog	644776	2480356	4256411	49603047	25441679	15164825	0.0001	12.2	Increase
B8I398	RUMCH SH3 type 3 domain protein	7863214	43596192	14594603	7838454	17426004	44168258	0.8486	1.1	Null
B8I399	RUMCH Stage 0 sporulation protein A homolog	50226949	59827125	47007555	2812694310	1584851131	271516562	0.0000	29.7	Increase
B8I3A0	RUMCH Stage IV sporulation protein B	0	0	0	0	186240	214378	0.2524	NA	Null
B8I3A1	RUMCH DNA repair protein RecN	5661233	11835779	11712612	19098901	23840582	8978638	0.2422	1.8	Null
B8I3A2	RUMCH Arginine repressor	30505695	51390405	36019678	28451670	10338320	10271777	0.1432	2.4	Null
B8I3A3	RUMCH NAD kinase	11701090	11527126	15708600	10098151	10365152	8966990	0.6029	1.3	Null
B8I3A4	RUMCH Hemolysin A	0	1385856	956451	256269	1316738	978716	0.9019	1.1	Null
B8I3A5	RUMCH 1-deoxy-D-xylulose-5-phosphate synthase	0	1302372	2244091	2366480	1283978	1645170	0.7939	1.5	Null
B8I3A7	RUMCH Polyprenyl synthetase	22706934	26438895	27071377	16019360	13783765	2128038	0.2769	2.4	Null
B8I3A8	RUMCH Exodeoxyribonuclease 7 small subunit	0	2069408	0	2425212	2085481	1506671	NA	2.9	Null
B8I3A9	RUMCH Exodeoxyribonuclease 7 large subunit	3870346	3331462	18768923	11976507	11380383	5931254	0.8564	1.1	Null
B8I3B0	RUMCH Transcription antitermination protein NusB	121928540	681874168	774338483	78847377	86246415	209337915	0.1492	4.2	Null
B8I3B2	RUMCH Uncharacterized protein	0	0	289496	1928762	4470911	5742465	0.0220	41.9	Increase
B8I3B3	RUMCH Uncharacterized protein	589936	472428	796859	8442717	952473	0	0.3984	5.1	Null
B8I3B4	RUMCH Uncharacterized protein	57047258	129214984	94399475	35029479	34018294	26276521	0.0057	2.9	Decrease
B8I3B5	RUMCH Stage III sporulation protein AG	191830	7251007	2114682	3016068	4935321	1762505	0.9265	1.0	Null
B8I3B6	RUMCH Uncharacterized protein	327048	0	419842	330299	214185	0	0.9016	1.4	Null
B8I3C1	RUMCH Stage III sporulation protein AA	0	671280	736567	1084310	1052360	2793947	0.3456	3.5	Null
B8I3C3	RUMCH ABC transporter related	244168	1721887	23006564	1814056	421805	5041761	0.5535	3.4	Null
B8I3C4	RUMCH Transcriptional regulator, GntR family	0	0	0	87485	0	505664	0.1849	NA	Null
B8I3C5	RUMCH Uncharacterized protein	21478478	21936113	83327256	5131847	4133610	11812809	0.0387	6.0	Decrease
B8I3C6	RUMCH Uncharacterized protein	0	0	0	521617	383384	4347830	NA	NA	Null
B8I3C7	RUMCH Elongation factor P	7971893316	13949608533	3809098134	3131001978	14775226587	13210990948	0.7952	1.2	Null
B8I3C8	RUMCH Peptidase M24	26426767	27606286	29811967	20922202	30187767	16277537	0.7578	1.2	Null
B8I3C9	RUMCH 3-dehydroquinate dehydratase	33922931	9310444	25271947	38220164	29869965	19926340	0.7969	1.3	Null
B8I3D0	RUMCH Xylose isomerase domain protein TIM barrel	48830763	32087735	25552311	10227425	11394970	6224204	0.0088	3.8	Decrease
B8I3D1	RUMCH M18 family aminopeptidase	182400618	51377742	92388690	39167350	35384030	3769249	0.1280	4.2	Null
B8I3D2	RUMCH Histidinol-phosphatase	0	0	0	118472	91996	110921	0.0749	NA	Null
B8I3D4	RUMCH Uncharacterized protein	1002889	12379165	6182943	3199891	8464129	2029936	0.8611	1.4	Null
B8I3D5	RUMCH tRNA-specific 2-thiouridylase MnmA	7581262	4154273	3118462	9164022	7770774	7028009	0.4761	1.6	Null
B8I3D6	RUMCH FeS cluster assembly scaffold protein NifU	163958523	1084708996	483196451	56390599	40031145	32166578	0.0000	13.5	Decrease
B8I3D7	RUMCH Cysteine desulfurase IscS	124779427	1123196909	881040737	541568722	724337898	476889959	0.9578	1.2	Null
B8I3D8	RUMCH Transcriptional regulator, BadM/Rrf2 family	17459978	23478654	22812033	29312375	18058387	5268812	0.8481	1.2	Null
B8I3D9	RUMCH Uncharacterized protein	0	734018	211475	0	163743	399193	0.9408	1.7	Null
B8I3E0	RUMCH Uncharacterized protein	14935857	1193205	2005614	612071	0	0	0.0918	29.6	Null
B8I3E1	RUMCH AAA ATPase central domain protein	2790327	4199462	4286088	2351888	1965738	3092835	0.5609	1.5	Null
B8I3E2	RUMCH Copper amine oxidase domain protein	79634269	103100601	124862041	181728457	132394665	173547188	0.1600	1.6	Null
B8I3E3	RUMCH Peptidase S1 and S6 chymotrypsin/Hap	507217065	737345828	1052595878	528190757	365202294	311530945	0.1756	1.9	Null
B8I3E5	RUMCH Nucleotidyl transferase	1548501	0	0	0	0	0	NA	NA	Null
B8I3E6	RUMCH Elongation factor 4	15325541	11269746	4553150	83409291	75014451	15380716	0.0339	5.6	Increase
B8I3E7	RUMCH Copper amine oxidase domain protein	1447478839	2622519988	958541945	1565918536	994679582	645398487	0.5396	1.6	Null
B8I3E9	RUMCH Stage II sporulation protein P	74092	388075	481265	525286	359543	424029	0.6565	1.4	Null
B8I3F0	RUMCH CoA-substrate-specific enzyme activase	2684234	10210350	4407035	8065035	28827942	12424880	0.1315	2.9	Null
B8I3F1	RUMCH Aspartate--tRNA ligase	35882952	26667968	15140351	86499994	114101013	48539360	0.0533	3.2	Increase
B8I3F2	RUMCH Histidine--tRNA ligase	12999617	44434194	40950384	39867181	92007001	130957436	0.1275	2.7	Null
B8I3F3	RUMCH Radical SAM domain protein	241891	0	0	320948	92875	2395536	0.2917	11.6	Null
B8I3F4	RUMCH Beta-lactamase domain protein	11316000	70606247	65996581	431314079	371337472	329847004	0.0004	7.7	Increase
B8I3F5	RUMCH D-aminoacyl-tRNA deacylase	0	276961	9216543	0	0	0	NA	NA	Null
B8I3F6	RUMCH (p)ppGpp synthase	0	2715077	0	271225	693678	838978	0.9560	1.5	Null
B8I3F7	RUMCH Adenine phosphoribosyltransferase	95404256	114363177	164989397	152423269	95790671	116941497	0.9342	1.0	Null
B8I3F8	RUMCH Single-stranded-DNA-specific exonuclease RecJ	0	0	0	0	50698	0	NA	NA	Null
B8I3F9	RUMCH DNA polymerase III, delta subunit	608875	0	0	100632	196248	0	0.8427	2.1	Null
B8I3G1	RUMCH NLP/P60 protein	1928650727	5877236101	2634026065	725843352	837738402	349161702	0.0023	5.5	Decrease
B8I3G2	RUMCH GCN5-related N-acetyltransferase	0	0	0	94056	107533	162721	0.0559	NA	Null
B8I3G4	RUMCH Transcriptional regulator, MarR family	0	0	0	799665	1699511	728013	0.0000	NA	Increase
B8I3G5	RUMCH YhgE/Pip C-terminal domain protein	45972928	17276515	22993002	3113018	6563639	2356106	0.0027	7.2	Decrease
B8I3G7	RUMCH O-acetylhomoserine/O-acetylserine sulfhydrylase	57795520	171204352	287672423	169993189	112611860	75280418	0.7464	1.4	Null
B8I3G8	RUMCH Transcriptional regulator, AsnC family	3823666	3883386	1414307	8765527	7429814	4838051	0.1960	2.3	Null
B8I3G9	RUMCH Rqc2 homolog RqcH	12148612	12894249	8827796	11911427	5909462	8249455	0.7341	1.3	Null
B8I3H0	RUMCH Aminotransferase class I and II	86953282	85465385	94803882	62413714	48785922	19742733	0.2016	2.0	Null
B8I3H1	RUMCH 3-demethylubiquinone-9 3-methyltransferase	0	0	0	214428	368292	0	0.2055	NA	Null
B8I3H2	RUMCH DUF362 domain-containing protein	0	0	0	0	14485	242529	0.2918	NA	Null
B8I3H7	RUMCH Glycoside hydrolase family 43	50856069	25321671	9625208	87005292	94023219	3364988	0.6140	2.1	Null
B8I3H9	RUMCH Uncharacterized protein	1750744	14046272	9226687	9460357	10220474	3882993	0.9711	1.1	Null
B8I3I0	RUMCH DUF4157 domain-containing protein	1616233	639562	70449	387379	424267	624611	0.7445	1.6	Null
B8I3I1	RUMCH DNA ligase	4297996	2034278	5739161	11495975	11044097	8501931	0.0854	2.6	Null
B8I3I3	RUMCH Germination protease	3909612	5451870	6548006	7691623	6366524	6192929	0.3668	1.3	Null
B8I3I4	RUMCH 30S ribosomal protein S20	217649020	563368850	131780737	6476980	2406763	4374359	0.0000	68.8	Decrease
B8I3I5	RUMCH Heme chaperone HemW	0	0	0	0	132906	0	NA	NA	Null
B8I3I7	RUMCH Stage 0 sporulation protein A homolog	0	141942	0	1342728	535174	491075	0.0988	16.7	Null
B8I3I9	RUMCH Periplasmic binding protein/LacI transcriptional regulator	4636354	7122881	4482105	4375796	2892769	2008648	0.2825	1.8	Null
B8I3J1	RUMCH ABC transporter related	182023946	249161370	74071787	175296378	95767731	54499976	0.5983	1.6	Null
B8I3J2	RUMCH Putative solute-binding component of ABC transporter	1311425816	3809600278	4937587187	1995913713	1879768463	1783251258	0.4472	1.8	Null
B8I3J4	RUMCH Thiamine-phosphate synthase	697587	391333	972727	1878918	2602182	292745	0.3790	2.3	Null
B8I3J5	RUMCH Hydroxyethylthiazole kinase	2711948	938878	2140454	244865	2101080	3859342	0.9146	1.1	Null
B8I3J6	RUMCH Phosphomethylpyrimidine synthase	1366164	2058635	2085973	1655003	1095656	1038210	0.4957	1.5	Null
B8I3J7	RUMCH Phosphomethylpyrimidine kinase	474890	1401551	872336	1020273	1118753	321320	0.9692	1.1	Null
B8I3K0	RUMCH Ferric uptake regulator, Fur family	0	3496932	0	647289	395657	0	0.7816	3.4	Null
B8I3K1	RUMCH Uncharacterized protein	0	0	0	0	0	81643	NA	NA	Null
B8I3K2	RUMCH Ferrous iron transport protein B	173532	2005737	668197	1060553	2815775	2201381	0.3592	2.1	Null
B8I3K3	RUMCH FeoA family protein	0	0	0	339866	401848	372781	0.0003	NA	Increase
B8I3K5	RUMCH Uncharacterized protein	0	503929	786546	0	535333	492575	0.9972	1.3	Null
B8I3K6	RUMCH Mg2 transporter protein CorA family protein	2938888	721767	851234	544539	2836698	0	0.8796	1.3	Null
B8I3K7	RUMCH 6,7-dimethyl-8-ribityllumazine synthase	351406982	169349617	188497633	194668994	284306747	216402924	0.9993	1.0	Null
B8I3K8	RUMCH Riboflavin biosynthesis protein RibBA	305853936	852904608	188004023	48932223	45044745	12608536	0.0003	12.6	Decrease
B8I3K9	RUMCH Riboflavin synthase, alpha subunit	1153068	3034280	3310939	2586472	2265596	1128457	0.8549	1.3	Null
B8I3L0	RUMCH Riboflavin biosynthesis protein RibD	121664072	144566899	315497065	331540624	214542737	215548315	0.5637	1.3	Null
B8I3L5	RUMCH Parallel beta-helix repeat protein	39766744	31742914	29851626	69112608	56284015	140365283	0.1050	2.6	Null
B8I3L6	RUMCH Abasic site processing protein	0	0	0	71508	0	0	NA	NA	Null
B8I3L8	RUMCH Uncharacterized protein	0	0	0	223653	0	0	NA	NA	Null
B8I3M0	RUMCH Cellulase	59226881	93178585	35797305	65901540	34409214	59167257	0.9113	1.2	Null
B8I3M1	RUMCH Uncharacterized protein	12797500	77855680	58633753	3498803	1657667	3259878	0.0000	17.7	Decrease
B8I3M2	RUMCH Uncharacterized protein	86871843	71214522	54313461	466208	7115537	5382047	0.0023	16.4	Decrease
B8I3M3	RUMCH Uncharacterized protein	0	320653	106881	0	0	0	0.2734	NA	Null
B8I3M4	RUMCH Uncharacterized protein	410247294	254029067	279983640	175703298	142788053	111672964	0.1193	2.2	Null
B8I3M5	RUMCH RNA polymerase, sigma 28 subunit, FliA/WhiG	1380608	5686312	5619599	0	302943	0	0.0388	41.9	Decrease
B8I3M7	RUMCH Probable chemoreceptor glutamine deamidase CheD	12513857	12062455	41149133	3388656	4499498	3823653	0.0066	5.6	Decrease
B8I3M8	RUMCH CheC, inhibitor of MCP methylation	3518072	4881512	6778089	16568875	16367484	7501703	0.0191	2.7	Increase
B8I3M9	RUMCH CheW protein	3536211	8524016	1121455	81145639	79624389	78525939	0.0000	18.2	Increase
B8I3N0	RUMCH Chemotaxis protein CheA	441808864	694286614	380503359	1101485685	1037023102	553626114	0.1991	1.8	Null
B8I3N1	RUMCH Protein-glutamate methylesterase/protein-glutamine glutaminase	18026591	161525640	38328276	9208316	19515078	8366359	0.0545	5.9	Null
B8I3N2	RUMCH Cobyrinic acid ac-diamide synthase	550623	1933397	662355	1975217	4137512	2747459	0.1028	2.8	Null

B8I3N3	RUMCH Flagella-associated GTP-binding protein	1943598	4596428	5456395	8232579	6906347	14118462	0.0981	2.4	Null
B8I3N4	RUMCH Flagellar biosynthesis protein FlhA	1769201	0	0	1146850	457898	5458127	0.6279	4.0	Null
B8I3N5	RUMCH Flagellar biosynthetic protein FlhB	0	0	0	0	73623	0	NA	NA	Null
B8I3N8	RUMCH Flagellar biosynthetic protein FliP	363065	0	0	0	0	0	0.2652	NA	Null
B8I3N9	RUMCH Uncharacterized protein	322597	153784	0	0	0	0	0.2439	NA	Null
B8I3P0	RUMCH Stage 0 sporulation protein A homolog	526821432	334175448	443259747	491531829	275729825	177342513	0.6256	1.4	Null
B8I3P1	RUMCH CheC, inhibitor of MCP methylation / FliN fusion protein	0	1235824	1710783	4055324	2093616	4261488	0.3673	3.5	Null
B8I3P2	RUMCH Flagellar motor switch protein FliM	0	0	0	298934	133374	0	0.2613	NA	Null
B8I3P3	RUMCH Flagellar protein FliL	8901948	6453746	24046462	40892103	33332781	21669779	0.1512	2.4	Null
B8I3P5	RUMCH Flagellar hook protein FlgE	22615374	9680351	19173527	4109674	4642024	3425134	0.0069	4.2	Decrease
B8I3P6	RUMCH Flagellar operon protein	557183	546010	0	0	0	356815	0.8118	3.1	Null
B8I3P7	RUMCH Basal-body rod modification protein FlgD	7150220	10551320	21472732	8899730	6038884	2363647	0.2697	2.3	Null
B8I3P8	RUMCH Flagellar hook-length control protein	3948736	5488977	18119842	8170533	2779646	54493175	0.3945	2.4	Null
B8I3P9	RUMCH Uncharacterized protein	59694888	61594520	41273032	16076091	24080455	21293653	0.0561	2.6	Null
B8I3Q0	RUMCH Flagellar FliJ protein	0	0	0	280929	0	515741	0.1280	NA	Null
B8I3Q1	RUMCH H(+)-transporting two-sector ATPase	641887	1656255	1129758	1563063	4480119	589331	0.4319	1.9	Null
B8I3Q2	RUMCH Flagellar assembly protein FliH	289329	2988742	3307591	1002102	768974	1697963	0.6901	1.9	Null
B8I3Q3	RUMCH Flagellar motor switch protein FliG	0	0	0	0	73017	0	NA	NA	Null
B8I3Q4	RUMCH Flagellar M-ring protein FliF	16852722	113247399	17910014	10939182	41077402	8782731	0.4797	2.4	Null
B8I3Q5	RUMCH Flagellar hook-basal body complex protein FliE	6883837	2727053	0	960152	647433	5513910	0.9286	1.3	Null
B8I3Q6	RUMCH Flagellar basal-body rod protein FlgC	1407024	663001	8814938	1266596	1538741	507298	0.3007	3.3	Null
B8I3Q7	RUMCH Flagellar basal body rod protein FlgB	0	484588	403714	461098	485443	0	0.9611	1.1	Null
B8I3Q8	RUMCH Methylenetetrahydrofolate--tRNA-(uracil-5-)-methyltransferase T	13512894	10618638	21879085	14849456	1433187	8904798	0.5977	1.8	Null
B8I3Q9	RUMCH DNA topoisomerase 1	420446809	226405105	149181018	33507170	32719346	35667476	0.0003	7.8	Decrease
B8I3R0	RUMCH DNA protecting protein DprA	0	0	0	125220	34879	34591	NA	NA	Null
B8I3R2	RUMCH Chromosomal replication initiator protein DnaA	4723323	11700398	8985228	20213800	24303736	8047401	0.1838	2.1	Null
B8I3R3	RUMCH Beta sliding clamp	6562913	5512196	14166672	39687069	32813166	67324018	0.0021	5.3	Increase
B8I3R4	RUMCH RNA-binding S4 domain protein	1050619	487936	0	0	0	2614518	0.8789	1.7	Null
B8I3R6	RUMCH Uncharacterized protein	58521513	27370737	39122383	28024539	15817136	28332751	0.4481	1.7	Null
B8I3R7	RUMCH DNA gyrase subunit B	19006180	21163378	5736919	17968960	26403536	2284773	0.9988	1.0	Null
B8I3R8	RUMCH Cobyrinic acid ac-diamide synthase	0	65593	0	579152	511263	0	0.3137	16.6	Null
B8I3R9	RUMCH ParB-like partition protein	21354970	227621024	28557171	14565741	11812988	31862277	NA	4.8	Null
B8I3S1	RUMCH TPR repeat-containing protein	25205189	34947258	38729068	31219135	37954702	27067293	0.9169	1.0	Null
B8I3S3	RUMCH HAD-superfamily hydrolase, subfamily IA, variant 1	256192	520905	4533689	2238617	1251289	0	0.8782	1.5	Null
B8I3S7	RUMCH Pyruvate flavodoxin/ferredoxin oxidoreductase domain protein	8772636585	22403299466	11638736970	12939842145	14507314168	8465541101	0.8887	1.2	Null
B8I3S8	RUMCH Spore coat protein, CotS family	0	0	0	0	0	227007	0.2991	NA	Null
B8I3S9	RUMCH Formate--tetrahydrofolate ligase	7102871	5408414	3709709	8320427	11676331	9614683	0.2682	1.8	Null
B8I3T1	RUMCH Uncharacterized protein	2175937	19989217	13801414	14366248	26010539	290018	0.9127	1.1	Null
B8I3T2	RUMCH Peptidoglycan-binding LysM	48032355	63519499	31959002	46781332	261063901	102006908	0.1698	2.9	Null
B8I3T3	RUMCH 4-diphosphocytidyl-2-C-methyl-D-erythritol kinase	4313788	3706068	2181685	5137883	3379672	3191763	0.8354	1.1	Null
B8I3T4	RUMCH Transcriptional regulator, GntR family	501746284	475166885	345660509	280887179	292794824	325892603	0.5338	1.5	Null
B8I3T5	RUMCH AAA ATPase central domain protein	1700989	4960938	5300685	8711570	13287749	7133102	0.0752	2.4	Null
B8I3T6	RUMCH Phage shock protein A, PspA	394516158	525065417	134971069	42276317	254994359	343560507	0.7614	1.6	Null
B8I3T7	RUMCH GCN5-related N-acetyltransferase	0	0	0	481223	0	0	0.2524	NA	Null
B8I3T8	RUMCH Uncharacterized protein	21591977	15322879	1383264	2339004	1013358	3001574	0.0932	6.0	Null
B8I3T9	RUMCH Uncharacterized protein	23219804	9608388	8229538	20461201	18505205	10121889	0.8764	1.2	Null
B8I3U0	RUMCH GAF domain-containing protein	167138	1124780	880830	644376	51268686	495132	NA	24.1	Null
B8I3U2	RUMCH Metallophosphoesterase	0	0	0	0	0	194099	NA	NA	Null
B8I3U3	RUMCH AAA	0	0	0	0	0	301758	0.2725	NA	Null
B8I3U4	RUMCH NLP/P60 protein	935414	1215335	1634437	3234882	3212251	5675589	0.0144	3.2	Increase
B8I3U5	RUMCH Monogalactosyldiacylglycerol synthase	0	0	0	0	0	67211	NA	NA	Null
B8I3U6	RUMCH Spore cortex-lytic enzyme	2070611	1056322	2507716	2176280	2272975	1340471	0.9475	1.0	Null
B8I3U7	RUMCH Germination protein YpeB	6940963	22802144	14577214	39949568	19485209	10403734	0.4827	1.6	Null
B8I3U8	RUMCH Transcriptional regulator, MarR family	0	1985640	594243	0	0	0	0.0353	NA	Decrease
B8I3U9	RUMCH Peptide deformylase	3797233	218432	1688310	1183636	0	1899165	0.8029	1.9	Null
B8I3V0	RUMCH S-layer domain protein	5823414	19529077	11584398	11330308	6569027	5817777	0.5858	1.6	Null
B8I3V1	RUMCH Abortive infection protein	0	73835	62433	109726	90258	0	0.8730	1.5	Null
B8I3V2	RUMCH IstB domain protein ATP-binding protein	449828	22970563	211820	750969	651645	267487	NA	14.2	Null
B8I3V3	RUMCH Primosome, DnaD subunit	765476	953228	0	0	127005	144374	0.4650	6.3	Null
B8I3V4	RUMCH Ferritin	258543962	111318472	410738572	220246255	230050348	178387860	0.8486	1.2	Null
B8I3V5	RUMCH Extracellular solute-binding protein family 5	13547357	16436684	4872672	1652224	1957035	721683	0.0010	8.0	Decrease
B8I3V6	RUMCH Oligopeptide/dipeptide ABC transporter, ATPase subunit	7629937	38525	3446856	879129	1072135	1244795	0.3848	3.5	Null
B8I3V7	RUMCH Oligopeptide/dipeptide ABC transporter, ATPase subunit	1655404	2415862	4264361	2615833	541402	926887	0.4176	2.0	Null
B8I3V9	RUMCH Binding-protein-dependent transport systems inner membrane co	0	0	0	175451	111544	0	0.2988	NA	Null
B8I3W0	RUMCH Methyl-accepting chemotaxis sensory transducer	13605237	1973654	5867960	11930866	25617852	47735496	0.1658	4.0	Null
B8I3W1	RUMCH Diacylglycerol kinase catalytic region	0	0	0	238906	0	0	NA	NA	Null
B8I3W2	RUMCH Nuclease SbcCD subunit D	1111888	6842514	0	2025193	557473	4764946	0.9692	1.1	Null
B8I3W3	RUMCH SMC domain protein	336839	923145	723316	12577646	3486640	35653296	0.0000	26.1	Increase
B8I3W4	RUMCH 1,4-alpha-glucan branching enzyme GlgB	1087295	277427	0	284126	240968	280662	0.7947	1.7	Null
B8I3W5	RUMCH Periplasmic sugar-binding protein	785427788	3182074	4364052	5092994	5622593	4500934	NA	52.1	Null
B8I3W6	RUMCH 30S ribosomal protein S6	69948168	250147650	387433254	157355718	267451431	364188162	0.7906	1.1	Null
B8I3W7	RUMCH Single-stranded DNA-binding protein	25263155	21579433	68133158	9458717	13498669	6000982	0.0325	4.0	Decrease
B8I3W8	RUMCH 30S ribosomal protein S18	348633564	660090969	1225041761	118817285	99739595	94608455	0.0001	7.1	Decrease
B8I3W9	RUMCH Transcriptional regulator, XRE family	15478010	4544909	23290638	7901040	5908375	4041364	0.2664	2.4	Null
B8I3X2	RUMCH Transcriptional repressor NrdR	71354781	34937348	31666053	13453323	7000755	5059840	0.0047	5.4	Decrease
B8I3X3	RUMCH Sporulation protein, YlmC/YmxH family	3975547	165406	0	0	0	1639613	NA	2.5	Null
B8I3X4	RUMCH RNA polymerase sigma factor	253547	221626	391275	1483587	498108	0	0.6788	2.3	Null
B8I3X5	RUMCH RNA polymerase sigma factor	1323814	4732441	5947661	2309331	2595957	3038665	0.7495	1.5	Null
B8I3X6	RUMCH Sporulation sigma-E factor-processing peptidase	0	458822	743754	0	404926	193568	0.8648	2.0	Null
B8I3X7	RUMCH Cell division protein FtsZ	96303666	264825111	276724504	729590808	436739066	447192988	0.0378	2.5	Increase
B8I3X8	RUMCH Cell division protein FtsA	60511037	25868472	36062449	32657712	20282180	19665373	0.4242	1.7	Null
B8I3Y0	RUMCH Polypeptide-transport-associated domain protein FtsQ-type	5126433	1197728	3888671	4384195	6556191	2202500	0.8307	1.3	Null
B8I3Y1	RUMCH Lipid II isoglutaminyl synthase (glutamine-hydrolyzing) subunit Mu	2139907	1731919	0	648047	709686	2456254	0.9790	1.0	Null
B8I3Y2	RUMCH Lipid II isoglutaminyl synthase (glutamine-hydrolyzing) subunit Ga	4500844	4455606	812601	5757651	1911766	2055047	0.9913	1.0	Null
B8I3Y3	RUMCH Phospho-2-dehydro-3-deoxyheptonate aldolase	624787697	2004067649	1177037563	1025487706	937569298	683456745	0.6618	1.4	Null
B8I3Y4	RUMCH Uncharacterized protein	23598954	26268683	7159637	40996839	51027195	21349583	0.3429	2.0	Null
B8I3Y5	RUMCH Lytic transglycosylase catalytic	1013639	10641385	15887973	3718215	72194	3247573	0.3935	3.9	Null
B8I3Y6	RUMCH Dephospho-CoA kinase	302081	947256	688218	364501	1794934	1044541	0.5079	1.7	Null
B8I3Y7	RUMCH DNA polymerase I	418362080	1624572	35479827	24359767	15199086	2216029	0.1015	10.9	Null
B8I3Y8	RUMCH Putative transmembrane anti-sigma factor	4292656	8047744	3135657	5078019	3576701	3214577	0.7765	1.3	Null
B8I3Z0	RUMCH Coat F domain protein	27655930	2650215	7297491	19272820	10869080	939425	0.8494	1.2	Null
B8I3Z3	RUMCH Tyrosine--tRNA ligase	13023612	45590734	31229125	5085164	6909923	13711554	0.1384	3.5	Null
B8I3Z4	RUMCH Trk system potassium uptake protein TrkA	0	0	0	390717	603649	1303307	0.0000	NA	Increase
B8I3Z5	RUMCH Trk system potassium uptake protein TrkA	4140161	7290594	6592798	14833844	12551793	8107526	0.0379	2.0	Increase
B8I400	RUMCH Uncharacterized protein	0	292971	0	0	0	0	0.3129	NA	Null
B8I401	RUMCH Short-chain dehydrogenase/reductase SDR	794458	0	0	0	158375	0	NA	5.0	Null
B8I402	RUMCH ThiJ/PfpI domain protein	5198316	8536609	3617326	9803237	7492017	10720438	0.3160	1.6	Null
B8I404	RUMCH Adenylate cyclase	0	64764	0	0	101484	89083	0.7468	2.9	Null
B8I405	RUMCH GatB/Yqey domain protein	154649032	487268944	129452846	62677285	39116685	30717687	0.0049	5.8	Decrease
B8I406	RUMCH 30S ribosomal protein S21	170364460	197731562	56590236	34862068	17145930	60492716	0.1336	3.8	Null
B8I407	RUMCH Histidine triad (HIT) protein	173553428	184123320	272521212	63343563	48054337	102362961	0.0779	2.9	Null
B8I408	RUMCH Alanine--tRNA ligase	617852495	632326251	645492488	175721158	232255835	165092255	0.0004	3.3	Decrease
B8I409	RUMCH HAD-superfamily hydrolase, subfamily IA, variant 3	1699385	1165645	0	511167	3145015	619972	0.8698	1.5	Null
B8I410	RUMCH Arginine biosynthesis bifunctional protein ArgJ	13320601	7013115	8898912	25790016	5251493	6662992	0.8382	1.3	Null
B8I411	RUMCH CheW protein	9740782	4746544	9191838	141278265	130138434	109788353	0.0000	16.1	Increase
B8I412	RUMCH Methyl-accepting chemotaxis sensory transducer	0	71365	0	380942	136549	0	0.5414	7.3	Null
B8I413	RUMCH Phosphate-binding protein	4279188	11873685	1474596	416732	1901202	4627633	0.5589	2.5	Null
B8I416	RUMCH Phosphate import ATP-binding protein PstB	3879041	8742875	8220035	6534738	6259661	4852404	0.8972	1.2	Null
B8I417	RUMCH Phosphate-specific transport system accessory protein PhoU	16401817	11225355	2343263	3140393	1888409	635439	0.0598	5.3	Null
B8I418	RUMCH Stage 0 sporulation protein A homolog	1193918	1101560	2511719	1704624	3903788	1101153	0.6433	1.4	Null
B8I421	RUMCH Glycosyltransferase 36	156218543	332289785	70367337	70200796	156481157	278124276	0.9769	1.1	Null
B8I422	RUMCH Binding-protein-dependent transport systems inner membrane co	7309229	2812990	9129300	7667250	7726116	2829757	0.9679	1.1	Null

B8I424	RUMCH Extracellular solute-binding protein family 1	1840624130752	1064986885709	1394287951793	744407174526	725283519629	491996180295	0.1258	2.2	Null
B8I425	RUMCH Stage 0 sporulation protein A homolog	2329317	7245403	1403087	1931367	7584621	2305969	0.8939	1.1	Null
B8I426	RUMCH Putative sensor with HAMP domain	0	0	0	268068	609009	341626	0.0004	NA	Increase
B8I427	RUMCH Extracellular solute-binding protein family 1	5073673	17534322	9218906	13577714	34279435	27818419	0.1483	2.4	Null
B8I428	RUMCH ATPase AAA-2 domain protein	14845606	50785956	36995778	19981426	31982530	48806712	0.8804	1.0	Null
B8I429	RUMCH Phosphate transporter	0	0	0	0	641208	0	0.2109	NA	Null
B8I430	RUMCH Uncharacterized protein	55973198	69349611	107748340	45795209	49149579	17132859	0.2324	2.1	Null
B8I431	RUMCH UvrABC system protein A	2127883	11255566	2701391	11370268	6864921	4998767	0.6146	1.4	Null
B8I433	RUMCH Resolvase domain protein	1535743	602765	198498	1224825	1088300	864629	0.8377	1.4	Null
B8I434	RUMCH Arabinogalactan endo-beta-1,4-galactanase	2866212	5323782	5952264	14784657	9640134	12157413	0.0080	2.6	Increase
B8I436	RUMCH Signal transduction histidine kinase regulating citrate/malate met	0	0	0	0	0	137620	NA	NA	Null
B8I439	RUMCH NLP/P60 protein	113607801	91379648	135144678	79873531	103309552	62337391	0.5755	1.4	Null
B8I440	RUMCH Glycerol-3-phosphate dehydrogenase [NAD(P)+]	10760221	29391628	17500314	47798225	29884662	13806467	0.4280	1.6	Null
B8I442	RUMCH GTPase Der	14876493	21579044	35148915	93630626	63314284	63865616	0.0027	3.1	Increase
B8I443	RUMCH Uncharacterized protein	0	302121	0	377629	761978	106429	0.5244	4.1	Null
B8I446	RUMCH Uncharacterized protein	273617	0	453735	1212399	370350	32616	0.7427	2.2	Null
B8I447	RUMCH Acetate kinase	383879928	668790260	676481916	587160322	427946216	436342408	0.8486	1.2	Null
B8I448	RUMCH Phosphate acetyltransferase	64688963	96105055	73036493	50472975	51724219	32906001	0.1680	1.7	Null
B8I449	RUMCH PABS domain-containing protein	25339872	144049264	91379627	9921587	12366566	7346299	0.0005	8.8	Decrease
B8I450	RUMCH Glutamate--tRNA ligase	19508849	43799496	45727319	86562565	131254334	82149982	0.0114	2.8	Increase
B8I451	RUMCH Phosphoglycerate mutase (2,3-diphosphoglycerate-independent)	21251575	67755276	42645490	34551752	31602015	14977210	0.5267	1.6	Null
B8I454	RUMCH Fascin family protein	635505	906096	1134613	11074858	12669181	6453781	0.0000	11.3	Increase
B8I456	RUMCH Stage 0 sporulation protein A homolog	5486916	94572016	16697742	44866548	40940329	24467811	0.9652	1.1	Null
B8I457	RUMCH Uncharacterized protein	15288819	1740881	0	3744890	1400100	797834	0.5831	2.9	Null
B8I458	RUMCH Uncharacterized protein	942715	0	0	0	0	0	NA	NA	Null
B8I465	RUMCH Alpha/beta hydrolase fold-3 domain protein	0	2471018	927813	991300	563054	72156	0.7766	2.1	Null
B8I466	RUMCH Uncharacterized protein	72141	0	189559	955074	1012656	262679	0.1201	8.5	Null
B8I468	RUMCH ABC transporter related	11414958	107281677	103650927	182577865	124351582	155969148	0.3007	2.1	Null
B8I470	RUMCH Transcriptional regulator, TetR family	221081	266174	298099	153356	402470	283230	0.8927	1.1	Null
B8I471	RUMCH Cellulase	948823753	581367342	686780433	754084037	1359371872	903385988	0.5906	1.4	Null
B8I473	RUMCH 5-formyltetrahydrofolate cyclo-ligase	3644812	3977458	2106448	2905288	1817792	4570920	0.9692	1.0	Null
B8I476	RUMCH 5-methylthioadenosine/S-adenosylhomocysteine deaminase	4759993	18222848	17608993	98254264	93816545	57903889	0.0002	6.2	Increase
B8I477	RUMCH Adenosylhomocysteinase	187346518	430171126	297032307	635531044	698368303	147964354	0.4773	1.6	Null
B8I478	RUMCH Carbohydrate-binding family 25 protein	1199392749	914820150	1174673114	696097090	391757253	318519094	0.0752	2.3	Null
B8I479	RUMCH SAM dependent methyltransferase	993497	4142244	2334176	4189567	3368475	5460495	0.3023	1.7	Null
B8I480	RUMCH Pseudouridine synthase	495649	8527219	16398313	0	1014727	928528	0.1780	13.1	Null
B8I481	RUMCH RNA methylase, NOL1/NOP2/sun family	175421	730079	0	1751323	2255742	1409805	0.2156	6.0	Null
B8I483	RUMCH Uncharacterized protein	12352782	32010494	13434419	21552797	13721951	15532944	0.9585	1.1	Null
B8I484	RUMCH Transcriptional regulator, GntR family	3549303	4967503	2421995	1681863	1266897	958965	0.0427	2.8	Decrease
B8I485	RUMCH Aconitate hydratase A	407908815	537015374	370915692	622146875	444495256	356984720	0.8086	1.1	Null
B8I486	RUMCH Pyruvate carboxyltransferase	80204024	157352258	89389964	56018559	101638600	58507260	0.5747	1.5	Null
B8I487	RUMCH Transposase IS3/IS911 family protein	10161753	4667414	39724338	951875	70469	388943	0.0002	38.7	Decrease
B8I488	RUMCH Methyl-accepting chemotaxis sensory transducer	286149365	172075870	129280212	295075323	240747304	157077222	0.8463	1.2	Null
B8I489	RUMCH Phosphoribosylamine--glycine ligase	0	3131232	2218190	1256700	4038643	565211	0.9276	1.1	Null
B8I490	RUMCH Bifunctional purine biosynthesis protein PurH	410841842	995657227	317683737	322821373	404868802	257053189	0.4551	1.8	Null
B8I491	RUMCH Phosphoribosylglycinamide formyltransferase	0	0	0	1200190	1397285	1361069	0.0000	NA	Increase
B8I492	RUMCH Phosphoribosylformylglycinamidine cyclo-ligase	37393548	103010076	132632866	58176506	17362434	54456871	0.4312	2.1	Null
B8I493	RUMCH Amidophosphoribosyltransferase	3034448	27031088	13639234	7176255	5646362	5792455	0.3812	2.3	Null
B8I494	RUMCH N5-carboxyaminoimidazole ribonucleotide mutase	72804907	21925926	34056748	63120968	76382411	13132571	0.9255	1.2	Null
B8I496	RUMCH Hydrolase (Metallo-beta-lactamase superfamily)-like protein	565043	428921	0	1940977	977375	1022150	0.3879	4.0	Null
B8I4A0	RUMCH Type III restriction protein res subunit	863526	0	0	0	0	0	NA	NA	Null
B8I4A6	RUMCH SIR2	0	0	0	162236	351971	83249	0.0190	NA	Increase
B8I4A8	RUMCH Uncharacterized protein	0	0	0	312312	0	0	0.2951	NA	Null
B8I4B0	RUMCH Uncharacterized protein	0	0	0	366082	0	323649	0.1580	NA	Null
B8I4B1	RUMCH DUF2007 domain-containing protein	6303724	4359572	5879655	6447918	7204948	6033016	0.6884	1.2	Null
B8I4B2	RUMCH Prephenate dehydratase	0	186013	378864	2027345	2011193	527151	0.1121	8.1	Null
B8I4B3	RUMCH Uncharacterized protein	543074	0	0	1712691	0	0	NA	3.2	Null
B8I4B4	RUMCH Cyclic-di-AMP phosphodiesterase	0	459787	0	1539697	809291	529233	0.3581	6.3	Null
B8I4B5	RUMCH 50S ribosomal protein L9	4831525080	4164422359	2739570492	580672193	552532172	324759042	0.0000	8.0	Decrease
B8I4B6	RUMCH Replicative DNA helicase	444066	1695271	4115296	3799596	7925777	7396959	0.1500	3.1	Null
B8I4B7	RUMCH tRNA(Ile)-lysidine synthase	0	238804	699164	321788	1357893	191111	0.6812	2.0	Null
B8I4B8	RUMCH Hypoxanthine phosphoribosyltransferase	846052	7015460	1656219	14272764	12708096	12991282	0.0480	4.2	Increase
B8I4B9	RUMCH ATP-dependent zinc metalloprotease FtsH	64232330	90527138	67991175	83493130	155145502	55653682	0.5890	1.3	Null
B8I4C0	RUMCH 4HBT domain-containing protein	51094422	40259429	113492945	13714525	14849665	4032744	0.0041	6.3	Decrease
B8I4C1	RUMCH S-adenosylmethionine synthase	14548344895	23467347736	24862547835	2245460128	2643658633	3373972221	0.0000	7.6	Decrease
B8I4C2	RUMCH Uncharacterized protein	0	0	0	52766	96714	0	NA	NA	Null
B8I4C3	RUMCH ATP-dependent RecD-like DNA helicase	250524	109142	0	743529	246196	339680	0.4472	3.7	Null
B8I4C4	RUMCH Phosphoribosyltransferase	0	280699	0	1907475	3542321	3154952	0.0353	30.7	Increase
B8I4C5	RUMCH Regulatory protein, MerR	60893942	301555917	19503838	10479304	634042	18636461	0.0587	12.8	Null
B8I4C6	RUMCH Anti-sigma-28 factor FlgM family protein	7794852	14526591	20831776	6444212	6010659	5205165	0.0765	2.4	Null
B8I4C7	RUMCH FlgN family protein	0	1128634	2866265	1771315	3156235	1614291	0.7576	1.6	Null
B8I4C8	RUMCH Flagellar hook-associated protein 1	183577	331790	492222	3460375	607148	191809	0.1968	4.2	Null
B8I4C9	RUMCH Flagellar hook-associated protein 1	0	0	0	4084648	670166	234557	0.0000	NA	Increase
B8I4D0	RUMCH Flagellar hook-associated protein 3	435896	1084862	705310	113504	902671	604185	0.8796	1.4	Null
B8I4D2	RUMCH Flagellar assembly factor FliW	9498665	20070946	15307531	12699244	9910787	13933912	0.8718	1.2	Null
B8I4D3	RUMCH Translational regulator CsrA	0	3078607	12007541	1423674	883995	0	0.4798	6.5	Null
B8I4D4	RUMCH Flagellin	96270704	52084878	137213301	80996082	102806928	67250411	0.9233	1.1	Null
B8I4D5	RUMCH Flagellin	16721239	6056201	38488150	29647418	23045746	6688805	0.9960	1.0	Null
B8I4D6	RUMCH Flagellin	6045737	6909867	8022881	4119639	3537289	8873586	0.8903	1.3	Null
B8I4D7	RUMCH Uncharacterized protein	652289	0	0	0	48584	0	0.4956	13.4	Null
B8I4D8	RUMCH Flagellar protein FlaG protein	3366274	4027021	5292262	1895473	1157909	632312	0.0137	3.4	Decrease
B8I4D9	RUMCH Flagellar hook-associated protein 2	7924876	22982380	12324137	18976932	9590226	6146555	0.8494	1.2	Null
B8I4E0	RUMCH Flagellar secretion chaperone FliS	0	0	0	639451	250963	87495	0.0044	NA	Increase
B8I4E1	RUMCH FlgN family protein	4273061	10373983	13243631	15821549	4630957	7962459	0.9126	1.0	Null
B8I4E2	RUMCH Kelch repeat protein	75625457515	51912875177	66150221143	40069974693	45882901995	42953892154	0.4761	1.5	Null
B8I4E3	RUMCH Hydrolase	4018744	2813324	1281586	960152	1135053	1813078	0.4007	2.1	Null
B8I4E8	RUMCH Type II secretion system protein E	0	1698825	0	0	0	0	NA	NA	Null
B8I4F7	RUMCH ABC transporter related	6051209	9535611	4581687	6209568	5653742	5009010	0.8601	1.2	Null
B8I4F8	RUMCH DEAD/DEAH box helicase domain protein	10402012	48319198	7032170	14613372	10540353	11778830	0.6313	1.8	Null
B8I4F9	RUMCH Transcriptional regulator, AraC family	576197	0	0	384163	183487	450335	0.8799	1.8	Null
B8I4G0	RUMCH 3-isopropylmalate dehydratase large subunit	2988381886	1768421792	2346854605	2021575521	2486123292	304734109	0.6845	1.5	Null
B8I4G1	RUMCH 3-isopropylmalate dehydratase small subunit	877892672	2755844375	3483554431	412340032	381742383	411550524	0.0013	5.9	Decrease
B8I4G2	RUMCH 3-isopropylmalate dehydrogenase	487176823	811736814	309973506	512385214	886742508	1825659480	0.2983	2.0	Null
B8I4G5	RUMCH Cyanophycinase	0	0	0	6648255	0	0	NA	NA	Null
B8I4G6	RUMCH Cyanophycin synthase	1289540	1235509	0	11083135	67114468	2539369	0.0367	32.0	Increase
B8I4G8	RUMCH Bifunctional ligase/repressor BirA	0	332567	136227	738127	1827322	1243859	0.0882	8.1	Null
B8I4G9	RUMCH Uncharacterized protein	60116493	255911116	204506790	122620944	33613967	42302391	0.2570	2.6	Null
B8I4H0	RUMCH Type III pantothenate kinase	0	0	0	1066288	65315	167399	0.0049	NA	Increase
B8I4H1	RUMCH L-lactate dehydrogenase	248275336	268822703	238042983	689140181	669908633	1398963713	0.0035	3.7	Increase
B8I4H2	RUMCH Malic protein NAD-binding	13919458915	10925054532	11012628493	4990892043	4679307767	1895127886	0.0247	3.1	Decrease
B8I4H3	RUMCH Mannitol dehydrogenase domain protein	22995922	43803110	61212080	31389476	22703372	12031592	0.2862	1.9	Null
B8I4H4	RUMCH Mannonate dehydratase	3660284	14008807	10361092	9328681	10540796	1677596	0.8617	1.3	Null
B8I4H5	RUMCH Xylan alpha-1,2-glucuronidase	0	450874	0	0	373914	73451	0.9754	1.0	Null
B8I4H6	RUMCH Transcriptional regulator, AraC family	0	0	0	0	71291	0	NA	NA	Null
B8I4H9	RUMCH Extracellular solute-binding protein family 1	5846209880	12281914993	7125877417	3847615519	3788750755	2891307875	0.0427	2.4	Decrease
B8I4I0	RUMCH Polysaccharide deacetylase	0	282427	1355090	1115011	2606495	1947136	0.3898	3.5	Null
B8I4I4	RUMCH Extracellular solute-binding protein family 1	2460481714	2102895096	1031820174	812647473	443715788	1245326528	0.3183	2.2	Null
B8I4I7	RUMCH Beta-xylanase	0	678675	603842	0	0	0	0.0917	NA	Null
B8I4I9	RUMCH Orn/Lys/Arg decarboxylase major region	0	0	0	75694	1377883	0	0.0834	NA	Null
B8I4J0	RUMCH Thymidylate kinase	0	0	11887943	8090414	2698957	8380667	0.8486	1.6	Null
B8I4J1	RUMCH Uncharacterized protein	4178643	39453095	116413841	60585143	30645557	114963828	0.7578	1.3	Null
B8I4J2	RUMCH Uncharacterized protein	0	859596	0	171976	1020952	0	0.9199	1.4	Null

B8I4J3	RUMCH DNA polymerase III subunit delta'	490279	356803	2213672	1353134	1430318	1309262	0.7077	1.3	Null
B8I4J4	RUMCH PSP1 domain protein	7887030	3662066	464052	11390547	8722732	13583540	0.3404	2.8	Null
B8I4J6	RUMCH Methyltransferase type 11	0	710165	275879	869103	852868	2135356	0.3028	3.9	Null
B8I4J7	RUMCH Ribosomal RNA small subunit methyltransferase I	960563	179866	1872536	3472229	3337510	1205378	0.3007	2.7	Null
B8I4J8	RUMCH Transcriptional regulator, AbrB family	55891080	84336295	188583436	31896502	8317708	43229938	0.1440	3.9	Null
B8I4K1	RUMCH Uncharacterized protein	0	0	0	98053	0	0	NA	NA	Null
B8I4K2	RUMCH Hydrolase, TatD family	1346047	0	0	0	44517	0	NA	30.2	Null
B8I4K3	RUMCH Uncharacterized protein	3901409	12226946	14383430	5979854	6248447	4266588	0.3990	1.8	Null
B8I4K4	RUMCH 3D domain protein	26788651	23106888	31738070	2483152	4015329	6089991	0.0010	6.5	Decrease
B8I4K5	RUMCH Ribosomal RNA small subunit methyltransferase A	4008215	4894603	5991714	2915657	3359440	2278258	0.1939	1.7	Null
B8I4K6	RUMCH Uncharacterized protein	3163690	6984885	3464216	4726143	378703	1911984	0.5717	1.9	Null
B8I4K7	RUMCH Hemerythrin-like metal-binding protein	40510016	268617934	949256820	141213403	153429800	149037430	0.3979	2.8	Null
B8I4K8	RUMCH Uncharacterized protein	3858854	10960229	4761787	1997232	1506540	0	0.2855	5.6	Null
B8I4K9	RUMCH Metalloenzyme domain protein	0	142660	609168	1293501	3469081	947927	0.1500	7.6	Null
B8I4L0	RUMCH Peptidase U32	196322	696355	198127	1031324	151357	163215	0.8764	1.2	Null
B8I4L1	RUMCH dUTP diphosphatase	7776943	25289242	15114204	25383670	9588957	9462891	0.9940	1.1	Null
B8I4L2	RUMCH GTPase HflX	658626	2634709	2597810	2982165	4541015	3184819	0.2841	1.8	Null
B8I4L3	RUMCH NUDIX hydrolase	835383986	4293698705	3626868552	874831998	883426365	178726273	0.0748	4.5	Null
B8I4L4	RUMCH Uncharacterized protein	445931	1070323	1191685	71115	64284	36384	0.0000	15.8	Decrease
B8I4L5	RUMCH Probable transcriptional regulatory protein Ccel	31443889	39688463	32476472	0	52117	0	0.0000	1988.0	Decrease
B8I4L6	RUMCH Uncharacterized protein	833889	0	223900	0	0	0	0.1039	NA	Null
B8I4L7	RUMCH Uncharacterized protein	2087683882	1014911084	723826367	530730135	971559942	699404584	0.4912	1.7	Null
B8I4L8	RUMCH Uncharacterized protein	0	1715043	421781	303046	605413	496189	0.9190	1.5	Null
B8I4L9	RUMCH Transcriptional regulator, AraC family	443866	177166	0	394174	1913118	873616	0.3111	5.1	Null
B8I4M1	RUMCH Methyltransferase small	0	235881	0	0	0	0	NA	NA	Null
B8I4M6	RUMCH Peptidoglycan-binding domain 1 protein	124484634	434236255	586997167	685673949	624059879	473616126	0.3979	1.6	Null
B8I4M7	RUMCH Alcohol dehydrogenase GroES domain protein	0	0	0	373272	0	0	0.2787	NA	Null
B8I4M8	RUMCH ROK family protein	12870092	2215603	3808366	6537239	2169690	873616	0.4957	2.0	Null
B8I4N2	RUMCH Transcriptional regulator, PadR-like family	0	147743	0	1208467	0	1399047	0.2813	17.6	Null
B8I4N4	RUMCH DUF4157 domain-containing protein	21825488	7926248	8496411	5374166	9918173	5985162	0.4624	1.8	Null
B8I4N6	RUMCH Transcriptional regulator, MerR family	0	405086	648010	943598	865319	740675	0.5284	2.4	Null
B8I4N9	RUMCH Uncharacterized protein	331129128	181357344	96655749	208123770	173281951	201160503	0.9721	1.0	Null
B8I4P0	RUMCH GMP synthase [glutamine-hydrolyzing]	19963843	33218324	44881303	52908065	115879540	25023205	0.2917	2.0	Null
B8I4P2	RUMCH Pyridinium-3,5-bisthiocarboxylic acid mononucleotide nickel inser	0	3507963	0	1382288	1688099	981235	NA	1.2	Null
B8I4P3	RUMCH 1-(5-phosphoribosyl)-5-amino-4-imidazole-carboxylate (AIR) carbo	0	0	0	3652601	3013053	2116095	0.0000	NA	Increase
B8I4P4	RUMCH Peptide chain release factor 2	1216680	792908	2249719	0	2714238	741007	0.9585	1.2	Null
B8I4P5	RUMCH Cof-like hydrolase	6111706	5816009	9034423	5574353	1437165	2133396	0.2424	2.3	Null
B8I4P6	RUMCH Aminotransferase	4828210	16421227	20364727	16066988	12065401	4817550	0.8698	1.3	Null
B8I4P7	RUMCH Transcriptional regulator, AsnC family	0	882160	836506	12498186	11197274	12974366	0.0110	21.3	Increase
B8I4P8	RUMCH Uncharacterized protein	1506685809	2301112031	1905315218	254128603	179062215	374950719	0.0000	7.1	Decrease
B8I4P9	RUMCH Thioredoxin	773850682	1158810430	365492749	293263721	218839567	156797943	0.0356	3.4	Decrease
B8I4Q0	RUMCH Uncharacterized protein	3166641	2287502	5442457	10891128	8042654	20091037	0.0320	3.6	Increase
B8I4Q1	RUMCH NAGPA domain-containing protein	2924704	31540852	3434942	3520239	30135727	347933	0.9988	1.1	Null
B8I4Q3	RUMCH Alpha-glucan phosphorylase	48903566	81339412	51752636	155608990	69925790	50612294	0.4490	1.5	Null
B8I4Q4	RUMCH Uncharacterized protein	219721509	73399497	31394712	34806936	9886152	3086432	0.0541	6.8	Decrease
B8I4Q5	RUMCH Uncharacterized protein	124445	0	436686	0	0	198579	0.8496	2.8	Null
B8I4Q6	RUMCH CoA-substrate-specific enzyme activase	359972	0	0	0	0	803980	NA	2.2	Null
B8I4Q7	RUMCH Uncharacterized protein	0	0	0	0	236153	0	NA	NA	Null
B8I4Q8	RUMCH DUF2229 domain-containing protein	0	0	0	0	148106	213669	0.2622	NA	Null
B8I4Q9	RUMCH MurNAc-LAA domain-containing protein	123361721	220565303	267351762	109837337	141420552	59577388	0.2427	2.0	Null
B8I4R0	RUMCH Fructose-1,6-bisphosphate aldolase, class II	12209507347	25775973546	21330720077	20442909129	14880663490	9084218586	0.6829	1.3	Null
B8I4R1	RUMCH Pyrophosphate--fructose 6-phosphate 1-phosphotransferase	6398797670	5151822753	4123329682	3366741434	1931115509	1300401699	0.1121	2.4	Null
B8I4R2	RUMCH Formate acetyltransferase	232377	321165	365525	436018	554755	147007	0.7930	1.2	Null
B8I4R3	RUMCH Flavin reductase domain protein FMN-binding	25537128	20625146	17520116	7366822	6269414	4450402	0.0024	3.5	Decrease
B8I4R4	RUMCH Endoglucanase	12053252	16852024	25090438	26162985	16530201	8592991	0.9972	1.1	Null
B8I4R5	RUMCH Platelet-activating factor acetylhydrolase plasma/intracellular isof	183451282	317003480	181661402	47055196	60337680	34711923	0.0001	4.8	Decrease
B8I4R6	RUMCH Platelet-activating factor acetylhydrolase plasma/intracellular isof	7988451	8758197	39354884	61266899	67460663	33132200	0.1440	2.9	Null
B8I4S0	RUMCH Hydrogenase, Fe-only	121856	0	0	90666	0	0	NA	1.3	Null
B8I4S4	RUMCH NADH-quinone oxidoreductase, E subunit	8234486	1257572	0	0	0	0	0.0046	NA	Decrease
B8I4S5	RUMCH Methyl-accepting chemotaxis sensory transducer	14373110	108925862	54892615	124113026	116973874	331130171	0.1275	3.2	Null
B8I4S6	RUMCH Uncharacterized protein	235340	4686345	8330193	922716	0	56571	0.1564	13.5	Null
B8I4S7	RUMCH Putative pre-16S rRNA nuclease	298505	2062859	0	0	952473	0	0.8463	2.5	Null
B8I4S8	RUMCH UPF0297 protein Ccel	147100509	377075976	118622295	941562	6804271	11401323	0.0001	33.6	Decrease
B8I4S9	RUMCH RNA modification enzyme, MiaB family	2569609	8378114	14884216	9856335	10360077	11800323	0.6757	1.2	Null
B8I4T1	RUMCH Probable pectate lyase C	933537040	594983677	867754992	679727027	328384575	299865125	0.2891	1.8	Null
B8I4T2	RUMCH HPrNtr domain-containing protein	59878024	1271118457	157347654	84642684	89684521	119400851	0.1926	5.1	Null
B8I4T3	RUMCH DUF523 domain-containing protein	0	519143	1726904	0	300527	0	0.5717	7.5	Null
B8I4T4	RUMCH Ribonuclease R	0	1098839	0	0	65557	1487343	NA	1.4	Null
B8I4T5	RUMCH Uncharacterized protein	3092432	5926676	1587841	4475783	10891916	6812219	0.2744	2.1	Null
B8I4T6	RUMCH Histidine kinase	0	56303	0	87616	68429	89536	0.5193	4.4	Null
B8I4T7	RUMCH Stage 0 sporulation protein A homolog	1664930	0	0	678484	3174911	1209541	0.7340	3.0	Null
B8I4T8	RUMCH Polysaccharide deacetylase	0	200113	157567	57231	60082	331503	0.8463	1.3	Null
B8I4T9	RUMCH Metal dependent phosphohydrolase	4251843	5651315	509041	1387532	340652	557927	0.1475	4.6	Null
B8I4U0	RUMCH Protein-export membrane protein SecG	811972	557585	803583	1580940	2772059	911599	0.1670	2.4	Null
B8I4U1	RUMCH Enolase	79553572	53161028	43639658	151806115	128199712	90561977	0.1631	2.1	Null
B8I4U2	RUMCH Transcriptional regulator, XRE family	4278769	31376543	14900881	4090570	4086473	4802263	0.1044	3.9	Null
B8I4U3	RUMCH Uncharacterized protein	2150339	3974472	789607	690303	449999	570706	0.0749	4.0	Null
B8I4U4	RUMCH 2,3-bisphosphoglycerate-independent phosphoglycerate mutase	59682611	74979990	217948031	85389276	191698163	97803935	0.8648	1.1	Null
B8I4U5	RUMCH Multifunctional fusion protein	4312097615	7772643937	5953259205	8569156797	12101107087	7252699521	0.2199	1.5	Null
B8I4U7	RUMCH Pentapeptide repeat protein	0	91089	0	433002	455242	447047	0.0994	14.7	Null
B8I4U8	RUMCH Uncharacterized protein	134229	258770	0	0	962007	247527	0.6587	3.1	Null
B8I4U9	RUMCH Biotin	1928489	2234016	1408103	305753	1115874	367637	0.1580	3.1	Null
B8I4V0	RUMCH ABC-type bacteriocin transporter	0	1077538	93369	0	0	0	0.1268	NA	Null
B8I4V2	RUMCH Signal transduction histidine kinase regulating citrate/malate met	443776	0	0	0	0	0	0.2439	NA	Null
B8I4V3	RUMCH Stage 0 sporulation protein A homolog	0	112239	0	852381	472797	687444	0.0704	17.9	Null
B8I4V4	RUMCH Protein translocase subunit SecA	2886333	477045	2390520	4129624	996068	1053680	0.9843	1.1	Null
B8I4V5	RUMCH Galactose-1-phosphate uridylyltransferase	3879598	35363360	32941810	14062572	487656	0	NA	5.0	Null
B8I4V6	RUMCH Trans-2-enoyl-CoA reductase [NADH]	114640734	168508556	100219990	46484544	67152771	133913556	0.7077	1.5	Null
B8I4V7	RUMCH Uncharacterized protein	170541305	100815440	47485081	414791	11193119	2408012	0.0027	22.7	Decrease
B8I4V8	RUMCH Glyceraldehyde-3-phosphate dehydrogenase	1445653926	4052914273	3857126472	6589709937	6016815132	4143487521	0.1813	1.8	Null
B8I4V9	RUMCH DUF2156 domain-containing protein	0	0	0	0	173672	234204	0.2517	NA	Null
B8I4W0	RUMCH Peptidase T-like protein	2977196	4746314	5274341	11316521	7345873	1481349	0.6006	1.5	Null
B8I4W1	RUMCH Putative tRNA (cytidine(34)-2'-O)-methyltransferase	4344452	1052781	1317205	1374011	1618582	748352	0.5122	1.8	Null
B8I4W2	RUMCH CheC domain protein	1030087	0	0	1489891	400434	0	0.9283	1.8	Null
B8I4W7	RUMCH RNA polymerase sigma factor	0	118595	0	333044	445863	0	0.5567	6.6	Null
B8I4W8	RUMCH Anti-sigma F factor	37409503	133079615	61344686	120114509	62378690	94005309	0.7087	1.2	Null
B8I4W9	RUMCH Anti-sigma F factor antagonist	2299132	2426893	1236835	591154	0	0	0.2566	10.1	Null
B8I4X0	RUMCH Probable cell division protein WhiA	636876	2130102	2962608	619536	490744	1143589	0.3391	2.5	Null
B8I4X1	RUMCH Glycogen debranching enzyme	340064	425535	427033	840885	1205490	928806	0.0366	2.5	Increase
B8I4X2	RUMCH Putative gluconeogenesis factor	159032	689445	0	533302	790615	0	0.8764	1.6	Null
B8I4X3	RUMCH Nucleotide-binding protein Ccel	0	2338642	0	2846642	4337232	2662903	0.5240	4.2	Null
B8I4X4	RUMCH UDP-N-acetylenolpyruvoylglucosamine reductase	1398598	1891520	1281791	483272	2139062	1007767	0.8804	1.3	Null
B8I4X5	RUMCH PHP domain protein	0	0	0	0	2233359	159314	0.0393	NA	Increase
B8I4X6	RUMCH HPr kinase/phosphorylase	1227150	475075	0	2731467	2266015	3836314	0.3045	5.2	Null
B8I4X7	RUMCH K(+)-insensitive pyrophosphate-energized proton pump	0	1246540	386136	1051201	932570	1800015	0.5610	2.3	Null
B8I4X8	RUMCH Polynucleotide adenylyltransferase/metal dependent phosphohyd	0	0	0	1498168	435689	848294	0.0000	NA	Increase
B8I4Y1	RUMCH Transposase IS605 OrfB	1718758	1057104	0	1130433	15040091	7102879	0.2310	8.4	Null
B8I4Y2	RUMCH Protein serine/threonine phosphatase	131313	0	188103	2400836	1201486	292754	0.0765	12.2	Null
B8I4Y3	RUMCH Putative PAS/PAC sensor protein	4550537	1245812	19468184	5680402	4978549	2081371	0.5945	2.0	Null
B8I4Y4	RUMCH Uncharacterized protein	9301153	12024119	10097310	9606929	9759255	1095342	0.6742	1.5	Null
B8I4Y6	RUMCH Hydrogenase, Fe-only	283536981	323267632	165351820	325643856	336109009	163899341	0.8997	1.1	Null
B8I4Y7	RUMCH Respiratory-chain NADH dehydrogenase domain 51 kDa subunit	406468843	838017364	278229496	490770942	540706234	385761333	0.9992	1.1	Null

B8I4Y8	RUMCH NADH dehydrogenase (Ubiquinone) 24 kDa subunit	126709003	1543840150	102025661	19191033	136346911	14510026	0.0749	10.4	Null
B8I4Y9	RUMCH Aldo/keto reductase	6012798	60574515	35801863	8123622	12550898	9069416	0.2000	3.4	Null
B8I4Z2	RUMCH Arsenate reductase and related	1430598	1111752	246265	371201	544282	70970	0.3201	2.8	Null
B8I4Z3	RUMCH CheB methylesterase	0	254877	667421	0	0	0	0.1455	NA	Null
B8I4Z5	RUMCH Methyl-accepting chemotaxis sensory transducer	581615	482729	592903	465692	359681	679406	0.9952	1.1	Null
B8I4Z8	RUMCH Appr-1-p processing domain protein	0	0	0	641312	1332958	1801217	0.0000	NA	Increase
B8I500	RUMCH VWFA domain-containing protein	498929	634157	715948	498987	362440	354531	0.5222	1.5	Null
B8I502	RUMCH ABC transporter related	0	4130363	25090386	7118438	5172879	6458269	0.9089	1.6	Null
B8I504	RUMCH Uncharacterized protein	0	70444	1101942	1188984	0	0	NA	1.0	Null
B8I507	RUMCH Extracellular solute-binding protein family 1	4413047	3386278	2013078	7531508	6231090	10203980	0.1393	2.4	Null
B8I510	RUMCH Glycoside hydrolase family 3 domain protein	31395523	15308621	32368110	10044221	19455800	15461061	0.4520	1.8	Null
B8I512	RUMCH tRNA synthetase class I (M)	10841210	70177097	53904761	95119392	175362862	133402867	0.0924	3.0	Null
B8I516	RUMCH Xanthine/uracil/vitamin C permease	0	0	0	0	181302	0	NA	NA	Null
B8I517	RUMCH Acetyl xylan esterase	4846133	3511744	4883841	4877376	4918360	1696588	0.8713	1.2	Null
B8I518	RUMCH ATPase	0	473728	0	0	2017003	633767	0.5707	5.6	Null
B8I519	RUMCH Phosphoenolpyruvate carboxykinase [GTP]	139204883	271951183	62208353	228384854	153107309	22284176	0.9056	1.2	Null
B8I520	RUMCH ThuA domain-containing protein	364139	0	0	0	499235	353920	0.8486	2.3	Null
B8I521	RUMCH Uronate isomerase	138051	91051	315922	72946	325445	259118	0.8427	1.2	Null
B8I526	RUMCH Methyl-accepting chemotaxis sensory transducer	0	0	0	253984	139275	0	0.2688	NA	Null
B8I528	RUMCH Methyl-accepting chemotaxis sensory transducer	0	0	0	2374017	673873	0	0.0229	NA	Increase
B8I529	RUMCH Transcriptional regulator, GntR family	427744	819567	905732	379746	26334	307231	0.3465	3.0	Null
B8I530	RUMCH ABC transporter related	359222	291960	870117	815235	3644225	769875	0.1615	3.4	Null
B8I531	RUMCH Uncharacterized protein	0	0	0	87158	0	19982	NA	NA	Null
B8I532	RUMCH Uncharacterized protein	7611112	5704495	317953	440771	452112	1766531	0.2244	5.1	Null
B8I533	RUMCH Uncharacterized protein	0	265701	0	148460	952806	623630	0.3425	6.5	Null
B8I534	RUMCH DUF4367 domain-containing protein	0	444998	804005	2958067	2385917	315060	0.3084	4.5	Null
B8I535	RUMCH RNA polymerase, sigma-24 subunit, ECF subfamily	0	90549	3114653	75351	47653	0	NA	26.1	Null
B8I536	RUMCH DUF4352 domain-containing protein	459528	2088087	1388676	1233299	2703740	5925396	0.2370	2.5	Null
B8I537	RUMCH NUDIX hydrolase	7916706	1818459	4346466	81269	2954709	156095	0.2855	4.4	Null
B8I540	RUMCH C	521475	744115	0	720371	2807618	0	0.7225	2.8	Null
B8I541	RUMCH Cadmium-translocating P-type ATPase	491496	255840	0	892595	774866	506688	0.5527	2.9	Null
B8I542	RUMCH Heavy metal transport/detoxification protein	338095	0	0	0	0	130366	0.8481	2.6	Null
B8I543	RUMCH Transcriptional regulator, ArsR family	0	2007702	0	0	0	1418044	NA	1.4	Null
B8I544	RUMCH Peptidase U57 YabG	707898	1389387	305061	4392442	2714501	2112897	0.0432	3.8	Increase
B8I546	RUMCH Uncharacterized protein	1145425	1478198	0	440525	564175	924261	0.9178	1.4	Null
B8I547	RUMCH Uncharacterized protein	0	0	0	0	256115	0	0.3023	NA	Null
B8I549	RUMCH DNA polymerase III subunit gamma/tau	16327573	19894007	4091872	5061545	8600406	3711616	0.3084	2.3	Null
B8I550	RUMCH Nucleoid-associated protein Ccel	255138987	205979001	611936475	167482081	13909812	21900214	0.0999	5.3	Null
B8I551	RUMCH Recombination protein RecR	4933952	25523	9746342	2542680	2065317	0	0.6078	3.2	Null
B8I553	RUMCH Stage 0 sporulation protein A homolog	0	0	286201	910489	1671135	1278771	0.1557	13.5	Null
B8I555	RUMCH Pyrophosphate phospho-hydrolase	5391702	1096537	11521454	13893482	16254077	3720511	0.5573	1.9	Null
B8I559	RUMCH Uncharacterized protein	1057043	1277332	829867	241382	1126751	849712	0.8089	1.4	Null
B8I560	RUMCH Uncharacterized protein	157289785	195737110	685968916	159105051	361542407	429653736	0.9620	1.1	Null
B8I561	RUMCH DUF4097 domain-containing protein	45909402856	69657067763	40270971937	31287817987	21129775958	17474835405	0.0722	2.2	Null
B8I562	RUMCH Peptide chain release factor 1	61913115	46235534	47962739	26928605	9302732	12860048	0.0399	3.2	Decrease
B8I564	RUMCH Threonylcarbamoyl-AMP synthase	2574033	3332789	5695808	2426195	4993103	2547797	0.9416	1.2	Null
B8I565	RUMCH Protein tyrosine phosphatase	3416153	1616855	2244317	399471	471441	546075	0.0068	5.1	Decrease
B8I566	RUMCH Sugar-phosphate isomerase, RpiB/LacA/LacB family	393479	16198400	3361644	1158695	1642355	1068848	0.2063	5.2	Null
B8I567	RUMCH Uracil phosphoribosyltransferase	154548515	281983440	129352199	3530052	1741929	185898130	0.6297	3.0	Null
B8I569	RUMCH UDP-N-acetylglucosamine 2-epimerase	904502328	1775764997	656609306	128930036	163635059	244603521	0.0025	6.2	Decrease
B8I570	RUMCH OmpA/MotB domain protein	1050721	997824	1124087	2583992	3458570	502669	0.3691	2.1	Null
B8I571	RUMCH MotA/TolQ/ExbB proton channel	0	149349	0	399792	0	125418	0.7211	3.5	Null
B8I574	RUMCH ATP synthase subunit c	1000805	1497560	0	2358994	2415423	0	0.8463	1.9	Null
B8I575	RUMCH ATP synthase subunit b	99533651	124401903	37596713	131929	0	43205	0.0000	1493.3	Decrease
B8I576	RUMCH ATP synthase subunit delta	57819513	12498425	37365447	139999494	21891707	20977817	0.7134	1.7	Null
B8I577	RUMCH ATP synthase subunit alpha	690828787	610911149	710208053	801323147	799284061	663436191	0.6935	1.1	Null
B8I578	RUMCH ATP synthase gamma chain	138663476	38125948	80840612	181089196	207290867	211554566	0.2480	2.3	Null
B8I579	RUMCH ATP synthase subunit beta	947290401	1067252301	1088903123	1223217383	1842196970	958679704	0.4881	1.3	Null
B8I580	RUMCH ATP synthase epsilon chain	39452433	170008455	175420073	70779716	58170459	102525803	0.6689	1.7	Null
B8I581	RUMCH S-layer domain protein	1179492	1298349	3117818	5414219	9827394	3704016	0.0354	3.4	Increase
B8I582	RUMCH S-layer domain protein	23987897	20041679	18099781	42756845	31905271	20480296	0.3585	1.5	Null
B8I583	RUMCH Fibronectin, type III	122999367	183658262	65299940	178428454	127171707	170878633	0.6517	1.3	Null
B8I584	RUMCH Fibronectin type III domain protein	242910491	141385442	434198369	81875027	23098800	37676073	0.0084	5.7	Decrease
B8I585	RUMCH S-layer domain protein	0	2328454	0	1150527	647433	1019919	NA	1.2	Null
B8I586	RUMCH Uncharacterized protein	202670	296842	248489	299997	497554	0	0.9711	1.1	Null
B8I587	RUMCH UDP-N-acetylglucosamine 1-carboxyvinyltransferase	0	0	0	151345	0	0	NA	NA	Null
B8I589	RUMCH Peptidase M23	71725839	129539678	295562386	69881803	70864825	65994138	0.2316	2.4	Null
B8I590	RUMCH D-alanine--D-alanine ligase	50757159	48203756	40388941	147130518	130176276	193783646	0.0027	3.4	Increase
B8I591	RUMCH Uncharacterized protein	21881433	24056132	75341486	33299446	24937609	11396785	0.5295	1.7	Null
B8I592	RUMCH Arginine--tRNA ligase	33930071	5896706	17344372	19589690	41251349	21763930	0.7464	1.4	Null
B8I593	RUMCH Cellulase	71246274227	69063778393	73360842378	35172678218	26753416432	27588158201	0.0094	2.4	Decrease
B8I594	RUMCH Methyl-accepting chemotaxis sensory transducer	231585882	179123269	79824541	180881626	290235272	190849674	0.6845	1.3	Null
B8I595	RUMCH Uncharacterized protein	867846801	673785245	604664992	1273708815	320305569	228702380	0.8565	1.2	Null
B8I596	RUMCH S-layer domain protein	158749228	966175341	360809157	291764632	461737380	453969831	0.9584	1.2	Null
B8I597	RUMCH Carboxyl-terminal protease	41147274	90774837	75056309	58690556	59170557	85449633	0.8782	1.0	Null
B8I598	RUMCH CTP synthase	14411409	32631816	43109229	65669262	87646405	46545527	0.0875	2.2	Null
B8I599	RUMCH Stage II sporulation protein R	6139337	23655288	22524630	19883149	15072857	19535519	0.8438	1.0	Null
B8I5A1	RUMCH ATPase, P-type (Transporting), HAD superfamily, subfamily IC	363608	727952	151080	360933	1009627	346510	0.7372	1.4	Null
B8I5A2	RUMCH ABC transporter related	81128893	582426651	396741529	578315278	754151809	693864587	0.2972	1.9	Null
B8I5A4	RUMCH ABC	568367189	663971482	260026714	419524230	713003639	514259492	0.8516	1.1	Null
B8I5A5	RUMCH Uncharacterized protein	1163696654	2438862427	1270053934	1445070598	2852875924	1736779734	0.6338	1.2	Null
B8I5A7	RUMCH Uncharacterized protein	850599	3142696	2895276	5592234	3685387	1671266	0.4761	1.6	Null
B8I5A9	RUMCH Dihydroxy-acid dehydratase	2274625596	2380531724	1817965370	1234324518	803316420	685755263	0.0352	2.4	Decrease
B8I5B0	RUMCH Acetolactate synthase	36424904	48747051	143754585	219510878	131111051	125971093	0.2320	2.1	Null
B8I5B1	RUMCH DEAD/DEAH box helicase domain protein	3528255	6252944	9714667	4428619	12751024	3813257	0.8668	1.1	Null
B8I5B2	RUMCH 50S ribosomal protein L33	114124	85127066	0	0	0	4330097	NA	19.7	Null
B8I5B3	RUMCH Protein translocase subunit SecE	26949227	21290470	14857930	12936645	432447	158214	0.2632	4.7	Null
B8I5B4	RUMCH Transcription termination/antitermination protein NusG	62203506	173505510	185199307	351438004	473897735	332473414	0.0214	2.8	Increase
B8I5B5	RUMCH 50S ribosomal protein L11	522628399	953419672	1035015338	276754531	135362980	128014337	0.0004	4.6	Decrease
B8I5B7	RUMCH Type IV pilus assembly PilZ	0	1085194	11436802	5595369	6119486	1449696	0.9560	1.1	Null
B8I5B9	RUMCH Beta-xylanase	7847385	2226763	9818764	1354878	997252	7801158	0.6848	2.0	Null
B8I5C0	RUMCH Beta-xylanase	162518273	146336808	142143366	349996572	141346173	37475868	0.9015	1.2	Null
B8I5C6	RUMCH Phosphopantetheine-binding	0	0	0	257506	68517	74665	0.0627	NA	Null
B8I5C9	RUMCH Amino acid adenylation domain protein	1537355	72911311	15805482	3792437	12601402	6433743	0.3162	4.0	Null
B8I5D1	RUMCH Amino acid adenylation domain protein	1058848	5121736	3722662	15031833	20803066	1442599	0.1722	3.8	Null
B8I5D4	RUMCH Copper amine oxidase domain protein	0	0	69986	0	463075	0	0.6347	6.6	Null
B8I5D5	RUMCH Copper amine oxidase domain protein	2710225	2636717	3676796	1802520	1426186	431202	0.1808	2.5	Null
B8I5D6	RUMCH Uncharacterized protein	532846	1018608	0	76072	281295	232062	0.6655	2.6	Null
B8I5D7	RUMCH Cellulase	350237798	212538728	308615039	536270423	644471021	516905766	0.1491	1.9	Null
B8I5E0	RUMCH Uncharacterized protein	0	0	0	0	59930	0	NA	NA	Null
B8I5E2	RUMCH ABC transporter related	1015422	572469	2149303	1670344	1038643	647605	0.9515	1.1	Null
B8I5E4	RUMCH FMN-dependent alpha-hydroxy acid dehydrogenase	0	472809	724398	1357246	1252479	676655	0.4666	2.7	Null
B8I5E5	RUMCH Uncharacterized protein	0	0	0	1100077	1674610	0	0.0231	NA	Increase
B8I5E8	RUMCH ABC transporter related	0	1477726	0	86048	1040369	957822	NA	1.4	Null
B8I5E9	RUMCH Radical SAM domain protein	6061449	3151090	2118743	1919467	5390502	2823205	0.9302	1.1	Null
B8I5F0	RUMCH Radical SAM domain protein	3544883	5156182	4451128	4523270	3443274	2110766	0.6587	1.3	Null
B8I5F1	RUMCH Radical SAM domain protein	35133640	106641779	97200321	62817746	41486797	115519359	0.9463	1.1	Null
B8I5F2	RUMCH Glycosyltransferase 36	0	423985	0	0	161127	92800	0.9470	1.7	Null
B8I5F6	RUMCH Ig domain protein group 2 domain protein	232968321	475206968	182807709	208174096	168966782	160768038	0.4446	1.7	Null
B8I5G1	RUMCH S-layer domain protein	96646902	59942259	45970739	114575061	200045520	62564781	0.3891	1.9	Null
B8I5G2	RUMCH Zn	0	819332	866745	313528	429693	659468	0.9988	1.2	Null
B8I5G3	RUMCH Uncharacterized protein	0	774918	1460263	467197	423450	629304	0.9265	1.5	Null

B8I5G4	RUMCH Uncharacterized protein	0	0	0	0	747038	2468915	0.0170	NA	Increase
B8I5G6	RUMCH Uncharacterized protein	6777660	8769908	8057397	5467216	4035139	3766891	0.1070	1.8	Null
B8I5G7	RUMCH Radical SAM domain protein	2536403	2337974	950322	194983	5090626	2148699	0.8486	1.3	Null
B8I5H3	RUMCH Beta-ketoacyl synthase	11145115	151315	11895453	2890140	466332	0	0.3007	6.9	Null
B8I5H4	RUMCH Beta-ketoacyl synthase	0	0	1432714	139071	825766	58481	NA	1.4	Null
B8I5H5	RUMCH Amino acid adenylation domain protein	8668202	0	1002935	44332540	0	838980	NA	4.7	Null
B8I5H7	RUMCH [Acyl-carrier-protein] S-malonyltransferase	0	4247861	0	2317808	1006702	879947	NA	1.0	Null
B8I5H8	RUMCH Amino acid adenylation domain protein	2394291	1120310	6860531	10602004	5340911	6314361	0.3051	2.1	Null
B8I5H9	RUMCH Uncharacterized protein	0	193792	0	0	243054	1214651	0.4641	7.5	Null
B8I5I0	RUMCH Condensation domain protein	1247279	0	0	713025	604145	122187	NA	1.2	Null
B8I5I2	RUMCH Beta-ketoacyl synthase	3499865	0	0	0	0	0	NA	NA	Null
B8I5I3	RUMCH Amino acid adenylation domain protein	560108	0	0	6314	127122	0	0.7090	4.2	Null
B8I5I5	RUMCH Pyruvate phosphate dikinase PEP/pyruvate-binding	0	0	0	251828	865643	0	0.0960	NA	Null
B8I5I6	RUMCH Cyclic peptide transporter	0	0	0	0	427491	491376	0.1050	NA	Null
B8I5I7	RUMCH Amino acid adenylation domain protein	1390856	0	1983802	0	194176	412356	0.5314	5.6	Null
B8I5I8	RUMCH Uncharacterized protein	0	0	1193897	1844009	996051	631358	0.6546	2.9	Null
B8I5I9	RUMCH Endoglucanase	1495076047	2356522550	2416668796	1841145840	1405404070	1221248957	0.4405	1.4	Null
B8I5J0	RUMCH S-layer domain protein	594354304	1241078899	845299572	1773837924	1892472965	1718323306	0.0342	2.0	Increase
B8I5J1	RUMCH Major facilitator superfamily MFS	669271	492745	0	0	152760	570093	0.9095	1.6	Null
B8I5J2	RUMCH Cellulase	619206717	1004348467	863390028	411099512	357224729	97104097	0.0915	2.9	Null
B8I5J3	RUMCH SMI1 / KNR4 family	15773103	8535134	12468988	66429153	83747887	71527671	0.0000	6.0	Increase
B8I5J4	RUMCH Tox-SHH domain-containing protein	50838905	28846567	36277491	44699805	80855890	19668592	0.8098	1.3	Null
B8I5J5	RUMCH Vitamin B12-dependent ribonucleotide reductase	2665321	1454056	2310426	2908614	3162426	1924189	0.7310	1.2	Null
B8I5J6	RUMCH DUF4015 domain-containing protein	136322426	246790237	132170712	151592318	166738949	206193920	0.8636	1.0	Null
B8I5J7	RUMCH PA14 domain protein	81483824	54687022	69234341	117579860	83781432	16935807	0.9711	1.1	Null
B8I5J8	RUMCH Uncharacterized protein	161103	662644	0	1223665	733993	353324	0.5337	2.8	Null
B8I5J9	RUMCH Uncharacterized protein	7523524	5418385	2878921	709207	4668987	5406291	0.8132	1.5	Null
B8I5K0	RUMCH Uncharacterized protein	40311665	139836665	167858979	37326494	81313455	30170981	0.3007	2.3	Null
B8I5K1	RUMCH Fimbrial assembly family protein	15585785	25703219	186579872	6424721	20137413	5464590	NA	7.1	Null
B8I5K2	RUMCH Uncharacterized protein	216273	0	524337	377817	261897	238636	0.9213	1.2	Null
B8I5K3	RUMCH Type IV pilus assembly protein PilM	0	1753982	6778089	3864303	3688143	3151816	0.8698	1.3	Null
B8I5K5	RUMCH Uncharacterized protein	0	292666	0	0	0	0	0.3129	NA	Null
B8I5K6	RUMCH Type II secretion system protein	0	0	0	127185	0	0	NA	NA	Null
B8I5K7	RUMCH Twitching motility protein	1983555	0	1603507	1146743	1034912	382770	0.8688	1.4	Null
B8I5K8	RUMCH Type II secretion system protein E	2988673	6189500	4460064	3890379	1237009	1026055	0.2744	2.2	Null
B8I5K9	RUMCH Uracil-DNA glycosylase superfamily	0	0	2648392	960152	0	0	NA	2.8	Null
B8I5L0	RUMCH Type-4 uracil-DNA glycosylase	649523	3761486	347037	116613	98491	44680	0.0033	18.3	Decrease
B8I5L1	RUMCH Dolichyl-phosphate-mannose--protein mannosyltransferase	646652	9706268	1318909	4674737	13267614	2493212	0.6026	1.8	Null
B8I5L3	RUMCH Adenylosuccinate synthetase	1684691376	538874219	483850948	497005886	1127433852	166423843	0.6845	1.5	Null
B8I5L5	RUMCH Hydro-lyase, Fe-S type, tartrate/fumarate subfamily, beta subunit	7954237	10634203	11040885	2292777	2336836	1981683	0.0000	4.5	Decrease
B8I5L6	RUMCH Hydro-lyase, Fe-S type, tartrate/fumarate subfamily, alpha subuni	12372691	19490779	13103231	7817864	3218489	3336201	0.0247	3.1	Decrease
B8I5L7	RUMCH UspA domain protein	39378837	18503231	14081967	7499451	3843573	1072031	0.0242	5.8	Decrease
B8I5L8	RUMCH Histone family protein DNA-binding protein	42808640175	15897537355	18462175469	8199342735	10282758064	11527741119	0.2025	2.6	Null
B8I5L9	RUMCH Stage 0 sporulation protein A homolog	15668533	9202288	8029239	21301542	15183359	11141348	0.5811	1.4	Null
B8I5M0	RUMCH Shikimate kinase	0	2135592	699712	211388	801326	59374	0.6992	2.6	Null
B8I5M1	RUMCH Chorismate synthase	117618456	336941394	616024410	24109831	39396172	52905878	0.0018	9.2	Decrease
B8I5M2	RUMCH 3-phosphoshikimate 1-carboxyvinyltransferase	15510781	10992335	23105095	11835794	8609972	6370927	0.2688	1.8	Null
B8I5M3	RUMCH Putative RNA methylase	3017625	5391026	1357631	805983	2574173	6485690	0.9089	1.0	Null
B8I5M4	RUMCH Glutamate dehydrogenase	486209633	491473191	511703118	320683998	259807516	307087864	0.2334	1.7	Null
B8I5M5	RUMCH Pseudouridine synthase	501051	1609436	1159996	422547	522057	304096	0.1759	2.6	Null
B8I5M6	RUMCH Methyltrans	717476	572352	2179721	601035	994517	1821225	0.9192	1.0	Null
B8I5M7	RUMCH Uncharacterized protein	4723995	5887726	12579229	17954195	23718192	5404196	0.2951	2.0	Null
B8I5M8	RUMCH Agmatinase	411496	485925	1323357	1154686	443412	434330	0.9759	1.1	Null
B8I5M9	RUMCH 50S ribosomal protein L1	3573759975	4104860760	2024528404	1158281709	683916671	367126244	0.0070	4.4	Decrease
B8I5N0	RUMCH 50S ribosomal protein L10	11158439506	3538871472	4804217168	1369003706	779227746	856953662	0.0030	6.5	Decrease
B8I5N1	RUMCH 50S ribosomal protein L7/L12	14681107082	11044862962	17643088337	29156521237	32822082877	16195195931	0.2048	1.8	Null
B8I5N2	RUMCH DNA-directed RNA polymerase subunit beta	705429985	1251749810	604055166	1162578023	1433719569	603207482	0.6676	1.2	Null
B8I5N3	RUMCH DNA-directed RNA polymerase subunit beta'	2888244298	5525562842	2926137831	4090472702	1683662504	2681335581	0.7540	1.3	Null
B8I5N4	RUMCH Ribosomal protein L7Ae/L30e/S12e/Gadd45	4596030	422764	0	0	95503	0	0.1500	52.6	Null
B8I5N5	RUMCH 30S ribosomal protein S12	682837053	447728140	452167110	111724609	115977646	102440984	0.0003	4.8	Decrease
B8I5N6	RUMCH 30S ribosomal protein S7	1348782802	1143070524	1400208190	444041456	398202560	268237721	0.0002	3.5	Decrease
B8I5N7	RUMCH Elongation factor G	2002059194	1836732709	2346357793	1169810902	2499542482	680060814	0.6746	1.4	Null
B8I5N8	RUMCH Elongation factor Tu	85155909269	72072240188	73552399292	84683743404	43014819681	28362440639	0.5295	1.5	Null
B8I5N9	RUMCH Uncharacterized protein	1486230	688272	660790	1908797	1245063	3032334	0.3007	2.2	Null
B8I5P0	RUMCH YdaE	13825144	63010269	26521371	9533357	2794795	10702310	0.0904	4.5	Null
B8I5P1	RUMCH Beta-lactamase domain protein	23381787	81084431	109847583	175055026	59822628	39066383	0.7602	1.3	Null
B8I5P2	RUMCH NifU-like domain-containing protein	52134820	66766021	24070881	3264221	18337410	19176279	0.2129	3.5	Null
B8I5P3	RUMCH Uncharacterized protein	38023128	155096988	235272166	436456978	345755613	417546402	0.0915	2.8	Null
B8I5P5	RUMCH UvrB/UvrC protein	7160630	7345769	10875482	2458320	1998327	2108074	0.0001	3.9	Decrease
B8I5P6	RUMCH Protein-arginine kinase	9810165	13443511	20012099	20505368	19107460	7261177	0.8611	1.1	Null
B8I5P7	RUMCH ATPase AAA-2 domain protein	1782638495	1962000174	3554376275	1237678699	735359597	740007646	0.0356	2.7	Decrease
B8I5P8	RUMCH Radical SAM domain protein	692410	1656418	5023020	3832912	2508530	3256499	0.6901	1.3	Null
B8I5Q5	RUMCH DNA repair protein RadA	2621489	1842082	15663342	31930608	4553198	2565538	0.6161	1.9	Null
B8I5Q6	RUMCH DNA integrity scanning protein DisA	9231174	13840638	7601627	12197203	16010767	5781293	0.8484	1.1	Null
B8I5Q8	RUMCH Uncharacterized protein	2311187	41171139	10603573	49807065	36087851	28565901	0.3844	2.1	Null
B8I5Q9	RUMCH Transcriptional regulator, CarD family	7586702	8327506	12746824	6402338	6471909	6462322	0.4772	1.5	Null
B8I5R0	RUMCH PilT protein domain protein	153080	1949708	3508415	3153962	1868404	1694929	0.8262	1.2	Null
B8I5R1	RUMCH 2-C-methyl-D-erythritol 4-phosphate cytidylyltransferase	23774183	24726600	21117953	34185807	26317931	25054990	0.5296	1.2	Null
B8I5R2	RUMCH ABC transporter related	0	446364	0	1628833	1120557	1607095	0.2369	9.8	Null
B8I5R3	RUMCH 2-C-methyl-D-erythritol 2,4-cyclodiphosphate synthase	0	664445	93117	0	0	0	0.1977	NA	Null
B8I5R4	RUMCH Proline--tRNA ligase	1553142	10568409	326046	7893395	2307316	1523825	0.9993	1.1	Null
B8I5R5	RUMCH SCP-like extracellular	4736985	14960333	12474042	3414087	4422429	2517441	0.0476	3.1	Decrease
B8I5R6	RUMCH Pseudouridine synthase	0	0	0	57633816	2374081	580096	NA	NA	Null
B8I5R7	RUMCH Nucleotidyl transferase	582321	503575	588870	1610195	2178861	261211	0.3084	2.4	Null
B8I5S1	RUMCH UV-endonuclease UvdE	0	358557	0	0	0	0	0.2917	NA	Null
B8I5S2	RUMCH RNA binding S1 domain protein	48227043	76605612	49602080	78250639	1063620454	71415569	NA	7.0	Null
B8I5S5	RUMCH Uncharacterized protein	477923	0	0	106048	76057	0	0.7948	2.6	Null
B8I5S6	RUMCH Uncharacterized protein	46688354	73141120	69157602	13831157	9889307	12247242	0.0000	5.3	Decrease
B8I5S8	RUMCH FAD-dependent pyridine nucleotide-disulphide oxidoreductase	14560808	29601551	19367491	25819044	52929349	26165019	0.2855	1.7	Null
B8I5T0	RUMCH Cytochrome b5	204713189	273422173	351887811	951529653	448509839	538993433	0.0329	2.3	Increase
B8I5T5	RUMCH UDP-glucose 4-epimerase	8460632	5299189	6489535	11176150	17755810	6776971	0.3317	1.8	Null
B8I5T6	RUMCH NTP	8914999	33409560	14000756	38246653	29622843	40706480	0.2279	1.9	Null
B8I5T7	RUMCH zf-trcl domain-containing protein	0	60451696	37705924	0	13073165	13104242	0.7396	3.7	Null
B8I5T8	RUMCH Ribosome hibernation promoting factor	3069523363	779268706	3072475611	402534955	6009680581	5994950272	0.6048	1.8	Null
B8I5T9	RUMCH Sulfatase	4127821	3112418	19616058	2782573	2843939	9280186	0.7432	1.8	Null
B8I5U0	RUMCH Shikimate kinase	4969607	3009555	5832826	17897785	25673929	6681998	0.0371	3.6	Increase
B8I5U1	RUMCH ATP-dependent DNA helicase	19778302	7819500	51058173	28422480	25579129	10193872	0.8825	1.2	Null
B8I5U2	RUMCH Beta-glucosidase	0	0	0	1034647	1869597	822972	0.0000	NA	Increase
B8I5U4	RUMCH Uncharacterized protein	314154	0	1243198	686683	679679	861957	0.8285	1.4	Null
B8I5U6	RUMCH Uncharacterized protein	385706016	369164977	480257352	187129434	152634818	94055009	0.0046	2.8	Decrease
B8I5U7	RUMCH Cellulase	376862432	209674777	163696985	541135964	499552048	445888920	0.2633	2.0	Null
B8I5U8	RUMCH ATP phosphoribosyltransferase regulatory subunit	591299	2238011	144068526	5630416	6397096	4532675	NA	8.9	Null
B8I5U9	RUMCH ATP phosphoribosyltransferase	1947302	0	536766	1729929	5602785	461103	0.6078	3.1	Null
B8I5V0	RUMCH Histidinol dehydrogenase	7151592	20155074	11324366	12374374	10269095	14177006	0.9302	1.0	Null
B8I5V1	RUMCH Histidinol-phosphate aminotransferase	6658989	785460	1036375	1581315	5055934	1066374	NA	1.1	Null
B8I5V2	RUMCH Imidazoleglycerol-phosphate dehydratase	0	0	0	0	0	1137434	NA	NA	Null
B8I5V3	RUMCH Phosphoribosylaminoimidazole-succinocarboxamide synthase	2540780	51339836	31625741	38416304	29993040	30050290	0.8261	1.2	Null
B8I5V4	RUMCH Imidazole glycerol phosphate synthase subunit HisH	0	840571	2134707	2325012	2948155	2223807	0.5353	2.5	Null
B8I5V5	RUMCH 1-(5-phosphoribosyl)-5-[(5-phosphoribosylamino)methylideneami	17315961	14341329	5519054	8852918	3423924	5648954	0.3357	2.1	Null
B8I5V6	RUMCH Imidazole glycerol phosphate synthase subunit HisF	44329665	44850872	91195921	41805222	45407940	43264559	0.6831	1.4	Null
B8I5V7	RUMCH Histidine biosynthesis bifunctional protein HisIE	12391129	25712898	38049576	14169409	14065362	17363454	0.5193	1.7	Null
B8I5V8	RUMCH Uncharacterized protein	169104	262787	0	0	0	0	0.2632	NA	Null

B8I5V9	RUMCH 10 kDa chaperonin	5387226693	4575261205	8252726977	1283715290	1193120803	2634904924	0.0573	3.6	Null
B8I5W0	RUMCH 60 kDa chaperonin	156275719899	209697204925	150222733001	196629039229	191504965060	205426190812	0.6249	1.1	Null
B8I5W1	RUMCH Uncharacterized protein	12470415	12529652	2289288	513655	2904118	1087133	0.0634	6.1	Null
B8I5W2	RUMCH IMP dehydrogenase/GMP reductase	67783527	245922155	173003331	106773037	279883331	268825557	0.5836	1.3	Null
B8I5W3	RUMCH Transcription elongation factor GreA	1328248620	2913490878	2642835376	2619302210	1673945388	2124862447	0.9687	1.1	Null
B8I5W4	RUMCH Lysine--tRNA ligase	498451548	191053463	452815765	834040068	615864680	872097323	0.2565	2.0	Null
B8I5W5	RUMCH Uncharacterized protein	61529758	89875892	159225359	151874178	141247076	39010955	0.8983	1.1	Null
B8I5W6	RUMCH Anti-sigma factor antagonist	20582256	44349355	35910404	26852544	31196849	30337916	0.9679	1.1	Null
B8I5W7	RUMCH Putative anti-sigma regulatory factor, serine/threonine protein kin	5431629	8355950	4735685	2151845	3823914	19798169	0.6799	1.4	Null
B8I5W8	RUMCH RNA polymerase, sigma 28 subunit, Sig B/F/G subfamily	11921702	13652065	16589036	3575739	880179	5153069	0.0740	4.4	Null
B8I5X0	RUMCH Uncharacterized protein	9042893811	10878431321	15773752837	8103128032	7399766216	4393794826	0.2083	1.8	Null
B8I5X5	RUMCH Uncharacterized protein	884337	6602190	5826328	1093011	1307316	911599	0.1053	4.0	Null
B8I5X7	RUMCH Transcriptional regulator, MarR family	10953048	4169843	7129100	0	0	3108301	0.4773	7.2	Null
B8I5Y0	RUMCH Pyridoxal-5'-phosphate-dependent protein beta subunit	166484670	184137815	133976151	110027875	65444343	60380790	0.1126	2.1	Null
B8I5Y1	RUMCH Phosphoribosyltransferase	102832577	121101956	143641617	234117552	99595116	101288823	0.7349	1.2	Null
B8I5Y2	RUMCH Pyridoxal phosphate homeostasis protein	13074469	12862213	36490454	15434863	19547061	12336118	0.8101	1.3	Null
B8I5Y3	RUMCH ATP-grasp domain-containing protein	5007127	24003366	7002529	13303252	10357875	3488450	0.8468	1.3	Null
B8I5Y4	RUMCH Cellulase	712753147	579281898	252126011	158595508	281219826	260944777	0.2917	2.2	Null
B8I5Y7	RUMCH Uncharacterized protein	176852	366004	391540	291486	563162	425288	0.5980	1.4	Null
B8I5Z4	RUMCH Glycoside hydrolase family 5	13622931703	45709915181	29072514623	13576841172	7971815145	10037785161	0.0882	2.8	Null
B8I5Z5	RUMCH Cellulase	8635820120	22805194963	8433736456	9193553362	7489795601	6099750004	0.4153	1.8	Null
B8I5Z7	RUMCH Putative spore-coat protein	0	0	0	193503	342043	0	0.2232	NA	Null
B8I5Z8	RUMCH Uncharacterized protein	0	0	595628	0	0	0	0.2385	NA	Null
B8I5Z9	RUMCH DUF4825 domain-containing protein	22315089	34918126	51972283	179350089	111028376	86787441	0.0019	3.5	Increase
B8I601	RUMCH Glutamyl-tRNA(Gln) amidotransferase subunit A	740091	2128225	1476622	1432764	1550960	394925	0.8463	1.3	Null
B8I602	RUMCH Aspartyl/glutamyl-tRNA(Asn/Gln) amidotransferase subunit B	965042	1052728	2390538	1188874	1993043	451642	0.8927	1.2	Null
B8I606	RUMCH Cellulase	5354685086	16672621154	7351708105	5208304642	5195814537	5222901542	0.3599	1.9	Null
B8I609	RUMCH Uncharacterized protein	15501211	14717054	97110125	2466799	7723521	9450672	0.0767	6.5	Null
B8I611	RUMCH Peptidase M16 domain protein	16706731270	26189092579	18286239258	40813001891	24895878282	25299596516	0.2605	1.5	Null
B8I612	RUMCH FAD dependent oxidoreductase	266593	351123	0	521479	466888	0	0.8874	1.6	Null
B8I614	RUMCH Tryptophan synthase beta chain	149718206	180639723	227978010	224214152	265073802	170302824	0.5609	1.2	Null
B8I615	RUMCH Beta-galactosidase	0	0	0	0	150767	240927	0.2530	NA	Null
B8I618	RUMCH Glycoside hydrolase family 3 domain protein	0	407967	0	518053	221608	449398	0.6279	2.9	Null
B8I619	RUMCH Glycoside hydrolase family 31	514636	363488	307977	251410	0	181549	0.5626	2.7	Null
B8I622	RUMCH Extracellular solute-binding protein family 1	161334067	89767515	120653053	75682909	41806947	35997316	0.1121	2.4	Null
B8I623	RUMCH DUF218 domain-containing protein	2602570	1345822	508790	762311	728362	0	0.4982	3.0	Null
B8I624	RUMCH Germane domain-containing protein	391324251	906173333	1007847567	199339919	218268148	580497871	0.3821	2.3	Null
B8I625	RUMCH Acyl-ACP thioesterase	4161867	7420538	6334572	17219147	10181732	17263627	0.0207	2.5	Increase
B8I626	RUMCH Signal peptidase I	250533593	252729352	122274855	30643152	29379890	8298227	0.0002	9.2	Decrease
B8I627	RUMCH Pyrrolo-quinoline quinone	405390866	262085297	271261666	193650793	281084801	148172841	0.5167	1.5	Null
B8I628	RUMCH Pyrrolo-quinoline quinone	27821587	34969016	43339442	19830725	23547048	2854999	0.3183	2.3	Null
B8I629	RUMCH ATPase, P-type (Transporting), HAD superfamily, subfamily IC	20671377	35107469	8287816	23286199	43112261	18135560	0.7153	1.3	Null
B8I631	RUMCH Hydrogenase, Fe-only	0	1741504	2082747	675211	1790466	1467363	0.9269	1.0	Null
B8I632	RUMCH DNA mismatch repair protein MutS domain protein	800078	2858688	0	0	215542	684679	0.6657	4.1	Null
B8I634	RUMCH Inositol-1-monophosphatase	1803368	8660500	11118788	29092454	44863232	23393118	0.0150	4.5	Increase
B8I635	RUMCH AMP-dependent synthetase and ligase	10187765	30188710	17459706	6488523	5478758	4695627	0.0113	3.5	Decrease
B8I636	RUMCH Dihydropteroate synthase DHPS	27621740	16988250	8820493	42616833	877770	6071548	0.9366	1.1	Null
B8I637	RUMCH 5,10-methylenetetrahydrofolate reductase	2389991	2694710	1449070	5182867	1688081	3069113	0.5335	1.5	Null
B8I639	RUMCH Penicillin-binding protein transpeptidase	10952993	43407485	26169393	15813647	38173633	122488353	0.3137	2.2	Null
B8I644	RUMCH HAD-superfamily hydrolase, subfamily IA, variant 1	0	0	0	433091	282207	975997	0.0002	NA	Increase
B8I645	RUMCH Drug resistance transporter, EmrB/QacA subfamily	121558	0	0	489307	357745	241718	0.3065	9.0	Null
B8I646	RUMCH FAD-dependent pyridine nucleotide-disulphide oxidoreductase	136017585	126068325	119656647	625612533	536296016	370701757	0.0000	4.0	Increase
B8I647	RUMCH Hemerythrin-like metal-binding protein	4570337	2415601	0	288484	218754	0	0.2385	13.8	Null
B8I648	RUMCH L-lactate dehydrogenase	16441436	36115301	39175526	47936154	48670252	43344729	0.2447	1.5	Null
B8I649	RUMCH 3-dehydroquinate synthase	386812	248786	0	445300	630263	867727	0.4954	3.1	Null
B8I650	RUMCH 2,3-diketo-5-methylthio-1-phosphopentane phosphatase	0	1627777	0	795732	1181784	1094959	NA	1.9	Null
B8I651	RUMCH DEAD/DEAH box helicase domain protein	15421964	39074943	11595920	47162653	43148983	25276236	0.3232	1.7	Null
B8I652	RUMCH Na-Ca exchanger/integrin-beta4	9980927871	44715311642	4630347108	5193186719	6791239383	4029867979	0.1877	3.7	Null
B8I653	RUMCH Uncharacterized protein	633709	1866891	5866027	40316387	5206738	18771735	0.0229	7.7	Increase
B8I654	RUMCH Tail Collar domain protein	0	5206788	15980130	438286	7322462	7180289	0.9605	1.4	Null
B8I655	RUMCH Tail Collar domain protein	2810269	4213969	3119716	2772153	5229748	2275381	0.9149	1.0	Null
B8I657	RUMCH Nitroreductase	65207320	8266848	14068683	7753596	8294419	3591970	0.1070	4.5	Null
B8I658	RUMCH Hemerythrin HHE cation binding domain protein	0	0	0	0	0	184714	NA	NA	Null
B8I659	RUMCH Uncharacterized protein	396578	350719	368186	1315123	958882	864185	0.0210	2.8	Increase
B8I663	RUMCH Protein Tlp homolog	290350	0	0	0	0	0	0.2862	NA	Null
B8I664	RUMCH Uncharacterized protein	0	289429	0	0	0	0	0.3129	NA	Null
B8I666	RUMCH Aldo/keto reductase	381915161	124920983	88780689	28105950	32696915	65062821	0.0774	4.7	Null
B8I667	RUMCH Uncharacterized protein	0	0	0	179384	199205	0	0.2697	NA	Null
B8I670	RUMCH Adenylate cyclase	819402	6237543	3501264	4171695	1426075	1228127	0.7534	1.5	Null
B8I671	RUMCH Uncharacterized protein	1737878	2734814	3767273	3311880	2483901	1093137	0.8698	1.2	Null
B8I672	RUMCH Phosphopantetheine-binding	0	0	0	0	55385	0	NA	NA	Null
B8I673	RUMCH ATP-grasp domain-containing protein	0	7468211	10256909	0	102471	390122	0.1500	36.0	Null
B8I674	RUMCH Orn/DAP/Arg decarboxylase 2	496638	242881	652986	1837219	1812810	272440	0.2721	2.8	Null
B8I675	RUMCH Thioesterase	0	0	0	0	144840	44543	NA	NA	Null
B8I677	RUMCH Uncharacterized protein	0	270805	264260	34572	73512	0	0.5414	5.0	Null
B8I678	RUMCH Uncharacterized protein	7907737	2665428	3242346	1510608	1322457	70125	0.1432	4.8	Null
B8I679	RUMCH AMP-dependent synthetase and ligase	4567236	11475693	13967861	7467494	4855073	1079510	0.3517	2.2	Null
B8I680	RUMCH BtrH	163802	0	0	0	1269965	734344	0.4174	12.2	Null
B8I681	RUMCH Phosphopantetheine-binding	0	0	0	0	0	180159	NA	NA	Null
B8I682	RUMCH 5'-Nucleotidase domain protein	56548654	77258081	33623598	7154543	2137596	13002974	0.0144	7.5	Decrease
B8I685	RUMCH ABC transporter related	2981629	4725015	17283498	35311237	22966392	20690028	0.0939	3.2	Null
B8I688	RUMCH Beta-lactamase	1765065	16426993	22482906	8541172	14305777	16146389	0.9228	1.0	Null
B8I689	RUMCH Methyl-accepting chemotaxis sensory transducer	107369982	362609085	109401938	522547081	371459484	474615591	0.1384	2.4	Null
B8I692	RUMCH Uncharacterized protein	1806108697	1978975626	2458950116	2923940173	2476657174	3226174411	0.3084	1.4	Null
B8I693	RUMCH Cellulase	193378995	186779031	260050765	120497313	391629467	79251805	0.9754	1.1	Null
B8I694	RUMCH Uncharacterized protein	637349	220099	702017	1241889	562060	359130	0.7723	1.4	Null
B8I695	RUMCH Uncharacterized protein	5838424	3487373	1218090	4820295	8948970	6469228	0.4472	1.9	Null
B8I696	RUMCH Meso-diaminopimelate D-dehydrogenase	229566918	151276458	135474072	186844288	66759725	132873624	0.7442	1.3	Null
B8I697	RUMCH Diaminopimelate decarboxylase	22782301	8531847	14543714	13019994	5936403	5766959	0.3752	1.9	Null
B8I698	RUMCH Transcriptional regulator, TrmB	432972	772344	0	1678677	1456724	2025776	0.3051	4.3	Null
B8I6A1	RUMCH ATPase associated with various cellular activities AAA	16648632	22373916	2666071	14617223	37727077	4934717	0.8132	1.4	Null
B8I6A4	RUMCH Uncharacterized protein	6144613	4424238	6058824	6308100	9993662	5206578	0.6115	1.3	Null
B8I6A5	RUMCH Metallophosphoesterase	710057	912096	377465	1010974	501528	207500	0.8915	1.2	Null
B8I6A6	RUMCH Uncharacterized protein	145423	948171	2850388	1084979	2780969	3057522	0.5389	1.8	Null
B8I6A7	RUMCH Flavodoxin/nitric oxide synthase	508524289	522675807	52418776	20522026	72651254	24436138	0.0193	9.2	Decrease
B8I6A8	RUMCH Glutamate synthase (NADPH), homotetrameric	88025358	13367777	21874478	49210277	38360563	38674377	0.9871	1.0	Null
B8I6A9	RUMCH Oxidoreductase FAD/NAD(P)-binding domain protein	70177716	81020993	131832988	52587708	52106917	43443961	0.1545	1.9	Null
B8I6B0	RUMCH Peptidase M50	0	0	0	0	55886	0	NA	NA	Null
B8I6B1	RUMCH Peptidase M23	34955644	119648795	146334309	35548985	14905988	7455541	0.0254	5.2	Decrease
B8I6B9	RUMCH MGS domain protein	1604555	1356508	821558	1249853	959324	2957113	0.6720	1.4	Null
B8I6C0	RUMCH Cell division topological specificity factor	15457193	28245729	10683112	42996081	34874752	33304425	0.1415	2.0	Null
B8I6C1	RUMCH Cell division inhibitor MinD	80793503	242455665	228653143	506590399	492862981	514926153	0.0184	2.7	Increase
B8I6C2	RUMCH Probable septum site-determining protein MinC	9222967	115557837	78947489	42957765	44682087	51690714	0.8494	1.5	Null
B8I6C3	RUMCH Penicillin-binding protein 2	540032321	554974911	644545783	116125474	104610399	32835204	0.0001	6.9	Decrease
B8I6C5	RUMCH ABC transporter related	0	0	225172	277307	483995	0	0.7386	3.4	Null
B8I6C7	RUMCH Extracellular solute-binding protein family 3	0	0	0	1040268	592544	0	0.0552	NA	Null
B8I6D2	RUMCH 30S ribosomal protein S2	399194650	1010208510	573991083	794908332	514661771	211222294	0.7950	1.3	Null
B8I6D3	RUMCH Elongation factor Ts	3671489184	11594858526	19535157707	5197666594	2145614687	2811003034	0.1085	3.4	Null
B8I6D4	RUMCH Uridylate kinase	78129418	107519775	137552956	108716950	81687385	73686937	0.7783	1.2	Null
B8I6D5	RUMCH Ribosome-recycling factor	22046657094	12274997628	35801794937	1282370411	273612045	1552815759	0.0000	22.6	Decrease
B8I6D7	RUMCH Isoprenyl transferase	6215989	1306254	2683043	2892536	2667666	4594132	0.9960	1.0	Null
B8I6D9	RUMCH 1-deoxy-D-xylulose 5-phosphate reductoisomerase	4767334	1021260	1382333	597327	1968908	448623	0.3901	2.4	Null

B8I6E0	RUMCH Zinc metalloprotease	0	646014	18842925	32344102	457947	20510987	0.6481	2.7	Null
B8I6E1	RUMCH 4-hydroxy-3-methylbut-2-en-1-yl diphosphate synthase (flavodoxi	3067300	2737578	3809338	3987822	3331464	3144689	0.7841	1.1	Null
B8I6E2	RUMCH DNA polymerase III PolC-type	86873	202152	0	881679	16074920	1136480	0.0032	62.6	Increase
B8I6E3	RUMCH Ribosome maturation factor RimP	6386690	5806502	0	7761519	1084125	5724801	0.9455	1.2	Null
B8I6E4	RUMCH Transcription termination/antitermination protein NusA	477649044	986833789	2689329556	119966569	135593413	128498255	0.0002	10.8	Decrease
B8I6E6	RUMCH Ribosomal protein L7Ae/L30e/S12e/Gadd45	5353959	22192669	7201582	222619	1083205	0	0.0230	26.6	Decrease
B8I6E7	RUMCH Translation initiation factor IF-2	3345291562	11893837546	11839563768	2325195214	2615619684	4722077154	0.2245	2.8	Null
B8I6E8	RUMCH Ribosome-binding factor A	6497569	3552569	15592053	3467859	274985	3206972	0.2673	3.7	Null
B8I6E9	RUMCH Phosphoesterase RecJ domain protein	0	2214058	2670848	6712788	1743089	6805343	0.4532	3.1	Null
B8I6F0	RUMCH tRNA pseudouridine synthase B	673408	830365	772482	1523000	1014727	574944	0.5732	1.4	Null
B8I6F1	RUMCH Riboflavin biosynthesis protein	792106	0	333929	1479604	871572	1778933	0.4394	3.7	Null
B8I6F2	RUMCH Carbamoyltransferase	0	0	0	56324	61418	0	NA	NA	Null
B8I6F3	RUMCH Hydrogenase assembly chaperone hypC/hupF	668057	1590045	1413092	3030506	2121249	2786529	0.0723	2.2	Null
B8I6F4	RUMCH Hydrogenase expression/formation protein HypD	2177771	2473308	3599022	3142218	2960237	1557808	0.9730	1.1	Null
B8I6F5	RUMCH Hydrogenase expression/formation protein HypE	1077894	14156668	0	520354	464498	234259	0.2050	12.5	Null
B8I6F6	RUMCH ABC transporter related	21227260	10155694	7576409	6639321	4826312	335150	0.2490	3.3	Null
B8I6F8	RUMCH Efflux transporter, RND family, MFP subunit	6810229744	3377002069	1077907087	476797130	161120518	263646734	0.0010	12.5	Decrease
B8I6F9	RUMCH Uncharacterized protein	315272	8613813	13288216	8286098	4980221	266876	0.8151	1.6	Null
B8I6G1	RUMCH Peptidase M16 domain protein	4839739	4222393	24714600	5505161	8351355	3634993	0.5609	1.9	Null
B8I6G2	RUMCH Peptidase M16 domain protein	2808695	4925665	5588532	10318069	6716800	7455207	0.0881	1.8	Null
B8I6G3	RUMCH Phosphatidylglycerol--prolipoprotein diacylglyceryl transferase	3408565	10665106	7981754	13502382	4904331	12259924	0.5573	1.4	Null
B8I6G4	RUMCH Rod shape-determining protein RodA	0	20938	0	68692	83036	0	NA	7.2	Null
B8I6G5	RUMCH Transcriptional regulator MraZ	2518614	3393673	4264361	129688	79093	33394	0.0000	42.0	Decrease
B8I6G6	RUMCH Ribosomal RNA small subunit methyltransferase H	9327785	7136247	6242333	6411550	5764556	4159172	0.5732	1.4	Null
B8I6G7	RUMCH Cell division protein FtsL	1381893	2702676	476262	702193	1108106	0	0.6211	2.5	Null
B8I6G8	RUMCH Penicillin-binding protein transpeptidase	991205273	3948584792	1034758964	761021310	899680247	439943052	0.1914	2.8	Null
B8I6G9	RUMCH UDP-N-acetylmuramoyl-L-alanyl-D-glutamate--2,6-diaminopimela	11623095	30649145	15637015	87719366	63665563	69394171	0.0010	3.8	Increase
B8I6H0	RUMCH UDP-N-acetylmuramoyl-tripeptide--D-alanyl-D-alanine ligase	1015872	16674078	6933944	13632796	9655088	14031439	0.5952	1.5	Null
B8I6H1	RUMCH Phospho-N-acetylmuramoyl-pentapeptide-transferase	0	0	0	0	33883	0	NA	NA	Null
B8I6H3	RUMCH UDP-N-acetylglucosamine--N-acetylmuramyl-(pentapeptide) pyro	412374	891034	468835	8202480	2165471	2370205	0.0016	7.2	Increase
B8I6H4	RUMCH Small acid-soluble spore protein alpha/beta type	16287943	4390470	0	2756299	8404177	0	0.8350	1.9	Null
B8I6H6	RUMCH Monogalactosyldiacylglycerol synthase	1715169	3607245	0	996159	333707	0	0.5920	4.0	Null
B8I6H7	RUMCH Serine-type D-Ala-D-Ala carboxypeptidase	186126	0	246615	0	0	69729	0.6492	6.2	Null
B8I6H9	RUMCH Putative serine protein kinase, PrkA	657833	8338900	3379505	14304910	15890216	3669242	0.2681	2.7	Null
B8I6I0	RUMCH UPF0229 protein CceI	0	287537	469800	119268	319089	448578	0.8697	1.2	Null
B8I6I1	RUMCH SpoVR family protein	0	669194	208464	296180	463478	71665	0.9809	1.1	Null
B8I6I2	RUMCH Heat shock protein Hsp90	1204529637	655760579	831306260	895079796	707593598	367117078	0.6445	1.4	Null
B8I6I3	RUMCH Methyl-accepting chemotaxis sensory transducer	0	306232	0	0	172779	86144	0.9972	1.2	Null
B8I6I4	RUMCH AMP-dependent synthetase and ligase	40061327	85182903	117744428	58526042	125579238	87313891	0.7683	1.1	Null
B8I6I5	RUMCH Transcriptional regulator, XRE family	12034127	18117026	31172402	715194871	451011278	542001558	0.0000	27.9	Increase
B8I6I6	RUMCH Methyl-accepting chemotaxis sensory transducer	415292436	427848175	298225553	640802471	405422720	508200529	0.4708	1.4	Null
B8I6I7	RUMCH Metal dependent phosphohydrolase	99380061	34315034	26253585	54864345	51060595	39786703	0.9115	1.1	Null
B8I6I8	RUMCH Transcriptional regulator, LysR family	486166	0	0	0	0	0	0.2327	NA	Null
B8I6I9	RUMCH Phosphoribosylformylglycinamidine synthase	6137910	5854035	5450733	8208402	9088020	19940220	0.1985	2.1	Null
B8I6J0	RUMCH Xylose isomerase domain protein TIM barrel	228410364	381669182	472232887	266962701	288768978	208465788	0.5467	1.4	Null
B8I6J1	RUMCH Uncharacterized protein	1934269	3320393	1130705	3320002	2141509	1734571	0.8463	1.1	Null
B8I6K3	RUMCH Patatin	0	344528	0	0	212815	0	0.9475	1.6	Null
B8I6K5	RUMCH Uncharacterized protein	0	0	975253	0	0	4703600	NA	4.8	Null
B8I6K6	RUMCH Uncharacterized protein	0	0	0	0	0	43683	NA	NA	Null
B8I6K9	RUMCH 4-hydroxy-tetrahydrodipicolinate reductase	5740463	9728568	13252652	7118369	4724203	3918611	0.2670	1.8	Null
B8I6L0	RUMCH Serine-type D-Ala-D-Ala carboxypeptidase	1574792	1839938	2331155	11090892	992296	16484810	0.0780	5.0	Null
B8I6L3	RUMCH Germination protein, Ger(X)C family	1611776	6525129	159013	210200	0	377382	0.1484	14.1	Null
B8I6L4	RUMCH Aspartate kinase	269517	0	0	5116802	4461748	427483	0.0805	37.1	Null
B8I6L5	RUMCH 4-hydroxy-tetrahydrodipicolinate reductase	0	9295916	23074843	54340430	53366724	63448396	0.3063	5.3	Null
B8I6L6	RUMCH 4-hydroxy-tetrahydrodipicolinate synthase	6450565	7363071	11932612	24453111	10165810	21566569	0.1280	2.2	Null
B8I6L7	RUMCH SpoIID/LytB domain protein	409756083	1025819267	683642041	436102854	445125264	581581435	0.6791	1.4	Null
B8I6L8	RUMCH Aspartokinase	23170376	43737192	40585719	108830598	20029772	43072412	0.5085	1.6	Null
B8I6L9	RUMCH S-adenosylmethionine:tRNA ribosyltransferase-isomerase	0	0	0	2707807	336044	0	0.0270	NA	Increase
B8I6M0	RUMCH Queuine tRNA-ribosyltransferase	0	64718	270942	0	0	0	0.2989	NA	Null
B8I6M1	RUMCH Preprotein translocase, YajC subunit	3083937	4059530	8326725	49038800	16413352	11443201	0.0124	5.0	Increase
B8I6M4	RUMCH Radical SAM domain protein	4924932	8298663	3097272	1774765	990782	23719018	0.6546	1.6	Null
B8I6M6	RUMCH Protein translocase subunit SecD	405461168	939510872	454050327	683286814	805708007	290967385	0.9560	1.0	Null
B8I6M7	RUMCH Protein-export membrane protein SecF	6051198	51031396	5852957	19733223	97432422	243972249	0.0982	5.7	Null
B8I6M9	RUMCH Uncharacterized protein	347119	323007	913462	1738206	1444273	930591	0.1173	2.6	Null
B8I6N0	RUMCH DNA primase	767821	24610828	0	2202309	1475335	238971	0.3976	6.5	Null
B8I6N1	RUMCH RNA polymerase sigma factor SigA	71755368	16031768	104218607	19320947	19902125	5151331	0.0924	4.3	Null
B8I6N3	RUMCH Uncharacterized protein	238007	339415	514241	371545	265230	0	0.7930	1.7	Null
B8I6N4	RUMCH GTP cyclohydrolase 1 type 2 homolog	0	0	234627	383940	0	0	0.9163	1.6	Null
B8I6N5	RUMCH Uncharacterized protein	4606427	7797439	8150560	4633542	3192084	3734601	0.2312	1.8	Null
B8I6N6	RUMCH 4Fe-4S ferredoxin iron-sulfur binding domain protein	0	89972	128226	0	0	0	NA	NA	Null
B8I6N7	RUMCH Amidohydrolase 2	1269454	2233632	8793933	19997651	2047611	1481349	NA	1.9	Null
B8I6N8	RUMCH Uncharacterized protein	0	0	0	0	114484	0	NA	NA	Null
B8I6N9	RUMCH NADPH-dependent FMN reductase	17602747	30557733	62169887	16966254	12452849	0	0.5085	3.8	Null
B8I6P1	RUMCH Uncharacterized protein	34553322	22334394	18494110	53705840	61526099	159935162	0.0627	3.7	Null
B8I6P2	RUMCH DNA mismatch repair protein MutS domain protein	0	447112	0	0	0	0	0.2688	NA	Null
B8I6P3	RUMCH 4Fe-4S ferredoxin iron-sulfur binding domain protein	2757686	9267629	70070176	0	0	0	0.0000	NA	Decrease
B8I6P4	RUMCH Pyruvate flavodoxin/ferredoxin oxidoreductase domain protein	30058262	19279591	26728614	20957657	5399908	4061565	0.2370	2.5	Null
B8I6P5	RUMCH Thiamine pyrophosphate protein domain protein TPP-binding	29851602	44015012	2511679	9337794	6861247	9550902	0.3108	3.0	Null
B8I6P6	RUMCH Pyruvate ferredoxin/flavodoxin oxidoreductase	6863854	64215282	10891585	23452973	42449035	41715723	0.7087	1.3	Null
B8I6P7	RUMCH ABC transporter related	0	0	555256	508410	0	685657	0.8147	2.2	Null
B8I6P8	RUMCH Cell shape-determining protein MreC	126069939	75707181	93099738	162038982	52926283	42519997	0.8698	1.1	Null
B8I6P9	RUMCH Cell shape-determining protein MreB	73173022	72165077	175204606	142091918	51300622	106643666	0.9862	1.1	Null
B8I6Q0	RUMCH DNA repair protein RadC	0	0	0	225888	191400	464166	0.0025	NA	Increase
B8I6Q1	RUMCH dTTP/UTP pyrophosphatase	1827303	8792099	0	3137049	543336	159534	0.6592	2.8	Null
B8I6Q2	RUMCH Negative regulator of genetic competence	413170	292225	36908148	14565385	802136	154905	NA	2.4	Null
B8I6Q3	RUMCH D-malate dehydrogenase [decarboxylating]	22842220	95730196	57539298	88284747	115041592	84171451	0.3177	1.6	Null
B8I6Q4	RUMCH Lon protease	41797369	33619763	23480050	54429634	41956466	19549119	0.8463	1.2	Null
B8I6Q5	RUMCH Pyruvate kinase	48395851	19752053	19611527	35046009	27829118	34710019	0.9031	1.1	Null
B8I6Q7	RUMCH Citrate synthase	10294368	24426715	36764403	22337870	35973466	36405630	0.5573	1.3	Null
B8I6Q8	RUMCH SEC-C motif domain protein	155791381	43278563	68485863	45184836	34638492	37588816	0.2697	2.3	Null
B8I6Q9	RUMCH DUF4397 domain-containing protein	15623436	10279181	21596551	6814454	24252659	1021835	0.7925	1.5	Null
B8I6R0	RUMCH Asparagine--tRNA ligase	1550242	91704818	11109453	20782203	10526281	12337148	0.6048	2.4	Null
B8I6R1	RUMCH Aspartate--ammonia ligase	864551362	7241836	8263537	21263980	12415403	8091924	NA	21.1	Null
B8I6R2	RUMCH Isocitrate dehydrogenase [NADP]	201923410	388607463	160158823	135693761	229665501	258955207	0.9320	1.2	Null
B8I6R3	RUMCH Putative carbohydrate binding	0	0	0	179519	0	0	NA	NA	Null
B8I6R4	RUMCH Uncharacterized protein	114794	330218	173531	180657	194547	147446	0.9585	1.2	Null
B8I6R5	RUMCH Thioesterase superfamily protein	0	0	0	0	242772	357974	0.1759	NA	Null
B8I6R7	RUMCH Pyruvate formate-lyase-activating enzyme	0	1897389	0	1489891	0	1329416	0.8903	1.5	Null
B8I6R8	RUMCH Formate acetyltransferase	2906584	7142805	5180412	3018592	1896484	4635228	0.6608	1.6	Null
B8I6R9	RUMCH Uncharacterized protein	7233693900	7542040208	10269899196	8512145539	16795155198	9041393094	0.4708	1.4	Null
B8I6S0	RUMCH Uncharacterized protein	2527774	14495169	0	0	2178861	13294158	0.9704	1.1	Null
B8I6S1	RUMCH Peptidase M48 Ste24p	394897	574372	0	0	0	0	0.1202	NA	Null
B8I6S3	RUMCH ABC transporter related	0	0	0	51121	399747	677369	0.0047	NA	Increase
B8I6S5	RUMCH Extracellular solute-binding protein family 3	1967238	1269439	1963215	1779304	1729907	2109300	0.8488	1.1	Null
B8I6S6	RUMCH ErfK/YbiS/YcfS/YnhG family protein	156355411	244520621	364536252	167527252	87201342	100965062	0.1663	2.2	Null
B8I6S7	RUMCH Aspartate-semialdehyde dehydrogenase	6151352	25197771	16853251	4525830	5712967	6021059	0.1240	3.0	Null
B8I6S8	RUMCH Cyclic nucleotide-binding domain-containing protein	0	554574	382922	1594276	1224457	0	0.6540	3.0	Null
B8I6S9	RUMCH Endoribonuclease L-PSP	2781689	4449960	7517118	1954295	1281282	1744818	0.0557	3.0	Null
B8I6T0	RUMCH Gamma-glutamyl phosphate reductase	70347994	102179863	122825810	60970304	102649295	41726174	0.6099	1.4	Null
B8I6T1	RUMCH Glycerol kinase	649566	12464918	19409529	28848492	23090896	20237294	0.3836	2.2	Null
B8I6T5	RUMCH Uncharacterized protein	52705	638844	0	2837579	1825738	658377	0.1939	7.7	Null
B8I6T6	RUMCH Radical SAM domain protein	2629965	828388	0	0	1120557	0	0.7582	3.1	Null

B8I6T8	RUMCH Uncharacterized protein	0	0	710549	8048510	2832102	3093020	0.1262	19.7	Null
B8I6T9	RUMCH 4-hydroxythreonine-4-phosphate dehydrogenase	0	3503656	0	174172	1161355	0	0.8463	2.6	Null
B8I6U0	RUMCH Transcriptional regulator, GntR family	16350774	16856857	31086757	9017530	6710891	10470732	0.1173	2.5	Null
B8I6U1	RUMCH ABC transporter related	3663097	3044647	1757787	3054277	2332595	1492813	0.7986	1.2	Null
B8I6U2	RUMCH Uncharacterized protein	392167	11321143	5745038	497110	1454894	2586320	0.3462	3.8	Null
B8I6U4	RUMCH ABC transporter related	404427	78268	0	377711	884199	72525	0.6657	2.8	Null
B8I6U5	RUMCH Transcriptional regulator, GntR family	0	10725568	12386100	8389091	10591707	11162147	0.8496	1.3	Null
B8I6U7	RUMCH Glycerate kinase	2599363	2215679	6845946	6100749	3556462	5770370	0.6279	1.3	Null
B8I6U8	RUMCH ATP-dependent 6-phosphofructokinase	561243685	409209605	752610701	299795170	331534150	610603880	0.7816	1.4	Null
B8I6U9	RUMCH Phosphatidylserine decarboxylase proenzyme	0	264052	367592	248252	0	0	0.8324	2.5	Null
B8I6V0	RUMCH Glycoside hydrolase family 43	48163175	42341710	67564377	22265593	4199795	5121416	0.0244	5.0	Decrease
B8I6V1	RUMCH Methyltransferase type 11	283701	2395960	12211351	34269664	1698655	892786	0.5907	2.5	Null
B8I6V4	RUMCH Short-chain dehydrogenase/reductase SDR	2080127300	1576805830	731488498	1110400886	1521851529	1271939908	0.9255	1.1	Null
B8I6V5	RUMCH Translation initiation factor IF-3	39527958	22756166	108199317	32239798	29606141	29392740	0.4842	1.9	Null
B8I6V7	RUMCH Glucanase	379284239	98577911	28564857	95984296	104491603	201887956	0.8708	1.3	Null
B8I6W8	RUMCH S-layer domain protein	215273	20676576	3265338	3332059	3102215	3798197	0.6465	2.4	Null
B8I6W9	RUMCH Uncharacterized protein	0	0	0	0	328029	0	0.2813	NA	Null
B8I6X2	RUMCH Transcriptional regulator, MerR family	11976774	9936416	17162063	5507648	5278333	5202079	0.0423	2.4	Decrease
B8I6X4	RUMCH Ig domain protein group 2 domain protein	12546750	16638002	15504077	14670242	8993313	6953621	0.4387	1.5	Null
B8I6X6	RUMCH Cell wall/surface repeat protein	0	390688	0	0	155320	143303	0.9943	1.3	Null
B8I6Y7	RUMCH Uncharacterized protein	322607	0	0	0	0	0	0.2769	NA	Null
B8I6Z4	RUMCH Periplasmic binding protein	0	137244	264094	178799	346348	0	0.9041	1.3	Null
B8I6Z5	RUMCH zf-trcl domain-containing protein	0	0	0	1067755	892310	1000227	0.0000	NA	Increase
B8I6Z6	RUMCH Translation initiation factor IF-3	0	0	0	0	579213	0	0.2225	NA	Null
B8I700	RUMCH Radical SAM domain protein	0	0	0	132952	0	53521	NA	NA	Null
B8I701	RUMCH Glyoxalase-like	0	322095	0	3529812	2104845	2361296	0.0573	24.8	Null
B8I706	RUMCH Transcriptional regulator, AraC family	0	0	0	0	666109	297284	0.1046	NA	Null
B8I708	RUMCH Uncharacterized protein	0	29778	0	191111	310473	237694	0.0333	24.8	Increase
B8I711	RUMCH ABC transporter related	0	0	0	1249853	49269	124573	0.0074	NA	Increase
B8I712	RUMCH Putative solute-binding component of ABC transporter	29768517	28932968	47739294	14948656	17120938	13180101	0.0399	2.4	Decrease
B8I713	RUMCH Transcriptional regulator, DeoR family	0	0	0	1391186	708960	2405074	0.0000	NA	Increase
B8I714	RUMCH ABC transporter related	938188	2129043	57912575	181329575	171800131	6248552	NA	5.9	Null
B8I719	RUMCH Glutamine synthetase catalytic region	14047700	11131922	15636005	41990537	27613910	26229065	0.0219	2.3	Increase
B8I720	RUMCH Transglutaminase domain protein	2673675	796369	256176	382195	337150	0	0.2855	5.2	Null
B8I723	RUMCH Peptidase M24	0	0	0	90154	0	0	NA	NA	Null
B8I724	RUMCH Polysaccharide deacetylase	8403607	8480265	666654	8354248	3355446	441776	0.7930	1.4	Null
B8I726	RUMCH Uncharacterized protein	27825495	81615839	45342126	60617513	1830243	7537034	0.5573	2.2	Null
B8I727	RUMCH Uncharacterized protein	36317511	3429558	1920928	1846313	2417577	1763311	NA	6.9	Null
B8I728	RUMCH Polysaccharide deacetylase	3499085	5789029	134317	3474502	0	4259721	0.9692	1.2	Null
B8I730	RUMCH Sporulation protein YqfD	0	0	809894	89912	0	0	NA	9.0	Null
B8I731	RUMCH PhoH family protein	15351371	18281180	49419631	53164882	36621465	19707320	0.6628	1.3	Null
B8I732	RUMCH Metal dependent phosphohydrolase	1870537	6786261	2877041	817393	1352892	898463	0.0521	3.8	Decrease
B8I733	RUMCH Endoribonuclease YbeY	0	0	0	0	168062	80144	0.3007	NA	Null
B8I735	RUMCH Cytidine deaminase	7825008	7799151	11518249	2146105	4121295	5203713	0.2342	2.4	Null
B8I736	RUMCH GTPase Era	384794	1387580	4939256	7882586	3317694	2225139	0.4616	2.0	Null
B8I738	RUMCH DNA repair protein RecO	0	0	180753	284263	179607	302434	0.4862	4.2	Null
B8I740	RUMCH ABC transporter related	196612	529524	300206	256298	298769	219454	0.8463	1.3	Null
B8I741	RUMCH ABC transporter transmembrane region	0	0	0	45956	106338	61336	NA	NA	Null
B8I742	RUMCH Uncharacterized protein	0	0	0	754148	0	0	NA	NA	Null
B8I744	RUMCH UPF0291 protein Ccel	21417729	20444087	33021543	197571	0	4437717	0.1580	16.2	Null
B8I745	RUMCH CheW protein	5187297	1754581	83423610	166492685	78581652	81268733	0.2886	3.6	Null
B8I747	RUMCH Ribosome-binding ATPase YchF	0	1173060	859538	2062754	347906	671038	0.8062	1.5	Null
B8I749	RUMCH Transcriptional regulator, MerR family	87498153	24315238	9656709	3069986	28674609	25068756	0.5750	2.1	Null
B8I750	RUMCH Pseudouridine synthase	243275	1266716	713569	522026	149611	713239	0.8015	1.6	Null
B8I751	RUMCH Aluminium resistance family protein	2036227	5406905	1116930	2856542	1574587	2874070	0.9585	1.2	Null
B8I752	RUMCH Uncharacterized protein	3840191	5025325	7694866	2668644	1569283	1539743	0.0226	2.9	Decrease
B8I753	RUMCH Pyridoxal phosphate homeostasis protein	1580568	0	0	6081742	929401	422866	0.6297	4.7	Null
B8I754	RUMCH Cell division protein SepF	1471542	10822422	4236276	1979901	2777388	4653926	0.7146	1.8	Null
B8I756	RUMCH RNA-binding S4 domain protein	602607	6020637	2304917	5085201	4058906	3497893	0.6258	1.4	Null
B8I757	RUMCH DivIVA family protein	774720139	1261267776	2243664806	582266244	371005839	800584626	0.2400	2.4	Null
B8I758	RUMCH Cell divisionFtsK/SpoIIIE	185831563	18744541	22750716	25749442	19017457	5412622	NA	4.5	Null
B8I760	RUMCH UPF0313 protein Ccel	1829868	563903	674016	628045	395153	550497	0.4532	1.9	Null
B8I761	RUMCH Bifunctional protein FolD	42875180	343363874	465926094	648223659	816237860	559799248	0.2494	2.4	Null
B8I762	RUMCH Ribonuclease Y	213011247	323633388	202945306	147756838	88473655	437917803	0.9686	1.1	Null
B8I763	RUMCH Metallophosphoesterase	919698	254113	414709	7697771	6777145	6197259	0.0000	13.0	Increase
B8I764	RUMCH Purine nucleoside phosphorylase	2554972	3492044	2885071	3004640	220356	55374	NA	2.7	Null
B8I765	RUMCH Extracellular solute-binding protein family 5	4922808	4465115	13327574	8366243	11269817	19905981	0.3659	1.7	Null
B8I766	RUMCH Lipoprotein signal peptidase	0	0	0	1208467	1269965	454039	0.0000	NA	Increase
B8I767	RUMCH Pseudouridine synthase	6339702	10182298	15024535	2650872	3423983	4442273	0.0660	3.0	Null
B8I768	RUMCH Bifunctional protein PyrR	91680128	120947284	308118538	452928236	320690766	403379817	0.1280	2.3	Null
B8I769	RUMCH Aspartate carbamoyltransferase	31417801	22355659	29516988	46778506	243901995	14311816	0.2159	3.7	Null
B8I770	RUMCH Dihydroorotase	10598477	4304989	9862635	17657305	7994715	6375434	0.7805	1.3	Null
B8I771	RUMCH Orotidine 5'-phosphate decarboxylase	31239697	82788574	27405885	14263093	33951160	66041805	0.9667	1.2	Null
B8I772	RUMCH Carbamoyl-phosphate synthase small chain	3302092	28589083	33108540	5180353	8614012	6627165	0.2557	3.2	Null
B8I773	RUMCH Carbamoyl-phosphate synthase large chain	85206071	163530243	507222256	266584460	301927247	119287535	0.9972	1.1	Null
B8I774	RUMCH Dihydroorotate dehydrogenase B (NAD(+)), electron transfer subu	0	4682202	4752643	5486014	5302222	2770587	0.8236	1.4	Null
B8I775	RUMCH Dihydroorotate dehydrogenase	564299	979251	0	594788	0	271125	0.8572	1.8	Null
B8I776	RUMCH Phosphoglycerate mutase	379405	1703706	0	0	361185	528978	0.8350	2.3	Null
B8I777	RUMCH Uncharacterized protein	9636000	6069311	7786278	5297458	5229558	10752306	0.9840	1.1	Null
B8I778	RUMCH Uncharacterized protein	169254	0	0	0	0	0	NA	NA	Null
B8I779	RUMCH Cys-tRNA(Pro)/Cys-tRNA(Cys) deacylase	1330027	0	1168233	201740	0	0	0.3622	12.4	Null
B8I780	RUMCH Uncharacterized protein	18386358	25570291	17474970	88509405	176001160	88648875	0.0000	5.7	Increase
B8I781	RUMCH Radical SAM domain protein	0	0	0	0	76354	81441	NA	NA	Null
B8I783	RUMCH Diguanylate cyclase/phosphodiesterase	376470	184744	277668	444691	339071	332729	0.7429	1.3	Null
B8I784	RUMCH UPF0251 protein Ccel	10577910	1217295	6962641	1488122	1624857	1856169	0.1571	3.8	Null
B8I785	RUMCH Iron-sulfur cluster carrier protein	126591354	459451516	118028426	709131325	765191329	436871550	0.1114	2.7	Null
B8I787	RUMCH Transcriptional regulator, LysR family	335257	995453	1086599	7654946	1910032	2522394	0.0162	5.0	Increase
B8I791	RUMCH Flavodoxin	8704533	7163907	4137020	1750288	2810984	709073	0.0512	3.8	Decrease
B8I792	RUMCH Aromatic amino acid beta-eliminating lyase/threonine aldolase	0	0	591601	1365734	123254	1171152	0.5013	4.5	Null
B8I793	RUMCH Uncharacterized protein	17894362	12434852	21838015	2218282	4403229	5284302	0.0181	4.4	Decrease
B8I794	RUMCH Uncharacterized protein	65111382	27463390	27261182	10310726	14224848	4979175	0.0366	4.1	Decrease
B8I795	RUMCH Short-chain dehydrogenase/reductase SDR	0	145884	0	129702	218778	0	0.8260	2.4	Null
B8I799	RUMCH FMN-dependent NADH:quinone oxidoreductase	0	12575718	0	0	0	1043071	NA	12.1	Null
B8I7A0	RUMCH Glycoside hydrolase family 18	0	0	0	117604	0	0	NA	NA	Null
B8I7A3	RUMCH Nicotinate-nucleotide--dimethylbenzimidazole phosphoribosyltra	5852954	6696018	7720737	6547418	9529825	2485017	0.9573	1.1	Null
B8I7A4	RUMCH Uncharacterized protein	0	0	0	124429	0	0	NA	NA	Null
B8I7A5	RUMCH Fibronectin type III domain protein	1246837223	1118232524	881495417	1646808215	1974201337	796045708	0.5907	1.4	Null
B8I7A6	RUMCH Cellulase	9977602429	12508641290	11005251697	10166104025	23008406352	11752852121	0.5325	1.3	Null
B8I7A8	RUMCH Uncharacterized protein	0	0	502885	0	236220	0	0.8997	2.1	Null
B8I7B2	RUMCH Uncharacterized protein	0	0	0	749924	128985	0	0.1455	NA	Null
B8I7B3	RUMCH Uncharacterized protein	0	0	0	762993	2751590	1790743	0.0000	NA	Increase
B8I7B4	RUMCH Uncharacterized protein	254093	0	0	68130	0	0	0.7445	3.7	Null
B8I7B5	RUMCH GCN5-related N-acetyltransferase	7103450	94071	3356639	5238168	5328220	6165712	0.7945	1.6	Null
B8I7B8	RUMCH HAD superfamily (Subfamily IIIA) phosphatase, TIGR01668	3569502	17829002	10481313	2859316	3399023	10882980	0.6955	1.9	Null
B8I7B9	RUMCH Shikimate dehydrogenase (NADP(+))	26816669	28577093	45359701	31747070	60230841	46295163	0.4624	1.4	Null
B8I7C0	RUMCH Uncharacterized protein	6973704	0	0	0	0	0	NA	NA	Null
B8I7C2	RUMCH Cell envelope-related transcriptional attenuator	38952010	77863816	60846151	6303255	17998273	6591423	0.0027	5.8	Decrease
B8I7C3	RUMCH Indolepyruvate oxidoreductase subunit IorA	20533182	65937647	20735864	7030522	17378664	8623769	0.1372	3.2	Null
B8I7C4	RUMCH Pyruvate ferredoxin/flavodoxin oxidoreductase	61888553	4205119	2960554	9403066	8968586	5886510	NA	2.8	Null
B8I7C5	RUMCH Phenylacetate-coenzyme A ligase	687926221	227458754	290809954	280135320	656962691	177014491	0.9302	1.1	Null
B8I7C6	RUMCH Amino acid-binding ACT domain protein	23206279	42457300	68229768	299221680	245770366	320718665	0.0000	6.5	Increase
B8I7C8	RUMCH Thioesterase	0	9365600	16435537	4789096	1012007	4045502	0.7211	2.6	Null

B8I7C9	RUMCH NAD-dependent epimerase/dehydratase	1792411	1082254	1792086	1862364	1898722	0	0.9145	1.2	Null
B8I7D0	RUMCH Putative competence-damage inducible protein	4012853	1324703	2917956	2732596	2020320	954982	0.6546	1.4	Null
B8I7D2	RUMCH Protein RecA	299697503	291652717	485392362	2126818696	229940056	91405082	0.5152	2.3	Null
B8I7D3	RUMCH Regulatory protein RecX	8965686	2137155	6706807	3950192	2685960	2229980	0.3643	2.0	Null
B8I7D4	RUMCH Ribosomal protein S12 methylthiotransferase RimO	1840811	1306671	2364802	2762922	2596716	241275	0.9953	1.0	Null
B8I7D6	RUMCH Regulatory protein DeoR	922467	1444792	1468747	568161	0	0	0.3596	6.8	Null
B8I7D9	RUMCH Filamentation induced by cAMP protein Fic	6924312	14875717	9961764	7633370	6889390	13296980	0.9969	1.1	Null
B8I7E4	RUMCH ABC transporter related	14650960	26705392	14100843	21496617	15616967	13892996	0.9769	1.1	Null
B8I7E9	RUMCH SMC domain protein	18502644	13415382	2738015	14925463	20482719	25013233	0.5545	1.7	Null
B8I7F0	RUMCH Uncharacterized protein	0	327757	1814518	1837532	1942299	2589196	0.4601	3.0	Null
B8I7F1	RUMCH Uncharacterized protein	0	414645	141608	94940	1333780	838859	0.3414	4.1	Null
B8I7F2	RUMCH Beta-lactamase domain-containing protein	208339	414126	0	172894	322473	753336	0.6693	2.0	Null
B8I7F7	RUMCH Uncharacterized protein	0	0	0	268699	0	0	NA	NA	Null
B8I7F9	RUMCH pPIWI	0	92420	0	89924	318345	284910	0.2855	7.5	Null
B8I7G1	RUMCH Uncharacterized protein	106353	1080348	1045244	4204804	4988672	3862305	0.0139	5.8	Increase
B8I7G2	RUMCH Restriction modification system DNA specificity domain protein	0	0	0	0	3156235	2817095	0.0058	NA	Increase
B8I7G5	RUMCH Site-specific DNA-methyltransferase (adenine-specific)	0	62919	0	0	73364	165348	0.6720	3.8	Null
B8I7G6	RUMCH Type I restriction enzyme R Protein	6325569	2979004	2738341	10173740	4091166	4605216	0.6035	1.6	Null
B8I7G9	RUMCH Resolvase domain protein	0	0	0	51528	0	0	NA	NA	Null
B8I7H3	RUMCH Uncharacterized protein	0	0	0	179615	67989	41569	0.1262	NA	Null
B8I7H4	RUMCH Uncharacterized protein	903178573	1352840911	1334504857	652361825	600420054	297727733	0.0464	2.3	Decrease
B8I7I0	RUMCH Cu	0	0	0	112594	152059	0	NA	NA	Null
B8I7I9	RUMCH Pirin domain protein	50210061	95157371	133184833	48293007	20612536	43229643	0.1900	2.5	Null
B8I7J0	RUMCH Zinc finger CDGSH-type domain protein	441573	0	0	624431	589122	4532675	0.2626	13.0	Null
B8I7J1	RUMCH Uncharacterized protein	0	517283	383254	2376001	4342515	826493	0.1141	8.4	Null
B8I7J2	RUMCH Uncharacterized protein	4621209	3142534	5252591	265030	246205	1552005	0.0759	6.3	Null
B8I7J7	RUMCH Uncharacterized protein	0	1549348	0	817430	1757042	715352	NA	2.1	Null
B8I7K5	RUMCH SAF domain protein	0	352986	0	0	69682	58068	0.8647	2.8	Null
B8I7L0	RUMCH S-layer domain protein	2243060	1231485	4308880	1484975	2039499	4573132	0.8804	1.0	Null
B8I7L1	RUMCH Uncharacterized protein	0	0	0	0	501535	0	0.2381	NA	Null
B8I7L6	RUMCH Uncharacterized protein	17685729689	22851928089	19098072423	10736226394	12781853529	10551725286	0.1511	1.8	Null
B8I7L7	RUMCH Transcriptional regulator, XRE family	0	1394222	3166883	729129	383138	371519	0.6002	3.1	Null
B8I7L9	RUMCH Uncharacterized protein	470351	0	453203	666510	595283	499788	0.7341	1.9	Null
B8I7M0	RUMCH Transcriptional regulator, XRE family	23966393	64506246	34361768	2946674	35271145	2066398	NA	3.0	Null
B8I7M1	RUMCH Uncharacterized protein	0	0	18448970	0	154570	6001363	NA	3.0	Null
B8I7M2	RUMCH Peptidase	3603647636	5262599329	4289147347	1595759769	1641241554	1690888481	0.0032	2.7	Decrease
B8I7M6	RUMCH Polymorphic outer membrane protein	630597	2283486	0	2053253	369144	715352	0.9585	1.1	Null
B8I7M7	RUMCH Uncharacterized protein	0	0	0	0	0	497200	0.2196	NA	Null
B8I7M9	RUMCH NAD-dependent epimerase/dehydratase	196026	0	0	417756	292587	0	0.7930	3.6	Null
B8I7N0	RUMCH Nucleotide sugar dehydrogenase	13567126	12141118	5721617	18308883	12544743	27716638	0.3300	1.9	Null
B8I7N1	RUMCH DNA polymerase beta domain protein region	1840412	7089282	11346696	7320576	5196572	2769918	0.8494	1.3	Null
B8I7N2	RUMCH N-acetyltransferase domain-containing protein	3601877	10253661	5725155	2438359	4777997	1785855	0.2891	2.2	Null
B8I7N3	RUMCH Methyltransferase type 11	3916613	2529620	3462414	1531277	3162461	2855079	0.8010	1.3	Null
B8I7N4	RUMCH DNA polymerase beta domain protein region	0	0	509122	0	535725	1688110	0.6148	4.4	Null
B8I7N5	RUMCH Uncharacterized protein	4333940	2946652	5349001	677450	874385	2734589	0.2681	2.9	Null
B8I7N6	RUMCH Uncharacterized protein	0	804545	0	0	0	0	NA	NA	Null
B8I7P1	RUMCH N-acetylmuramyl-L-alanine amidase, negative regulator of AmpC,	0	0	0	0	45055	0	NA	NA	Null
B8I7Q4	RUMCH Phosphate acyltransferase	18636372	12333452	28008538	20769801	15881695	10608332	0.7950	1.2	Null
B8I7Q5	RUMCH 3-oxoacyl-[acyl-carrier-protein] synthase 3	9454210	70274878	18992384	28757015	30783934	29868133	0.9730	1.1	Null
B8I7Q6	RUMCH Malonyl CoA-acyl carrier protein transacylase	58567322	106276787	131783154	187414586	48617448	128448406	0.7197	1.2	Null
B8I7Q7	RUMCH 3-oxoacyl-[acyl-carrier-protein] reductase	4659582	3960665	2204957	1421087	1231642	1317875	0.0911	2.7	Null
B8I7Q9	RUMCH 3-oxoacyl-[acyl-carrier-protein] synthase 2	26600901	61688847	48827995	33505829	8786136	15876031	0.2407	2.4	Null
B8I7R0	RUMCH Ribonuclease 3	74610050	17175262	19757720	17278155	19019788	12173650	0.3041	2.3	Null
B8I7R1	RUMCH Radical SAM domain protein	0	0	0	430222	353158	1240788	0.0001	NA	Increase
B8I7R2	RUMCH Stage V sporulation protein S	37639794	23770093	146597551	107739852	158384579	12363063	0.8112	1.3	Null
B8I7R3	RUMCH Superoxide dismutase	0	0	0	86419	0	0	NA	NA	Null
B8I7R4	RUMCH Coat F domain protein	769067	3431868	0	2463012	782370	2500995	0.8494	1.4	Null
B8I7R5	RUMCH Uncharacterized protein	0	2426893	3119716	2690081	0	1525663	0.9692	1.3	Null
B8I7R6	RUMCH TM1586	293217	0	0	0	75438	0	0.7580	3.9	Null
B8I7R7	RUMCH Aldo/keto reductase	534347	12103368	1508505	1703126	3050405	3971768	0.8486	1.6	Null
B8I7R8	RUMCH Chromosome partition protein Smc	9430165	14809479	7231835	11128059	23437173	9001303	0.5815	1.4	Null
B8I7R9	RUMCH Signal recognition particle receptor FtsY	36392138	61063784	50383989	115353645	73993305	114432350	0.0480	2.1	Increase
B8I7S0	RUMCH Uncharacterized protein	358824499	164977816	30272896	265812574	492411396	121413482	0.7580	1.6	Null
B8I7S1	RUMCH Amino acid transporter-like protein	5096396	3119931	9369716	9226098	2718769	4421378	0.9765	1.1	Null
B8I7S2	RUMCH ROK family protein	18048165	3607162	1223332	5261080	4616538	5982740	0.7723	1.4	Null
B8I7S3	RUMCH PC4 domain-containing protein	12934132	17936945	17134067	16681991	14006962	15303633	0.9404	1.0	Null
B8I7S4	RUMCH Anaerobic ribonucleoside-triphosphate reductase	10276327	214855506	28829130	10543773	16174391	8230353	0.0751	7.3	Null
B8I7S5	RUMCH Anaerobic ribonucleoside-triphosphate reductase-activating prote	7648660	8096997	15015675	5830020	4184003	3355192	0.0914	2.3	Null
B8I7S6	RUMCH Sulfatase	7309490	6875022	11264235	3713136	3479952	3421216	0.0388	2.4	Decrease
B8I7S7	RUMCH Pantothenate kinase	0	0	0	1071933	791321	604654	0.0000	NA	Increase
B8I7S9	RUMCH HTHT-type domain-containing protein	15742882	8924531	2983869	1794598	7762033	18952409	0.9463	1.0	Null
B8I7T0	RUMCH UPF0122 protein Ccel	0	186693	449753	4551152	216957	1697886	0.1268	10.2	Null
B8I7T1	RUMCH Signal recognition particle protein	131682586	106211171	99886397	202447742	167301830	123263428	0.3646	1.5	Null
B8I7T2	RUMCH 30S ribosomal protein S16	886926277	425411546	140910126	393892530	214961381	25290340	0.4322	2.3	Null
B8I7T3	RUMCH UPF0109 protein Ccel	117107339	346504	67152784	877380	89330287	1236519	0.7570	2.0	Null
B8I7T4	RUMCH Ribosome maturation factor RimM	25683850	28569875	99466626	88484463	49141366	80304350	0.5750	1.4	Null
B8I7T5	RUMCH tRNA (guanine-N(1)-)-methyltransferase	0	71384	591610	270045	204039	192813	0.9573	1.0	Null
B8I7T6	RUMCH 50S ribosomal protein L19	353921450	501043852	767338364	159823983	280451700	242863730	0.1498	2.4	Null
B8I7T7	RUMCH Signal peptidase I	24121264	19433384	15317641	67088642	29019188	31612802	0.1725	2.2	Null
B8I7T8	RUMCH Ribosome biogenesis GTPase A	5102658	3568413	5812829	6498237	3699216	4259660	0.9584	1.0	Null
B8I7T9	RUMCH Ribonuclease HII	3091013	2393799	5431449	403713	1849436	2215693	0.3979	2.4	Null
B8I7U0	RUMCH Uncharacterized protein	3046803	3840609	611436	7361908	3683883	4229144	0.4014	2.0	Null
B8I7U2	RUMCH UPF0102 protein Ccel	1145610	0	0	0	0	0	NA	NA	Null
B8I7U3	RUMCH Peptidase M19 renal dipeptidase	0	111355	0	145108	238852	311879	0.3183	6.2	Null
B8I7U4	RUMCH Putative transcriptional regulator, GntR family	45796281	25683337	13527739	26109430	19604785	19399184	0.7638	1.3	Null
B8I7U5	RUMCH Adenylosuccinate lyase	109904251	138358150	49183155	136272571	178914588	95495874	0.6089	1.4	Null
B8I7U6	RUMCH Transcriptional regulator, GntR family	3915006	0	0	0	0	0	NA	NA	Null
B8I7U7	RUMCH Beta-lactamase class A-like protein	15195320	16296280	27062494	17534543	78881046	68700861	0.1280	2.8	Null
B8I7V0	RUMCH Cellulosome anchoring protein cohesin region	311617302703	499007114138	471139997605	187109087322	275099047889	270483315974	0.3041	1.7	Null
B8I7V4	RUMCH Glucanase	510666320555	588975390915	386273797032	373796196062	279190998416	245453689682	0.2787	1.7	Null
B8I7V5	RUMCH Cellulosome anchoring protein cohesin region	18995279274	34846663811	26753380901	14334512737	15735949028	21320667602	0.5207	1.6	Null
B8I7V6	RUMCH Glucanase	3044324576	2866158305	1825435863	3607223734	2338416415	1891118276	0.9654	1.0	Null
B8I7V7	RUMCH Cellulase	649886924	1238834971	248742860	1059890579	1028210121	558620529	0.7840	1.2	Null
B8I7V8	RUMCH Cellulase	43334444	44803837	17591541	41181009	62305744	64773711	0.4527	1.6	Null
B8I7V9	RUMCH Glucanase	504739446	910041265	247982405	313982384	362723226	533591352	0.8151	1.4	Null
B8I7W0	RUMCH Cellulase	135147624	117234277	61872939	77682445	88950009	44224306	0.5648	1.5	Null
B8I7W1	RUMCH Cellulase	776969498	189697640	116499500	155532221	271929258	198371775	0.5919	1.7	Null
B8I7W7	RUMCH Copper-exporting P-type ATPase	4136943	3895296	570262	2332093	2128487	1668592	0.7765	1.4	Null
B8I7W8	RUMCH NLP/P60 protein	24260426	18804764	31519554	10486380	9132971	6598164	0.0100	2.8	Decrease
B8I7X1	RUMCH Endo-1,4-beta-xylanase	9224880576	12162343147	25555324807	35294952191	19830721806	13073677835	0.5152	1.5	Null
B8I7X2	RUMCH PpiC-type peptidyl-prolyl cis-trans isomerase	2113296583	2007757334	2648939496	1146121347	961346102	693659056	0.0102	2.4	Decrease
B8I7X6	RUMCH Cellulase	2573044529	2591349934	3535056580	2253561659	3403474461	2790644966	0.9393	1.0	Null
B8I7X7	RUMCH Glutamine-rich	0	0	0	182691	0	0	NA	NA	Null
B8I7X8	RUMCH 30S ribosomal protein S10	132217797	918319505	1356373291	310111437	380580541	481347604	0.5648	2.1	Null
B8I7X9	RUMCH 50S ribosomal protein L3	9649254273	11079372987	5956200651	3523050375	1966231601	2159727086	0.0085	3.5	Decrease
B8I7Y0	RUMCH 50S ribosomal protein L4	288904175	363260604	284264623	205060886	66819652	44842477	0.1009	3.0	Null
B8I7Y1	RUMCH 50S ribosomal protein L23	753677446	568089373	902128136	522763322	265968238	565303408	0.4972	1.6	Null
B8I7Y2	RUMCH 50S ribosomal protein L2	9729530515	11799974527	10320483583	3311211942	2989015298	3049823367	0.0000	3.4	Decrease
B8I7Y3	RUMCH 30S ribosomal protein S19	807598139	508016165	668140582	67534749	119486589	11515803	0.0026	10.0	Decrease
B8I7Y4	RUMCH 50S ribosomal protein L22	1735428393	214148696	1269494033	163999934	423834112	315234681	0.1974	3.6	Null
B8I7Y5	RUMCH 30S ribosomal protein S3	316088347	437815352	469820175	750250223	158076575	129639289	0.8813	1.2	Null
B8I7Y6	RUMCH 50S ribosomal protein L16	112320295	179988408	328441188	42936731	39758283	41862892	0.0008	5.0	Decrease

B8I7Y7	RUMCH 50S ribosomal protein L29	139459417	166615744	145376686	1700477	2001566	1861577	0.0000	81.1	Decrease
B8I7Y8	RUMCH 30S ribosomal protein S17	321660	21600904	19593219	8226163	4935799	2665740	0.5267	2.6	Null
B8I7Y9	RUMCH 50S ribosomal protein L14	418083802	1186184027	599901174	196856845	263807917	184459963	0.0193	3.4	Decrease
B8I7Z0	RUMCH 50S ribosomal protein L24	376574852	167239430	139847720	7457437	7176456	5696788	0.0000	33.6	Decrease
B8I7Z1	RUMCH 50S ribosomal protein L5	5209319387	7151694897	10487298751	2905015780	2980230289	2961177169	0.0227	2.6	Decrease
B8I7Z2	RUMCH 30S ribosomal protein S14 type Z	43699236	112803256	100407925	3702755	9210505	222642	0.0031	19.6	Decrease
B8I7Z3	RUMCH 30S ribosomal protein S8	2432340171	3879193511	3808377007	1090406002	876605653	1694607498	0.0771	2.8	Null
B8I7Z4	RUMCH 50S ribosomal protein L6	22807489795	18175787360	33911534053	4270834037	3210720373	3537033187	0.0000	6.8	Decrease
B8I7Z5	RUMCH 50S ribosomal protein L18	501969	7898637	4928934	763243	849657	1130277	0.1423	4.9	Null
B8I7Z6	RUMCH 30S ribosomal protein S5	22000557369	22396523772	33329530642	10297085366	9174073053	10798303303	0.0201	2.6	Decrease
B8I7Z7	RUMCH 50S ribosomal protein L30	4713762	7210846	6433423	1959053	1698610	2615678	0.0252	2.9	Decrease
B8I7Z8	RUMCH 50S ribosomal protein L15	3124909879	13771217977	2879243919	918223329	4282274452	3574672588	0.5244	2.3	Null
B8I800	RUMCH Adenylate kinase	2791013050	3327776364	4513479901	2087441796	1691954052	2001851438	0.1699	1.8	Null
B8I801	RUMCH Methionine aminopeptidase	186741158	132600204	158364768	68853442	44897480	74556594	0.0854	2.5	Null
B8I802	RUMCH Uncharacterized protein	4429549	34079948	83262979	17035244	2186484	13285495	0.3016	3.7	Null
B8I803	RUMCH Translation initiation factor IF-1	130254755	145558158	279128280	94713458	60117836	10709245	0.1485	3.4	Null
B8I804	RUMCH 50S ribosomal protein L36	893308	0	0	0	0	0	NA	NA	Null
B8I805	RUMCH 30S ribosomal protein S13	678245082	3057996171	1320912294	154418312	98344595	380907250	0.0147	8.0	Decrease
B8I806	RUMCH 30S ribosomal protein S11	202607503	115622875	27027981	109323167	90034815	58946651	0.7805	1.3	Null
B8I807	RUMCH 30S ribosomal protein S4	16204476100	21838423209	6852196520	4041157619	3139398406	2857435721	0.0078	4.5	Decrease
B8I808	RUMCH DNA-directed RNA polymerase subunit alpha	115097494	101079566	95668851	327242764	127525964	167773457	0.1939	2.0	Null
B8I809	RUMCH 50S ribosomal protein L17	3663937905	1435405345	3650769870	255682986	369601160	125196706	0.0000	11.7	Decrease
B8I810	RUMCH Energy-coupling factor transporter ATP-binding protein EcfA	2225685	3983704	341775	5272559	699872	696361	0.9993	1.0	Null
B8I811	RUMCH Methyltransferase small	250036	783441	2803877	1658143	943043	457671	0.9218	1.3	Null
B8I812	RUMCH Energy-coupling factor transporter ATP-binding protein EcfA	10871025	22997474	23444554	41555638	78220836	27774467	0.0627	2.6	Null
B8I814	RUMCH tRNA pseudouridine synthase A	0	93919	0	0	0	91802	NA	1.0	Null
B8I815	RUMCH 50S ribosomal protein L13	189633477	3168312744	303221918	149785270	134851410	950813521	NA	3.0	Null
B8I816	RUMCH 30S ribosomal protein S9	1094223278	842698965	3651731596	538732815	187969464	343912010	0.0299	5.2	Decrease
B8I818	RUMCH Iron dependent repressor	0	140664	0	233146	108916	143346	0.5796	3.5	Null
B8I819	RUMCH Amidohydrolase	4035580	8358074	24446173	25332554	20880711	9240522	0.5811	1.5	Null
B8I820	RUMCH t(6)A37 threonylcarbamoyladenosine biosynthesis protein TsaE	0	250669	538620	435995	0	618311	0.8724	1.3	Null
B8I821	RUMCH Peptidase M22 glycoprotease	0	93223	101378	12247146	189178	6298700	0.0004	96.3	Increase
B8I823	RUMCH Endopeptidase La	22171385	52974226	114915982	20173827	19207380	26264654	0.1968	2.9	Null
B8I824	RUMCH RNA polymerase, sigma-24 subunit, ECF subfamily	0	269277	0	0	0	0	NA	NA	Null
B8I828	RUMCH Phage major capsid protein, HK97 family	0	0	0	94077	4184802	295139	0.0004	NA	Increase
B8I834	RUMCH Uncharacterized protein	0	1731919	0	0	0	0	NA	NA	Null
B8I846	RUMCH AntA/AntB antirepressor domain protein	0	267984	246853	137365	144414	0	0.8629	1.8	Null
B8I849	RUMCH Transcriptional regulator, TetR family	0	0	0	151976	107107	0	NA	NA	Null
B8I852	RUMCH Uncharacterized protein	4647867	2590494	10687349	3845793	2395499	1145882	0.2741	2.4	Null
B8I854	RUMCH rRNA biogenesis protein rrp5, putative	779845	948295	1415795	191834	295063	0	0.1791	6.5	Null
B8I855	RUMCH Uncharacterized protein	0	1578012	303371	0	798999	797649	0.9864	1.2	Null
B8I858	RUMCH HTH cro/C1-type domain-containing protein	954504	681080	408934	1814924	1090095	1607960	0.2359	2.2	Null
B8I859	RUMCH Transcriptional regulator, XRE family	0	1367885	0	0	0	0	NA	NA	Null
B8I860	RUMCH Restriction endonuclease MspI	2213425	419972	5957379	14050975	12789303	9187488	0.1093	4.2	Null
B8I861	RUMCH Cytosine-specific methyltransferase	285321	176267	192432	183193	0	0	0.5717	3.6	Null
B8I862	RUMCH RNA methyltransferase, TrmA family	0	226498	123174	2178773	4331932	554168	0.0120	20.2	Increase
B8I866	RUMCH Uncharacterized protein	3550261	1999772	636018	1717681	1435996	1088915	0.6994	1.5	Null
B8I868	RUMCH Methyl-accepting chemotaxis sensory transducer	23257744	9527454	8895053	10640946	20882629	12501932	0.9585	1.1	Null
B8I870	RUMCH DD-transpeptidase	1121755800	1064339402	2026022071	276526259	556029007	542719730	0.0758	3.1	Null
B8I871	RUMCH Glutamate 5-kinase	6505055	6328751	8371613	14052508	15062461	7048399	0.2175	1.7	Null
B8I872	RUMCH Uncharacterized protein	0	0	0	0	26193	0	NA	NA	Null
B8I873	RUMCH Elongation factor G	103409328	324007439	445273271	286306909	176592185	251635165	0.9320	1.2	Null
B8I874	RUMCH Phospho-2-dehydro-3-deoxyheptonate aldolase	1421048336	1840204209	1222395890	751804498	666621120	1223495486	0.4650	1.7	Null
B8I875	RUMCH Prephenate dehydrogenase	1647288	4424182	2864351	5825680	9458274	6549933	0.0469	2.4	Increase
B8I876	RUMCH Redox-sensing transcriptional repressor Rex	0	0	0	98274	0	0	NA	NA	Null
B8I877	RUMCH Polysaccharide pyruvyl transferase	267473	1805772	198679	3244936	2053276	3457031	0.1270	3.9	Null
B8I878	RUMCH ABC transporter related	565774	2880868	0	5631976	1759685	3174625	0.5069	3.1	Null
B8I879	RUMCH Beta-ketoacyl-[acyl-carrier-protein] synthase III	0	0	0	499369	559939	0	0.0991	NA	Null
B8I880	RUMCH Methyl-accepting chemotaxis sensory transducer	794172746	937516536	565701687	815073493	1073200885	256909856	0.9585	1.1	Null
B8I882	RUMCH Methyl-accepting chemotaxis sensory transducer	1209851	21278321	0	8219233	127834	50644412	0.6481	2.6	Null
B8I884	RUMCH Cell wall hydrolase/autolysin	34271225	141304641	134762457	65277697	60798100	43571383	0.4501	1.8	Null
B8I885	RUMCH Uncharacterized protein	1124204	1870883	1279166	692560	52279	520968	0.2669	3.4	Null
B8I887	RUMCH Chitinase II	253695367	367246247	466077366	239300357	328997885	298204358	0.8225	1.3	Null
B8I894	RUMCH 33 kDa chaperonin	0	9164724	5521303	7597347	395710	6798597	0.9490	1.0	Null
B8I895	RUMCH Cold-shock DNA-binding domain protein	21381487	28252005	39970552	13637243	38694870	118084612	0.4060	1.9	Null
B8I898	RUMCH Carboxyl-terminal protease	3192779	4673000	6478657	15197066	12682250	5973273	0.0583	2.4	Null
B8I899	RUMCH Peptidase M23	76103333	130806620	103950500	38309127	6646654	33752407	0.0908	3.9	Null
B8I8A0	RUMCH Cell division protein FtsX	0	418014	282729	236860	294078	0	0.9562	1.3	Null
B8I8A1	RUMCH Cell division ATP-binding protein FtsE	597375	5657180	3300774	5384016	6989879	2939799	0.5337	1.6	Null
B8I8A2	RUMCH Transcriptional regulator, CdaR	10788689	1271878	4150569	6433928	6671686	6429761	0.8990	1.2	Null
B8I8A3	RUMCH ABC transporter related	12435566647	16887201544	14258888742	14687203884	16189455687	20218418449	0.6054	1.2	Null
B8I8A4	RUMCH N-acetyl-gamma-glutamyl-phosphate reductase	0	1974608	0	280225	1532674	0	0.9953	1.1	Null
B8I8A5	RUMCH Acetylglutamate kinase	37795943	32839787	28903134	44091743	31832909	19230364	0.9688	1.0	Null
B8I8A6	RUMCH Acetylornithine aminotransferase	42807315	10158117	5595175	10274192	17250201	14456047	0.7765	1.4	Null
B8I8A7	RUMCH Carbamoyl-phosphate synthase small chain	14851660	1359244	19705834	7972773	7061646	3937603	0.5759	1.9	Null
B8I8A8	RUMCH Carbamoyl-phosphate synthase large chain	199572510	19879160	17093126	23800569	15461203	10595764	NA	4.7	Null
B8I8A9	RUMCH Ornithine carbamoyltransferase	4491538	8082363	10713067	5144995	5836670	5635173	0.6757	1.4	Null
B8I8B0	RUMCH GCN5-related N-acetyltransferase	0	0	0	0	0	110527	NA	NA	Null
B8I8B1	RUMCH DUF3794 domain-containing protein	249332	478696	203418	216776	233294	235973	0.8308	1.4	Null
B8I8B2	RUMCH Anaerobic carbon-monoxide dehydrogenase	154940	75945	609538	1821977	880682	326824	0.2199	3.6	Null
B8I8B3	RUMCH Cupin	4294541	978645	0	0	1165229	0	0.6590	4.5	Null
B8I8B5	RUMCH N-acetylglucosaminyldiphosphoundecaprenol N-acetyl-beta-D-ma	3685437	593896	1613762	2027907	5867722	3044008	0.5732	1.9	Null
B8I8B6	RUMCH Uncharacterized protein	0	1691816	974074	2025385	404432	967493	0.8648	1.3	Null
B8I8B7	RUMCH Uncharacterized protein	202976509	1244890594	815264009	320195472	261794863	516394319	0.5099	2.1	Null
B8I8B8	RUMCH mRNA interferase	1268740	2933508	7110371	5082565	5998427	2308860	0.7964	1.2	Null
B8I8B9	RUMCH Putative transcriptional regulator, CopG family	393284	0	0	0	0	0	0.2575	NA	Null
B8I8C0	RUMCH Alanine racemase	0	279598	0	454293	1053787	296243	0.3392	6.5	Null
B8I8C1	RUMCH Bifunctional NAD(P)H-hydrate repair enzyme	0	0	0	140108	0	0	NA	NA	Null
B8I8C2	RUMCH Uncharacterized protein	890449	372733	1914249	549551	1380983	429779	0.8463	1.3	Null
B8I8C3	RUMCH Nucleotide sugar dehydrogenase	67804093	97027696	116387012	330795047	290760301	252139479	0.0000	3.1	Increase
B8I8C4	RUMCH TPR/glycosyl transferase domain-containing protein	42445909	103239321	17231203	32726515	18295500	44284596	0.6665	1.7	Null
B8I8C5	RUMCH Uncharacterized protein	16445877	11770801	1661660	5071846	2335197	1169550	0.2083	3.5	Null
B8I8D0	RUMCH PMT	0	0	114859	331888	344267	235425	0.2609	7.9	Null
B8I8D1	RUMCH NAD(+) diphosphatase	1436768	0	589353	0	0	0	0.0396	NA	Decrease
B8I8D2	RUMCH Heat shock protein Hsp20	81569747	146668676	101489878	237107155	414888285	409354889	0.0053	3.2	Increase
B8I8D5	RUMCH Cell wall hydrolase/autolysin	422760	0	86928	0	83800	495037	0.9605	1.1	Null
B8I8D6	RUMCH tRNA-dihydrouridine synthase	136385	484114	0	156345	59694	66845	0.7534	2.2	Null
B8I8D9	RUMCH Transcriptional modulator of MazE/toxin, MazF	4734908	8380058	10150134	3178435	28985074	1336187	0.7683	1.4	Null
B8I8E0	RUMCH Uncharacterized protein	0	650590	268482	0	0	0	0.1455	NA	Null
B8I8F0	RUMCH Uncharacterized protein	0	361412	483814	2178410	2089807	898938	0.1671	6.1	Null
B8I8F1	RUMCH Phosphocarrier, HPr family	226055235	130916511	87199960	15179900	1931900	7060865	0.0001	18.4	Decrease
B8I8F2	RUMCH UvrABC system protein C	20875222	22517811	5380991	969408	2404758	7805392	0.2109	4.4	Null
B8I8F4	RUMCH Trigger factor	4122217120	9839670468	7906462551	5896457956	3431476943	3037708936	0.3035	1.8	Null
B8I8F5	RUMCH ATP-dependent Clp protease proteolytic subunit	365854484	742935678	1535588670	885221252	963677904	602698910	0.9782	1.1	Null
B8I8F6	RUMCH ATP-dependent Clp protease ATP-binding subunit ClpX	12642056	36655291	13477262	45816498	42744804	51548896	0.1215	2.2	Null
B8I8F9	RUMCH tRNA N6-adenosine threonylcarbamoyltransferase	8808341	6999756	6553649	27873761	15055846	10753931	0.0935	2.4	Null
B8I8G0	RUMCH Lytic transglycosylase catalytic	1501084	95778	1098993	1608469	1953348	3146284	0.4007	2.5	Null
B8I8G2	RUMCH DUF402 domain-containing protein	1300808	1205877	1931856	7002489	4268025	4015644	0.0013	3.4	Increase
B8I8G3	RUMCH PHP domain protein	93026416	75448958	583872677	69572322	16367021	7102455	0.0480	8.1	Decrease
B8I8G4	RUMCH SsrA-binding protein	0	0	103158	0	57716	0	NA	1.8	Null
B8I8G5	RUMCH Integrase family protein	346451	0	0	0	0	0	0.2673	NA	Null
B8I8G6	RUMCH Ribose ABC transporter permease component	5440501	13302210	21785119	0	0	2886784	0.3084	14.0	Null

B8I8G7	RUMCH Uncharacterized protein	52971448	120146275	92632254	49455490	102244186	46663683	0.7821	1.3	Null
B8I8H1	RUMCH Uncharacterized protein	7846443	9538086	11399899	5498496	3162530	3277803	0.0262	2.4	Decrease
B8I8H4	RUMCH SH3 type 3 domain protein	41321778	407828799	27922355	3951537	4693220	19561404	0.0194	16.9	Decrease
B8I8H9	RUMCH Uncharacterized protein	169329797064	182523923482	142188462978	116608526270	54634361387	99337930515	0.3000	1.8	Null
B8I8I6	RUMCH Uncharacterized protein	0	0	0	747574	437958	269626	0.0001	NA	Increase
B8I8I8	RUMCH Uncharacterized protein	43674807	25983732	34053872	36932364	34598670	18679873	0.8564	1.1	Null
B8I8I9	RUMCH Uncharacterized protein	6026805	626073	469115	1613425	3423571	598742	0.8438	1.3	Null
B8I8J3	RUMCH [Acyl-carrier-protein] S-malonyltransferase	30351083	32756529	24102545	33237478	15267390	3656510	0.5643	1.7	Null
B8I8J4	RUMCH ABC transporter related	196544072	1150275807	228972860	765319792	788078599	905767429	0.5207	1.6	Null
B8I8J5	RUMCH FtsX domain-containing protein	55554024	98517347	187574164	90341854	77141665	70857088	0.6935	1.4	Null
B8I8J6	RUMCH FtsX domain-containing protein	52418813	323626187	101114965	97846189	93052895	73560349	0.5747	1.8	Null
B8I8J7	RUMCH Beta-ketoacyl synthase	11263009	3822341	30123061	12683318	2944547	1942875	0.3709	2.6	Null
B8I8J8	RUMCH Beta-ketoacyl synthase	841336	9922920	210951	387980	3759689	19349046	0.5747	2.1	Null
B8I8J9	RUMCH Beta-ketoacyl synthase	65465994	2506802272	2003980030	1138052928	2027700250	324790321	0.9255	1.3	Null
B8I8K0	RUMCH Beta-ketoacyl synthase	243985013	403128348	21193983	409261113	127161685	44550330	0.9223	1.2	Null
B8I8K1	RUMCH Beta-ketoacyl synthase	18815619546	14307165943	46444713735	43766535405	11264632509	9125370571	0.8636	1.2	Null
B8I8K2	RUMCH Beta-ketoacyl synthase	362101	1771971	0	198745	0	0	0.4403	10.7	Null
B8I8K3	RUMCH Amino acid adenylation domain protein	2933029	542340	0	226214	597203	350102	0.5747	3.0	Null
B8I8K4	RUMCH Acyl carrier protein	0	3463838	1470169	4875460	4225113	624162	0.7084	2.0	Null
B8I8K5	RUMCH Beta-ketoacyl synthase	0	268466	0	578849	438156	306300	0.4280	4.9	Null
B8I8K6	RUMCH Beta-ketoacyl synthase	0	799927	678249	157761	341711	187060	0.7723	2.2	Null
B8I8K7	RUMCH Beta-ketoacyl synthase	724662	0	731544	1481614	1643484	561933	0.6111	2.5	Null
B8I8K8	RUMCH PKS	0	389151	0	0	0	163519	0.9045	2.4	Null
B8I8L0	RUMCH Uncharacterized protein	0	111515	0	152394	150678	274962	0.3984	5.2	Null
B8I8L1	RUMCH Short-chain dehydrogenase/reductase SDR	0	974442	0	0	2224	0	NA	438.1	Null
B8I8L2	RUMCH Radical SAM domain protein	0	829363	674889	1623255	2549424	2176897	0.2787	4.2	Null
B8I8L3	RUMCH 3-Oxoacyl-(Acyl-carrier-protein (ACP)) synthase III domain protein	0	2459987	0	988938	1935047	2424601	0.7379	2.2	Null
B8I8L4	RUMCH Coenzyme F390 synthetase-like protein	123885	114720	0	526713	507853	480102	0.1595	6.3	Null
B8I8L7	RUMCH Uncharacterized protein	14546349	717809	1225362	227609	562398	1088855	NA	8.8	Null
B8I8M0	RUMCH Carbohydrate-binding, CenC-like protein	26754277	30623980	7207271	56698757	97282136	52029020	0.1058	3.2	Null
B8I8M2	RUMCH Polysaccharide deacetylase	0	0	0	203959	65863	1109523	0.0037	NA	Increase
B8I8M3	RUMCH Peptidase M23	886722	176449	285066	274567	384424	109590	0.5941	1.8	Null
B8I8M4	RUMCH Extracellular solute-binding protein family 3	28103546	66055098	38850067	26613742	21657372	26081107	0.3084	1.8	Null
B8I8M6	RUMCH ABC transporter related	0	0	0	1161400	940023	0	0.0358	NA	Increase
B8I8M7	RUMCH Transcriptional regulator, XRE family	1392672	1829950	1023321	0	0	0	0.0000	NA	Decrease
B8I8M8	RUMCH Spermidine/putrescine import ATP-binding protein PotA	5376122	4248806	4710538	22044258	10946250	9088634	0.0241	2.9	Increase
B8I8N1	RUMCH Extracellular solute-binding protein family 1	2996451	1221574	1739812	2883117	3876063	1266110	0.7650	1.3	Null
B8I8N2	RUMCH Rubrerythrin	73923826	49361216	89962845	29903985	47543449	22082412	0.1959	2.1	Null
B8I8N3	RUMCH Iron-containing alcohol dehydrogenase	3331986475	11305396749	9726315195	11816638500	10156977754	10275437640	0.5079	1.3	Null
B8I8N4	RUMCH Uncharacterized protein	109392809	211609820	168142968	249327967	254630273	260525623	0.1616	1.6	Null
B8I8N5	RUMCH AMP-dependent synthetase and ligase	5317717	3961316	207827	1345360	3844569	88212168	NA	9.8	Null
B8I8N7	RUMCH Probable GTP-binding protein EngB	6776395	2404830	7804038	12771678	11121437	11708026	0.2500	2.1	Null
B8I8N8	RUMCH Uncharacterized protein	0	339993	874690	8134371	539191	1790024	0.1758	8.6	Null
B8I8P0	RUMCH Copper amine oxidase domain protein	49058208	11720483	72687797	92705537	190760867	51701025	0.2925	2.5	Null
B8I8P1	RUMCH Rubrerythrin	554812640	2929847177	2582918465	316904793	263508858	1243016580	0.3035	3.3	Null
B8I8P5	RUMCH DUF362 domain-containing protein	33287563	57391597	31684344	9106351	3092840	2673464	0.0001	8.2	Decrease
B8I8P8	RUMCH Amidohydrolase 3	570713	846047	684082	827717	1525203	599707	0.5466	1.4	Null
B8I8P9	RUMCH Short-chain dehydrogenase/reductase SDR	442437139	521408005	273371286	612007619	474161318	53159252	0.9438	1.1	Null
B8I8Q5	RUMCH Cellulase	2030447106	739655447	622738568	4003275384	4849362487	4220854673	0.0583	3.9	Null
B8I8Q6	RUMCH DNA topoisomerase 3	13788938	49560369	20465289	11244802	3624599	5923199	0.0500	4.0	Decrease
B8I8Q7	RUMCH ExsB family protein	20306348	1609197	9636135	3486753	5864248	2834660	0.3361	2.6	Null
B8I8Q8	RUMCH Transcriptional regulator, BadM/Rrf2 family	1002870	0	956386	1796147	2228663	1785216	0.5218	3.0	Null
B8I8Q9	RUMCH Uncharacterized protein	672572	1942362	5109375	4954588	6069483	7157641	0.2316	2.4	Null
B8I8R1	RUMCH Uncharacterized protein	169385	0	0	266607	145007	0	0.8698	2.4	Null
B8I8R3	RUMCH Alkaline phosphatase	1444593	1451810	2674811	1722029	1161868	1245413	0.7121	1.3	Null
B8I8R5	RUMCH Carbamoyl-phosphate synthase L chain ATP-binding	16515369	3174476	9108478	23072212	12626866	10193113	0.6598	1.6	Null
B8I8R6	RUMCH Biotin carboxyl carrier protein of acetyl-CoA carboxylase	4017746	8246656	18934126	7736217	3397745	6429004	0.5802	1.8	Null
B8I8S6	RUMCH Uncharacterized protein	0	0	0	73991	0	55825	NA	NA	Null
B8I8S8	RUMCH Uncharacterized protein	3480367	2768864	1970462	2565924	1830243	2038438	0.7596	1.3	Null
B8I8T6	RUMCH Uncharacterized protein	9046485	2846084	681515	8687029	3737184	5120532	0.8473	1.4	Null
B8I8T7	RUMCH Uncharacterized protein	6785689	8322973	8099233	5477109	2587246	7702524	0.7121	1.5	Null
B8I8U0	RUMCH Uncharacterized protein	248241	0	0	0	0	0	0.3005	NA	Null
B8I8U2	RUMCH Uncharacterized protein	509864	0	1543900	0	112893	0	0.3084	18.2	Null
B8I8U8	RUMCH Stage II sporulation protein E, protein serine/threonine phosphata	0	0	0	3410195	409119	343834	0.0000	NA	Increase
B8I8U9	RUMCH Nicotinate phosphoribosyltransferase	0	0	0	0	0	103360	NA	NA	Null
B8I8V2	RUMCH Peptidase S1 and S6 chymotrypsin/Hap	59793038	53713831	21060413	7174069	5768970	10022544	0.0055	5.9	Decrease
B8I8V4	RUMCH Uncharacterized protein	0	0	0	332094	331268	169179	0.0024	NA	Increase
B8I8V5	RUMCH LUD	42704860	19265121	55819382	3353750	3159359	5610453	0.0002	9.7	Decrease
B8I8V6	RUMCH UPF0178 protein Ccel	2159773	8161815	11341833	2496749	9934516	2363159	0.8209	1.5	Null
B8I8V7	RUMCH Binding-protein-dependent transport systems inner membrane co	823047	681743	0	1355898	899654	508337	0.7765	1.8	Null
B8I8V8	RUMCH Binding-protein-dependent transport systems inner membrane co	0	0	0	0	411802	0	0.2595	NA	Null
B8I8V9	RUMCH Extracellular solute-binding protein family 1	24426226289	52499442025	33490105977	11690234415	9158695356	21996221268	0.2112	2.6	Null
B8I8W1	RUMCH Transcriptional regulator, LacI family	6729307	7900721	6175044	9689266	11824922	10211059	0.2268	1.5	Null
B8I8W3	RUMCH Glycosidase PH1107-related	1621890	1676762	5453893	6034059	11130866	6678732	0.1103	2.7	Null
B8I8W4	RUMCH Cellobiose 2-epimerase	5003731	983378	0	0	759434	848294	0.6405	3.7	Null
B8I8W5	RUMCH 4-O-beta-D-mannosyl-D-glucose phosphorylase	830059	5787195	6796415	24524147	16696750	12596826	0.0446	4.0	Increase
B8I8W8	RUMCH Cobalamin synthesis protein P47K	0	385960	0	758476	493817	525431	0.4542	4.6	Null
B8I8X0	RUMCH GCN5-related N-acetyltransferase	0	227178	426097	1377530	853817	854588	0.2404	4.7	Null
B8I8X1	RUMCH Transcriptional regulator, CarD family	624694	2399306	3613003	1640805	3365570	1020797	0.9943	1.1	Null
B8I8X2	RUMCH Uncharacterized protein	0	0	180792	201571	280154	0	0.7964	2.7	Null
B8I8X9	RUMCH S-layer domain protein	162380775	238368808	228441689	100212145	84942639	225612508	0.7095	1.5	Null
B8I8Y0	RUMCH Uncharacterized protein	6720904	5547881	4068766	66324684	54273638	235410384	0.0000	21.8	Increase
B8I8Y3	RUMCH Uncharacterized protein	40173635	40988792	34316124	87017069	79364938	104450146	0.0235	2.3	Increase
B8I8Y5	RUMCH Peptidase M15B and M15C DD-carboxypeptidase VanY/endolysin	16703337	59635378	157665494	3521407	3599984	1728206	0.0000	26.4	Decrease
B8I8Y8	RUMCH Uncharacterized protein	0	294471	174518	169886	299810	260347	0.7418	1.6	Null
B8I8Y9	RUMCH Uncharacterized protein	0	0	0	1225022	0	0	NA	NA	Null
B8I8Z0	RUMCH Uncharacterized protein	0	96096	192945	214919	239920	182236	0.5815	2.2	Null
B8I8Z1	RUMCH Uncharacterized protein	2917010	2152169	2325809	902212	753263	854624	0.0252	2.9	Decrease
B8I8Z2	RUMCH Uncharacterized protein	619576702	812002474	629722600	285431922	373773503	612164791	0.5648	1.6	Null
B8I8Z8	RUMCH Uncharacterized protein	2231000	17486087	13769102	8281437	6555258	12553484	0.9761	1.2	Null
B8I900	RUMCH Uncharacterized protein	0	31324737	0	0	0	0	NA	NA	Null
B8I901	RUMCH Uncharacterized protein	0	0	0	88803	0	0	NA	NA	Null
B8I902	RUMCH Uncharacterized protein	65047826	17831767	23520552	225895075	101883529	149045243	0.0512	4.5	Increase
B8I903	RUMCH Uncharacterized protein	27083233	7579962	9696345	10605379	8562503	4260257	0.4138	1.9	Null
B8I904	RUMCH Putative phage major capsid protein	11650364	92558044	95341656	35170050	25736363	15480152	0.3007	2.6	Null
B8I905	RUMCH Minor structural GP20 protein	10358881	0	0	0	0	8270933	NA	1.3	Null
B8I908	RUMCH Uncharacterized protein	0	0	0	469473	1967531	0	0.0314	NA	Increase
B8I911	RUMCH Terminase small subunit	14353466	11582898	111493	3547183	865319	4336428	0.4918	3.0	Null
B8I912	RUMCH Uncharacterized protein	170724	10484078	36849324	3649643	4033387	3247283	0.3283	4.3	Null
B8I913	RUMCH Uncharacterized protein	3332465	4555940	6778089	726655	0	0	0.1286	20.2	Null
B8I915	RUMCH SNF2-related protein	0	0	0	0	0	597943	0.2003	NA	Null
B8I916	RUMCH VRR-NUC domain protein	772470	1554540	0	310158	0	185045	0.5631	4.7	Null
B8I917	RUMCH Virulence-associated E family protein	0	237619	0	156982	0	0	0.9468	1.5	Null
B8I918	RUMCH Uncharacterized protein	0	605384	1086819	3534466	3946851	1981463	0.2184	5.6	Null
B8I919	RUMCH Methyltransferase	0	78822	0	6216157	0	0	NA	78.9	Null
B8I922	RUMCH DNA-directed DNA polymerase	0	0	0	158826	172891	0	0.2855	NA	Null
B8I923	RUMCH Uncharacterized protein	2853892	3247265	3692660	2189226	1675751	623043	0.2055	2.2	Null
B8I924	RUMCH Uncharacterized protein	0	0	0	0	28763	0	NA	NA	Null
B8I925	RUMCH Uncharacterized protein	88742666	136234538	208856814	31430799	27070143	29240689	0.0001	4.9	Decrease
B8I926	RUMCH Uncharacterized protein	15835831	24722151	16282008	13911188	10044752	11568183	0.3222	1.6	Null
B8I927	RUMCH Uncharacterized protein	5774699	116739771	16586118	597326	481061	33677003	0.5637	4.0	Null
B8I928	RUMCH Uncharacterized protein	3759372	185760	3734570	2574201	1271522	873616	0.7077	1.6	Null

B8I935	RUMCH Transcriptional regulator, XRE family	12777878	31756451	35606632	4378487	2399033	2400425	0.0000	8.7	Decrease
B8I936	RUMCH Helix-turn-helix domain protein	547148	0	65985368	168249	0	470168	NA	104.2	Null
B8I938	RUMCH Resolvase domain protein	7673103	2671610	2159881	3777950	1353425	3176893	0.6845	1.5	Null
B8I939	RUMCH Peptidyl-prolyl cis-trans isomerase	30953244	20368571	12366645	0	12707658	7979842	0.6048	3.1	Null
B8I940	RUMCH Biotin carboxylase	2757374	2779369	6982033	5024493	777238	3695508	0.8698	1.3	Null
B8I941	RUMCH Acetyl-coenzyme A carboxylase carboxyl transferase subunit beta	2538073	10876893	12383088	7691240	5808220	4354862	0.7587	1.4	Null
B8I942	RUMCH Acetyl-coenzyme A carboxylase carboxyl transferase subunit alpha	474401	0	0	1263988	3532347	3975049	0.1755	18.5	Null
B8I943	RUMCH Amine oxidase	769389	0	122934408	40210587	38022283	16014426	NA	1.3	Null
B8I945	RUMCH Uncharacterized protein	0	0	0	0	114860	0	NA	NA	Null
B8I946	RUMCH FAD dependent oxidoreductase	0	0	191738	0	0	0	NA	NA	Null
B8I951	RUMCH Extracellular solute-binding protein family 1	1141907	136918	762102	1064316	1507122	179916	0.8698	1.3	Null
B8I952	RUMCH FAD dependent oxidoreductase	852027	1218980	1596529	482251	509163	0	0.3836	3.7	Null
B8I953	RUMCH HI0933 family protein	0	164852	0	0	69270	0	NA	2.4	Null
B8I954	RUMCH Uncharacterized protein	0	0	0	0	65343	0	NA	NA	Null
B8I956	RUMCH Alcohol dehydrogenase GroES domain protein	209719	362590	506705	1431951	794972	1386659	0.0229	3.3	Increase
B8I957	RUMCH PfkB domain protein	217993	227152	497242	316354	544235	436520	0.6226	1.4	Null
B8I961	RUMCH Transcriptional regulator, XRE family	108156083	26168356	12485113	7596588	6710938	6926543	0.0275	6.9	Decrease
B8I962	RUMCH Methyltransferase type 11	3467891	1575303	1884879	5752635	7425005	4595980	0.1294	2.6	Null
B8I963	RUMCH Radical SAM domain protein	11927878	9360154	1875185	1009815	4213264	5598340	0.5535	2.1	Null
B8I964	RUMCH Uncharacterized protein	0	152407	79673	10667219	5314713	5598577	0.0000	93.0	Increase
B8I965	RUMCH Uncharacterized protein	3499280	7857828	10685433	5286998	16529110	5366205	0.7362	1.2	Null
B8I966	RUMCH DEAD/DEAH box helicase domain protein	3457716	4046956	4151099	3232345	3244762	1932738	0.5034	1.4	Null
B8I967	RUMCH Amino acid adenylation domain protein	610640	7498483	2289288	330955	462987	773034	0.0727	6.6	Null
B8I968	RUMCH ABC transporter related	338399129	561047362	396226087	1478410881	2989086829	3631677045	0.0001	6.3	Increase
B8I969	RUMCH Transport permease protein	3533753	15256331	2985052	10717768	9088962	2937376	0.9280	1.0	Null
B8I970	RUMCH Radical SAM domain protein	7339326	3589590	1820801	1546161	1466977	1253449	0.1511	3.0	Null
B8I971	RUMCH NAD-dependent epimerase/dehydratase	6541024	13456357	2150932	7999096	9641392	7171673	0.8611	1.1	Null
B8I972	RUMCH Nucleotidyl transferase	3950940	1991921	444185	1292671	0	0	0.4849	4.9	Null
B8I973	RUMCH NAD-dependent epimerase/dehydratase	2260050	2598083	3872827	3871279	3381562	3386845	0.5412	1.2	Null
B8I974	RUMCH Glycosyltransferase, MGT family	5791720	2382768	1779569	5512994	5255094	2177710	0.8350	1.3	Null
B8I975	RUMCH Beta-lactamase	5778643	5839925	8887825	4362070	4500904	3431159	0.2652	1.7	Null
B8I976	RUMCH AMP-dependent synthetase and ligase	67659628	31315683	15355276	12413218	45231767	33389978	0.8647	1.3	Null
B8I977	RUMCH Acyl transferase	561289	0	1752100	869103	654952	487212	0.9724	1.2	Null
B8I978	RUMCH Beta-ketoacyl synthase	3528919	3836309	1954007	6308827	8579756	2953445	0.2962	1.9	Null
B8I979	RUMCH Amino acid adenylation domain protein	73967708	178277950	70028461	136018945	203720071	6718683296	NA	21.9	Null
B8I980	RUMCH Beta-ketoacyl synthase	4771721	3423457	3963133	9037011	23186844	10757224	0.0220	3.5	Increase
B8I981	RUMCH Amino acid adenylation domain protein	1579008	471385	436219	675622	1461806	2735966	0.5198	2.0	Null
B8I982	RUMCH Amino acid adenylation domain protein	854718	4124417	2055885	14969753	2068619	732620	0.4280	2.5	Null
B8I983	RUMCH Pyridoxal-dependent decarboxylase	0	93306	36808164	2037199	14250296	2662721	NA	1.9	Null
B8I984	RUMCH Amino acid adenylation domain protein	0	609532	243028	3064176	4869336	801483	0.0870	10.2	Null
B8I985	RUMCH Radical SAM domain protein	4147602	4884187	6710088	9182085	10272583	10566457	0.0577	1.9	Null
B8I986	RUMCH Uncharacterized protein	225962175	97654125	113257754	116447530	165146297	103052270	0.8872	1.1	Null
B8I988	RUMCH 3-hydroxyacyl-CoA dehydrogenase NAD-binding	0	1266224	552856	3095663	2639534	2355137	0.2783	4.4	Null
B8I989	RUMCH Carrier domain-containing protein	3107053	1441140	2266844	5026662	8480528	2816177	0.2154	2.4	Null
B8I990	RUMCH Acyl-CoA dehydrogenase domain protein	209291	2308007	1330675	3274578	8357562	1572120	0.1855	3.4	Null
B8I991	RUMCH FkbH like protein	154198	1180743	0	1638403	512219	1012888	0.6048	2.4	Null
B8I992	RUMCH Transcriptional regulator, AraC family	0	0	0	0	1052078	803980	0.0384	NA	Increase
B8I995	RUMCH Carbohydrate-binding family 9	663805	1109075	1055268	9513353	26115664	4916471	0.0000	14.3	Increase
B8I996	RUMCH Putative sensor with HAMP domain	0	366408	114873	340554	417852	302286	0.5802	2.2	Null
B8I998	RUMCH Extracellular solute-binding protein family 1	374796	708166	633637	465746	462735	0	0.7576	1.8	Null
B8I9A0	RUMCH Extracellular solute-binding protein family 1	5637136	513050272	10984120	3270845	5144409	7795992	NA	32.7	Null
B8I9A6	RUMCH Glycoside hydrolase family 31	0	0	0	0	38737	0	NA	NA	Null
B8I9A7	RUMCH Transcriptional regulator, LacI family	0	1308899	4244098	3095663	2810619	2513229	0.7950	1.5	Null
B8I9B0	RUMCH D-tagatose-bisphosphate aldolase class II accessory protein AgaZ	0	76704	0	0	0	0	NA	NA	Null
B8I9B1	RUMCH Aldo/keto reductase	0	132197	0	312254	0	0	0.8494	2.4	Null
B8I9B2	RUMCH D-ribose pyranase	0	0	0	117239	0	0	NA	NA	Null
B8I9B3	RUMCH Glycoside hydrolase family 43	16077558	16311997	0	0	865319	80672	0.1492	34.2	Null
B8I9B8	RUMCH Extracellular solute-binding protein family 1	3300658	35098069	1798171	941040	1037152	10797083	0.5796	3.1	Null
B8I9C2	RUMCH Glyco	3581216	1071519	1508713	3564028	2858104	1835547	0.7950	1.3	Null
B8I9C7	RUMCH Extracellular solute-binding protein family 1	130804180	189708294	104733631	93502309	49102131	158458394	0.7952	1.4	Null
B8I9D0	RUMCH Deoxyribose-phosphate aldolase	40803025	3515911	11915246	12262731	9436828	589783	0.4346	2.5	Null
B8I9D1	RUMCH Alcohol dehydrogenase GroES domain protein	1875272	919803	7432752	5176454	3860588	2905723	0.8463	1.2	Null
B8I9D2	RUMCH Alcohol dehydrogenase GroES domain protein	96236	0	1319472	325310	240640	944206	0.9218	1.1	Null
B8I9D4	RUMCH Short-chain dehydrogenase/reductase SDR	0	1268603	1210775	126581	171323	2604978	NA	1.2	Null
B8I9D5	RUMCH L-arabinose isomerase	4722848	19862057	2365154	8459491	15372521	909617	0.9868	1.1	Null
B8I9D6	RUMCH Transcriptional regulator, DeoR family	1534506	483748	2470820	711833	562421	4596979	0.7797	1.3	Null
B8I9D7	RUMCH Glyoxalase/bleomycin resistance protein/dioxygenase	1907735	3066710	9695809	8503784	6892295	5235041	0.5956	1.4	Null
B8I9D8	RUMCH Alpha-L-arabinofuranosidase domain protein	1277860	15584296	42030798	2268302	496740	5644046	0.1838	7.0	Null
B8I9D9	RUMCH Uncharacterized protein	4084569	5603916	3793036	7580512	5592366	8558906	0.2517	1.6	Null
B8I9E0	RUMCH ATPase, P-type (Transporting), HAD superfamily, subfamily IC	305832	710170	436448	208119	247356	367077	0.5869	1.8	Null
B8I9E1	RUMCH Pyridoxal-dependent decarboxylase	23540516	27103841	23620569	30680173	43209706	20270118	0.5722	1.3	Null
B8I9E2	RUMCH Collagenase and related protease-like protein	109875857	213882154	268950537	234575299	153301594	134181778	0.9470	1.1	Null
B8I9E3	RUMCH Uncharacterized protein	39839176	12108835	4566670	16076421	17859593	29893125	0.9342	1.1	Null
B8I9E4	RUMCH Aminotransferase class I and II	2682035	32101174	7945177	11275591	8309828	22331255	0.9015	1.0	Null
B8I9E5	RUMCH Haloacid dehalogenase domain protein hydrolase type 3	268881	306391	1192201	808243	412398	1228790	0.6690	1.4	Null
B8I9E6	RUMCH Methyltransferase type 11	0	10843799	397270	1655435	0	4038892	0.8992	2.0	Null
B8I9E7	RUMCH Radical SAM domain protein	90835255	238686599	278222870	295665907	331612506	342568259	0.2813	1.6	Null
B8I9E8	RUMCH Radical SAM domain protein	162375919	100258852	155923727	127367403	246915368	72149475	0.9265	1.1	Null
B8I9E9	RUMCH Chloramphenicol acetyltransferase	1142182	1927203	5873070	2416907	6173467	3014640	0.7037	1.3	Null
B8I9F0	RUMCH DNA helicase	1144930880	1172922106	856202394	788736465	425887448	280396363	0.1734	2.1	Null
B8I9F1	RUMCH Transcriptional regulator, AraC family	1223336	643687	1158625	1255105	2522961	3128057	0.2226	2.3	Null
B8I9F2	RUMCH Methyltransferase type 12	11996933	6919469	17457625	2855625	3025504	4393403	0.0432	3.5	Decrease
B8I9F3	RUMCH Small GTP-binding protein	691086	1013596	3110880	1001129	484084	588751	0.3423	2.3	Null
B8I9F4	RUMCH tRNA-dihydrouridine synthase	2868908	854793	1441400	171105	182058	892608	0.2245	4.1	Null
B8I9F5	RUMCH Nitroreductase	0	0	350867	100332	0	0	0.7969	3.5	Null
B8I9F6	RUMCH Uncharacterized protein	0	0	0	112570	143171	0	NA	NA	Null
B8I9F7	RUMCH Pyridine nucleotide-disulphide oxidoreductase domain protein	2287418	672604	1867090	465916	1028320	0	0.4773	3.2	Null
B8I9F8	RUMCH HI0933 family protein	1746201	2073302	2476687	1247353	2001170	1603686	0.7723	1.3	Null
B8I9F9	RUMCH Transcriptional regulator, TetR family	2492499	3987911	1538584	3007633	784390	759666	0.5295	1.8	Null
B8I9G0	RUMCH Cellulase	783961165	2767030497	1663895754	905982368	1015805253	650998019	0.2841	2.0	Null
F4BSU6	METSG Periplasmic copper-binding protein (NosD), putative	0	0	0	0	91055	121754	NA	NA	Null
F4BSV0	METSG Bifunctional enzyme Fae/Hps	49167517	1098220	1784722	3189782	1810407	2778653	NA	6.7	Null
F4BSV1	METSG Transposase, IS4 family, putative	0	0	0	4370347	0	0	NA	NA	Null
F4BSX1	METSG Transposase, IS605 OrfB family, putative	0	0	0	1365734	109886	429070	0.0006	NA	Increase
F4BSY2	METSG Universal stress protein	2363304	893728	218524	1218153	3070322	1578149	0.6935	1.7	Null
F4BSY4	METSG Thioredoxin reductase	126574	0	170940	0	0	0	0.2962	NA	Null
F4BSZ0	METSG Thermosome subunit delta	10209196	8832634	10197371	25193751	10111614	25356684	0.1935	2.1	Null
F4BSZ2	METSG Oligosaccharyl transferase	0	0	0	0	634982	0	0.2129	NA	Null
F4BSZ7	METSG Methyl-coenzyme M reductase operon protein C	8665212	881756	8179545	3377087	387051	3536702	0.5046	2.4	Null
F4BT01	METSG CBS domain protein	0	597957	359755	0	242001	0	0.7432	4.0	Null
F4BT19	METSG LTD domain-containing protein	7879891	8394843	2332134	5628478	5722940	5475927	0.9560	1.1	Null
F4BT31	METSG Uncharacterized protein	0	287204	0	0	0	0	0.3129	NA	Null
F4BT45	METSG Probable membrane transporter protein	0	0	0	707990	195529	993897	0.0001	NA	Increase
F4BT63	METSG Protein ArsC (Arsenate reductase) (Arsenical pumpmodifier)	0	0	0	0	195500	0	NA	NA	Null
F4BT66	METSG DUF4297 domain-containing protein	396776	0	0	0	0	0	0.2548	NA	Null
F4BT85	METSG Uncharacterized protein	0	0	0	0	262158	513737	0.1304	NA	Null
F4BT93	METSG Fibronectin type-III domain-containing protein	0	0	0	0	192815	0	NA	NA	Null
F4BT94	METSG Acylamino-acid-releasing enzyme	0	0	0	0	0	30652	NA	NA	Null
F4BTA1	METSG Iron-sulfur flavoprotein	392037	0	3621324	0	41197	0	0.0795	97.4	Null
F4BTA6	METSG Ferredoxin-thioredoxin reductase subunit B	0	0	161287	27686	0	0	NA	5.8	Null
F4BTA8	METSG Uncharacterized protein	353575	0	359122	389946	0	0	0.8669	1.8	Null
F4BTB1	METSG Myo-inositol-1-phosphate synthase	0	0	0	0	51899	397348	0.2332	NA	Null

F4BTC6	METSG Uncharacterized protein	1762530	363148	842243	1391399	1355419	1038210	0.8444	1.3	Null
F4BTC7	METSG Uncharacterized protein	6704773	10075767	16424788	3918434	6643051	4855490	0.2274	2.2	Null
F4BTC8	METSG 2-amino-3,7-dideoxy-D-threo-hept-6-ulosonate synthase	547664	2393799	507282	810717	3767636	5374995	0.2426	2.9	Null
F4BTD5	METSG KaiC	0	446531	599984	684924	376830	436021	0.8027	1.4	Null
F4BTF7	METSG DNA primase large subunit PriL	0	12024151	0	0	0	0	NA	NA	Null
F4BTF8	METSG DNA polymerase sliding clamp	0	321008	6306765	1288499	1066336	2075524	NA	1.5	Null
F4BTF9	METSG Transcription elongation factor IIS/RNA polymerase subunit homol	0	136907	0	0	0	0	NA	NA	Null
F4BTG4	METSG Glutamyl-tRNA(Gln) amidotransferase subunit E	20408918	0	0	0	0	0	NA	NA	Null
F4BTI8	METSG Transcriptional regulatory protein, AsnC family	0	0	0	1167081	1151684	0	0.0307	NA	Increase
F4BTI9	METSG Uncharacterized protein	529064	486046	0	0	72667	75101	0.4419	6.9	Null
F4BTJ4	METSG Transcriptional regulator TrmB, putative	0	0	142760	117772	0	0	NA	1.2	Null
F4BTJ9	METSG 4Fe-4S ferredoxin, iron-sulfur binding domain protein	1876381	2512646	3491867	841408	2307710	178375	0.4103	2.4	Null
F4BTL5	METSG Ribulose bisphosphate carboxylase, large chain (Form II)	0	0	113014	166441	81158	0	0.8463	2.2	Null
F4BTN1	METSG Two-component hybrid sensor and regulator	0	0	0	0	0	4184495	NA	NA	Null
F4BTN7	METSG TM2 domain-containing protein	0	1059452	0	16131453	0	0	NA	15.2	Null
F4BTP0	METSG DNA-directed RNA polymerase subunit L	0	0	0	106163	0	241806	0.2655	NA	Null
F4BTP1	METSG Exosome complex component Csl4	0	0	0	0	158752	0	NA	NA	Null
F4BTP4	METSG Peptide chain release factor subunit 1	0	157781	0	0	0	88393	NA	1.8	Null
F4BTQ6	METSG Uncharacterized protein	0	159156	0	0	0	84907	NA	1.9	Null
F4BTQ7	METSG Ribosomal L15	0	189198	533893	0	113962	0	0.6138	6.3	Null
F4BTR2	METSG Exosome complex component Rrp41	0	0	0	0	213484	0	NA	NA	Null
F4BTR8	METSG Prefoldin subunit beta	0	1103133	1010263	332103	254094	0	0.6405	3.6	Null
F4BTS0	METSG Lipoprotein, putative	1088280	0	1162312	0	2459000	2994351	0.7463	2.4	Null
F4BTS2	METSG M6 family metalloprotease domain protein	0	0	218290	225950	450641	813081	0.3082	6.8	Null
F4BTS7	METSG Beta-lactamase domain protein	0	0	0	0	104420	0	NA	NA	Null
F4BTV3	METSG Uncharacterized protein	1702557	3083311	1722058	550380	463352	438352	0.0008	4.5	Decrease
F4BTZ1	METSG Transposase, IS605 OrfB family	0	0	147275	0	0	0	NA	NA	Null
F4BTZ6	METSG Fasciclin domain protein	0	319794	0	0	0	0	0.3018	NA	Null
F4BTZ9	METSG Lipoprotein, putative	0	81804	0	0	246206	0	0.7911	3.0	Null
F4BU06	METSG Cna protein B-type domain protein	181670	0	0	0	280937	284682	0.7930	3.1	Null
F4BU14	METSG Uncharacterized protein	585216	2239360	0	0	0	0	0.0296	NA	Decrease
F4BU16	METSG DNA mismatch repair protein MutL	0	0	0	0	0	4893516	NA	NA	Null
F4BU22	METSG LIM zinc-binding domain-containing protein	0	0	0	0	265228	0	0.3000	NA	Null
F4BU43	METSG Metallo-beta-lactamase domain protein	0	0	0	0	58451	0	NA	NA	Null
F4BU54	METSG DNA primase small subunit PriS	0	0	0	0	0	1861182	NA	NA	Null
F4BU58	METSG Periplasmic binding protein	215844	0	246159	0	0	0	0.2500	NA	Null
F4BU62	METSG Uncharacterized protein	770811	625141	999265	0	0	358664	0.4532	6.7	Null
F4BU63	METSG Rubrerythrin	0	0	0	0	119828	0	NA	NA	Null
F4BU67	METSG Multi-sensor hybrid histidine kinase	0	0	304956	0	0	0	0.3076	NA	Null
F4BU75	METSG Elongation factor 1-alpha	4139027	12090148	34398899	5117255	7342687	7102636	0.3357	2.6	Null
F4BU76	METSG Elongation factor 2	576123	2340480	1964773	931819	0	92646	0.3549	4.8	Null
F4BU77	METSG Aspartokinase	0	0	0	0	112988	0	NA	NA	Null
F4BU92	METSG DNA helicase	0	0	0	113127	11082	0	NA	NA	Null
F4BU95	METSG 30S ribosomal protein S27ae	0	356755	0	0	0	0	0.2917	NA	Null
F4BU96	METSG 30S ribosomal protein S24e	1586458	0	1952528	120407	0	0	0.2102	29.4	Null
F4BUA1	METSG Translation initiation factor 2 subunit gamma	0	0	0	0	0	147751	NA	NA	Null
F4BUA8	METSG Uncharacterized protein	128786	134302	263524	353415	240368	671038	0.2583	2.4	Null
F4BUB1	METSG Uncharacterized protein	0	0	11738213	426021	145631	0	NA	20.5	Null
F4BUE7	METSG SMC	0	1636563	6221327	308549	0	441326	0.3604	10.5	Null
F4BUF9	METSG Crispr-associated helicase Cas3	4964331	0	0	0	7719392	4722591	0.8463	2.5	Null
F4BUH1	METSG Elongation factor 1-beta	822361	414937	967478	935321	0	0	NA	2.4	Null
F4BUH5	METSG Glutamate--tRNA ligase	0	4026550	0	0	0	0	NA	NA	Null
F4BUH7	METSG Acylphosphatase	0	0	0	0	0	1728241	NA	NA	Null
F4BUI2	METSG Thermosome subunit alpha	9012149	38636017	43857142	11589971	11733173	32387518	0.7765	1.6	Null
F4BUI4	METSG DUF5611 domain-containing protein	150743	0	0	0	0	0	NA	NA	Null
F4BUI5	METSG Enolase	574531	0	0	48798	188131	216573	0.9412	1.3	Null
F4BUJ0	METSG Rubrerythrin	948380	0	193534	0	0	0	0.0949	NA	Null
F4BUJ1	METSG Uncharacterized protein	1466763	1420377	1870342	777839	722137	677369	0.0884	2.2	Null
F4BUJ2	METSG TPR repeat-containing protein	0	13215535	11592253	4882413	4463552	3956595	0.8453	1.9	Null
F4BUJ7	METSG CBS domain protein	0	0	117998	0	0	0	NA	NA	Null
F4BUJ8	METSG Universal stress protein	0	1440690	323268	1497797	824099	114940	0.8638	1.4	Null
F4BUM4	METSG Uncharacterized protein	0	0	0	826200	0	0	NA	NA	Null
F4BUM9	METSG Uncharacterized protein	0	0	0	0	50574	0	NA	NA	Null
F4BUQ0	METSG Chromosomal protein MC1b	0	575388	0	0	0	0	0.2435	NA	Null
F4BUR1	METSG Precorrin-8X methylmutase	0	0	0	90814	0	0	NA	NA	Null
F4BUS3	METSG Transcription initiation factor IIB	0	298292	447370	217167	146197	215493	0.9723	1.3	Null
F4BUS8	METSG Thermosome subunit gamma	0	1784935	179993	1556392	2160864	3003455	0.4101	3.4	Null
F4BUS9	METSG Cold shock DNA binding domain protein	0	384107	682037	0	0	0	0.1193	NA	Null
F4BUT5	METSG 50S ribosomal protein L7Ae	223722	263113	428043	305376	421498	1088855	0.3476	2.0	Null
F4BUT6	METSG Uncharacterized protein	0	379420	0	0	0	0	0.2862	NA	Null
F4BUT9	METSG Uncharacterized protein	261682	0	0	0	0	0	0.2965	NA	Null
F4BUU0	METSG Acetate--CoA ligase (ADP-forming)	0	0	0	0	102256	0	NA	NA	Null
F4BUU3	METSG Signal recognition particle 54 kDa protein	348519	0	884088	44437464	209759	0	NA	36.2	Null
F4BUU6	METSG Uncharacterized protein	0	0	2139701	0	0	0	NA	NA	Null
F4BUV9	METSG Tungsten formylmethanofuran dehydrogenase, subunit B	416243	0	1350108	296967	434790	342440	0.8496	1.6	Null
F4BUW0	METSG Tungsten formylmethanofuran dehydrogenase, subunit D	0	0	0	0	375150	0	0.2664	NA	Null
F4BUW8	METSG Crispr-associated protein, Csh2 family	0	0	0	130396	153344	43908	0.0960	NA	Null
F4BUW9	METSG Crispr-associated protein, TM1802 family	0	0	0	0	0	182484	NA	NA	Null
F4BUY1	METSG CoB-CoM heterodisulfide reductase, subunit B	0	1378916	0	84682	2739139	158684	NA	2.2	Null
F4BUY6	METSG Triosephosphate isomerase	362954	1610221	2132117	826256	738494	326953	0.4267	2.2	Null
F4BUY7	METSG Acylphosphatase	0	0	247117	0	0	50811	0.7804	4.9	Null
F4BUZ5	METSG Glutamate dehydrogenase	0	0	0	56055	20295	0	NA	NA	Null
F4BV02	METSG GTP cyclohydrolase MptA	0	0	0	84853	0	50293	NA	NA	Null
F4BV07	METSG 2-amino-3,7-dideoxy-D-threo-hept-6-ulosonate synthase	942128	3736234	1236523	1218450	859094	879947	0.4243	2.0	Null
F4BV11	METSG Conserved domain protein	0	0	0	133613	0	0	NA	NA	Null
F4BV16	METSG TRAM domain protein	0	0	0	43292	0	199823	0.2989	NA	Null
F4BV33	METSG Uncharacterized protein	645903	0	2379064	0	202889	0	0.3642	14.9	Null
F4BV36	METSG Argininosuccinate synthase	0	0	0	186947	345483	295332	0.0023	NA	Increase
F4BV37	METSG Uncharacterized protein	582949	520430	238508	1359066	800906	0	0.8463	1.6	Null
F4BV39	METSG Extracellular protein, putative	0	0	0	186744	0	0	NA	NA	Null
F4BV41	METSG Uncharacterized protein	0	0	0	238988	119438	0	0.2786	NA	Null
F4BV52	METSG Bifunctional protein FolD	0	0	183238	172740	512401	121819	0.4950	4.4	Null
F4BV55	METSG Tyrosine--tRNA ligase	0	0	0	228441	356537	155326	0.0042	NA	Increase
F4BV65	METSG Formylmethanofuran dehydrogenase subunit D	0	98197	0	844272	154689	0	0.4549	10.2	Null
F4BV66	METSG Formylmethanofuran dehydrogenase	1574178	0	0	73144	0	0	NA	21.5	Null
F4BV67	METSG Formylmethanofuran dehydrogenase subunit A	0	104084	347848	110184	0	0	0.7277	4.1	Null
F4BV79	METSG AAA family ATPase, CDC48 subfamily	0	372720	277897	328481	1369393	655887	0.3320	3.6	Null
F4BVA0	METSG Thioredoxin	0	345231	0	0	0	0	0.2939	NA	Null
F4BVA4	METSG Uncharacterized protein	273879	0	0	0	0	0	0.2925	NA	Null
F4BVB1	METSG Phage shock protein A, putative	0	322843	0	0	0	0	0.3018	NA	Null
F4BVB6	METSG Aspartyl/glutamyl-tRNA(Asn/Gln) amidotransferase subunit C	0	0	0	91962	0	298751	0.2524	NA	Null
F4BVC1	METSG DJ-1/PfpI family/rubredoxin fusion protein	0	105185	0	0	102488	0	NA	1.0	Null
F4BVC3	METSG Transposase, IS4	0	0	0	1456782	0	0	NA	NA	Null
F4BVD5	METSG Peroxiredoxin	0	0	0	289434	0	248845	0.2070	NA	Null
F4BVE1	METSG SufBD domain protein	0	0	0	0	118936	0	NA	NA	Null
F4BVE2	METSG ABC transporter ATP-binding protein	0	252960	10638457	293054	0	0	NA	37.2	Null
F4BVE4	METSG Diaminopimelate decarboxylase	0	0	0	0	51638	0	NA	NA	Null
F4BVF3	METSG Uncharacterized protein	783659	0	0	0	0	0	NA	NA	Null
F4BVF6	METSG Cupin domain protein	0	0	0	347514	241163	311542	0.0013	NA	Increase
F4BVK0	METSG Transposase, IS116/IS110/IS902 family	646029	287758	0	114556078	0	92734	NA	122.8	Null
F4BVK8	METSG D-3-phosphoglycerate dehydrogenase	592840	614689	370741	1176037	1045312	454876	0.4226	1.7	Null
F4BVK9	METSG Flavoprotein	0	0	0	64640	77034	0	NA	NA	Null

F4BVM0	METSG Uncharacterized protein	0	0	0	0	490994	0	0.2401	NA	Null
F4BVN2	METSG Uncharacterized protein family (UPF0150)	0	0	0	0	73344	64082	NA	NA	Null
F4BVN6	METSG Pentapeptide repeat protein	0	0	0	0	30808	0	NA	NA	Null
F4BVR1	METSG CoB--CoM heterodisulfide reductase iron-sulfur subunit D	529734	0	864267	531894	461874	334866	0.9940	1.0	Null
F4BVT8	METSG Ion transport protein	0	0	0	0	4083808	0	NA	NA	Null
F4BVU5	METSG Uncharacterized protein	0	0	1515401	0	0	0	NA	NA	Null
F4BVU9	METSG Aconitate hydratase, putative	0	0	0	0	141964	0	NA	NA	Null
F4BVX9	METSG Heavy metal-associated domain protein	0	0	277825	0	0	0	NA	NA	Null
F4BVZ3	METSG Methyl coenzyme M reductase system, component A2	0	0	0	369191	249563	263264	0.0014	NA	Increase
F4BVZ4	METSG UPF0288 protein MCON	181564	0	533424	262085	208246	0	0.8947	1.5	Null
F4BVZ6	METSG Putative methanogenesis marker protein 5	0	83794	99789	0	34819	86388	0.9679	1.5	Null
F4BVZ7	METSG Putative methanogenesis marker protein 15	0	75702	0	0	1325992	1236026	0.1737	33.8	Null
F4BW05	METSG Lipoprotein, putative	0	172478	204725	603008	260390	153934	0.4957	2.7	Null
F4BW08	METSG Uncharacterized protein	0	0	0	193515	952473	639386	0.0002	NA	Increase
F4BW15	METSG Uncharacterized protein	0	0	0	0	46871	0	NA	NA	Null
F4BW30	METSG Phosphoribosylamine--glycine ligase	0	0	3972588	0	0	0	NA	NA	Null
F4BW31	METSG Glutamine synthetase	0	0	0	37868068	0	166388	NA	NA	Null
F4BW40	METSG tRNA uridine(34) acetyltransferase	0	0	0	337628	0	0	0.2864	NA	Null
F4BW41	METSG 30S ribosomal protein S8e	0	392074	373888	552439	462332	0	0.9056	1.3	Null
F4BW56	METSG Manganese-dependent inorganic pyrophosphatase	24628603	22606267	36995419	20271221	17082268	27279148	0.7969	1.3	Null
F4BW60	METSG Uncharacterized protein	273934	0	0	0	0	0	0.2925	NA	Null
F4BW64	METSG Peptidase M24	0	0	105592	0	0	0	NA	NA	Null
F4BW70	METSG Methanogen homoaconitase small subunit	0	0	0	0	27443	0	NA	NA	Null
F4BW73	METSG Sirohydrochlorin cobaltochelatase	416775	1165066	0	247355	0	0	0.5732	6.4	Null
F4BW81	METSG MTH865-like family protein	0	174628	0	0	0	0	NA	NA	Null
F4BW82	METSG Pyridoxamine 5'-phosphate oxidase-related, FMN-binding protein	1110025	0	1476214	399936	884965	208522	0.8066	1.7	Null
F4BW83	METSG Putative methanogenesis marker protein 8	0	0	0	110404	0	0	NA	NA	Null
F4BW85	METSG Pept	0	79205	0	0	0	0	NA	NA	Null
F4BW87	METSG 30S ribosomal protein S3Ae	0	143914	274847	100727	176003	0	0.9313	1.5	Null
F4BWA5	METSG Uncharacterized protein	0	0	0	0	0	3576762	NA	NA	Null
F4BWB0	METSG TPR-repeat protein	341570	270620	362520	0	0	0	0.0014	NA	Decrease
F4BWB2	METSG TPR-repeat protein	0	1467167	0	0	1263739	0	NA	1.2	Null
F4BWC0	METSG TPR-repeat protein	0	0	0	0	99930	77315	NA	NA	Null
F4BWD4	METSG TPR-repeat protein	0	0	535314	0	0	196680	0.8817	2.7	Null
F4BWF8	METSG Uncharacterized protein	1407282	0	0	0	190132	0	NA	7.4	Null
F4BWG9	METSG 3-hexulose-6-phosphate synthase	0	0	0	0	428985	0	0.2548	NA	Null
F4BWK6	METSG Uncharacterized protein	3038883	4529481	4748645	1148294	1128040	400011	0.0021	4.6	Decrease
F4BWL4	METSG ABC transporter, extracellular solute-binding protein, family 5	0	0	92705	0	0	0	NA	NA	Null
F4BWM5	METSG Rubrerythrin domain-containing protein	1434971	491197	936844	0	0	0	0.0000	NA	Decrease
F4BWM9	METSG Acetyl-CoA decarbonylase/synthase complex subunit beta	0	0	0	103958	56563	0	NA	NA	Null
F4BWP1	METSG Uncharacterized protein	0	0	0	74459	0	0	NA	NA	Null
F4BWP2	METSG tRNA(Phe) (4-demethylwyosine(37)-C(7)) aminocarboxypropyltran	171091	0	0	153806	0	0	0.9475	1.1	Null
F4BWP4	METSG Heat shock protein Hsp20, putative	0	1439495	4354137	0	0	0	0.0096	NA	Decrease
F4BWP7	METSG Adenylate kinase	16856780	8270453	1895564	1114978	3401677	9853029	0.6890	1.9	Null
F4BWQ0	METSG Uncharacterized protein	0	237196	478457	0	169687	213956	0.8927	1.9	Null
F4BWQ4	METSG Cation-transporting P-type ATPase	56436	0	0	0	0	0	NA	NA	Null
F4BWT3	METSG TATA-box-binding protein	0	0	0	214807	185734	165210	0.0110	NA	Increase
F4BWT6	METSG CBS domain containing membrane protein	0	12622755	0	297785	156638	0	NA	27.8	Null
F4BWT7	METSG CBS domain protein	159044	669643	1412659	387763	458859	814513	0.9247	1.3	Null
F4BWT9	METSG CBS domain pair protein	0	1012911	0	0	0	0	NA	NA	Null
F4BWU0	METSG CBS domain protein	0	0	140983	0	182208	0	0.9455	1.3	Null
F4BWU7	METSG Uncharacterized protein	0	0	0	0	0	2969029	NA	NA	Null
F4BWV2	METSG PyrE-like protein	0	0	0	1048685	223405	0	0.0895	NA	Null
F4BWV6	METSG Pyridoxamine 5'-phosphate oxidase-related, FMN-binding protein	842657	371471	252372	539897	123087	529194	0.8868	1.2	Null
F4BWW9	METSG Peptidase M28, putative	0	0	0	269367	0	0	NA	NA	Null
F4BWZ9	METSG Two-component hybrid sensor and regulator	0	236417	0	0	0	0	NA	NA	Null
F4BX03	METSG Adenylosuccinate synthetase	0	1963577	451012	939359	1078086	897451	0.8698	1.2	Null
F4BX04	METSG Tetrahydromethanopterin:alpha-L-glutamate ligase	0	0	863656	0	277784	0	NA	3.1	Null
F4BX05	METSG Acetyl-coenzyme A synthetase	7070524	36173650	9747715	4156396	4912551	4418417	0.0836	3.9	Null
F4BX06	METSG Acetyl-coenzyme A synthetase	112121910	40555821	352737581	82252067	43861005	83937828	0.4014	2.4	Null
F4BX07	METSG Acetyl-coenzyme A synthetase	203314	0	0	0	277587	474542	0.7386	3.7	Null
F4BX14	METSG Uncharacterized protein	0	0	0	179854	0	0	NA	NA	Null
F4BX21	METSG Uncharacterized protein	254247	0	0	691362	137651	0	0.8112	3.3	Null
F4BX24	METSG DUF89 domain-containing protein	0	0	588579	0	0	0	0.2401	NA	Null
F4BX26	METSG AMP phosphorylase	0	0	0	796863	0	0	NA	NA	Null
F4BX30	METSG Proteasome-activating nucleotidase	0	0	0	506945	340597	351928	0.0002	NA	Increase
F4BX70	METSG Uncharacterized protein	0	0	0	82560	0	0	NA	NA	Null
F4BX98	METSG Periplasmic binding protein	0	145358	316521	269845	131949	0	0.9761	1.1	Null
F4BX99	METSG Periplasmic binding protein	0	0	168484	0	290183	0	0.8927	1.7	Null
F4BXA2	METSG ABC transporter, CydDC cysteine exporter family, permease/ATP-b	562738	0	0	0	0	0	0.2183	NA	Null
F4BXD2	METSG ABC transporter, ATP-binding protein	774790	0	0	0	0	0	NA	NA	Null
F4BXD4	METSG Imidazole glycerol phosphate synthase subunit HisF	96542	0	0	0	0	0	NA	NA	Null
F4BXD7	METSG Transposase, IS4	0	0	0	0	269585	0	0.3000	NA	Null
F4BXF0	METSG 30S ribosomal protein S13	0	645236	0	0	0	0	0.2319	NA	Null
F4BXH1	METSG Adenosylcobinamide amidohydrolase	0	89684	0	204368	191983	89367	0.4137	5.4	Null
F4BXH8	METSG Magnesium-chelatase subunit ChlD	0	0	0	0	92929	0	NA	NA	Null
F4BXI2	METSG Periplasmic binding protein	0	426970	0	0	0	0	0.2737	NA	Null
F4BXJ5	METSG Polymorphic outer membrane protein	0	0	0	0	0	236862	0.2950	NA	Null
F4BXK7	METSG Uncharacterized protein	0	0	765751	0	0	0	NA	NA	Null
F4BXL3	METSG Transposase, IS116/IS110/IS902 family	0	0	0	0	74285	0	NA	NA	Null
F4BXN7	METSG Periplasmic binding protein	0	0	432399	0	0	0	0.2721	NA	Null
F4BXN8	METSG CobN/magnesium chelatase domain protein	0	0	0	0	0	306572	0.2688	NA	Null
F4BXQ1	METSG Magnesium-chelatase subunit ChlD (Mg-protoporphyrin IXchelatas	0	0	0	86647	0	0	NA	NA	Null
F4BXQ5	METSG Two-component hybrid sensor and regulator	0	0	3882812	0	865319	993897	0.9029	2.1	Null
F4BXS7	METSG Dinitrogenase iron-molybdenum cofactor biosynthesis protein	661020	0	0	283717	533177	69277	0.9692	1.3	Null
F4BXT6	METSG Heat shock protein Hsp20	298165	1552530	1309289	569040	170227	363203	0.2855	2.9	Null
F4BXU3	METSG AAA	0	147104	0	0	0	0	NA	NA	Null
F4BXV1	METSG Conserved repeat domain protein	0	63206	0	92013	374853	0	0.5300	7.4	Null
F4BXV3	METSG Phosphoglucomutase/phosphomannomutase	0	0	4466357	0	0	0	NA	NA	Null
F4BXV8	METSG Uncharacterized protein	125687	1040691	329653	630385	1038011	1905496	0.2917	2.4	Null
F4BXX1	METSG LTD domain-containing protein	0	0	225573	0	92918	173762	0.9426	1.2	Null
F4BXX2	METSG Cobalt chelatase	4412739	5904907	5992549	2027907	1992101	0	0.3847	4.1	Null
F4BXX8	METSG Uncharacterized protein	0	0	0	649597	471852	0	0.0933	NA	Null
F4BXY1	METSG Methyl-coenzyme M reductase subunit alpha	1507504	14613955	14916751	5479489	568050	1190025	0.2633	4.3	Null
F4BXY2	METSG Coenzyme-B sulfoethylthiotransferase	790205745	744903453	302681611	121229499	124172576	116030697	0.0030	5.1	Decrease
F4BXY4	METSG Methyl-coenzyme M reductase subunit beta	67903876	682437046	68321545	94683064	91191821	192608186	0.8804	2.2	Null
F4BXZ5	METSG Cation-transporting P-type ATPase	0	0	593116	0	0	0	0.2401	NA	Null
F4BXZ6	METSG S-layer-related duplication domain protein	221302	617332	0	0	102418	321734	0.8724	2.0	Null
F4BY44	METSG Coenzyme F420 hydrogenase subunit beta	1023598	539867	690007	246161	173244	213823	0.0611	3.6	Null
F4BY47	METSG Flavoprotein	0	0	0	364557	0	0	0.2813	NA	Null
F4BY48	METSG Phosphate binding protein	0	6276828	3366600	0	112972	0	0.0929	85.4	Null
F4BY61	METSG TPR-repeat protein	50825297	11582898	6037437	0	177728	0	0.0002	385.1	Decrease
F4BY65	METSG Uncharacterized protein	0	287674	96516	615142	524138	797649	0.2140	5.0	Null
F4BY67	METSG Uncharacterized protein	0	0	11959188	70604288	76623710	73814230	0.2226	18.5	Null
F4BY70	METSG Pyridoxal 5'-phosphate synthase subunit PdxS	0	0	47236	100068	61511	221993	0.3007	8.1	Null
F4BY71	METSG WD40-like Beta Propeller Repeat protein	0	396560	0	0	0	0	0.2818	NA	Null
F4BY73	METSG Phosphoglycerate kinase	497033	0	0	1036938	3037066	168195	0.3995	8.5	Null
F4BY85	METSG Histidinol dehydrogenase	0	0	0	494964	393292	344116	0.0002	NA	Increase
F4BY87	METSG Homoserine dehydrogenase	0	0	0	216647	1226387	540991	0.0001	NA	Increase
F4BY88	METSG Amino acid-binding ACT domain protein	0	0	0	968429	0	0	NA	NA	Null
F4BY95	METSG Uncharacterized protein	424592	1810630	3259776	721816	1059175	826074	0.5007	2.1	Null
F4BYA4	METSG Phenylacetate-coenzyme A ligase	0	0	1630727	1655435	0	2171379	0.7962	2.3	Null

F4BYA5	METSG Amino acid-binding ACT domain protein	302648	0	530091	860826	2129058	924261	0.2925	4.7	Null
F4BYB3	METSG AAA family ATPase, CDC48 subfamily	127006	0	1003844	195795	261293	179342	0.8463	1.8	Null
F4BYD5	METSG Uncharacterized protein family (UPF0175)	0	0	0	174710	159948	48177	0.0627	NA	Null
F4BYH6	METSG Carbon monoxide dehydrogenase accessory protein	0	303461	0	0	0	0	0.3084	NA	Null
F4BYH7	METSG Extracellular solute-binding protein, family 5	0	11913838	21097362	87649	0	0	0.0144	376.6	Decrease
F4BYI3	METSG 5,10-methylenetetrahydromethanopterin reductase	82507	232214	223337	649634	165211	226570	0.4318	1.9	Null
F4BYJ1	METSG Uncharacterized protein	0	0	0	0	747038	0	NA	NA	Null
F4BYK4	METSG Leucine--tRNA ligase	0	0	287090	251914	0	0	0.9809	1.1	Null
F4BYM0	METSG DUF1156 domain-containing protein	0	0	0	0	0	791319	NA	NA	Null
F4BYM4	METSG Chromosome partition protein Smc	0	0	22129787	0	938705	0	NA	23.6	Null
F4BYN1	METSG Probable GTP 3',8-cyclase	0	0	0	8270	0	690030	0.1852	NA	Null
F4BYP0	METSG Glycosyltransferase family 57 protein	0	0	0	70146	0	0	NA	NA	Null
F4BYP5	METSG Glutamate-1-semialdehyde 2,1-aminomutase	0	0	0	169426	0	0	NA	NA	Null
F4BYQ2	METSG Radical SAM domain protein	527945	410555	566004	160979	521957	0	0.6618	2.2	Null
F4BYQ8	METSG Chromosomal protein MC1a	0	1852674	2160300	491446	441803	307509	0.5747	3.2	Null
F4BYR2	METSG Translation initiation factor 2 subunit beta	956635	317963	0	350944	0	299329	0.8186	2.0	Null
F4BYS7	METSG Uncharacterized protein	0	0	0	0	803066	292777	0.0917	NA	Null
F4BYW0	METSG Universal stress protein	0	0	0	0	0	11626	NA	NA	Null
F4BYW5	METSG Phosphoadenosine phosphosulfate reductase, putative	0	0	0	0	0	402369	0.2435	NA	Null
F4BYW6	METSG Cold shock DNA binding domain protein	191579	409409	0	0	0	203178	0.8354	3.0	Null
F4BYX4	METSG WD	0	1709856	2069869	0	0	0	0.0175	NA	Decrease
F4BYY7	METSG Uncharacterized protein	427705	0	341930	0	0	0	0.1500	NA	Null
F4BYZ4	METSG Uncharacterized protein	0	0	139747	0	0	0	NA	NA	Null
F4BYZ7	METSG Soluble hydrogenase small subunit	0	1456136	0	2042316	3330201	1767080	0.4580	4.9	Null
F4BYZ8	METSG Tryptophan synthase beta chain	0	536459	767172	679251	460498	418870	0.8782	1.2	Null
F4BZ00	METSG V-type ATP synthase subunit D	8016310	0	0	6170113	424661	2258907	0.9902	1.1	Null
F4BZ01	METSG V-type ATP synthase beta chain	10029269	16731000	37351868	37485838	23254178	74063721	0.2655	2.1	Null
F4BZ02	METSG V-type ATP synthase alpha chain	20172726	30493483	23256104	52950283	51281549	75120046	0.0206	2.4	Increase
F4BZ03	METSG V-type ATP synthase subunit F	4827217	2812990	970066	4362070	1244151	860955	0.7805	1.3	Null
F4BZ04	METSG V-type ATP synthase subunit C	0	432363	0	910489	293404	0	0.7841	2.8	Null
F4BZ05	METSG V-type proton ATPase subunit E	6643193	5036618	2438744	5024244	722137	5874752	0.9149	1.2	Null
F4BZ07	METSG V-type ATP synthase subunit I	3251615	1563687	1733528	2200837	1384000	1938619	0.8648	1.2	Null
F4BZ08	METSG V-type ATP synthase subunit H	11747498	26492797	33512725	7165823	7264944	13199200	0.2083	2.6	Null
F4BZ09	METSG Fructose-1,6-bisphosphatase class 1	0	0	0	799143	157809	88594	0.0059	NA	Increase
F4BZ10	METSG Uncharacterized protein	0	0	0	3166565	196282	2856704	0.0000	NA	Increase
F4BZ27	METSG Nitroreductase/iron-sulfur domain protein	0	0	0	122083	0	0	NA	NA	Null
F4BZ82	METSG Cna B domain protein	0	0	0	88896	28799	0	NA	NA	Null
F4BZ83	METSG Formaldehyde-activating enzyme	0	0	0	0	0	784988	NA	NA	Null
F4BZ86	METSG Chaperone protein DnaJ	0	0	433822	0	0	0	0.2721	NA	Null
F4BZ87	METSG Chaperone protein DnaK	1406209	3150443	7688808	9858638	7564450	2485444	0.5384	1.6	Null
F4BZ89	METSG Thermosome beta subunit	110576	1597226	2760612	217925	319341	326431	0.1877	5.2	Null
F4BZA4	METSG Uncharacterized protein	0	0	0	0	53511	0	NA	NA	Null
F4BZB0	METSG Tetrahydromethanopterin S-methyltransferase subunit H	0	4101534	3001670	1286364	596286	2065009	0.8664	1.8	Null
F4BZB5	METSG 30S ribosomal protein S2	152111	390303	612755	0	181899	479082	0.8764	1.7	Null
F4BZB8	METSG 30S ribosomal protein S9	3154943	2768864	0	0	96632	702691	0.4636	7.4	Null
F4BZC4	METSG DegT/DnrJ/EryC1/StrS aminotransferase	0	0	224260	0	0	0	NA	NA	Null
F4BZE7	METSG Iron-sulfur flavoprotein	0	0	0	1266408	1369570	0	0.0247	NA	Increase
F4BZG3	METSG Signal recognition particle receptor FtsY	4846133	0	0	0	238777	133772	NA	13.0	Null
F4BZG4	METSG Prefoldin subunit alpha	100680	0	0	0	0	0	NA	NA	Null
F4BZH0	METSG 30S ribosomal protein S19e	0	1361669	1371347	337261	444191	0	0.6590	3.5	Null
F4BZH1	METSG Cation-transporting P-type ATPase	0	0	5386561	256557	268674	344181	NA	6.2	Null
F4BZH5	METSG Universal stress protein	0	0	0	0	47712	0	NA	NA	Null
F4BZH6	METSG Aspartate carbamoyltransferase	0	0	0	0	35397	0	NA	NA	Null
F4BZH8	METSG Peptidyl-prolyl cis-trans isomerase	0	319605	204857	1059478	1475427	603538	0.1564	6.0	Null
F4BZI5	METSG Cellulase	0	0	0	0	144145	0	NA	NA	Null
F4BZI6	METSG Universal stress protein	6367347	12950783	56248217	13776440	18440620	14937260	0.7600	1.6	Null
F4BZJ2	METSG Tetrahydromethanopterin S-methyltransferase subunit A	1526236	1378487	863313	2240790	5637448	5105814	0.0435	3.4	Increase
F4BZJ3	METSG Tetrahydromethanopterin S-methyltransferase subunit B	0	196799	0	399230	884821	740675	0.2094	10.3	Null
F4BZK4	METSG Uncharacterized protein	4284967	2008134	3043788	2150901	1429379	1716650	0.3898	1.8	Null
F4BZL1	METSG ATPase associated with various cellular activities (AAA)	0	0	0	188570	0	0	NA	NA	Null
F4BZL4	METSG DNA gyrase subunit A	0	0	0	0	49229	65357	NA	NA	Null
F4BZL5	METSG Dead/deah box helicase domain protein	0	0	128316	0	0	4059397	NA	31.6	Null
F4BZM2	METSG Uncharacterized protein	0	0	299574	178866	162468	141855	0.8463	1.6	Null
F4BZQ0	METSG 30S ribosomal protein S6e	1556310	1653826	4889085	1145713	1157300	539454	0.1625	2.8	Null
F4BZQ3	METSG Dihydroxy-acid dehydratase	0	600662	1067982	146941	711938	326137	0.9449	1.4	Null
F4BZQ7	METSG S-layer-related duplication domain protein	9719846	5682704	5110953	13842895	27958862	6794005	0.2613	2.4	Null
F4BZR0	METSG Heat shock protein Hsp20	0	238328	294822	109344	175942	192295	0.9724	1.1	Null
F4BZR7	METSG Uncharacterized protein	1348718	0	0	0	0	0	NA	NA	Null
F4BZR9	METSG Acetylglutamate kinase	0	116403	0	0	204377	0	0.8950	1.8	Null
F4BZT8	METSG Cytidylate kinase	0	0	0	369952	0	0	0.2787	NA	Null
F4BZU1	METSG 50S ribosomal protein L15	0	0	0	234564	0	188060	0.2529	NA	Null
F4BZU3	METSG 30S ribosomal protein S5	0	284709	0	0	221929	0	0.9836	1.3	Null
F4BZU4	METSG 50S ribosomal protein L18	382728	44990	0	0	0	0	0.2519	NA	Null
F4BZU5	METSG 50S ribosomal protein L19e	0	408265	468949	253140	80495	148259	0.8324	1.8	Null
F4BZU6	METSG 50S ribosomal protein L32e	0	199215	0	0	0	0	NA	NA	Null
F4BZU7	METSG 50S ribosomal protein L6	383998	2701067	3143015	296056	442204	637102	0.1439	4.5	Null
F4BZV0	METSG 50S ribosomal protein L5	0	431427	481633	118801	164475	192177	0.8218	1.9	Null
F4BZV3	METSG 50S ribosomal protein L14	407028	384629	641501	177312	0	0	0.2905	8.1	Null
F4BZV7	METSG 30S ribosomal protein S3	0	241765	0	0	0	126739	0.9560	1.9	Null
F4BZW2	METSG 50S ribosomal protein L4	0	0	0	0	0	115879	NA	NA	Null
F4BZW3	METSG 50S ribosomal protein L3	0	0	0	0	0	79105	NA	NA	Null
F4BZY7	METSG Glutamate synthase (NADPH), homotetrameric	0	0	0	0	39340	0	NA	NA	Null
F4BZZ2	METSG Uncharacterized protein	483081	178712	184228	832758	406193	307361	0.5648	1.8	Null
F4C004	METSG Sulfite reductase, assimilatory-type	0	322597	542151	396656	261216	0	0.9560	1.3	Null
F4C011	METSG Arginine--tRNA ligase	0	0	0	0	33912	0	NA	NA	Null
F4C013	METSG DUF4139 domain-containing protein	0	609523	0	594966	0	317621	0.8970	1.5	Null
F4C017	METSG Uncharacterized protein	0	377359	0	0	0	0	0.2862	NA	Null
F4C019	METSG DUF4139 domain-containing protein	405980	1713340	2219364	1092593	827967	557356	0.5883	1.8	Null
F4C020	METSG Heat shock protein Hsp20	2222504	3781164	13216860	9625444	4987615	5970169	0.8698	1.1	Null
F4C031	METSG Putative snRNP Sm-like protein	5911121	2459987	3927700	1175359	2334247	1886504	0.2697	2.3	Null
F4C036	METSG Aspartate-semialdehyde dehydrogenase	0	160096	0	0	0	0	NA	NA	Null
F4C059	METSG 3-dehydroquinate synthase	0	536689	0	0	0	0	0.2517	NA	Null
F4C064	METSG Uncharacterized protein	2948793	0	854564	655901	28219981	0	NA	7.6	Null
F4C069	METSG PRC-barrel domain protein	0	0	0	0	136044	936922	0.0924	NA	Null
F4C070	METSG Peptidylprolyl isomerase	0	0	0	0	339406	0	0.2783	NA	Null
F4C071	METSG Peptidylprolyl isomerase	0	1445104	458711	398452	212977	1994124	0.8112	1.4	Null
F4C080	METSG Succinate-semialdehyde dehydrogenase [NADP+] (Ssdh)	0	146980	0	0	0	0	NA	NA	Null
F4C098	METSG Translation initiation factor 5A	917479	791677	6517601	307388	1434326	1680447	0.5796	2.4	Null
F4C0A1	METSG Uncharacterized protein	0	1216058	0	1015980	0	434935	0.9472	1.2	Null
F4C0D6	METSG S-adenosylmethionine synthase	0	101788	226002	270075	84311	0	0.9724	1.1	Null
F4C0E4	METSG Natural resistance-associated macrophage protein	0	0	0	918766	740813	0	0.0521	NA	Increase
F4C0E9	METSG Uncharacterized protein	0	129223	0	181027	270140	154769	0.4532	4.7	Null
F4C0F3	METSG DNA repair and recombination protein RadA	0	0	0	263400	0	0	NA	NA	Null
F4C0F9	METSG 3-isopropylmalate dehydratase small subunit	0	0	0	0	0	157077	NA	NA	Null
F4C0G4	METSG DNA-directed RNA polymerase subunit	0	97132	0	0	122502	0	NA	1.3	Null
F4C0G5	METSG DNA-directed RNA polymerase subunit A''	0	0	0	0	0	146319	NA	NA	Null
F4C0G7	METSG Probable transcription termination protein NusA	0	89849	257617	0	0	0	0.2925	NA	Null
F4C0H4	METSG Uncharacterized protein	0	361495	0	0	0	0	0.2917	NA	Null
F4C0H6	METSG Elongation factor Tu, domain 2 protein	0	0	119007	0	0	0	NA	NA	Null
F4C0I1	METSG Anthranilate synthase component 1	0	731261	0	0	0	0	0.3372	NA	Null
F4C0J6	METSG Uncharacterized protein	0	829248	546271	438688	405816	1333936	0.7372	1.6	Null
F4C0K3	METSG Acetyl-CoA decarbonylase/synthase complex subunit gamma	819316	4953068	2464227	3653332	2289007	3311242	0.7986	1.1	Null

F4C0K4	METSG Acetyl-CoA decarbonylase/synthase complex subunit delta	1356396	5071268	1515709	6120016	6517804	6969720	0.1310	2.5	Null
F4C0K5	METSG Nucleotide binding domain protein	0	386533	3911379	2755956	315218	1699292	0.9255	1.1	Null
F4C0K6	METSG Acetyl-CoA decarbonylase/synthase complex subunit beta	97820343	360187032	304217672	86882546	56941425	62481789	0.0242	3.7	Decrease
F4C0K7	METSG Acetyl-CoA decarbonylase/synthase complex subunit epsilon	1257343	168218	5911447	5815943	4104849	867715	0.7950	1.5	Null
F4C0K8	METSG Acetyl-CoA decarbonylase/synthase complex subunit alpha	1942113	141911	2376135	4464719	13336731	2963891	0.1758	4.7	Null
F4C0L1	METSG TPR-repeat protein	5240128	39942970	50121832	0	0	0	0.0000	NA	Decrease
F4C0L3	METSG Uncharacterized protein	151090	1136153	1077528	134528	86341	134383	0.0466	6.7	Decrease
F4C0L7	METSG Indolepyruvate oxidoreductase subunit IorA	0	0	266413	0	0	0	NA	NA	Null
F4C0L8	METSG Indolepyruvate ferredoxin oxidoreductase subunit beta	0	0	295107	119144	167625	0	0.9940	1.0	Null
F4C0N5	METSG Acylphosphatase	0	0	0	0	62860	0	NA	NA	Null
F4C0N9	METSG Uncharacterized protein	0	159216	332699	90743	54032	76210	0.7650	2.2	Null
F4C0P1	METSG Proteasome subunit beta	392539	0	0	215950	0	0	0.8698	1.8	Null
F4C0Q1	METSG Acetyl-coenzyme A synthetase	530795	1607245	1689059	3938792	2561795	0	0.7979	1.7	Null
F4C0R4	METSG Phosphoenolpyruvate synthase	0	0	0	190392	295849	0	0.2385	NA	Null
F4C0S9	METSG Uncharacterized protein	0	0	0	0	1149037	2133297	0.0155	NA	Increase
F4C0T2	METSG 50S ribosomal protein L12	1673255	1709856	3860368	1208467	2085481	3063987	0.9992	1.1	Null
F4C0T3	METSG 50S ribosomal protein L10	739988	1250831	2149090	1205236	853332	1583934	0.9962	1.1	Null
F4C0T4	METSG 50S ribosomal protein L1	0	1329499	651807	215021	301201	721683	0.9149	1.6	Null
F4C0T5	METSG 50S ribosomal protein L11	0	3292172	1942129	0	568280	96333	0.4387	7.9	Null
F4C0T6	METSG Transcription elongation factor Spt5	0	0	0	392953	383052	0	0.1500	NA	Null
F4C0T7	METSG Uncharacterized protein	2480584	1389948	0	11381184	14262674	21075908	0.1168	12.1	Null
F4C0U1	METSG S-inosyl-L-homocysteine hydrolase	0	0	0	0	0	335072	0.2613	NA	Null
F4C0U6	METSG Carboxymuconolactone decarboxylase family protein	314139	498788	577985	336104	454352	337714	0.8782	1.2	Null
P00131	DESVH Cytochrome c3	0	0	0	0	0	56444	NA	NA	Null
P04032	DESVH Cytochrome c-553	0	0	0	0	0	56008	NA	NA	Null
P07598	DESVH Periplasmic [Fe] hydrogenase large subunit	499305	537795	421081	0	0	734344	0.8718	2.0	Null
P17901	RUMCH Endoglucanase A	347395669	661818037	1107576055	1729154787	1783315761	524262802	0.2962	1.9	Null
P20418	DESVH Desulfoferrodoxin	0	538840	0	0	0	64793	0.6559	8.3	Null
P24092	DESVH High-molecular-weight cytochrome c	151747	0	238995	1824631	1612582	1911592	0.0260	13.7	Increase
P24931	DESVH Rubrerythrin	799175	1387808	1462646	1645162	1589215	1443366	0.4503	1.3	Null
P25472	RUMCH Endoglucanase D	935495	712149	3407939	8694952	7995135	3581063	0.0509	4.0	Increase
P30820	DESVH Nigerythrin	8571748	13914787	15676592	22373199	13826428	7248481	0.7979	1.1	Null
P31101	DESVH Hydroxylamine reductase	109088	0	0	1838151	2964890	755129	0.0193	51.0	Increase
P33389	DESVH Protein DVU	4688535	0	2334176	7995483	2837885	8292355	0.6205	2.7	Null
P33393	DESVH Protein DVU	0	0	0	0	257922	0	0.3023	NA	Null
P33394	DESVH Protein Rrf1	0	0	0	141965	84368	141942	0.0573	NA	Null
P35841	DESVH Chemoreceptor protein A	0	0	0	0	29159	7621309	NA	NA	Null
P37698	RUMCH Endoglucanase F	219615305923	158327767578	139885214042	115199966589	84270358951	96981042615	0.2983	1.7	Null
P37699	RUMCH Endoglucanase C	397418866	259977220	165326007	402935626	632137903	353580915	0.4226	1.7	Null
P37700	RUMCH Endoglucanase G	32488603359	29843133078	34259805022	37079659188	40500840412	26434277511	0.8054	1.1	Null
P45573	DESVH Sulfite reductase, dissimilatory-type subunit gamma	3865973	1277462	0	1680266	2303367	2199197	0.9560	1.2	Null
P45574	DESVH Sulfite reductase, dissimilatory-type subunit alpha	3670623	9485325	3999589	15541409	13240552	21055364	0.0351	2.9	Increase
P45575	DESVH Sulfite reductase, dissimilatory-type subunit beta	3466171	19133093	3443619	43566529	52858850	43074780	0.0151	5.4	Increase
P61522	DESVH Argininosuccinate synthase	0	0	0	1153086	1040572	0	0.0336	NA	Increase
P61654	DESVH 2-dehydro-3-deoxyphosphooctonate aldolase	0	0	0	242804	273737	175021	0.0049	NA	Increase
P61678	DESVH UDP-N-acetylmuramate--L-alanine ligase	0	1437061272	1726557	0	16938701	897444544	NA	1.6	Null
P61686	DESVH Translation initiation factor IF-1 1	55947226	912313	3105128	6870054	1357119	6317890	NA	4.1	Null
P61687	DESVH Translation initiation factor IF-1 2	0	293912	315062	215904	190144	771602	0.6338	1.9	Null
P61700	DESVH Alanine--tRNA ligase	0	0	0	860826	707881	511393	0.0000	NA	Increase
P61735	DESVH L-seryl-tRNA(Sec) selenium transferase	0	0	0	246281	0	0	NA	NA	Null
P61940	DESVH 6,7-dimethyl-8-ribityllumazine synthase	0	4269125	0	0	2664435	0	NA	1.6	Null
P62051	DESVH L-lactate dehydrogenase	7682894	0	352371	3179489	3197838	0	NA	1.3	Null
P62060	DESVH Arginine biosynthesis bifunctional protein ArgJ	0	282005	0	71862734	74837693	4149233	0.0001	534.9	Increase
P62214	DESVH Protein RecA	148478	687073	204666	3737994	6791820	6517156	0.0000	16.4	Increase
P62229	DESVH 30S ribosomal protein S16	15036845	10446671	9718253	8182335	7092665	6097816	0.3414	1.6	Null
P62353	DESVH 1-(5-phosphoribosyl)-5-[(5-phosphoribosylamino)methylideneamin	0	0	0	173069	0	0	NA	NA	Null
P62364	DESVH ATP phosphoribosyltransferase	0	144685	249568	210613	822158	281120	0.3832	3.3	Null
P62379	DESVH Histidine--tRNA ligase	1026907	2570300	0	85098	457334	1456027	0.8698	1.8	Null
P62386	DESVH Phosphoribosyl-AMP cyclohydrolase	0	0	0	306256	255666	0	0.2094	NA	Null
P62412	DESVH Phosphoglycerate kinase	120833	240004	153981	782150	878082	7104984	NA	17.0	Null
P62433	DESVH 50S ribosomal protein L11	0	1086286	1956881	69019452	53156059	130870781	0.0004	83.2	Increase
P62450	DESVH Imidazole glycerol phosphate synthase subunit HisF	0	0	0	0	2664435	2620848	0.0070	NA	Increase
P62457	DESVH Histidinol dehydrogenase	0	0	0	0	109860	2798104	NA	NA	Null
P62637	DESVH Protein-glutamate methylesterase/protein-glutamine glutaminase	846430	1608513	368412	3108982	6617571	3361523	0.0220	4.6	Increase
Q05805	DESVH Sulfite reductase, assimilatory-type	766954	1103133	1712906	1564386	1388246	5754471	0.2379	2.4	Null
Q06173	DESVH Periplasmic [NiFe] hydrogenase small subunit 1	0	0	0	252654	0	209553	0.2385	NA	Null
Q0PRM9	RUMCH Glucanase	3603472421	3326805298	2712946656	1438483877	1940765419	2008913993	0.2962	1.8	Null
Q0PRN0	RUMCH Cellulase	12501323	626313	512849	1565468	7423204	261832	0.8009	1.5	Null
Q0PRN1	RUMCH Cellulase	2256287	8172690	1958634	1234838	22799653	8512828	0.3371	2.6	Null
Q0PRN3	RUMCH Cellulase	4269511	6262382	9673459	49119427	17610051	6727494	0.0854	3.6	Null
Q0PRN4	RUMCH Glucanase	185728725	436869857	56157964	89435667	208242228	88614892	0.6310	1.8	Null
Q0PRN5	RUMCH Beta-xylanase	2513995007	4841159150	1646965475	4483768409	5232582163	5014535827	0.3224	1.6	Null
Q2FKW4	METHJ Multi-sensor signal transduction histidine kinase	0	0	0	1547831	605829	0	0.0368	NA	Increase
Q2FL75	METHJ Uncharacterized protein	0	0	0	0	64619	0	NA	NA	Null
Q2FLA8	METHJ Acetyl-coenzyme A synthetase	0	0	0	0	8597162	0	NA	NA	Null
Q2FLB2	METHJ DNA primase DnaG	212837	640514	1408310	670907	377426	0	0.7084	2.2	Null
Q2FLB4	METHJ UPF0235 protein Mhun	0	0	121417	3576277	134088	893816	0.0434	37.9	Increase
Q2FLD8	METHJ Argininosuccinate synthase	0	0	0	893935	0	0	NA	NA	Null
Q2FLE0	METHJ Carbamoyl-phosphate synthase small chain	0	658796	955771	34197	54664	60226	0.1977	10.8	Null
Q2FLH3	METHJ Chromosome partition protein Smc	2504682	4379439	0	0	0	0	0.0062	NA	Decrease
Q2FLJ4	METHJ Histidine kinase	0	287406	0	0	0	0	0.3129	NA	Null
Q2FLR0	METHJ ATPase, E1-E2 type	0	1448257849	0	267941	0	0	NA	5405.1	Null
Q2FLR9	METHJ Transposase, IS4 family	0	976630	0	0	0	0	NA	NA	Null
Q2FLW0	METHJ Uncharacterized protein	530230	0	0	0	0	0	0.2244	NA	Null
Q2FM11	METHJ Uncharacterized protein	0	0	292308	0	0	0	0.3111	NA	Null
Q2FM95	METHJ Exonuclease SbcC	0	279808	1168953	0	0	0	0.0846	NA	Null
Q2FMI6	METHJ Methyltranfer	62405	0	0	0	0	0	NA	NA	Null
Q2FMK1	METHJ HhH-GPD	160656	0	0	0	0	0	NA	NA	Null
Q2FMN4	METHJ Pyruvate kinase	0	0	72069	0	0	340370	0.6618	4.7	Null
Q2FMN6	METHJ UbiE/COQ5 methyltransferase	0	0	0	0	256893	0	0.3023	NA	Null
Q2FMQ6	METHJ Uncharacterized protein	0	314629	0	0	0	0	0.3047	NA	Null
Q2FMV7	METHJ Uncharacterized protein	0	0	0	0	0	114618	NA	NA	Null
Q2FMW7	METHJ Extracellular solute-binding protein, family 5	0	0	0	0	144800	0	NA	NA	Null
Q2FMX6	METHJ ABC-3	0	0	0	0	0	14616	NA	NA	Null
Q2FMY5	METHJ UspA	0	0	0	0	478877	0	0.2424	NA	Null
Q2FMZ1	METHJ Dihydroxy-acid dehydratase	2500928	0	0	0	0	0	NA	NA	Null
Q2FN61	METHJ Putative phosphoserine phosphatase	0	0	0	0	62277	0	NA	NA	Null
Q2FND5	METHJ PKD	0	0	0	628161	0	0	0.2232	NA	Null
Q2FNF8	METHJ Uncharacterized protein	0	0	0	2193451	0	0	NA	NA	Null
Q2FNH4	METHJ Pyruvate carboxylase subunit A	0	0	1371017	0	0	0	NA	NA	Null
Q2FNL0	METHJ Multi-sensor signal transduction histidine kinase	3307612	0	0	0	0	0	NA	NA	Null
Q2FNP8	METHJ Methyl-accepting chemotaxis sensory transducer	0	0	0	0	300167	0	0.2905	NA	Null
Q2FNX5	METHJ Multi-sensor signal transduction histidine kinase	3493920	0	0	0	2907223	633055	0.9843	1.0	Null
Q2FNX6	METHJ Ribosomal RNA large subunit methyltransferase E	0	326677	0	0	83887	0	0.7886	3.9	Null
Q2FNY9	METHJ Periplasmic binding protein	665898	0	0	0	0	0	0.1991	NA	Null
Q2FNZ4	METHJ McrBC 5-methylcytosine restriction system component-like protei	0	0	0	0	0	3516256	NA	NA	Null
Q2FP01	METHJ EVE domain-containing protein	0	0	0	0	295902	0	0.2905	NA	Null
Q2FP42	METHJ Radical SAM	0	479972	0	0	0	0	0.2633	NA	Null
Q2FP50	METHJ V-type ATP synthase subunit D	0	0	805921	0	0	0	NA	NA	Null
Q2FP51	METHJ V-type ATP synthase beta chain 1	136555	0	0	0	0	0	NA	NA	Null
Q2FP52	METHJ V-type ATP synthase alpha chain 1	319212536	656396	139417383	169015824	3086614	1121400	0.6052	2.7	Null
Q2FP59	METHJ Multi-sensor signal transduction histidine kinase	0	309189	0	0	0	671038	0.8229	2.2	Null

Q2FP71	METHJ Uncharacterized protein	0	0	39725885	411888	0	0	NA	96.4	Null
Q2FPA8	METHJ Uncharacterized protein	0	0	0	0	0	322223	0.2664	NA	Null
Q2FPB6	METHJ S-layer domain-like protein	8155687	0	0	0	0	0	NA	NA	Null
Q2FPF2	METHJ Heat shock protein 70	0	0	210120	0	0	0	NA	NA	Null
Q2FPI5	METHJ 8-oxoguanine DNA glycosylase-like protein	0	0	233703	0	0	0	NA	NA	Null
Q2FPK0	METHJ DNA-directed RNA polymerase subunit	9060258	0	81661579	0	0	0	0.0000	NA	Decrease
Q2FPK4	METHJ Phage integrase	0	0	0	183323	0	0	NA	NA	Null
Q2FPU9	METHJ Molybdenum cofactor guanylyltransferase	0	0	0	506863	0	0	0.2451	NA	Null
Q2FPY4	METHJ Methyltransf	0	0	373861	0	0	0	0.2879	NA	Null
Q2FPZ9	METHJ Holliday junction DNA helicase subunit RuvA	0	617571	0	0	0	404729	0.9782	1.5	Null
Q2FQ42	METHJ Coenzyme F420 hydrogenase/dehydrogenase beta subunit-like pro	0	0	0	70209	0	0	NA	NA	Null
Q2FQ54	METHJ 3-phosphoshikimate 1-carboxyvinyltransferase	0	0	0	0	0	28077	NA	NA	Null
Q2FQ55	METHJ Shikimate dehydrogenase (NADP(+))	9771065	2713708	0	0	0	0	0.0027	NA	Decrease
Q2FQ56	METHJ Proteasome-activating nucleotidase	136502	179823	132045	0	0	886277	NA	2.0	Null
Q2FQD6	METHJ Alanine--tRNA ligase	0	0	0	0	153863	0	NA	NA	Null
Q2FQE6	METHJ Uncharacterized protein	0	0	0	0	0	209467	NA	NA	Null
Q2FQS5	METHJ Glycosyl transferase, family 2	0	0	0	0	0	1557316	NA	NA	Null
Q2FQW8	METHJ Histone deacetylase superfamily	225246684	101708876	180113121	48835322	3523529	0	0.2379	9.7	Null
Q2FQZ8	METHJ Uncharacterized protein	0	3854930	985476	0	0	721683	0.6539	6.7	Null
Q2FR18	METHJ Glutamyl-tRNA(Gln) amidotransferase subunit E	0	0	0	3302592	0	0	NA	NA	Null
Q2FR40	METHJ Regulatory protein, Crp	0	69493	0	0	0	0	NA	NA	Null
Q2FR49	METHJ AMP-dependent synthetase and ligase	0	1478198	0	0	0	0	NA	NA	Null
Q2FRC9	METHJ Formylmethanofuran dehydrogenase, subunit A	0	0	0	0	132935	0	NA	NA	Null
Q2FRD0	METHJ Formylmethanofuran dehydrogenase	0	0	0	2074232	0	0	NA	NA	Null
Q2FRE5	METHJ Tetratricopeptide TPR	0	0	0	0	0	320591	0.2664	NA	Null
Q2FRG8	METHJ Transcriptional regulator, ArsR family	0	0	3905256	0	73408	0	NA	53.2	Null
Q2FRH1	METHJ Methyl-accepting chemotaxis sensory transducer with Pas/Pac sen	0	0	0	217239	0	0	NA	NA	Null
Q2FRJ9	METHJ Formate dehydrogenase, beta subunit (F420)	0	11362271	0	0	0	0	NA	NA	Null
Q2FRL9	METHJ Formylmethanofuran dehydrogenase, subunit A	0	0	0	0	162979	0	NA	NA	Null
Q2FRQ4	METHJ CoA enzyme activase	1525188	285845	0	174871	273189	0	0.5538	4.0	Null
Q2FRW1	METHJ Uncharacterized protein	0	8141123	13713286	0	2614633	3494464	0.7349	3.6	Null
Q2FRZ8	METHJ Uncharacterized protein	0	0	0	0	1064529	0	NA	NA	Null
Q2FSJ7	METHJ Uncharacterized protein	38532667	0	37481484	0	0	0	0.0002	NA	Decrease
Q2FSL5	METHJ Uncharacterized protein	53646968	173750	0	140200	0	0	NA	383.9	Null
Q2FSM2	METHJ Uncharacterized protein	0	0	0	0	0	272269	0.2846	NA	Null
Q2FSN2	METHJ Methyl-coenzyme M reductase operon protein C	0	0	0	19817	0	0	NA	NA	Null
Q2FSV3	METHJ Formate dehydrogenase, beta subunit (F420)	0	0	0	0	0	421727	0.2109	NA	Null
Q2FT52	METHJ Thiol-driven fumarate reductase, flavoprotein subunit	0	0	5511255104	1822955453	555369	0	NA	3.0	Null
Q2FT95	METHJ Deoxyhypusine synthase	0	0	0	0	0	71563	NA	NA	Null
Q2FTH3	METHJ Geranylgeranyl-diphosphate synthase / farnesyl-diphosphate synth	1753260	0	0	183622	0	0	NA	9.5	Null
Q2FTL9	METHJ Thermosome subunit	0	0	0	60767	0	0	NA	NA	Null
Q2FTM1	METHJ tRNA(Ile2) 2-agmatinylcytidine synthetase TiaS	0	0	753050	0	0	0	NA	NA	Null
Q2FTP2	METHJ Ribonuclease J	0	0	0	0	0	3374184	NA	NA	Null
Q2FTP5	METHJ Tetratricopeptide TPR	420845	0	0	0	0	0	0.2494	NA	Null
Q2FTS5	METHJ AMP phosphorylase	0	0	0	0	8030658	0	NA	NA	Null
Q2FTU9	METHJ OBG-type G domain-containing protein	0	0	0	0	468109	0	0.2443	NA	Null
Q2FTX8	METHJ Tetratricopeptide TPR	0	1798107	0	0	23527150	0	NA	13.1	Null
Q2FU43	METHJ Nucleic acid binding, OB-fold, tRNA/helicase-type	0	0	0	0	641208	377294	0.0948	NA	Null
Q2FU46	METHJ Cell surface protein	0	0	0	0	1039628	0	NA	NA	Null
Q2FU49	METHJ Uncharacterized protein	2096398	0	0	0	0	0	NA	NA	Null
Q2FU82	METHJ Uncharacterized protein	0	27453	0	0	0	0	NA	NA	Null
Q2FU87	METHJ Histidine kinase	0	0	0	0	6661089	2032107	0.0039	NA	Increase
Q2FUB5	METHJ Phosphoribulokinase	0	0	0	1142250	0	0	NA	NA	Null
Q2FUC4	METHJ Pyruvate ferredoxin oxidoreductase, alpha subunit	0	221542	0	0	0	0	NA	NA	Null
Q2FUF5	METHJ Uncharacterized protein	0	0	0	0	0	284644	0.2808	NA	Null
Q2FUN6	METHJ N-acetylneuraminate synthase	0	0	0	827717	0	0	NA	NA	Null
Q3V891	DESVH Pyridoxine 5'-phosphate synthase	0	227259	234969	443471	210596	263867	0.6321	2.0	Null
Q3V892	DESVH Acetolactate synthase	0	0	0	187764	0	639386	0.1239	NA	Null
Q46582	DESVH Protein DsvD	0	0	0	3973043	0	2981690	0.0048	NA	Increase
Q68GY5	RUMCH 60 kDa chaperonin (Fragment)	0	499282	0	0	0	0	0.2596	NA	Null
Q725H4	DESVH Glucose-6-phosphate isomerase	0	0	0	0	31387	0	NA	NA	Null
Q725H5	DESVH Histidine kinase	0	7037989	0	9187662	144208	90603	NA	1.3	Null
Q725H8	DESVH Pyrazinamidase/nicotinamidase	0	0	0	0	0	94404	NA	NA	Null
Q725I1	DESVH Glutamate-1-semialdehyde 2,1-aminomutase	134856	441893	0	844272	276723	949583	0.3821	3.6	Null
Q725I2	DESVH Siroheme decarboxylase beta subunit	1894985	3071118	1988507	651899	817760	1217323	0.1564	2.6	Null
Q725I7	DESVH ABC transporter, periplasmic substrate-binding protein	1381505	2066714	6521868	12060272	34691204	7123922	0.0300	5.4	Increase
Q725I8	DESVH ABC transporter, ATP-binding protein	0	0	0	157567	92577	0	NA	NA	Null
Q725J7	DESVH UDP-glucose/GDP-mannose dehydrogenase family protein	0	72963	0	1021878	1273701	5781175	0.0005	110.7	Increase
Q725K4	DESVH Uncharacterized protein	6383601	3689425	948882	10639831	4919719	7960171	0.4254	2.1	Null
Q725K7	DESVH DNA-binding protein HU	4077773	4376797	4406353	1382288	1017884	529054	0.0006	4.4	Decrease
Q725K9	DESVH Adenylosuccinate synthetase	0	0	0	0	260853	127006	0.2583	NA	Null
Q725L3	DESVH Recombination protein RecR	0	0	0	0	169594	97998	0.2928	NA	Null
Q725L4	DESVH Nucleoid-associated protein DVU	578884	209563	0	0	239492	0	0.7464	3.3	Null
Q725L5	DESVH DNA polymerase III subunit gamma/tau	0	0	0	629758	0	0	0.2232	NA	Null
Q725L6	DESVH Branched-chain-amino-acid aminotransferase	0	0	0	390922	0	148433	0.2187	NA	Null
Q725L9	DESVH GTPase Der	0	0	0	1372707	1410091	0	0.0229	NA	Increase
Q725M0	DESVH DNA-binding domain, excisionase family	0	0	0	179212	0	0	NA	NA	Null
Q725M4	DESVH Transcription elongation factor GreA	2087486	7980509	7119244	13794348	6744160	8513248	0.3270	1.7	Null
Q725M7	DESVH DNA-directed RNA polymerase subunit omega	838046	0	742017	590015	349380	0	0.8463	1.7	Null
Q725N2	DESVH Glutamine synthetase, type I	0	0	0	74420	138086	0	NA	NA	Null
Q725N5	DESVH DNA topoisomerase 1	0	0	0	0	194220	153559	0.2688	NA	Null
Q725N7	DESVH Uncharacterized protein	0	0	0	0	41544	0	NA	NA	Null
Q725P0	DESVH Zinc resistance-associated protein homolog	51848546	36645625	28599950	16490330	16627033	18027052	0.1439	2.3	Null
Q725P2	DESVH Histidine kinase	0	28702	78514	0	0	0	NA	NA	Null
Q725P5	DESVH Vitamin B12-dependent ribonucleotide reductase	0	406230	0	6704546	883995	596265	0.1436	20.1	Null
Q725Q1	DESVH Dihydroxy-acid dehydratase	1389926	1365438	159568	1621266	1574480	2623232	0.4862	2.0	Null
Q725Q7	DESVH Aspartate--tRNA(Asp/Asn) ligase	0	0	0	161163	199251	264035	0.0085	NA	Increase
Q725R1	DESVH Sun protein	0	0	0	0	39021	0	NA	NA	Null
Q725R2	DESVH DUF1992 domain-containing protein	0	174951	0	0	0	0	NA	NA	Null
Q725R3	DESVH ADP-heptose synthase, putative	0	0	0	0	65560	0	NA	NA	Null
Q725R4	DESVH ParB family protein	1163182	471955	30156938	430473	128700	213139	0.0037	41.2	Decrease
Q725R8	DESVH NAD-dependent epimerase/dehydratase family protein	0	191732	0	144498	90098	0	0.9573	1.2	Null
Q725R9	DESVH SPFH domain/Band 7 family protein	0	0	0	938482	204698	0	0.1028	NA	Null
Q725S1	DESVH Coenzyme A biosynthesis bifunctional protein CoaBC	0	0	0	0	84525	108850	NA	NA	Null
Q725S2	DESVH Lipoprotein, putative	906802	951866	244007	935321	902671	658377	0.8496	1.2	Null
Q725S3	DESVH S-adenosylmethionine:tRNA ribosyltransferase-isomerase	0	0	0	125932	188529	126424	0.0279	NA	Increase
Q725S5	DESVH Pyruvate flavodoxin/ferredoxin oxidoreductase, thiamine diP-bindi	0	0	0	1614049	916909	0	0.0274	NA	Increase
Q725S6	DESVH Pyruvate ferredoxin/flavodoxin oxidoreductase, beta subunit, puta	0	0	0	685941	127608	941842	0.0004	NA	Increase
Q725S7	DESVH Pyruvate ferredoxin/flavodoxin oxidoreductase family protein	737402	1863227	2180743	1677814	1342794	2312424	0.7654	1.1	Null
Q725S9	DESVH Uncharacterized protein	0	0	0	0	65960	0	NA	NA	Null
Q725T7	DESVH Potassium-transporting ATPase ATP-binding subunit	0	91827	0	0	0	0	NA	NA	Null
Q725V3	DESVH Conserved domain protein	0	0	0	140570	846643	0	0.1193	NA	Null
Q725V6	DESVH Bifunctional protein PutA	617796	802620	1770747	2959727	1853425	550079	0.5270	1.7	Null
Q725V9	DESVH Dihydroorotate dehydrogenase	0	0	0	0	310892	259813	0.1901	NA	Null
Q725W1	DESVH Peptidase, U32 family	0	0	0	0	223774	138369	0.2652	NA	Null
Q725W7	DESVH UPF0173 metal-dependent hydrolase DVU	0	0	0	131965	297045	0	0.2575	NA	Null
Q725Y0	DESVH Aldehyde dehydrogenase (NADP) family protein	0	361460	0	93918	417699	189438	0.7816	1.9	Null
Q725Z2	DESVH ADP-ribosylglycohydrolase family protein	0	163944	49601246	1490406	18304454	26121944	0.9682	1.1	Null
Q725Z5	DESVH Peptidase/PDZ domain protein	102441	311186	344154	674388	870662	1189372	0.0379	3.6	Increase
Q725Z9	DESVH Uncharacterized protein	0	0	0	139867	179950	235154	0.0132	NA	Increase
Q726A0	DESVH Uncharacterized protein	0	0	0	1162916	327860	0	0.0681	NA	Null
Q726A1	DESVH TPR domain protein	0	0	0	515526	1614551	655506	0.0000	NA	Increase

Q726A2	DESVH Cytochrome d ubiquinol oxidase, subunit I	0	0	0	125447	373827	391775	0.0029	NA	Increase
Q726A8	DESVH Tartrate dehydratase beta subunit, putative	0	293080	0	0	0	0	0.3129	NA	Null
Q726B2	DESVH Phenylacetate-coenzyme A ligase, putative	4609736	0	199504	2627469	483400	2760120	0.9682	1.2	Null
Q726B7	DESVH Rubredoxin-oxygen oxidoreductase	0	0	0	70945	1314725	0	0.0905	NA	Null
Q726B8	DESVH Phosphoribosylformylglycinamidine synthase subunit PurL	0	0	0	217126	118426	227076	0.0132	NA	Increase
Q726C1	DESVH IMP cyclohydrolase, putative	623445	766598	1048844	2600997	3207419	2348635	0.0005	3.3	Increase
Q726C3	DESVH Flagellar synthesis regulator FleN	0	0	0	156434	0	0	NA	NA	Null
Q726C5	DESVH Chemotaxis protein CheY	0	0	0	858220	204625	0	0.1121	NA	Null
Q726C9	DESVH Fumarate reductase, flavoprotein subunit	0	0	0	0	0	202285	NA	NA	Null
Q726D3	DESVH UDP-N-acetylglucosamine 1-carboxyvinyltransferase	0	0	0	0	103571	0	NA	NA	Null
Q726D4	DESVH DNA internalization-related competence protein ComEC/Rec2	0	0	0	0	0	135861	NA	NA	Null
Q726D8	DESVH Response regulator	0	0	0	336167	338674	458087	0.0003	NA	Increase
Q726E1	DESVH Pyridine nucleotide-disulfide oxidoreductase	0	8019778	0	2528106	2078507	14109986	NA	2.3	Null
Q726E6	DESVH RNB-like family protein	0	232300	0	0	0	0	NA	NA	Null
Q726E7	DESVH Phosphoribosylaminoimidazolecarboxamide formyltransferase, pu	114920	0	0	2592252	3073307	2722205	0.0041	73.0	Increase
Q726F2	DESVH Glutamine--fructose-6-phosphate aminotransferase [isomerizing]	378176	0	0	507913	1673664	1761629	0.2917	10.4	Null
Q726F3	DESVH Methyl-accepting chemotaxis protein DcrH	0	0	0	0	0	36293	NA	NA	Null
Q726F4	DESVH dITP/XTP pyrophosphatase	0	0	317118	491384	384037	2076421	0.2575	9.3	Null
Q726F8	DESVH 30S ribosomal protein S1	1203259	6174777	1677460	16746818	7012423	22176178	0.0247	5.1	Increase
Q726G1	DESVH 6-phosphofructo-2-kinase/fructose-2, 6-biphosphatase	0	0	0	0	0	100173	NA	NA	Null
Q726G6	DESVH Sigma-54 dependent transcriptional regulator	13947407	0	0	0	19851015	154536	NA	1.4	Null
Q726G8	DESVH Capsular polysaccharide transport protein, putative	487671	114327	0	803866	837697	6192515	NA	13.0	Null
Q726H2	DESVH Nitroreductase family protein	0	0	0	372469	189626	0	0.2171	NA	Null
Q726H4	DESVH Glycerol kinase	0	0	0	0	1388246	4849202	0.0060	NA	Increase
Q726I1	DESVH Paraquat-inducible protein B	0	0	0	0	65028	37131	NA	NA	Null
Q726I2	DESVH Lipoprotein, putative	355950	0	0	0	0	0	0.2652	NA	Null
Q726I8	DESVH AMP-binding enzyme family protein	0	0	0	0	0	189999	NA	NA	Null
Q726J1	DESVH Peptide chain release factor 3	464299	0	3896371	115251	106216	0	0.2080	19.7	Null
Q726J3	DESVH 3-deoxy-manno-octulosonate cytidylyltransferase	0	0	0	0	94216	249356	0.2652	NA	Null
Q726J4	DESVH Carbamoyl-phosphate synthase small chain	0	0	0	757151	616022	730265	0.0000	NA	Increase
Q726K3	DESVH Peptidoglycan-associated protein	0	5470927	215394	682767	110537	176269	0.4138	5.9	Null
Q726K4	DESVH Tol-Pal system protein TolB	380778	287214	87414	1652440	781642	1424965	0.0379	5.1	Increase
Q726K5	DESVH Uncharacterized protein	0	0	0	0	596233	293859	0.1162	NA	Null
Q726K6	DESVH TonB protein, putative	0	0	0	481265	395992	0	0.1275	NA	Null
Q726K7	DESVH Biopolymer transport protein, ExbD/TolR family	0	0	0	0	84887	0	NA	NA	Null
Q726K8	DESVH TolQ protein	0	0	0	0	178875	181068	0.2633	NA	Null
Q726L0	DESVH Outer membrane efflux protein	730922	734743	598051	610007	2075863	2792014	0.1939	2.7	Null
Q726L6	DESVH Outer membrane protein, OMPP1/FadL/TodX family	0	0	0	255452	142897	204698	0.0101	NA	Increase
Q726L8	DESVH ATP-dependent RNA helicase, DEAD/DEAH box family	0	0	0	310148	617885	43633	0.0092	NA	Increase
Q726M4	DESVH Methyl-accepting chemotaxis protein	0	0	0	207952	176201	205739	0.0088	NA	Increase
Q726M6	DESVH Transcriptional regulator, putative	13612529	10782525	18982344	13022634	627181	4421835	0.4260	2.4	Null
Q726N5	DESVH Oxidoreductase, FAD/iron-sulfur cluster-binding domain protein	0	0	0	3718547	437822	2672157	0.0000	NA	Increase
Q726N6	DESVH Putative pre-16S rRNA nuclease	0	0	0	364709	0	0	0.2813	NA	Null
Q726P0	DESVH DNA-binding protein	0	0	0	0	340496	137346	0.2319	NA	Null
Q726P1	DESVH AMP-binding enzyme family protein	0	158547	0	378680	700553	349372	0.2416	9.0	Null
Q726P4	DESVH Histidine kinase	0	0	0	0	0	147347	NA	NA	Null
Q726P7	DESVH Signal recognition particle receptor FtsY	0	0	0	253822	262664	0	0.2319	NA	Null
Q726Q1	DESVH Ribonuclease, Rne/Rng family	0	75821	113420	334567	252760	320653	0.2105	4.8	Null
Q726Q3	DESVH tRNA (adenine(58)-N(1))-methyltransferase TrmI	0	0	0	444033	812145	591284	0.0000	NA	Increase
Q726Q8	DESVH Aspartate-semialdehyde dehydrogenase	833917	5641560	8382263	4751097	4879212	13332699	0.5633	1.5	Null
Q726R4	DESVH Lipoprotein, putative	0	423877	637300	0	0	0	0.1186	NA	Null
Q726R9	DESVH Rhodanese-like domain protein	0	0	0	90085	118219	0	NA	NA	Null
Q726S1	DESVH Methyl-accepting chemotaxis protein, putative	287306	0	0	485628	1459164	0	0.6081	6.8	Null
Q726S3	DESVH Iron-sulfur cluster-binding protein	3918117	2504112	2632529	27522085	27346139	39111203	0.0000	10.4	Increase
Q726S4	DESVH LUD	3641597	0	0	6605184	164971	1275134	0.8611	2.2	Null
Q726S5	DESVH DRTGG domain-containing protein	0	0	0	63108	202113	43917	0.1202	NA	Null
Q726S6	DESVH Acetate kinase	183323	780034	175089	3201124	3158864	1487178	0.0044	6.9	Increase
Q726S7	DESVH Phosphate acetyltransferase	0	4513403	372314	3099935	5137795	1822966	0.6788	2.1	Null
Q726S8	DESVH Iron-sulfur cluster-binding protein	0	0	201836	333275	0	356544	0.7040	3.4	Null
Q726S9	DESVH Glycolate oxidase, subunit GlcD	9839897	26649050	16135520	11673324	23363824	52205090	0.4322	1.7	Null
Q726T0	DESVH L-lactate permease	0	0	0	0	0	113961	NA	NA	Null
Q726T1	DESVH Pyruvate:ferredoxin oxidoreductase	8103345	7983559	18648841	65630892	33040467	22189838	0.0242	3.5	Increase
Q726T5	DESVH HDIG domain protein	0	0	0	0	1052078	0	NA	NA	Null
Q726T8	DESVH Radical SAM domain protein	0	0	0	0	188842	0	NA	NA	Null
Q726V1	DESVH Aminotransferase, DegT/DnrJ/EryC1/StrS family	0	0	0	0	88390	0	NA	NA	Null
Q726V4	DESVH Conserved domain protein	0	0	0	0	0	42651	NA	NA	Null
Q726W0	DESVH NAD-dependent epimerase/dehydratase family protein	743814	0	0	0	82351	0	NA	9.0	Null
Q726W3	DESVH Glycosyl transferase, group 1/2 family protein	3382180	124181191	0	0	0	10367604	NA	12.3	Null
Q726W4	DESVH Glycosyl transferase, group 2 family protein	0	252138	0	0	0	0	NA	NA	Null
Q726W6	DESVH Molybdopterin molybdenumtransferase	0	0	0	1037851	752673	1116780	0.0000	NA	Increase
Q726W8	DESVH Phage shock protein A	868596	773411	0	0	121612	0	0.3646	13.5	Null
Q726X1	DESVH 3-isopropylmalate dehydrogenase	0	0	0	527393	569790	57615	0.0034	NA	Increase
Q726X2	DESVH DUF89 domain-containing protein	0	485233	399580	618081	0	0	0.9448	1.4	Null
Q726X3	DESVH 3-isopropylmalate dehydratase small subunit	970129	1478198	4839614	8707586	4575494	144187	0.6657	1.8	Null
Q726X4	DESVH 3-isopropylmalate dehydratase large subunit	0	0	0	1539793	1238838	1031880	0.0000	NA	Increase
Q726X5	DESVH 2-isopropylmalate synthase	2293222	2968293	747484	2372068	1550155	310436	0.7596	1.4	Null
Q726X8	DESVH Hydrolase, haloacid dehalogenase-like family	0	0	351635	735175	0	173954	0.7979	2.6	Null
Q726Y0	DESVH Uncharacterized protein	248516	609085	685889	546996	4458817	105371	0.3222	3.3	Null
Q726Y3	DESVH Integration host factor, beta subunit	0	56464	0	523322	0	0	0.5955	9.3	Null
Q726Y6	DESVH Acetyltransferase, GNAT family	0	1016375	0	0	0	0	NA	NA	Null
Q726Y7	DESVH Acetyl-coenzyme A synthetase	0	0	0	0	400353	0	0.2621	NA	Null
Q726Y8	DESVH Histidine kinase	0	0	0	0	166873	0	NA	NA	Null
Q726Z0	DESVH Response regulator	0	1512903	3860368	1645839	1559560	1101177	0.9724	1.2	Null
Q727A5	DESVH Glutamine--tRNA ligase	131591	228018	232688	978593	0	613551	0.5631	2.7	Null
Q727A8	DESVH Anaerobic ribonucleoside-triphosphate reductase, putative	0	0	0	238431	913753	0	0.0935	NA	Null
Q727B3	DESVH Adenylosuccinate lyase	0	0	0	0	158734	133335	0.2855	NA	Null
Q727B7	DESVH HcyBio domain-containing protein	459414	1224478	197954	884805	4362224	91095	0.4387	2.8	Null
Q727B8	DESVH TPR domain protein/response regulator receiver domain protein	0	113404	225311	226987	264479	296350	0.5401	2.3	Null
Q727C0	DESVH 2,3-bisphosphoglycerate-dependent phosphoglycerate mutase	127464	249127	187294	158816	477906	358052	0.4473	1.8	Null
Q727C6	DESVH DNA-directed RNA polymerase subunit beta'	1062846	1506599	650607	27778016	15532569	30071183	0.0000	22.8	Increase
Q727C7	DESVH DNA-directed RNA polymerase subunit beta	0	2661579	7379592	75550271	44606111	53585055	0.0577	17.3	Null
Q727C8	DESVH 50S ribosomal protein L7/L12	4844549	5088038	2468091	3100679	3695202	582105	0.5747	1.7	Null
Q727C9	DESVH 50S ribosomal protein L10	1787571	4054225	7570441	12434654	8945512	10390782	0.1116	2.4	Null
Q727D0	DESVH 50S ribosomal protein L1	4388859	2813819	7954366	1281745	2222438	2460173	0.2324	2.5	Null
Q727D2	DESVH Transcription termination/antitermination protein NusG	0	0	0	1669821	358731	930591	0.0000	NA	Increase
Q727D3	DESVH Protein translocase subunit SecE	295109	157974	142912	0	0	0	0.0165	NA	Decrease
Q727D5	DESVH Elongation factor Tu	5121160	4236539	3737885	72421472	61736126	56020746	0.0000	14.5	Increase
Q727E1	DESVH Peptide chain release factor 1	0	229798	0	1206198	998463	1739113	0.0950	17.2	Null
Q727E2	DESVH Lipoprotein, putative	0	0	0	0	0	130847	NA	NA	Null
Q727E4	DESVH Hemolysin A	0	0	0	26974	0	0	NA	NA	Null
Q727F2	DESVH HD domain protein	0	230461	0	327052	104543	0	0.8713	1.9	Null
Q727F7	DESVH Acetyltransf	0	0	0	322662	355213	0	0.1737	NA	Null
Q727F8	DESVH MEMO1 family protein DVU	0	269009	216102	328543	461742	56431	0.7493	1.7	Null
Q727G1	DESVH Sigma-54 dependent transcriptional regulator	0	0	72028	231363	136720	232289	0.2552	8.3	Null
Q727G3	DESVH GTP cyclohydrolase FolE2	0	0	0	555542	678559	187494	0.0003	NA	Increase
Q727G4	DESVH Putative nickel-responsive regulator	0	161243	126818	2002218	447169	284736	0.0960	9.5	Null
Q727H1	DESVH M18 family aminopeptidase	0	0	0	188962	91034	0	0.3013	NA	Null
Q727H6	DESVH Prim	0	0	431448	0	0	0	0.2721	NA	Null
Q727I5	DESVH Uncharacterized protein	120112	574139	33848064	18291955	0	0	NA	1.9	Null
Q727J7	DESVH Tail tube protein, putative	0	0	0	0	96061	0	NA	NA	Null
Q727K4	DESVH Tail protein, putative	0	117128	0	0	0	0	NA	NA	Null
Q727L0	DESVH HIT family protein	0	0	584929	0	0	0	0.2422	NA	Null

Q727L2	DESVH Very short patch repair endonuclease	3070896	0	0	0	0	0	NA	NA	Null
Q727L8	DESVH HSDR	0	0	0	2635598	527838	494638	0.0000	NA	Increase
Q727L9	DESVH HTH cro/C1-type domain-containing protein	222179	0	221061	208799	228019	0	0.9868	1.0	Null
Q727N3	DESVH TRAP transporter solute receptor DctP	0	0	0	0	270855	61785	0.2781	NA	Null
Q727N8	DESVH Multidrug resistance protein	0	0	0	27469	0	130410	NA	NA	Null
Q727N9	DESVH Efflux pump membrane transporter	0	0	0	0	846643	0	NA	NA	Null
Q727P3	DESVH Formate dehydrogenase 2 subunit alpha (cytochrome c-553)	0	0	0	0	0	32711	NA	NA	Null
Q727P4	DESVH Formate dehydrogenase 2 subunit beta (cytochrome c-553)	0	4522846	0	0	0	0	NA	NA	Null
Q727R1	DESVH Ion-translocating oxidoreductase complex subunit G	0	0	0	801017	780230	368604	0.0000	NA	Increase
Q727R3	DESVH Electron transport complex protein RnfC, putative	0	0	0	0	469105	0	0.2443	NA	Null
Q727R7	DESVH Transcriptional regulator, ArsR family	0	0	0	0	3685387	0	NA	NA	Null
Q727R8	DESVH Conserved domain protein	0	189905	0	0	320300	734344	0.5573	5.6	Null
Q727S1	DESVH Dehydrogenase, FMN-dependent family	226669	191623	0	382626	302585	355488	0.5920	2.5	Null
Q727T0	DESVH CBS domain protein/ACT domain protein	223028	0	636724	937817	2145857	239549	0.4153	3.9	Null
Q727T2	DESVH Uncharacterized protein	429596	268950	47815801	0	1774215	194739	NA	24.6	Null
Q727T3	DESVH Tim44 domain-containing protein	0	0	0	83491	40458	0	NA	NA	Null
Q727T4	DESVH Response regulator	0	0	0	272165	474215	11802008	NA	NA	Null
Q727T5	DESVH Uncharacterized protein	1218373	2282144	3188357	3024420	452868	2197463	0.9585	1.2	Null
Q727T7	DESVH Iron-sulfur flavoprotein, putative	0	0	0	0	48876	0	NA	NA	Null
Q727T9	DESVH Metallo-beta-lactamase family protein	0	0	0	217175	566938	201378	0.0018	NA	Increase
Q727U1	DESVH TPR/GGDEF domain protein	0	0	0	0	0	7577	NA	NA	Null
Q727U7	DESVH Cyclic dehypoxanthine futalosine synthase	0	0	0	112409	141526	0	NA	NA	Null
Q727U8	DESVH Aminodeoxyfutalosine synthase	0	0	0	117502	0	0	NA	NA	Null
Q727U9	DESVH 1,4-dihydroxy-6-naphtoate synthase	0	0	0	0	248034	0	NA	NA	Null
Q727W0	DESVH High-affinity branched-chain amino acid ABC transporter, perisplas	0	0	0	0	0	242352	0.2950	NA	Null
Q727W6	DESVH Methyl-accepting chemotaxis protein	573282	1060154	526669	2850043	3669443	1630051	0.0061	3.8	Increase
Q727Y1	DESVH Tail protein, putative	0	0	0	81502	0	0	NA	NA	Null
Q727Y3	DESVH Phage tail tape measure protein, TP901 family, putative	0	0	0	141561	58202	72250	0.1230	NA	Null
Q727Y4	DESVH Uncharacterized protein	0	0	0	0	409349	0	0.2595	NA	Null
Q727Y5	DESVH Uncharacterized protein	0	0	0	999314	0	311847	0.0773	NA	Null
Q727Y7	DESVH Uncharacterized protein	0	0	215527	733856	717160	385705	0.2663	8.5	Null
Q727Z9	DESVH Phage uncharacterized protein	0	0	318346	0	0	0	0.3007	NA	Null
Q728A7	DESVH Uncharacterized protein	0	0	0	0	199161	199183	0.2524	NA	Null
Q728B3	DESVH Uncharacterized protein	0	0	0	0	86905	126614	NA	NA	Null
Q728B5	DESVH Bacteriophage DNA transposition B protein	0	0	0	0	0	232413	0.2991	NA	Null
Q728B8	DESVH Peptidase, S24 family	0	0	0	166595	202540	160617	0.0149	NA	Increase
Q728C4	DESVH Histidine kinase	0	0	0	0	330894	0	0.2813	NA	Null
Q728D0	DESVH Anaerobic glycerol-3-phosphate dehydrogenase, subunit A, truncat	0	0	0	43516	0	25149	NA	NA	Null
Q728D2	DESVH Ribonuclease Y	336536	1013510	1497399	357946	451988	1232027	0.9019	1.4	Null
Q728D4	DESVH Conserved domain protein	0	0	0	652553	0	115551	0.1636	NA	Null
Q728D5	DESVH Bifunctional protein GlmU	1881292	2162141	1914729	478075	650490	341314	0.0014	4.1	Decrease
Q728F1	DESVH Uncharacterized protein	0	0	182652	0	0	180567	0.9582	1.0	Null
Q728F6	DESVH Endoribonuclease, L-PSP family	0	0	0	0	0	664708	0.1914	NA	Null
Q728G0	DESVH Chaperone protein HtpG	4180180	339972	4230384	8229403	5932727	915943	0.7295	1.7	Null
Q728G1	DESVH Alanyl-tRNA synthetase family protein, putative	0	0	0	35480	167735	119028	0.0871	NA	Null
Q728G3	DESVH Uncharacterized protein	0	0	0	0	246887	0	NA	NA	Null
Q728G7	DESVH HlyD	0	0	167634	0	0	0	NA	NA	Null
Q728H0	DESVH Transcriptional regulator, putative	0	0	0	0	46188	0	NA	NA	Null
Q728I4	DESVH Uncharacterized protein	0	0	0	577835	993591	409504	0.0000	NA	Increase
Q728J2	DESVH Uncharacterized protein	1380955	0	0	0	0	0	NA	NA	Null
Q728J4	DESVH Chemotaxis MotB protein, putative	0	0	0	0	101153	0	NA	NA	Null
Q728L2	DESVH Tail fiber assembly protein, putative	0	0	0	3103940	2471451	0	0.0072	NA	Increase
Q728L3	DESVH Sensory box protein	0	0	0	1862364	414286	0	0.0375	NA	Increase
Q728L7	DESVH Energy-dependent translational throttle protein EttA	0	1742950	0	787113	1197122	1370772	0.7765	1.9	Null
Q728L8	DESVH Methyl-accepting chemotaxis protein	0	504971	0	41246	0	0	0.5466	12.2	Null
Q728M1	DESVH Transcriptional regulator, TetR family	0	289003	0	0	1245063	1280088	0.4319	8.7	Null
Q728M3	DESVH Response regulator	142452	0	0	107859	499769	6457162	0.0627	49.6	Null
Q728M4	DESVH TPR domain protein	0	0	0	116687	123498	854624	0.0051	NA	Increase
Q728M6	DESVH DNA-binding response regulator, LuxR family	0	0	0	0	0	62305	NA	NA	Null
Q728N1	DESVH Ferrous iron transport protein A, putative	0	0	0	3851317	132158	0	0.0248	NA	Increase
Q728N2	DESVH Ferrous iron transport protein B	0	4059530	0	532339	603386	2486682	NA	1.1	Null
Q728N4	DESVH Peptidyl-prolyl cis-trans isomerase	0	0	0	1200190	0	2139726	0.0154	NA	Increase
Q728P4	DESVH Adenosylmethionine-8-amino-7-oxononanoate aminotransferase	0	15742	0	0	0	0	NA	NA	Null
Q728P6	DESVH Bifunctional ligase/repressor BirA	0	0	0	441837	0	440168	0.1165	NA	Null
Q728P9	DESVH DUF2156 domain-containing protein	0	0	0	164672	0	0	NA	NA	Null
Q728Q1	DESVH Glutamate--tRNA ligase	0	0	0	1083289	812307	547744	0.0000	NA	Increase
Q728Q5	DESVH FMN-dependent NADH:quinone oxidoreductase	474685	337942	656112	6269571	7091696	5753756	0.0000	13.0	Increase
Q728Q8	DESVH Alcohol dehydrogenase, iron-containing	0	0	0	207971	83817	2471921	0.0011	NA	Increase
Q728R0	DESVH Hydroxylamine reductase	331166	131875	0	523068	1440042	498912	0.2926	5.3	Null
Q728R5	DESVH Threonine--tRNA ligase	0	0	0	795907	582599	522374	0.0000	NA	Increase
Q728R6	DESVH Translation initiation factor IF-3	1186923	722984	4419282	1260095	1020952	1683927	0.7495	1.6	Null
Q728R7	DESVH 50S ribosomal protein L35	3367765	2040796	2114508	835995	0	0	0.2855	9.0	Null
Q728S0	DESVH Phenylalanine--tRNA ligase beta subunit	0	0	0	2405550	2506036	225647	0.0000	NA	Increase
Q728S3	DESVH Transketolase	0	168602	0	0	61898	0	NA	2.7	Null
Q728T3	DESVH 30S ribosomal protein S9	21491630	9884073	17412121	9781237	7240684	8282253	0.2855	1.9	Null
Q728T4	DESVH 50S ribosomal protein L13	0	9820924	1749510	9601764	8964280	4960785	0.6835	2.0	Null
Q728T6	DESVH CBS domain protein	0	0	0	0	90629	0	NA	NA	Null
Q728T8	DESVH Pyruvate kinase	0	0	0	1585583	1793121	658377	0.0000	NA	Increase
Q728T9	DESVH Transcriptional regulator MraZ	0	0	0	111407	73144	0	NA	NA	Null
Q728U3	DESVH UDP-N-acetylmuramoyl-L-alanyl-D-glutamate--2,6-diaminopimelat	0	38296	0	109374	98646	58870	0.3476	7.0	Null
Q728V2	DESVH Cell division protein FtsA	0	48342	0	533579	366330	293034	0.0313	24.7	Increase
Q728V3	DESVH Cell division protein FtsZ	0	0	0	546733	565178	78677	0.0022	NA	Increase
Q728V6	DESVH Lipoprotein, putative	0	0	0	0	0	251650	0.2917	NA	Null
Q728V7	DESVH Thioesterase family protein	0	202059	446301	419931	431922	432627	0.6286	2.0	Null
Q728W3	DESVH Uncharacterized protein	0	0	0	0	24682	0	NA	NA	Null
Q728W8	DESVH Cytochrome c family protein	0	0	0	234520	283492	0	0.2316	NA	Null
Q728W9	DESVH Cytochrome c family protein	0	0	0	0	72276	0	NA	NA	Null
Q728X0	DESVH Formate dehydrogenase, alpha subunit, selenocysteine-containing	0	0	145719	346834	425636	218407	0.3148	6.8	Null
Q728X1	DESVH Formate dehydrogenase, beta subunit, putative	0	0	240059	0	0	0	NA	NA	Null
Q728X6	DESVH Glutamate synthase, small subunit	0	0	0	11382851	66729	257684	NA	NA	Null
Q728X8	DESVH Uncharacterized protein	0	0	0	0	0	41247	NA	NA	Null
Q728Y0	DESVH Glyco	0	0	0	0	20245	0	NA	NA	Null
Q728Y5	DESVH Ribonuclease R	0	479840	790397	0	5023382	7782023	0.2841	10.1	Null
Q728Z2	DESVH Uncharacterized protein	0	0	0	0	259658	0	0.3023	NA	Null
Q729A3	DESVH S-adenosylmethionine synthase	0	6975468	2430631	3810234	10848397	8995714	0.5660	2.5	Null
Q729A6	DESVH 3-methyl-2-oxobutanoate hydroxymethyltransferase	0	0	0	177055	0	227625	0.2524	NA	Null
Q729A8	DESVH Flagellin	197221822	105832643	54188019	91369970	116279221	378176462	0.6002	1.6	Null
Q729B0	DESVH Heat shock protein, Hsp20 family	2568194	18508935	5505534	9955774	8414374	11982759	0.7950	1.1	Null
Q729B1	DESVH Heat shock protein, Hsp20 family	15125311	18720843	36171080	43101874	61627977	21962702	0.2789	1.8	Null
Q729B3	DESVH Efflux transporter, RND family, MFP subunit	0	220507	447311	56737	699325	753543	0.6002	2.3	Null
Q729B7	DESVH Uncharacterized protein	0	0	0	0	129000	0	NA	NA	Null
Q729C3	DESVH Lipoprotein, putative	946990	2548238	0	1223346	3137560	2874070	0.6676	2.1	Null
Q729C4	DESVH Uncharacterized protein	0	1029484	0	5286054	11028209	3342531	0.1411	19.1	Null
Q729D1	DESVH Uncharacterized protein	0	1610574	2379064	1274685	1282415	949583	0.9962	1.1	Null
Q729D5	DESVH EVE domain-containing protein	271200	1088988	0	951740	324590	78491	0.9962	1.0	Null
Q729E1	DESVH Superoxide dismutase	0	0	201937	0	0	0	NA	NA	Null
Q729E6	DESVH Alcohol dehydrogenase, iron-containing	39857029	8648602	12865790	32282602	146184534	105629533	0.1073	4.6	Null
Q729E7	DESVH Heterodisulfide reductase, C subunit	247406	716385	925030	1067755	1108106	949583	0.3445	1.7	Null
Q729E8	DESVH Heterodisulfide reductase, B subunit	0	772791	783942	70119	0	0	0.2744	0.0	Null
Q729E9	DESVH Heterodisulfide reductase, A subunit	687186	0	641768	1126093	1093567	1538362	0.2804	2.8	Null
Q729F0	DESVH Hydrogenase, iron-sulfur cluster-binding subunit, putative	0	415809	250477	439597	589804	569015	0.5179	2.4	Null
Q729F1	DESVH Hydrogenase, putative	6776706	7844565	2148815	7123077	764539	2650875	0.6927	1.6	Null

Q729F2	DESVH Hydrogenase, putative	0	308758	11423997	1713375	1783285	2604801	NA	1.9	Null
Q729F3	DESVH Rubrerythrin domain-containing protein	1133349	2178115	2976241	16004036	11290216	15031644	0.0000	6.7	Increase
Q729F4	DESVH Uncharacterized protein	0	0	0	0	14442734	0	NA	NA	Null
Q729F7	DESVH Sigma-54 dependent transcriptional regulator/response regulator	0	0	0	94749	0	0	NA	NA	Null
Q729H2	DESVH Peptidase, M16 family, putative	0	1257572	0	805068	0	1462357	0.8463	1.8	Null
Q729H5	DESVH Lysine--tRNA ligase	0	0	0	0	112123	0	NA	NA	Null
Q729H8	DESVH Outer membrane protein assembly factor BamA	1019442	788352	690171	2795690	1612430	1818642	0.0727	2.5	Null
Q729I0	DESVH N-acetylmuramoyl-L-alanine amidase	0	136236	1136785	294966	33233	459699	0.9093	1.6	Null
Q729I1	DESVH Outer membrane protein OmpH, putative	2439138	3596214	1773207	0	0	560199	0.2583	13.9	Null
Q729I6	DESVH Aminotransferase, classes I and II	0	0	0	159952	162950	958894	0.0024	NA	Increase
Q729I7	DESVH Hydroxyethylthiazole kinase	0	1047986	0	0	133226	2563873	NA	2.6	Null
Q729J0	DESVH Oxidoreductase, FAD/NAD-binding family	0	0	0	38202	47597	24391	NA	NA	Null
Q729J6	DESVH Glycosyl transferase, group 2 family protein	0	1062157	0	960152	0	0	NA	1.1	Null
Q729K1	DESVH Glycogen phosphorylase	0	0	0	0	0	36950	NA	NA	Null
Q729K2	DESVH dUTP diphosphatase	0	0	0	0	0	499482	0.2196	NA	Null
Q729K3	DESVH Acetylornithine aminotransferase	707374	614760	0	178264	0	1181728	0.9679	1.0	Null
Q729K8	DESVH Amino acid ABC transporter, periplasmic amino acid-binding protei	0	0	0	71741	43809	88836	NA	NA	Null
Q729L4	DESVH Carboxyl-terminal protease	2756449	9693033	339136	9322129	5401786	5039830	0.6995	1.5	Null
Q729L5	DESVH Conserved domain protein	0	0	0	34181	65226	0	NA	NA	Null
Q729L7	DESVH Nucleoside diphosphate kinase	0	0	0	5348887	4026334	5739466	0.0000	NA	Increase
Q729L9	DESVH Smr family protein	0	275790	408773	369023	354941	194963	0.8463	1.3	Null
Q729M0	DESVH Iron-sulfur cluster carrier protein	0	0	0	0	3837763	0	NA	NA	Null
Q729M1	DESVH Hydrogenase accessory protein HypB	0	130223	0	184545	135749	0	0.8195	2.5	Null
Q729M4	DESVH Conserved domain protein	0	607974	0	98212	23336	203873	0.9019	1.9	Null
Q729M6	DESVH Copper-translocating P-type ATPase	0	0	0	42267	179958	0	NA	NA	Null
Q729M9	DESVH Uncharacterized protein	300937	0	0	0	0	0	0.2843	NA	Null
Q729N0	DESVH 3-octaprenyl-4-hydroxybenzoate carboxy-lyase family protein	0	0	0	333371	529397	172339	0.0013	NA	Increase
Q729N2	DESVH Rubrerythrin, putative	6067516	0	2805500	0	0	0	0.0041	NA	Decrease
Q729N3	DESVH Methyl-accepting chemotaxis protein, putative	272630	0	286062	5105151	1427307	938065	0.0679	13.4	Null
Q729N7	DESVH 6-phosphogluconolactonase	0	0	0	261439	0	0	NA	NA	Null
Q729P0	DESVH Metallo-beta-lactamase family protein	300227	382717	718516	885658	691010	0	0.9560	1.1	Null
Q729P1	DESVH Methyl-accepting chemotaxis protein, putative	681717	952147	1838303	20231663	2383375	1117721	0.0661	6.8	Null
Q729P7	DESVH Conserved domain protein	0	0	0	315663	94835	82742	0.0367	NA	Increase
Q729Q1	DESVH Glycine/betaine/L-proline ABC transporter, ATP binding protein	0	529065	300649	3876490	868940	901322	0.1786	6.8	Null
Q729Q3	DESVH Glycine/betaine/L-proline ABC transporter, periplasmic-binding pro	2105010	501421	110936	122337	408517	238473	0.3043	3.5	Null
Q729Q4	DESVH Methyl-accepting chemotaxis protein	201275	314776	0	143079	295651	407544	0.7715	1.6	Null
Q729Q9	DESVH Hydrogenase, CooU subunit, putative	0	0	0	0	933797	0	NA	NA	Null
Q729R0	DESVH Hydrogenase, CooX subunit, putative	0	87277	0	0	0	0	NA	NA	Null
Q729R7	DESVH Uncharacterized protein	0	296680	0	43147	0	0	0.6482	6.9	Null
Q729S7	DESVH Formate acetyltransferase, putative	0	0	0	0	0	4298444	NA	NA	Null
Q729U3	DESVH Holliday junction ATP-dependent DNA helicase RuvA	0	0	0	0	30471	0	NA	NA	Null
Q729U7	DESVH DNA-binding protein	0	0	182046	1018137	0	336454	0.5085	7.4	Null
Q729U8	DESVH AMP-binding protein	1614605	916106	1116488	2337461	1556329	1583197	0.4954	1.5	Null
Q729V1	DESVH Alkyl hydroperoxide reductase C	0	0	0	0	0	671038	0.1900	NA	Null
Q729V2	DESVH S1 RNA binding domain protein	0	0	0	1276410	151565	0	0.0832	NA	Null
Q729V6	DESVH Asparaginase family protein	0	0	0	154972	42892431	0	NA	NA	Null
Q729W3	DESVH Uncharacterized protein	0	0	0	122340	344825	6728328	NA	NA	Null
Q729W5	DESVH Uncharacterized protein	0	496413	1549839	0	0	210127	0.5479	9.7	Null
Q729W6	DESVH 50S ribosomal subunit assembly factor BipA	0	0	632672	3619588	1277372	881508	0.2813	9.1	Null
Q729X0	DESVH PilZ domain-containing protein	0	0	0	0	46747	0	NA	NA	Null
Q729X1	DESVH Acetyl-CoA carboxylase, biotin carboxylase, putative	0	0	0	751523	760473	821157	0.0000	NA	Increase
Q729X2	DESVH Acetyl-coenzyme A carboxylase carboxyl transferase subunits beta/	0	0	0	149192	556974	924379	0.0004	NA	Increase
Q729X5	DESVH Single-stranded DNA-binding protein	9698046	4483056	2558616	2458320	2215992	557768	0.1708	3.2	Null
Q729Y2	DESVH RNA-binding protein	1199981	3629308	6508761	11377485	14138992	12920426	0.0342	3.4	Increase
Q729Y8	DESVH Uncharacterized protein	0	0	0	0	161356	0	NA	NA	Null
Q729Z1	DESVH Uncharacterized protein	0	0	0	0	135762	218355	0.2619	NA	Null
Q729Z3	DESVH Tryptophanase	3752042	2697001	3903517	7093537	7435938	4080154	0.2083	1.8	Null
Q729Z4	DESVH Endoribonuclease, L-PSP family	4487244	4568723	0	621659	6252186	13673991	0.6812	2.3	Null
Q729Z6	DESVH Alcohol dehydrogenase, iron-containing	0	0	261430	12091198	7553682	5024615	0.0026	94.4	Increase
Q72A22	DESVH Adenine specific DNA methyltransferase, putative	690223	0	0	0	0	0	0.1968	NA	Null
Q72A40	DESVH Tail tape meausure protein, putative	9922664	0	0	0	89697	0	NA	110.6	Null
Q72A47	DESVH RNA polymerase-binding transcription factor DksA	299789	0	281421	0	0	0	0.2067	NA	Null
Q72A49	DESVH CBS/transporter associated domain protein	0	0	0	78520	0	98308	NA	NA	Null
Q72A54	DESVH Fructose-1,6-bisphosphate aldolase, class II	0	0	0	1961690	548346	2734164	0.0000	NA	Increase
Q72A55	DESVH 5'-nucleotidase SurE	0	0	0	0	0	353035	0.2583	NA	Null
Q72A59	DESVH LUD	3424108	0	4460921	2160342	1599073	829302	0.8093	1.7	Null
Q72A61	DESVH Uncharacterized protein	0	0	0	0	0	23281	NA	NA	Null
Q72A62	DESVH Uncharacterized protein	0	210327	230931	171834	213891	263995	0.7813	1.5	Null
Q72A64	DESVH UPF0324 membrane protein DVU	0	0	0	107549	1187737	87090	0.0050	NA	Increase
Q72A88	DESVH Iron-sulfur cluster carrier protein	18211031	11476249	17622129	22585407	13192917	15607328	0.8647	1.1	Null
Q72A89	DESVH MTH1175-like domain family protein	5360595	0	1857454	2618511	1469175	2272262	0.9468	1.1	Null
Q72A94	DESVH Iron-sulfur cluster-binding/ATPase domain protein	178979	1215548	1845234	1721652	2080377	2102956	0.0014	1.8	Increase
Q72A96	DESVH Uncharacterized protein	0	0	0	0	158912	331449	0.2154	NA	Null
Q72A97	DESVH Universal stress protein family	0	0	2780713	12937619	23060979	10545419	0.1981	16.7	Null
Q72AA3	DESVH Thiazole synthase	0	0	0	643519	649401	1627286	0.0000	NA	Increase
Q72AB3	DESVH Oligopeptide-binding protein, putative	0	0	0	0	99645	0	NA	NA	Null
Q72AB4	DESVH GTP pyrophosphokinase	0	0	0	0	160446	0	NA	NA	Null
Q72AB5	DESVH Flagellin	1173107	1512762	2735760	1551814	240459	1471338	0.7087	1.7	Null
Q72AC0	DESVH Uncharacterized protein	0	1237291	541275	4557653	2345895	709022	0.3280	4.3	Null
Q72AC1	DESVH Chemotaxis protein methyltransferase	0	0	0	179738	0	0	NA	NA	Null
Q72AC3	DESVH Chemotaxis protein CheW	0	0	0	173941	561324	333877	0.0013	NA	Increase
Q72AC4	DESVH Chemotaxis protein CheY	7189609	5485214	12449677	24946525	16862135	16907855	0.0627	2.3	Null
Q72AC5	DESVH Histidine kinase	251987	152198	219768	1503025	1031841	702691	0.0027	5.2	Increase
Q72AC7	DESVH Cell division coordinator CpoB	270054	1310990	14162166	731926	1107466	972298	0.2320	5.6	Null
Q72AD3	DESVH Oxidoreductase, 2-nitropropane dioxygenase family	0	936898	0	4098017	3560892	4573461	0.1968	13.1	Null
Q72AD6	DESVH ATP-dependent 6-phosphofructokinase	0	0	0	0	86146	210136	0.2786	NA	Null
Q72AD8	DESVH Glycosyl transferase, group 2 family protein	0	0	0	738263	55041	222366	0.0070	NA	Increase
Q72AE0	DESVH PSP1 C-terminal domain-containing protein	0	0	0	0	1039628	0	NA	NA	Null
Q72AE2	DESVH Methionine--tRNA ligase	184700	331637	0	705099	263186	120596	0.7001	2.1	Null
Q72AF2	DESVH VWFA domain-containing protein	12316987	9928198	15665914	6538967	6156838	6457162	0.1510	2.0	Null
Q72AF3	DESVH Fic family protein	0	0	0	380579	131411	377334	0.0028	NA	Increase
Q72AG0	DESVH Plasmid stabilization system family protein	0	0	0	0	0	54297	NA	NA	Null
Q72AH5	DESVH PglZ domain-containing protein	0	0	0	0	0	1057202	NA	NA	Null
Q72AK5	DESVH Excinuclease ABC, A subunit	0	0	0	2536232	1959490	815957	0.0000	NA	Increase
Q72AK6	DESVH DrsE domain-containing protein	0	445448	124090	0	0	166949	0.8324	3.4	Null
Q72AK8	DESVH Peptide methionine sulfoxide reductase MsrA	0	0	310217	0	0	0	0.3035	NA	Null
Q72AL1	DESVH UPF0234 protein DVU	438402	600597	63443	2127234	1581230	149577	0.3085	3.5	Null
Q72AL2	DESVH Uncharacterized protein	0	0	0	918766	0	412002	0.0721	NA	Null
Q72AL3	DESVH ATP-binding protein Uup	0	0	0	0	81921	190469	0.2886	NA	Null
Q72AL5	DESVH 10 kDa chaperonin	7031668	703860	1613499	11590619	9518509	13142512	0.2312	3.7	Null
Q72AL6	DESVH 60 kDa chaperonin	176758868	387388120	367920701	4514081152	4012548748	1764774460	0.0000	11.0	Increase
Q72AL7	DESVH Methyl-accepting chemotaxis protein	7302535	54367	1908982	1292827	357872	406215	0.2748	4.5	Null
Q72AL9	DESVH Rhodanese-like domain protein	0	0	0	1117254	2707579	205519	0.0000	NA	Increase
Q72AN0	DESVH Methyl-accepting chemotaxis protein	0	0	0	943598	383036	1115933	0.0000	NA	Increase
Q72AN9	DESVH Gamma-glutamyl phosphate reductase	0	267487	0	71344	105593	239709	0.8382	1.6	Null
Q72AP0	DESVH TPR	0	607846	792099	1107594	994410	865954	0.5945	2.1	Null
Q72AP1	DESVH Indolepyruvate oxidoreductase subunit IorA	0	242406	0	972416	10899786	475252	NA	50.9	Null
Q72AP3	DESVH Nif-specific regulatory protein	0	0	0	309207	283523	313576	0.0012	NA	Increase
Q72AP4	DESVH Uncharacterized protein	945756	745374	780412	2297649	2109668	2052571	0.0198	2.6	Increase
Q72AP5	DESVH Pyruvate ferredoxin oxidoreductase, gamma subunit, putative	0	0	0	69575	61474	455598	0.0313	NA	Increase
Q72AP6	DESVH Pyruvate ferredoxin oxidoreductase, beta subunit, putative	0	0	0	291044	104448	265266	0.0095	NA	Increase
Q72AP7	DESVH Pyruvate ferredoxin oxidoreductase, alpha subunit, putative	0	0	0	159552	143899	84910	0.0512	NA	Increase

Q72AP8	DESVH Pyruvate ferredoxin oxidoreductase, iron-sulfur binding subunit, pu	209557	0	0	615851	600306	0	0.6540	5.8	Null
Q72AQ2	DESVH Anaerobic glycerol-3-phosphate dehydrogenase, A subunit, putativ	0	0	0	398322	166303	0	0.2154	NA	Null
Q72AQ5	DESVH Phosphonate ABC transporter, periplasmic phosphonate-binding pr	2176655	15233393	8457596	71789909	67894706	48194121	0.0008	7.3	Increase
Q72AQ6	DESVH Phosphonates import ATP-binding protein PhnC	0	0	0	202018	127697	133623	0.0264	NA	Increase
Q72AR0	DESVH Adenylate kinase	4537151	12173866	20106630	90427912	131556531	61377461	0.0001	7.7	Increase
Q72AR1	DESVH Iron-sulfur cluster-binding protein	0	0	0	0	382589	0	0.2664	NA	Null
Q72AR2	DESVH Cell division coordinator CpoB	1832764	214349	62698	0	0	911506	0.8206	2.3	Null
Q72AR5	DESVH Isoleucine--tRNA ligase	0	0	0	0	0	86446	NA	NA	Null
Q72AS2	DESVH Hydrogenase expression/formation protein, putative	0	0	0	0	0	149153	NA	NA	Null
Q72AS3	DESVH Periplasmic [NiFeSe] hydrogenase, large subunit, selenocysteine-co	6865684	2939352	2223527	9805339	12795594	9483166	0.1842	2.7	Null
Q72AS4	DESVH Cytochrome-c3 hydrogenase	1084490	152232374	33644022	5921854	6791073	106771081	0.9455	1.6	Null
Q72AS7	DESVH (R)-citramalate synthase	0	0	0	231747	0	0	NA	NA	Null
Q72AS8	DESVH Aspartokinase	0	0	0	663016	650917	372007	0.0000	NA	Increase
Q72AT0	DESVH CBS domain protein	1528722	162693	0	644186	768107	658377	0.9635	1.2	Null
Q72AT2	DESVH Holo-[acyl-carrier-protein] synthase	0	0	0	694903	0	71521	0.1784	NA	Null
Q72AT3	DESVH UDP-glucose 6-dehydrogenase	0	270960	0	3037365	2074884	2050401	0.0458	26.4	Increase
Q72AT6	DESVH Chemotaxis protein CheW	0	0	0	6737619	4550706	5518896	0.0000	NA	Increase
Q72AT7	DESVH Transcription-repair-coupling factor	0	0	0	0	315684	0	0.2846	NA	Null
Q72AT9	DESVH Peptidyl-prolyl cis-trans isomerase domain protein	0	6651893	0	5060276	5196013	1604423	NA	1.8	Null
Q72AU0	DESVH DUF4115 domain-containing protein	0	0	0	404661	430704	5034620	NA	NA	Null
Q72AU2	DESVH Glycine--tRNA ligase alpha subunit	0	0	0	0	0	90684	NA	NA	Null
Q72AU3	DESVH Glycine--tRNA ligase beta subunit	0	0	0	669061	1360385	2496261	0.0000	NA	Increase
Q72AU7	DESVH Endopeptidase La	0	0	0	173300	323971	168771	0.0072	NA	Increase
Q72AV0	DESVH Porphobilinogen deaminase	0	0	613967	0	67614	433947	0.9893	1.2	Null
Q72AV1	DESVH Phosphoheptose isomerase	0	0	0	269654	0	0	NA	NA	Null
Q72AV3	DESVH Uncharacterized protein	0	0	0	169715	195330	116297	0.0214	NA	Increase
Q72AV4	DESVH Uncharacterized protein	0	0	0	34975	142635	0	NA	NA	Null
Q72AV5	DESVH Aspartyl/glutamyl-tRNA(Asn/Gln) amidotransferase subunit B	493005	448761	419164	1293004	2849937	1931564	0.0035	4.5	Increase
Q72AV9	DESVH PhoH family protein	0	0	0	314294	168652	80782	0.0205	NA	Increase
Q72AW0	DESVH Uncharacterized protein	586027	3166417	2708650	4932578	3598819	2219106	0.4260	1.7	Null
Q72AW4	DESVH DnaJ protein, putative	468152	1489230	2962608	2756299	2247339	2833689	0.4549	1.6	Null
Q72AW6	DESVH Chaperone protein ClpB	769093	398223	115393	3472394	3155676	19783928	0.0019	20.6	Increase
Q72AW7	DESVH Peptidyl-prolyl cis-trans isomerase	2185070	10370471	766072	38618425	46302435	38169012	0.0062	9.2	Increase
Q72AX1	DESVH Methyl-accepting chemotaxis protein	483467	168687	550206	1763674	1428191	1689546	0.0275	4.1	Increase
Q72AX2	DESVH 4-hydroxy-tetrahydrodipicolinate synthase	0	0	0	820640	148226	38712	0.0134	NA	Increase
Q72AX7	DESVH Flagellar synthesis regulator FleN, putative	0	0	0	0	0	63429	NA	NA	Null
Q72AY3	DESVH Methyl-accepting chemotaxis protein	0	174164	0	600234	214923	426792	0.2926	7.1	Null
Q72AY6	DESVH Uncharacterized protein	731546	1007766	1090581	2930118	1842124	1486431	0.0414	2.2	Increase
Q72AY9	DESVH Peptidase, M23/M37 family	0	0	0	123602	1500301	1187471	0.0001	NA	Increase
Q72AZ0	DESVH CBS domain protein	0	0	0	0	337817	531151	0.1124	NA	Null
Q72AZ3	DESVH Iron-sulfur cluster carrier protein	0	159905	0	1707987	1476673	4379308	0.0101	47.3	Increase
Q72AZ8	DESVH Lipoprotein, putative	0	0	0	0	37277	0	NA	NA	Null
Q72B01	DESVH Thioredoxin	4867369	3241520	3662253	13562108	11716046	12581217	0.0048	3.2	Increase
Q72B02	DESVH Thioredoxin-disulfide reductase	0	0	0	588605	810731	587049	0.0000	NA	Increase
Q72B06	DESVH Pyruvate carboxylase	617698	231359	226137	2601707	1374822	324628	0.1944	4.0	Null
Q72B07	DESVH Phosphoenolpyruvate synthase	9377099	1795250	2046795	16775579	23009713	22315921	0.0886	4.7	Null
Q72B12	DESVH Acetylornithine deacetylase/succinyl-diaminopimelate desuccinyla	0	0	0	461586	274034	136544	0.0029	NA	Increase
Q72B13	DESVH Uncharacterized protein	2738341	3386619	0	0	483426	1095185	0.6589	3.9	Null
Q72B16	DESVH Glutamate synthase (NADPH)	0	0	0	179596	104912	729207	0.0049	NA	Increase
Q72B17	DESVH Glutamate synthase, amidotransferase subunit, putative	0	0	0	0	0	58305	NA	NA	Null
Q72B18	DESVH GXGXG domain-containing protein	0	524760	762959	827436	537158	784988	0.7134	1.7	Null
Q72B19	DESVH Sec translocon accessory complex subunit YajC	0	0	0	0	10334025	7216829	0.0012	NA	Increase
Q72B20	DESVH Protein translocase subunit SecD	0	0	0	2988060	5131144	197414	0.0000	NA	Increase
Q72B22	DESVH Uncharacterized protein	0	0	0	343755	3467501	0	0.0193	NA	Increase
Q72B26	DESVH Cytochrome c oxidase subunit 2	0	0	0	0	42501	0	NA	NA	Null
Q72B29	DESVH L-aspartate oxidase	0	0	0	0	589758	0	0.2199	NA	Null
Q72B31	DESVH Quinolinate phosphoribosyltransferase [decarboxylating]	0	0	0	173548	126274	0	0.2947	NA	Null
Q72B43	DESVH DNA-binding protein HU	4767334	10612141	15598582	11753586	7408127	8229717	0.9758	1.1	Null
Q72B47	DESVH GatB/Yqey family protein	0	196684	931170	1175359	1177871	1185699	0.4187	3.1	Null
Q72B48	DESVH Endonuclease MutS2	0	0	0	0	175256	0	NA	NA	Null
Q72B49	DESVH DNA primase	0	519433	0	0	0	0	0.2559	NA	Null
Q72B50	DESVH RNA polymerase sigma factor RpoD	0	0	0	208637	166693	118529	0.0203	NA	Increase
Q72B56	DESVH Iron-sulfur cluster-binding protein	0	0	26035043	0	52616	319802	NA	69.9	Null
Q72B57	DESVH LUD	0	0	0	139840	93584	0	NA	NA	Null
Q72B68	DESVH GTP-binding protein	0	0	0	0	44973	92280	NA	NA	Null
Q72B71	DESVH ThiH protein, putative	0	0	0	359697	0	180622	0.2140	NA	Null
Q72B85	DESVH Uncharacterized protein	0	13678851	0	0	223160	0	NA	61.3	Null
Q72B89	DESVH ATPase, histidine kinase-, DNA gyrase B-, and HSP90-like domain pr	0	0	0	0	210259	0	NA	NA	Null
Q72B91	DESVH DNA-binding protein	0	0	0	0	0	90728	NA	NA	Null
Q72BD1	DESVH Type I restriction-modification enzyme, S subunit	0	0	0	0	0	2633509	NA	NA	Null
Q72BE6	DESVH Transcriptional regulator, TetR family	0	0	0	0	0	854624	NA	NA	Null
Q72BE8	DESVH 1-acyl-sn-glycerol-3-phosphate acyltransferase, putative	0	0	0	258934	0	179577	0.2494	NA	Null
Q72BF1	DESVH Ribosomal RNA small subunit methyltransferase E	0	0	0	109787	49860	0	NA	NA	Null
Q72BF2	DESVH Glycine cleavage system T protein	0	0	0	937551	914268	1561337	0.0000	NA	Increase
Q72BF4	DESVH GAF domain protein	0	228879	28911	291366	321224	206218	0.4402	3.2	Null
Q72BF5	DESVH Cell shape-determining protein MreB	0	0	0	118469267	93103	94580	NA	NA	Null
Q72BG0	DESVH Protein-export membrane protein SecG	0	0	0	0	137699	517131	0.1606	NA	Null
Q72BG3	DESVH Acyltransferase, putative	0	0	0	0	0	4766905	NA	NA	Null
Q72BG8	DESVH Outer-membrane lipoprotein carrier protein	381307	629800	958503	717353	6772066	184861	0.2682	3.9	Null
Q72BH1	DESVH 3-dehydroquinate dehydratase	0	0	0	1945136	1058304	0	0.0211	NA	Increase
Q72BH8	DESVH Transaldolase, putative	0	0	0	266127	106877	0	0.2722	NA	Null
Q72BI1	DESVH LL-diaminopimelate aminotransferase	0	0	0	720043	1014190	259353	0.0000	NA	Increase
Q72BI5	DESVH LapA	1566550	0	517772	2681804	2908269	0	0.7805	2.7	Null
Q72BI6	DESVH TPR	0	0	0	135425	0	0	NA	NA	Null
Q72BJ9	DESVH Pyrophosphate phospho-hydrolase	59727935	1953235	595453	118123877	253024924	281130268	0.1296	10.5	Null
Q72BK2	DESVH PTS system, IIB component	0	0	0	943598	0	0	NA	NA	Null
Q72BK6	DESVH Ribosome hibernation promoting factor	1162252	801580	0	3377087	2910074	2334038	0.3517	4.4	Null
Q72BK9	DESVH Uncharacterized protein	0	79538	0	63963	33064	0	NA	1.2	Null
Q72BL0	DESVH 3-deoxy-D-manno-octulosonate 8-phosphate phosphatase KdsC	0	0	0	0	0	286681	0.2769	NA	Null
Q72BL2	DESVH CTP synthase	0	0	0	845743	1073694	802347	0.0000	NA	Increase
Q72BL3	DESVH Phosphoribosylformylglycinamidine synthase I	0	0	0	630163	538652	1335746	0.0000	NA	Increase
Q72BL6	DESVH 2,3-bisphosphoglycerate-independent phosphoglycerate mutase	0	0	0	224767	324092	212182	0.0034	NA	Increase
Q72BL7	DESVH Ribosomal silencing factor RsfS	0	0	0	110977	606250	234194	0.0041	NA	Increase
Q72BM0	DESVH Phenylacetate-coenzyme A ligase	296153	0	8955157	1208467	902671	841963	NA	3.1	Null
Q72BM3	DESVH ACT domain protein	4375047	819172	1368889	76345820	38587983	38901649	0.0000	23.4	Increase
Q72BM6	DESVH 4-hydroxy-tetrahydrodipicolinate reductase	0	0	0	230903	527533	0	0.1530	NA	Null
Q72BN0	DESVH UvrABC system protein B	0	0	0	120120	146032	1323085	0.0023	NA	Increase
Q72BN1	DESVH Uncharacterized protein	0	0	0	260992	239829	0	0.2359	NA	Null
Q72BN3	DESVH ATP-dependent Clp protease, ATP-binding subunit ClpA	0	0	713686	415762	488555	400878	NA	1.8	Null
Q72BN7	DESVH DUF190 domain-containing protein	0	0	0	0	240308	206111	0.2359	NA	Null
Q72BP1	DESVH Chemotaxis protein CheY	694266	56302	946394	2384245	1400696	1823199	0.2517	3.3	Null
Q72BP2	DESVH Arginine N-succinyltransferase, beta subunit, putative	0	0	0	0	0	102659	NA	NA	Null
Q72BP5	DESVH Znf/thioredoxin	0	0	0	0	0	34602	NA	NA	Null
Q72BP6	DESVH Hypoxanthine phosphoribosyltransferase	0	0	0	580666	197900	98681	0.0058	NA	Increase
Q72BP8	DESVH Thioredoxin family protein	0	0	0	96164	107230	119184	0.0678	NA	Null
Q72BP9	DESVH 5-methyltetrahydrofolate--homocysteine methyltransferase	0	0	0	1036524	307956	375386	0.0001	NA	Increase
Q72BQ0	DESVH RNA polymerase sigma factor	0	116934	0	0	0	16052	NA	7.3	Null
Q72BQ1	DESVH TPR domain protein	0	0	0	70139	74905	37365	NA	NA	Null
Q72BQ2	DESVH Uncharacterized protein	0	0	0	187361	310167	0	0.2353	NA	Null
Q72BQ3	DESVH Uncharacterized protein	0	275255	0	361235	0	129069	0.8722	1.8	Null
Q72BQ4	DESVH Ribose 5-phosphate isomerase, putative	0	0	1128484	1180188	307129	443699	0.8382	1.7	Null
Q72BQ5	DESVH Cysteine--tRNA ligase	0	0	0	684506	642178	1328761	0.0000	NA	Increase

Q72BQ9	DESVH Ribose-phosphate pyrophosphokinase	0	0	0	2183847	1333976	892608	0.0000	NA	Increase
Q72BR0	DESVH 50S ribosomal protein L25	314921	0	1631872	9710838	9050215	7647402	0.0656	13.6	Null
Q72BR3	DESVH Transcription termination factor Rho	854287	280005	389697	18331250	16215164	2740421	0.0000	24.5	Increase
Q72BR4	DESVH Pyruvate ferredoxin oxidoreductase, beta subunit	269073	0	0	128943	238037	106029	0.8820	1.8	Null
Q72BT1	DESVH AMP-binding protein	0	0	0	0	0	24073	NA	NA	Null
Q72BT6	DESVH Outer membrane transport protein, OmpP1/FadL/TodX family	0	0	0	0	0	5077102	NA	NA	Null
Q72BT9	DESVH Hemolysin-type calcium-binding repeat/calx-beta domain protein	0	82703	0	356778	156521	373100	0.1820	10.7	Null
Q72BU0	DESVH Mechanosensitive ion channel family protein	0	0	0	691653	75547	822972	0.0011	NA	Increase
Q72BU5	DESVH Fructose-1,6-bisphosphatase	4084166	5484842	4638260	34698861	24334483	13118408	0.0000	5.1	Increase
Q72BU7	DESVH Lipoprotein, putative	4416050	19024981	1042352	23153773	32166430	4339937	0.4319	2.4	Null
Q72BV4	DESVH Metallo-beta-lactamase family protein	840463	0	155719	0	60875	186819	0.6266	4.0	Null
Q72BW1	DESVH Uncharacterized protein	0	0	0	79114	136880	0	NA	NA	Null
Q72BX5	DESVH Uncharacterized protein	0	0	0	150828	0	110637	0.3007	NA	Null
Q72BZ4	DESVH Tail tape measure protein, putative	0	0	0	0	24389	0	NA	NA	Null
Q72C12	DESVH Endopeptidase La	0	130645	0	0	1400696	0	NA	10.7	Null
Q72C14	DESVH Peptidylprolyl isomerase	997882	1820170	1818763	2427360	1462949	1367399	0.7349	1.1	Null
Q72C15	DESVH Ribosomal protein S1, putative	6022992	12718875	3637396	144243873	143221656	136054053	0.0000	18.9	Increase
Q72C16	DESVH Periplasmic serine endoprotease DegP-like	17207130	18302322	59808048	125677063	59690441	51981835	0.1696	2.5	Null
Q72C17	DESVH ATP-dependent protease ATPase subunit HslU	0	1974608	2081558	1340902	337060	173715	0.7480	2.2	Null
Q72C18	DESVH Acetylglutamate kinase	0	122568	0	1928629	2672152	1256658	0.0052	47.8	Increase
Q72C23	DESVH Glutamyl-tRNA reductase	0	0	0	267887	184650	156937	0.0091	NA	Increase
Q72C26	DESVH Chemotaxis protein CheZ	451600	1066122	2183099	1423674	0	2285128	0.9463	1.0	Null
Q72C27	DESVH Thioredoxin reductase, putative	0	663388	331547	6562866	6471133	4323767	0.0141	17.4	Increase
Q72C29	DESVH zf-RING	150060964	15555852	31179860	17673129	22438475	15045583	0.1984	3.6	Null
Q72C30	DESVH Bifunctional enzyme IspD/IspF	0	0	0	0	69672	201269	0.2880	NA	Null
Q72C36	DESVH Uncharacterized protein	0	0	0	110458	0	115336	NA	NA	Null
Q72C39	DESVH Flagellar hook-length control domain protein	0	61455	50158	738141	52948	695679	0.0583	13.3	Null
Q72C40	DESVH Basal-body rod modification protein FlgD	0	0	0	297848	0	0	0.2989	NA	Null
Q72C41	DESVH Flagellar hook protein FlgE	128229	17539817	732004	1934008	245804	710802	NA	6.4	Null
Q72C43	DESVH Flagellin	8509148	8836976	609301	1233299	113020	2323312	0.2643	4.9	Null
Q72C46	DESVH Cobyrinic acid a,c-diamide synthase family protein	0	0	0	1729456	1255010	1196969	0.0000	NA	Increase
Q72C50	DESVH Uncharacterized protein	1761455	1268603	1981835	14970010	10945805	14056236	0.0000	8.0	Increase
Q72C52	DESVH Radical SAM domain protein	0	0	0	0	57147	0	NA	NA	Null
Q72C54	DESVH Peptidase, M16 family	0	307305	150818	2294770	4148860	2135850	0.0064	18.7	Increase
Q72C56	DESVH Phosphoglucomutase, alpha-D-glucose phosphate-specific	0	0	0	0	172658	456322	0.1676	NA	Null
Q72C60	DESVH Glycine dehydrogenase (aminomethyl-transferring)	0	2846084	0	438168	3918955	4139615	0.6405	3.0	Null
Q72C62	DESVH OmpA family protein	145768588	377034177	189884926	818692466	425167193	485717809	0.0589	2.4	Null
Q72C63	DESVH Uncharacterized protein	0	0	0	352810	156590	63496	0.0252	NA	Increase
Q72C66	DESVH Histidine kinase	0	0	0	95301	137008	184757	0.0355	NA	Increase
Q72C69	DESVH AsmA family protein	0	0	0	351974	0	131315	0.2427	NA	Null
Q72C70	DESVH Sensory box/GGDEF domain/EAL domain protein	0	0	0	0	0	458238	0.2300	NA	Null
Q72C73	DESVH Thiamine biosynthesis protein ThiC	0	0	1905794	4979537	3880984	3956595	0.3666	6.7	Null
Q72C76	DESVH Uncharacterized protein	0	0	0	342864	0	36272	0.2721	NA	Null
Q72C78	DESVH Phosphoribosylformylglycinamidine cyclo-ligase	0	0	0	279667	1220623	133234	0.0011	NA	Increase
Q72C87	DESVH Bacterioferritin	2288710	7435117	11950496	9580033	8080461	15618596	0.4650	1.5	Null
Q72C94	DESVH Uncharacterized protein	0	0	0	83580	110974	0	NA	NA	Null
Q72CA2	DESVH HesB family selenoprotein	1942413	2113467	1695512	5348595	5359997	5273349	0.0019	2.8	Increase
Q72CA6	DESVH Ketol-acid reductoisomerase (NADP(+))	2407277	2489511	3784283	6444786	8767195	5767508	0.0155	2.4	Increase
Q72CA7	DESVH Acetolactate synthase	0	100121	0	929596	680101	356980	0.0634	19.6	Null
Q72CB0	DESVH Cell division initiation protein DivIVA	678632	669170	870033	1782695	297066	851949	0.7581	1.3	Null
Q72CB3	DESVH Uncharacterized protein	0	0	0	91091	0	0	NA	NA	Null
Q72CB5	DESVH Rhodanese-like domain protein	0	0	0	390847	261719	288637	0.0010	NA	Increase
Q72CB6	DESVH Sec-independent protein translocase protein TatA	0	0	0	0	0	42967	NA	NA	Null
Q72CB7	DESVH Lipoprotein, putative	0	0	0	0	39620	0	NA	NA	Null
Q72CB9	DESVH dTDP-glucose 4,6-dehydratase	0	0	0	214929	200001	137871	0.0136	NA	Increase
Q72CC3	DESVH UDP-glucose 4-epimerase	0	0	0	0	84076	356099	0.2370	NA	Null
Q72CD0	DESVH DNA polymerase III subunit alpha	0	0	0	0	0	29334	NA	NA	Null
Q72CD3	DESVH 1-deoxy-D-xylulose-5-phosphate synthase	0	0	0	279430	120764	0	0.2673	NA	Null
Q72CD8	DESVH Proline--tRNA ligase	1359218	746783	1282399	3838041	8203759	7983952	0.0014	5.9	Increase
Q72CD9	DESVH 4-hydroxy-3-methylbut-2-en-1-yl diphosphate synthase (flavodoxin	0	124824	0	763928	482009	85085	0.2271	10.7	Null
Q72CE0	DESVH Cation ABC transporter, periplasmc-binding protein	5831119	1248131	1335102	4260502	1022379	765997	0.7465	1.4	Null
Q72CE6	DESVH Lon protease	0	2658473	108404533	1464740	1581568	4337290	NA	15.0	Null
Q72CE7	DESVH ATP-dependent Clp protease ATP-binding subunit ClpX	0	0	0	1002716	1422966	601073	0.0000	NA	Increase
Q72CE8	DESVH ATP-dependent Clp protease proteolytic subunit	626420	0	207926	4069895	4154523	1559599	0.1072	11.7	Null
Q72CE9	DESVH Trigger factor	2486610	8709257	9649876	33770687	19838229	30722404	0.0070	4.0	Increase
Q72CF3	DESVH 50S ribosomal protein L17	5409187	2936713	7482680	3366886	1182810	2867740	0.3289	2.1	Null
Q72CF4	DESVH DNA-directed RNA polymerase subunit alpha	0	130378	0	1402710	1088231	425036	0.0553	22.4	Null
Q72CF5	DESVH 30S ribosomal protein S4	2611637	2325846	1299844	1185937	834192	82218	0.2688	3.0	Null
Q72CF6	DESVH 30S ribosomal protein S11	970649	0	0	353770	0	0	NA	2.7	Null
Q72CF7	DESVH 30S ribosomal protein S13	3805029	4218399	5630509	2442724	212799	590499	0.1202	4.2	Null
Q72CG1	DESVH 50S ribosomal protein L15	423426	7126240	3503291	1738206	2560284	661044	0.5482	2.2	Null
Q72CG2	DESVH 50S ribosomal protein L30	0	0	632616	0	0	0	0.2334	NA	Null
Q72CG3	DESVH 30S ribosomal protein S5	3843056	5864077	5988625	32020137	15565007	5644568	0.0553	3.4	Null
Q72CG4	DESVH 50S ribosomal protein L18	6422111	17414855	28237433	462310	14180804	7913189	0.6052	2.3	Null
Q72CG5	DESVH 50S ribosomal protein L6	2731009	3598932	6131524	7651366	574576	899656	0.8285	1.4	Null
Q72CG6	DESVH 30S ribosomal protein S8	1407167	8273499	0	9200804	220551	2134570	0.9359	1.2	Null
Q72CG8	DESVH 50S ribosomal protein L5	606968	136289	0	0	2686364	794230	0.5811	4.7	Null
Q72CH0	DESVH 50S ribosomal protein L14	5240128	40292586	49870574	25609574	16781659	6325802	0.5660	2.0	Null
Q72CH1	DESVH 30S ribosomal protein S17	203604	678249	10912532	1629872	1895283	633055	NA	2.8	Null
Q72CH2	DESVH 50S ribosomal protein L29	2026909	2316580	3658372	1092587	1056993	1785216	0.3043	2.0	Null
Q72CH3	DESVH 50S ribosomal protein L16	2604926	3430744	4825461	2839070	2126002	0	0.6831	2.2	Null
Q72CH4	DESVH 30S ribosomal protein S3	0	443048	438596	360355	1556383	818745	0.4102	3.1	Null
Q72CH5	DESVH 50S ribosomal protein L22	9267378	2829644	5788670	2808974	2630368	945360	0.1752	2.8	Null
Q72CH6	DESVH 30S ribosomal protein S19	0	888176	198274	1707397	1625071	1797877	0.2622	4.7	Null
Q72CH7	DESVH 50S ribosomal protein L2	4824113	11768755	9782382	3654476	4111191	5196546	0.2725	2.0	Null
Q72CH8	DESVH 50S ribosomal protein L23	2397339	7435117	4279490	3658511	2409364	4918838	0.8907	1.3	Null
Q72CH9	DESVH 50S ribosomal protein L4	9669119	4611097	5950999	7548782	7137133	8707239	0.8285	1.2	Null
Q72CI0	DESVH 50S ribosomal protein L3	1827903	1320973	1495917	2880943	1102556	385654	0.9438	1.1	Null
Q72CI1	DESVH 30S ribosomal protein S10	209414	629267	161818	0	528844	4887899	0.3084	5.4	Null
Q72CI3	DESVH Elongation factor G	3267137	2425112	7318951	211022626	87190522	34275557	0.0000	25.6	Increase
Q72CI4	DESVH 30S ribosomal protein S7	795416	1324231	2319523	8355739	8831701	13186836	0.0001	6.8	Increase
Q72CI5	DESVH 30S ribosomal protein S12	117489174	31846070	40623645	5348114	8155165	12667433	0.0119	7.3	Decrease
Q72CI8	DESVH Sulfate adenylyltransferase	10728282	7413178	24320040	217506295	123289317	120928735	0.0000	10.9	Increase
Q72CJ2	DESVH RsbRD	0	0	0	0	47976	0	NA	NA	Null
Q72CJ3	DESVH Nitrate reductase, gamma subunit, putative	0	138460	0	558890	0	0	0.7570	4.0	Null
Q72CJ4	DESVH Reductase, iron-sulfur binding subunit, putative	509652	434379	0	1316071	1251289	997530	0.3846	3.8	Null
Q72CJ6	DESVH Reductase, iron-sulfur binding subunit, putative	1137403	5920200	6239433	6381701	4861972	4564328	0.7432	1.2	Null
Q72CJ7	DESVH Reductase, transmembrane subunit, putative	701172	0	0	0	0	0	NA	NA	Null
Q72CJ8	DESVH Response regulator	0	0	0	0	114495	0	NA	NA	Null
Q72CK0	DESVH UTP--glucose-1-phosphate uridylyltransferase	0	4070561	0	11519977	5046471	1175554	0.5640	4.4	Null
Q72CK1	DESVH Phosphoglucosamine mutase	0	0	0	0	0	652092	0.1933	NA	Null
Q72CK2	DESVH Uncharacterized protein	0	0	0	152726	195542	0	0.2787	NA	Null
Q72CK5	DESVH ATP-dependent zinc metalloprotease FtsH	0	0	73337	262534	1905883	3541916	0.0031	77.9	Increase
Q72CL1	DESVH General secretion pathway protein E, putative	0	0	0	0	99581	0	NA	NA	Null
Q72CL7	DESVH Uncharacterized protein	0	0	0	52448	0	21897	NA	NA	Null
Q72CM3	DESVH Outer membrane protein P1, putative	0	0	0	49540	429091	0	0.2433	NA	Null
Q72CM5	DESVH Glutamine synthetase, type III	0	0	0	639515	858182	710367	0.0000	NA	Increase
Q72CM6	DESVH RNA-binding protein	0	125121	0	1159532	1624007	1398864	0.0181	33.4	Increase
Q72CM8	DESVH Sua5/YciO/YrdC/YwlC family protein	0	0	0	0	666109	0	0.2072	NA	Null
Q72CN4	DESVH Malonyl CoA-acyl carrier protein transacylase	0	0	0	213714	212728	0	0.2616	NA	Null
Q72CN5	DESVH Arginine--tRNA ligase	0	0	0	0	13903344	0	NA	NA	Null
Q72CN6	DESVH SPOR domain-containing protein	0	0	0	0	0	2146057	NA	NA	Null

Q72CP0	DESVH Uncharacterized protein	0	155474	0	273717	124115	0	0.8217	2.6	Null
Q72CP2	DESVH Cupin	314578	277449	0	0	541896	129519	0.9679	1.1	Null
Q72CP5	DESVH Amino acid ABC transporter, periplasmic amino acid-binding protei	0	0	0	339419	554575	0	0.1251	NA	Null
Q72CP7	DESVH Amino acid ABC transporter, ATP-binding protein	0	0	0	401964	327393	344258	0.0004	NA	Increase
Q72CQ0	DESVH Bifunctional uridylyltransferase/uridylyl-removing enzyme	0	0	95963	0	0	0	NA	NA	Null
Q72CQ1	DESVH Nitrogen regulatory protein P-II	799573	0	1807997	2375549	2301838	1063533	0.6626	2.2	Null
Q72CQ5	DESVH Thiol peroxidase	2647799	3056311	954797	6759353	10730209	6156448	0.0414	3.6	Increase
Q72CR0	DESVH YcaO domain-containing protein	222164	0	0	0	0	38256	0.7063	5.8	Null
Q72CR1	DESVH Uncharacterized protein	0	0	0	245081	207556	454170	0.0019	NA	Increase
Q72CS2	DESVH 50S ribosomal protein L28	156051	0	0	0	0	0	NA	NA	Null
Q72CS3	DESVH Uncharacterized protein	0	0	0	2019217	2338623	1253449	0.0000	NA	Increase
Q72CS6	DESVH 3-oxoacyl-[acyl-carrier-protein] synthase 3	0	0	0	769841	641794	1357245	0.0000	NA	Increase
Q72CS7	DESVH 3-oxoacyl-[acyl-carrier-protein] reductase	0	0	0	597757	740813	168889	0.0003	NA	Increase
Q72CS9	DESVH 3-oxoacyl-[acyl-carrier-protein] synthase 2	0	0	1094040	3401918	7088325	5235366	0.1887	14.4	Null
Q72CT0	DESVH Serine hydroxymethyltransferase	0	0	0	2333942	0	2132653	0.0097	NA	Increase
Q72CT4	DESVH Riboflavin biosynthesis protein RibBA	216261	0	394624	543083	486371	5882888	NA	11.3	Null
Q72CT6	DESVH Transcription antitermination protein NusB	0	102979	0	274277	54936	199890	0.4346	5.1	Null
Q72CT7	DESVH Leucine--tRNA ligase	0	0	0	0	91368	0	NA	NA	Null
Q72CT8	DESVH Lipoprotein, putative	0	0	0	88848	26982	0	NA	NA	Null
Q72CU2	DESVH Lon protease	257679	0	0	1945136	0	0	NA	7.5	Null
Q72CU7	DESVH MazG family protein	0	0	0	247997	472104	167468	0.0024	NA	Increase
Q72CV4	DESVH Aldehyde:ferredoxin oxidoreductase, tungsten-containing	2902132	4819519	2887093	24137284	11132573	8046128	0.0039	4.1	Increase
Q72CV6	DESVH Uncharacterized protein	0	0	0	365834	416355	3956419	NA	NA	Null
Q72CV7	DESVH Uncharacterized protein	0	0	295336	762690	1176585	3139954	0.1193	17.2	Null
Q72CV9	DESVH Uncharacterized protein	0	136680	0	176166	0	0	0.9573	1.3	Null
Q72CX0	DESVH Major facilitator superfamily protein	0	0	0	686899	0	0	0.2129	NA	Null
Q72CY7	DESVH Transcriptional regulator, Cro/CI family	0	51116	105825	271010	0	106790	0.7422	2.4	Null
Q72CY8	DESVH Uncharacterized protein	0	0	0	0	97184	229291	0.2681	NA	Null
Q72CZ0	DESVH Uncharacterized protein	0	0	0	0	3616909	0	NA	NA	Null
Q72CZ1	DESVH Bacteriophage transposase A protein, putative	0	0	0	82127	0	0	NA	NA	Null
Q72CZ2	DESVH Bacteriophage DNA transposition B protein, putative	0	0	0	2964987	1923772	1335854	0.0000	NA	Increase
Q72CZ3	DESVH Uncharacterized protein	0	0	0	105789	0	0	NA	NA	Null
Q72CZ5	DESVH Host-nuclease inhibitor protein Gam, putative	0	0	0	4198864	44872765	1639613	0.0000	NA	Increase
Q72D08	DESVH Conserved domain protein	0	0	0	0	57980	0	NA	NA	Null
Q72D10	DESVH Uncharacterized protein	0	0	0	0	52142	0	NA	NA	Null
Q72D13	DESVH Uncharacterized protein	0	0	0	672121	901364	1329416	0.0000	NA	Increase
Q72D14	DESVH Uncharacterized protein	0	0	0	7259081	5279068	6229263	0.0000	NA	Increase
Q72D15	DESVH Uncharacterized protein	0	0	0	400698	341995	292859	0.0005	NA	Increase
Q72D18	DESVH Uncharacterized protein	0	0	0	0	132960	0	NA	NA	Null
Q72D20	DESVH Uncharacterized protein	0	0	0	993474	627217	1376337	0.0000	NA	Increase
Q72D21	DESVH Uncharacterized protein	0	0	0	724590	691010	854624	0.0000	NA	Increase
Q72D22	DESVH ATPase domain protein	0	0	0	0	292998	0	0.2925	NA	Null
Q72D23	DESVH Uncharacterized protein	0	0	0	1873306	1294866	988895	0.0000	NA	Increase
Q72D24	DESVH Tail tape measure protein	0	0	318106	0	0	0	0.3007	NA	Null
Q72D29	DESVH Baseplate assembly protein, putative	0	0	0	0	0	671038	0.1900	NA	Null
Q72D31	DESVH Tail fiber protein, putative	0	0	0	0	110811	0	NA	NA	Null
Q72D33	DESVH Methyltransferase	0	0	0	0	197537	0	NA	NA	Null
Q72D35	DESVH Ornithine carbamoyltransferase	0	0	0	113129	0	0	NA	NA	Null
Q72D36	DESVH Argininosuccinate lyase	0	0	0	0	784390	0	NA	NA	Null
Q72D45	DESVH Phosphate-specific transport system accessory protein PhoU	0	0	0	0	156819	0	NA	NA	Null
Q72D46	DESVH Phosphate import ATP-binding protein PstB	0	0	0	148547	89920	442980	0.0131	NA	Increase
Q72D52	DESVH R3H domain protein	1388728	2849451	2640396	3455833	1730149	6644375	0.3730	1.7	Null
Q72D53	DESVH Membrane protein insertase YidC	0	0	0	268782	315624	0	0.2016	NA	Null
Q72D56	DESVH Conserved domain protein	145630	0	0	403008	1239643	114903	0.2864	12.1	Null
Q72D58	DESVH Uncharacterized protein	0	0	0	0	318937	0	0.2846	NA	Null
Q72D62	DESVH Membrane protein, Bmp family	0	0	0	647475	277670	0	0.1239	NA	Null
Q72D64	DESVH Peptidylprolyl isomerase	1208384	1810130	381144	6142134	3848251580	896404159	0.0000	1397.4	Increase
Q72D65	DESVH Aconitate hydratase A	0	63316	0	1114820	1085955	954850	0.0013	49.8	Increase
Q72D66	DESVH Transcriptional regulatory protein	0	0	0	0	90465	0	NA	NA	Null
Q72D68	DESVH Glycosyl transferase, group 1 family protein	0	0	0	64134	0	0	NA	NA	Null
Q72D69	DESVH Glycosyl transferase, group 1 family protein	0	0	0	0	52577	0	NA	NA	Null
Q72D85	DESVH Inosine-5'-monophosphate dehydrogenase	393931	723087	537309	2411649	3493209	4787474	0.0001	6.5	Increase
Q72D86	DESVH GMP synthase [glutamine-hydrolyzing]	0	0	0	38021	186695	0	NA	NA	Null
Q72D90	DESVH Lipoprotein, putative	0	0	0	170478	0	262275	0.2458	NA	Null
Q72D99	DESVH Universal stress protein	2287245	0	4039920	16932904	20496745	17782601	0.1810	8.7	Null
Q72DA1	DESVH Cytidylate kinase	0	0	0	0	0	273450	0.2846	NA	Null
Q72DA4	DESVH Uracil phosphoribosyltransferase	0	0	0	4629504	3723326	1641253	0.0000	NA	Increase
Q72DA7	DESVH SUF system FeS assembly ATPase SufC, putative	245947	0	0	0	0	0	0.3005	NA	Null
Q72DA8	DESVH Uncharacterized protein	298860	765008	822288	852549	1303096	1470075	0.2443	1.9	Null
Q72DA9	DESVH HD domain/sensory box protein	0	173274	0	222431	759460	173228	0.3290	6.7	Null
Q72DB6	DESVH Type I secretion outer membrane protein, TolC family	30761901	39090463	38390787	129169276	150752262	65017182	0.0017	3.2	Increase
Q72DB7	DESVH Hemolysin-type calcium-binding repeat protein	10376429	6989098	6214710	92722987	81402748	65549931	0.0000	10.2	Increase
Q72DC1	DESVH Uncharacterized protein	0	527937	242247	438439	424766	688251	0.6078	2.0	Null
Q72DC2	DESVH Bifunctional adenosylcobalamin biosynthesis protein	0	0	0	0	89318	0	NA	NA	Null
Q72DC4	DESVH Uncharacterized protein	0	953451	1160682	1505700	1641392	1300714	0.6035	2.1	Null
Q72DC7	DESVH Conserved domain protein	0	0	0	76501	0	0	NA	NA	Null
Q72DC8	DESVH Coenzyme A binding protein	0	0	0	893935	0	0	NA	NA	Null
Q72DC9	DESVH Peptidase, M24 family	0	0	0	0	94077	0	NA	NA	Null
Q72DD1	DESVH Heptosyltransferase family protein	0	0	0	0	103030	0	NA	NA	Null
Q72DD4	DESVH ThiJ/PfpI family protein	0	764721	1197612	869103	1072943	959103	0.7834	1.5	Null
Q72DD7	DESVH Chemotaxis protein CheV	0	0	198931	797913	2200764	1044541	0.0775	20.3	Null
Q72DD9	DESVH Endonuclease III	1492617	84087	0	0	0	0	0.0738	NA	Null
Q72DE1	DESVH Carbohydrate kinase, PfkB family	402635	688565	658618	4976504	7664885	4811219	0.0000	10.0	Increase
Q72DE8	DESVH Multiphosphoryl transfer protein, putative	0	0	0	239569	1078906	184699	0.0010	NA	Increase
Q72DE9	DESVH DAK2 domain protein	0	0	0	293064	0	0	0.3007	NA	Null
Q72DF0	DESVH DAK1 domain protein	149492	0	0	1801775	2237460	1897220	0.0313	39.7	Increase
Q72DF1	DESVH ABC transporter, periplasmic substrate-binding protein, putative	0	0	0	0	2857420	0	NA	NA	Null
Q72DF3	DESVH Response regulator	0	0	2334176	0	0	0	NA	NA	Null
Q72DG1	DESVH Amino acid ABC transporter, ATP-binding protein	0	0	0	675486	641234	1141133	0.0000	NA	Increase
Q72DG2	DESVH Amino acid ABC transporter, permease protein, His/Glu/Gln/Arg/o	0	0	0	641099	60480	0	0.1981	NA	Null
Q72DG3	DESVH Amino acid ABC transporter, periplasmic amino acid-binding protei	3046427	4561484	4954303	2963228	1599906	2304321	0.2622	1.8	Null
Q72DG5	DESVH Glutamate dehydrogenase	0	0	0	153154	123408	154301	0.0339	NA	Increase
Q72DG8	DESVH Uncharacterized protein	5712921	0	0	0	0	0	NA	NA	Null
Q72DH0	DESVH Replicative DNA helicase	0	0	0	446660	11876469	0	0.0044	NA	Increase
Q72DH1	DESVH 50S ribosomal protein L9	243006	3443086	6602965	18127692	10420947	10968245	0.1516	3.8	Null
Q72DH3	DESVH 30S ribosomal protein S6	6062950	4282455	7875556	32581566	35672944	32466092	0.0000	5.5	Increase
Q72DH5	DESVH LPS-assembly protein LptD	0	0	0	118179	0	0	NA	NA	Null
Q72DH6	DESVH Tyrosine--tRNA ligase	795407	658593	1663525	1316772	212821	588565	0.7647	1.5	Null
Q72DH8	DESVH Molybdopterin molybdenumtransferase	0	0	0	0	32454	0	NA	NA	Null
Q72DI0	DESVH Conserved domain protein	11363350	0	8099695	14542993	8512984	7482839	0.8635	1.6	Null
Q72DI6	DESVH UPF0324 membrane protein DVU	0	0	0	0	362337	0	0.2715	NA	Null
Q72DI7	DESVH Ferric uptake regulation protein	437429	354669	571124	601498	556653	753336	0.5345	1.4	Null
Q72DI8	DESVH Peptidase, M16 family	0	42651	0	0	0	608688	0.4334	14.3	Null
Q72DK0	DESVH GTPase Obg	0	0	0	225660	256060	199335	0.0047	NA	Increase
Q72DK1	DESVH 50S ribosomal protein L27	0	337764	0	0	0	0	0.2967	NA	Null
Q72DK2	DESVH 50S ribosomal protein L21	1807883	0	1387275	1150527	446312	0	0.7955	2.0	Null
Q72DL6	DESVH Flagellar FliJ protein	0	12135107	0	0	0	0	NA	NA	Null
Q72DM7	DESVH TPR domain protein	369750	106820	0	49750	56087	0	0.5469	4.5	Null
Q72DN1	DESVH Uncharacterized protein	0	0	0	0	28090	0	NA	NA	Null
Q72DN5	DESVH Chorismate synthase	0	0	0	0	1064529	543138	0.0505	NA	Increase
Q72DN6	DESVH Universal stress protein family	0	0	0	786660	392890	402169	0.0001	NA	Increase
Q72DN8	DESVH Aminotransferase	0	0	0	1113932	554566	328029	0.0000	NA	Increase

Q72DN9	DESVH Homoserine dehydrogenase	0	0	0	0	57489	0	NA	NA	Null
Q72DP5	DESVH Ferredoxin-thioredoxin reductase subunit B	0	298795	504819	865671	1168375	3574562	0.1464	7.0	Null
Q72DP6	DESVH Glutaredoxin, putative	0	0	727336	0	0	0	NA	NA	Null
Q72DP8	DESVH Elongation factor G	0	169944	2431126	4810813	4176860	2896234	0.3304	4.6	Null
Q72DQ5	DESVH 30S ribosomal protein S2	731619	2201351	828870	3457147	1817792	5532430	0.1186	2.9	Null
Q72DQ6	DESVH Elongation factor Ts	7909264	14223994	12044430	5978625	11196455	27868219	0.6405	1.3	Null
Q72DQ9	DESVH Ribosome-recycling factor	3579117	4823433	3568596	43073	126579	3633737	NA	3.1	Null
Q72DR3	DESVH 1-deoxy-D-xylulose 5-phosphate reductoisomerase	0	0	0	0	58358	0	NA	NA	Null
Q72DR4	DESVH Zinc metalloprotease	0	0	0	55354	0	0	NA	NA	Null
Q72DR6	DESVH Flagellar hook-associated protein 2	1159429	1877763	2987169	3546252	3607896	2827923	0.2356	1.7	Null
Q72DS0	DESVH Uncharacterized protein	0	0	0	57854	71055	0	NA	NA	Null
Q72DS2	DESVH Radical SAM domain protein	0	591845	2030641	3187440	1794075	0	0.8212	1.9	Null
Q72DS3	DESVH Delta-aminolevulinic acid dehydratase	0	0	0	119197	0	80725	NA	NA	Null
Q72DS4	DESVH AdoMet-dependent heme synthase	0	0	0	35856	19209	0	NA	NA	Null
Q72DS5	DESVH Siroheme decarboxylase alpha subunit	0	0	0	0	0	73750	NA	NA	Null
Q72DS6	DESVH 3'-phosphate/5'-hydroxy nucleic acid ligase	0	0	0	0	0	34626	NA	NA	Null
Q72DS8	DESVH Uncharacterized protein	11768873	41763970	104534884	82087278	62921524	39477800	0.8112	1.2	Null
Q72DT0	DESVH Heterodisulfide reductase, iron-sulfur-binding subunit, putative	355691	430962	525589	6096929	902742	626952	0.0000	5.8	Increase
Q72DT1	DESVH Heterodisulfide reductase, putative	1533890	116290	98801	676706	604882	63267	0.6537	0.8	Null
Q72DT2	DESVH Adenylyl-sulphate reductase, alpha subunit	8543845	4144439	4085863	9835695	13121652	4931539	0.5146	1.7	Null
Q72DT3	DESVH Adenylylsulphate reductase, beta subunit	0	0	0	329753	0	64699	0.2681	NA	Null
Q72DT6	DESVH EPSP	0	0	0	0	113235	0	NA	NA	Null
Q72DT8	DESVH Aminotransferase	0	0	0	0	172088	177472	0.2659	NA	Null
Q72DT9	DESVH Signal recognition particle protein	66465	195652	2760612	768303	779096	1040634	NA	1.2	Null
Q72DU1	DESVH UPF0109 protein DVU	0	0	0	0	0	1297763	NA	NA	Null
Q72DU4	DESVH 50S ribosomal protein L19	2444917	646986	3521388	1065621	1349321	2539787	0.8698	1.3	Null
Q72DU9	DESVH Phosphocarrier protein HPr	0	0	0	0	0	157999	NA	NA	Null
Q72DV0	DESVH Phosphoenolpyruvate-protein phosphotransferase	0	0	238681	0	3934400	136567	0.3222	17.1	Null
Q72DV4	DESVH Protein translocase subunit SecA	171277	0	0	319408	431570	119369	0.5414	5.1	Null
Q72DW0	DESVH FMN reductase, NADPH-dependent	4021420	1538868	2120027	1344321	2425182	2218698	0.8232	1.3	Null
Q72DW4	DESVH AsmA family protein	0	0	0	24128	0	0	NA	NA	Null
Q72DW5	DESVH Thioredoxin peroxidase	0	0	0	0	0	79655	NA	NA	Null
Q72DW7	DESVH Protein GrpE	0	0	0	0	261791	0	0.3023	NA	Null
Q72DW8	DESVH Chaperone protein DnaK	28035292	69108257	12190491	264639741	96149991	343872028	0.0129	6.4	Increase
Q72DW9	DESVH Peripla	0	0	0	233719	271660	238163	0.0033	NA	Increase
Q72DX1	DESVH Glutamyl-tRNA(Gln) amidotransferase subunit A	0	0	0	379457	275013	0	0.1816	NA	Null
Q72DX6	DESVH Histidine kinase	0	0	0	0	186453	0	NA	NA	Null
Q72DY0	DESVH Uncharacterized protein	15298383	23377069	33246632	407754785	330269460	75925040	0.0000	11.3	Increase
Q72DY2	DESVH Uncharacterized protein	3975011	3931037	7448811	42564137	84313499	34655996	0.0000	10.5	Increase
Q72DY4	DESVH Phosphoribosylaminoimidazole-succinocarboxamide synthase	96149	0	0	6304970	2333326	9198291	0.0004	185.5	Increase
Q72DY5	DESVH Enoyl-[acyl-carrier-protein] reductase [NADH]	0	644332	650997	2938397	3069119	2722406	0.1385	6.7	Null
Q72DZ0	DESVH Cell shape-determining protein MreB	186668	1255751	0	7290090	7695012	4317292	0.0654	13.4	Null
Q72DZ9	DESVH ATP synthase F0, B' subunit, putative	0	0	0	1596827	2112545	89228	0.0001	NA	Increase
Q72E00	DESVH ATP synthase subunit b	1223416	1368758	1701181	3294315	1900822	4507353	0.1201	2.3	Null
Q72E01	DESVH ATP synthase subunit delta	0	0	0	0	0	213162	NA	NA	Null
Q72E02	DESVH ATP synthase subunit alpha	66571242	25321947	21627432	177474020	238250349	95692599	0.0394	4.5	Increase
Q72E03	DESVH ATP synthase gamma chain	577639	936107	18875406	4076517	4609104	1437035	0.7066	2.0	Null
Q72E04	DESVH ATP synthase subunit beta	9861670	6598412	9299586	59543726	39320225	135424184	0.0003	9.1	Increase
Q72E05	DESVH ATP synthase epsilon chain	0	1261565	0	303495	0	0	NA	4.2	Null
Q72E12	DESVH Aminotransferase, class V	0	0	0	0	0	195755	NA	NA	Null
Q72E13	DESVH Transporter, putative	0	0	0	811146	2530688	363383	0.0000	NA	Increase
Q72E14	DESVH Hydroxypyruvate reductase, putative	0	0	0	591565	0	0	0.2315	NA	Null
Q72E18	DESVH Lipoprotein, putative	1360176	1027904	1892737	6799908	5771966	4423897	0.0003	4.0	Increase
Q72E20	DESVH Peptidase, M29 family	0	0	123630	382779	0	0	0.8040	3.1	Null
Q72E21	DESVH Uncharacterized protein	0	507948	0	0	0	0	0.2575	NA	Null
Q72E26	DESVH Amino acid ABC transporter, ATP-binding protein	0	0	0	134940	145454	222465	0.0201	NA	Increase
Q72E29	DESVH Methyl-accepting chemotaxis protein	0	0	0	60977	7435666	109714	NA	NA	Null
Q72E35	DESVH Sigma-54 dependent transcriptional regulator/response regulator	0	0	0	71666	224661	0	0.2951	NA	Null
Q72E38	DESVH Uncharacterized protein	0	0	2513728	0	0	0	NA	NA	Null
Q72E45	DESVH Uroporphyrinogen III methylase	0	346789	0	2237782	2698205	11846117	0.0235	48.4	Increase
Q72E47	DESVH Valine--tRNA ligase	0	0	132937	1093226	852868	0	0.3228	14.6	Null
Q72E54	DESVH Thioredox	0	42140	0	0	0	0	NA	NA	Null
Q72E56	DESVH Formate-dependent phosphoribosylglycinamide formyltransferase	0	0	0	363400	363933	1043578	0.0001	NA	Increase
Q72E63	DESVH Branched-chain amino acid ABC transporter, ATP-binding protein	0	0	0	98428	145806	28975	0.1329	NA	Null
Q72E64	DESVH Branched-chain amino acid ABC transporter, ATP binding protein	0	0	0	0	0	116964	NA	NA	Null
Q72E67	DESVH Amino acid ABC transporter, periplasmic-binding protein	0	0	0	0	653658	0	0.2094	NA	Null
Q72E72	DESVH TRAP transporter solute receptor DctP	0	0	0	660801	126815	284665	0.0021	NA	Increase
Q72E75	DESVH Signal peptidase I	0	0	116114	171291	0	717371	0.4862	7.7	Null
Q72E78	DESVH Malate synthase G	0	0	0	0	25088025	0	NA	NA	Null
Q72E80	DESVH dTDP-4-dehydrorhamnose 3,5-epimerase	0	169833	0	1155363	529827	1113022	0.0988	16.5	Null
Q72E84	DESVH Menaquinone reductase, molybdopterin-binding-like subunit	869171	1867170	3410531	7791130	7877132	4685405	0.0211	3.3	Increase
Q72E85	DESVH Menaquinone reductase, iron-sulfur cluster-binding subunit	2022480	4873803	8722054	11050026	9134902	8702065	0.2519	1.8	Null
Q72E92	DESVH Iron-sulfur cluster-binding protein	0	0	0	0	350931	204575	0.2000	NA	Null
Q72E94	DESVH Protein HflK	0	321477	0	464730	0	2801842	0.3935	10.2	Null
Q72E95	DESVH Protein HflC	3142024	1897389	0	3430688	2209987	626457	0.9560	1.2	Null
Q72E96	DESVH DNA-binding protein, putative	1378823	84367	3945303	7959359	17634415	5570291	0.1299	5.8	Null
Q72E97	DESVH Histidine kinase	0	0	0	0	0	80906	NA	NA	Null
Q72EA3	DESVH Amino acid ABC transporter, periplasmic amino acid-binding protei	0	57228	0	1592643	2091706	279015	0.0029	69.3	Increase
Q72EA7	DESVH Glu	0	0	0	173738	0	202438	0.2634	NA	Null
Q72EB0	DESVH Methyl-accepting chemotaxis protein	264776	780542	2875292	1571067	775671	592604	0.8804	1.3	Null
Q72EB3	DESVH Nitrogen fixation protein NifU	0	74791	0	1507219	3326403	2708657	0.0001	100.8	Increase
Q72EB4	DESVH Cysteine desulfurase	0	2316580	2539209	1497334	1644778	859840	0.9782	1.2	Null
Q72EB6	DESVH Serine O-acetyltransferase	0	0	0	0	32674	0	NA	NA	Null
Q72EC1	DESVH Heat shock protein, Hsp20 family	370287	273241	0	1342798	780108	621543	0.3392	4.3	Null
Q72EC4	DESVH Peptidase, U32 family	0	0	0	0	144445	0	NA	NA	Null
Q72EC8	DESVH Sirohydrochlorin cobaltochelatase CbiKP	385319	252264	374651	0	0	0	0.0013	NA	Decrease
Q72ED1	DESVH Iron compound ABC transporter, periplasmic iron compount-bindir	0	6938707	0	0	0	3405837	NA	2.0	Null
Q72ED2	DESVH Precorrin-2 C20-methyltransferase	0	0	0	0	0	102138	NA	NA	Null
Q72ED3	DESVH Methyl-accepting chemotaxis protein	0	103961	294371	0	47128	75469804	NA	189.6	Null
Q72ED5	DESVH ThiF protein, putative	0	0	0	0	0	281725	0.2808	NA	Null
Q72EE1	DESVH Uncharacterized protein	0	0	0	233863	929749	769955	0.0001	NA	Increase
Q72EE7	DESVH Uncharacterized protein	138828	0	0	0	2917396	71970	NA	21.5	Null
Q72EF1	DESVH Phosphotransbutyrylase	0	0	0	104297	0	170920	0.2925	NA	Null
Q72EF2	DESVH Acetolactate synthase	0	0	0	386511	239908	414852	0.0007	NA	Increase
Q72EF3	DESVH Cytochrome c nitrite reductase subunit NrfA	665878	1252848	1492874	2894755	3026021	5313063	0.0170	3.3	Increase
Q72EF6	DESVH Histidine kinase	0	0	0	0	0	1797877	NA	NA	Null
Q72EH0	DESVH Methyl-accepting chemotaxis protein	0	66408	0	1802835	306039	981902	0.0094	46.5	Increase
Q72EH1	DESVH Adenosylhomocysteinase	0	151975	0	3170474	367609	510070	0.0657	26.6	Null
Q72EH9	DESVH Carbon starvation protein A, putative	0	0	0	1936859	0	0	NA	NA	Null
Q72EI7	DESVH Methyl-accepting chemotaxis protein	0	0	0	297790	497340	92512	0.0047	NA	Increase
Q72EJ0	DESVH Formate dehydrogenase, beta subunit, putative	0	0	0	310690	98422	331095	0.0067	NA	Increase
Q72EJ1	DESVH Formate dehydrogenase, alpha subunit, selenocysteine-containing	0	0	0	149442	142534	137975	0.0311	NA	Increase
Q72EK1	DESVH Formate dehydrogenase formation protein FdhE, putative	0	0	0	524805	221464	0	0.1638	NA	Null
Q72EK5	DESVH CreA protein	0	0	0	107982	105019	224184	0.0331	NA	Increase
Q72EL2	DESVH GAF domain protein	1398074	1053255	3520748	1175754	1014323	193020	0.3143	2.5	Null
Q72EL3	DESVH Glyceraldehyde-3-phosphate dehydrogenase	226301	522562	0	0	307031	87250	0.8647	1.9	Null
Q72EM6	DESVH High-affinity branched-chain amino acid ABC transporter ATP-bindi	0	1125196	0	147766	0	0	NA	7.6	Null
Q72EM7	DESVH High-affinity branched-chain amino acid ABC transporter, ATP bind	0	0	0	288731	1133008	114687	0.0014	NA	Increase
Q72EN0	DESVH High-affinity branched chain amino acid ABC transporter, periplasn	1002610	37376621	0	1088884	2978804	6204938	0.6538	3.7	Null
Q72EN9	DESVH AP endonuclease, family 2	0	0	0	0	268226	0	0.3000	NA	Null
Q72EP7	DESVH Flagellar assembly factor FliW	0	403318	592982	0	11454582	355731	0.2925	11.9	Null
Q72EP8	DESVH Translational regulator CsrA	0	0	0	0	63078	0	NA	NA	Null

Q72EP9	DESVH Flagellar hook-associated protein FlgL, putative	577596	860637	0	4362435	4930451	3218932	0.1296	8.7	Null
Q72EQ0	DESVH Flagellar hook-associated protein 1	0	0	0	1956498	648801	1269174	0.0000	NA	Increase
Q72EQ3	DESVH Flagellar P-ring protein	421334	0	0	447429	206536	815450	0.6540	3.5	Null
Q72EQ7	DESVH Flagellar basal-body rod protein FlgF	0	0	0	86342	94385	0	NA	NA	Null
Q72EQ8	DESVH Ribosome maturation factor RimP	0	0	0	45947	28296	0	NA	NA	Null
Q72EQ9	DESVH Transcription termination/antitermination protein NusA	0	2758263	3924644	85025188	2556844	889608	NA	13.2	Null
Q72ER1	DESVH Translation initiation factor IF-2	3138062	42760995	36409759	4479909	6015466	22323756	0.5802	2.5	Null
Q72ER5	DESVH 30S ribosomal protein S15	1297251	8238094	6326720	6983580	2514270	0	0.8427	1.7	Null
Q72ER6	DESVH Polyribonucleotide nucleotidyltransferase	172796	264711	520570	9036273	5754803	3067008	0.0000	18.6	Increase
Q72ER8	DESVH Uncharacterized protein	0	0	0	0	558339	124748	0.1737	NA	Null
Q72ER9	DESVH SelB translation factor	0	0	0	84479	80429	0	NA	NA	Null
Q72ES3	DESVH DNA polymerase I	658353	0	0	0	0	0	0.2007	NA	Null
Q72ES6	DESVH Uncharacterized protein	0	0	0	0	182854	0	NA	NA	Null
Q72ES9	DESVH Phenylacetate-coenzyme A ligase	0	0	0	425207	295986	167269	0.0019	NA	Increase
Q72ET0	DESVH Phosphoribosylamine--glycine ligase	0	0	0	480334	655352	37181	0.0051	NA	Increase
Q72ET5	DESVH DNA mismatch repair protein MutL	0	422581	0	0	361337	173514	0.9255	1.3	Null
Q72ET9	DESVH Non-specific serine/threonine protein kinase	0	93671	0	0	0	0	NA	NA	Null
Q72EU1	DESVH Isocitrate dehydrogenase (NADP(+))	0	367434	181981	1091348	8422409	803350	NA	18.8	Null
Q72EU7	DESVH Tryptophan synthase alpha chain	0	0	109139	0	0	208107	0.8481	1.9	Null
Q72EU8	DESVH Tryptophan synthase beta chain	0	0	0	0	216056	0	NA	NA	Null
Q72EV1	DESVH Anthranilate phosphoribosyltransferase	0	37966	0	86140	94954	108622	0.3076	7.6	Null
Q72EV3	DESVH Anthranilate synthase, component I	0	0	0	0	0	324477	0.2664	NA	Null
Q72EV6	DESVH Bifunctional chorismate mutase/prephenate dehydratase	0	0	0	816008	511799	362988	0.0000	NA	Increase
Q72EV7	DESVH Predicted 3-dehydroquinate synthase	618954	0	0	498091	372619	488265	0.8232	2.2	Null
Q72EV8	DESVH 2-amino-3,7-dideoxy-D-threo-hept-6-ulosonate synthase	1212512	1626593	0	3595710	3809384	469462	0.6028	2.8	Null
Q72EX0	DESVH GDP-mannose 4,6-dehydratase	398368	0	0	0	1297624	243434	0.7521	3.9	Null
Q72EY1	DESVH Efflux transporter, RND family, MFP subunit	0	0	0	0	0	1101516	NA	NA	Null
Q72EY2	DESVH Transcriptional regulator, TetR family	0	0	0	57461	0	0	NA	NA	Null
Q72EY6	DESVH Ech hydrogenase, subunit EchC, putative	0	0	0	133442	150208	0	0.3005	NA	Null
Q72EY7	DESVH Ech hydrogenase, subunit EchD, putative	0	0	0	262425	1855144	0	0.0422	NA	Increase
Q72EY9	DESVH Ech hydrogenase, subunit EchF, putative	759235	0	737452	1282962	161803	0	0.9689	1.0	Null
Q72F00	DESVH Saccharopine dehydrogenase	1270743	0	0	5297391	17556039	0	0.3931	18.0	Null
Q72F01	DESVH Arginine decarboxylase	0	0	0	0	32353	0	NA	NA	Null
Q72F03	DESVH Probable cytosol aminopeptidase	0	0	0	4893542	2353646	434144	0.0000	NA	Increase
Q72F06	DESVH Potassium uptake protein TrkA, putative	0	963737	0	978333	580397	1601630	0.5802	3.3	Null
Q72F08	DESVH Uncharacterized protein	0	0	0	0	146571	0	NA	NA	Null
Q72F14	DESVH Uncharacterized protein	0	0	0	0	0	505136	0.2175	NA	Null
Q72F17	DESVH HMGL-like domain protein	0	0	0	1455592	713324	1265920	0.0000	NA	Increase
Q72F18	DESVH Endolytic peptidoglycan transglycosylase RlpA	0	0	0	0	0	126090	NA	NA	Null
Q72F19	DESVH Integration host factor subunit alpha	286652	1443322	940363	349271	840418	690030	0.8496	1.4	Null
Q72F23	DESVH Aminotransferase	0	0	1192387	0	834192	0	NA	1.4	Null
Q72F25	DESVH Glycolate oxidase, subunit GlcD, putative	0	0	0	269597	244020	0	0.2320	NA	Null
Q72F27	DESVH Amino acid ABC transporter, ATP-binding protein	0	0	0	0	31645	0	NA	NA	Null
Q72F29	DESVH Amino acid ABC transporter, periplasmic amino acid-binding protei	99939	0	510482	269289	0	0	0.8463	2.3	Null
Q72F31	DESVH Flavoredoxin	0	0	0	152548	91292	0	NA	NA	Null
Q72F49	DESVH 5-formyltetrahydrofolate cyclo-ligase	0	0	0	0	38999	59563	NA	NA	Null
Q72F59	DESVH Sensory box/GGDEF domain protein	0	0	0	0	1998327	0	NA	NA	Null
Q72F60	DESVH Conserved domain protein	197750	0	0	220889	0	803247	0.6540	5.2	Null
Q72F61	DESVH Alcohol dehydrogenase, iron-containing	1056587	886671	816371	2687963	1660417	282378	0.6288	1.7	Null
Q72F62	DESVH Aminotransferase, DegT/DnrJ/EryC1/StrS family	0	0	0	0	103921	0	NA	NA	Null
Q72F65	DESVH NeuB family protein	0	0	0	7184586	3953076	421296	0.0000	NA	Increase
Q72F71	DESVH HPCH/HPAI aldolase family protein	0	0	0	1244068	767681	327143	0.0000	NA	Increase
Q72F72	DESVH NAD-dependent epimerase/dehydratase family protein	0	285104	0	1440228	989825	740675	0.2029	11.1	Null
Q72F73	DESVH 3-deoxy-D-manno-octulosonate cytidylyltransferase	806597	1709856	3052384	1796147	1332218	1267526	0.8698	1.3	Null
Q72F75	DESVH D-isomer specific 2-hydroxyacid dehydrogenase family protein	496496	144091	0	5505829	3379222	2385814	0.0413	17.6	Increase
Q72F76	DESVH Hydrolase, haloacid dehalogenase-like family	0	0	230785	190718	196794	251444	0.6450	2.8	Null
Q72F88	DESVH Hydrogenase expression/formation protein HypE	0	119738	94709	1361253	888224	849439	0.0052	14.5	Increase
Q72F91	DESVH Bifunctional protein FolD	341449	363019	415497	0	0	0	0.0005	NA	Decrease
Q72F92	DESVH Enolase	0	408975	121543	4934837	5024850	4761403	0.0021	27.7	Increase
Q72F95	DESVH NAD-dependent epimerase/dehydratase family protein	61171	260181	171980	2563283	6693569	1526159	0.0000	21.9	Increase
Q72F96	DESVH TPR domain protein	0	0	0	2771544	2664390	2409683	0.0000	NA	Increase
Q72FA1	DESVH Flagellar M-ring protein	0	0	0	36459	0	0	NA	NA	Null
Q72FA2	DESVH Flagellar motor switch protein FliG	764806	189591	0	296965	741890	1520862	0.6078	2.7	Null
Q72FA7	DESVH Flagellar hook protein FlgE	372673	2437924	0	117543	1283012	233961	0.8648	1.7	Null
Q72FB2	DESVH Chemotaxis protein CheX, putative	0	248808	81265	943224	782905	351005	0.1609	6.3	Null
Q72FB6	DESVH Uncharacterized protein	0	0	0	102272	119813	0	NA	NA	Null
Q72FB8	DESVH Peptidase, M24 family	0	0	0	0	179081	0	NA	NA	Null
Q72FB9	DESVH Amine oxidase, flavin-containing	0	0	0	0	244262	0	NA	NA	Null
Q72FC1	DESVH Prokaryotic dksA/traR C4-type zinc finger family protein	0	0	0	92658	102240	0	NA	NA	Null
Q72FC5	DESVH Cyclic pyranopterin monophosphate synthase	0	0	0	330617	468392	170249	0.0014	NA	Increase
Q72FD1	DESVH AhpF family protein/thioredoxin reductase	0	0	125622	432601	810744	6876311	0.0143	64.6	Increase
Q72FD6	DESVH Glyoxalase family protein	0	682086	582626	877380	1033403	0	0.8698	1.5	Null
Q72FD7	DESVH Transcriptional regulator, AraC family	0	0	0	137892	584201	0	0.1685	NA	Null
Q72FD8	DESVH DUF302 domain-containing protein	0	0	0	460452	416556	194710	0.0007	NA	Increase
Q72FE1	DESVH Uncharacterized protein	0	0	0	0	120370	0	NA	NA	Null
Q72FE3	DESVH Histidine kinase	0	95631	0	0	915122	0	NA	9.6	Null
Q72FE5	DESVH Transcriptional regulator, rrf2 protein, putative	0	0	0	558117	353120	170315	0.0010	NA	Increase
Q72FF3	DESVH Universal stress protein family	314685	380597	406928	498769	470427	273977	0.8201	1.1	Null
Q72FF5	DESVH DNA-binding response regulator	1561842	0	326614	213357	229966	0	0.5345	4.3	Null
Q72FG1	DESVH Oxidoreductase, FAD/iron-sulfur cluster-binding domain protein	231627	497693	614922	4260591	2702951	2657275	0.0000	7.2	Increase
Q72FH1	DESVH SEC-C motif domain protein	0	0	0	133359	0	0	NA	NA	Null
Q72FH2	DESVH MTH1175-like domain family protein	629402	3121867	0	5843684	6380949	3608414	0.3582	4.2	Null
Q72FH6	DESVH Serine--tRNA ligase	336920	403700	0	461081	329992	934858	0.6078	2.3	Null
Q72FI6	DESVH Uncharacterized protein	0	0	0	122495	0	0	NA	NA	Null
Q72FN1	DESVH Radical SAM domain protein	0	0	0	0	0	463062	0.2300	NA	Null
Q72FN6	DESVH Molybdenum ABC transporter, periplasmic molybdenum-binding p	250211	556284	786975	193694	1117305	409069	0.8804	1.1	Null
Q72FN8	DESVH Molybdopterin molybdenumtransferase	0	0	0	76509	225583	0	0.2892	NA	Null
Q72FP3	DESVH Methyl-accepting chemotaxis protein	0	909227	0	101895	76327	0	NA	5.1	Null
Q72FP4	DESVH Oligopeptide/dipeptide ABC transporter, periplasmic oligopeptide/	419323	396886	255027	1792433	280726	251912	0.5014	2.2	Null
Q72FP8	DESVH Oligopeptide/dipeptide ABC transporter, ATP-binding protein	525140	0	0	655868	2266015	0	0.6623	5.6	Null
Q72FQ1	DESVH Carbamoyl-phosphate synthase large chain	0	0	0	0	464582	199007	0.1680	NA	Null
Q72FQ3	DESVH Carbohydrate isomerase, KpsF/GutQ family	0	0	0	334831	0	0	0.2902	NA	Null
Q72FQ5	DESVH TsaA-like domain-containing protein	0	0	0	189569	100953	179344	0.0258	NA	Increase
Q72FR5	DESVH Lipoprotein, putative	0	0	0	226535	579446	46017	0.0111	NA	Increase
Q72FR9	DESVH D-beta-D-heptose 1-phosphate adenylyltransferase	0	92904	0	87736	0	0	NA	1.1	Null
Q72FS1	DESVH Tryptophan--tRNA ligase	0	0	0	87193	285104	0	0.2681	NA	Null
Q72FS5	DESVH Response regulator	0	0	0	10749258	3761281	2893862	0.0000	NA	Increase
Q72FT0	DESVH Uncharacterized protein	2898909	549183	237572	838056	1547087	1702920	0.9652	1.1	Null
Q72FU3	DESVH ABC transporter, substrate-binding protein, putative	0	0	0	0	114520	0	NA	NA	Null
Q72FV6	DESVH Glutamine ABC transporter, periplasmic glutamine-binding protein	2276614	3099804	1231639	198694	195177	264626	0.0001	10.0	Decrease
Q72FV8	DESVH Glutamine ABC transporter, ATP-binding protein	0	0	0	0	0	547928	0.2094	NA	Null
Q72FW3	DESVH TonB-dependent receptor	204578	0	290320	2808769	3515467	2870358	0.0116	18.6	Increase
Q72FW8	DESVH Putrescine-binding periplasmic protein	186871	119263	402963	1602044	2575765	1315640	0.0006	7.7	Increase
Q72FX2	DESVH HTHT-type domain-containing protein	6107865	6317904	10719249	677144	10940569	747005	0.6806	1.9	Null
Q72FX3	DESVH GDP-L-fucose synthase	0	0	0	64703	92960	472976	0.0257	NA	Increase
Q72FX8	DESVH Tryptophan synthase beta chain	0	0	0	0	369393	0	0.2687	NA	Null
Q72FX9	DESVH Methylthioribose-1-phosphate isomerase	0	0	0	235210	652285	538133	0.0002	NA	Increase
Q72FY3	DESVH Fumarate hydratase class II	137443	0	0	0	0	0	NA	NA	Null
Q72FZ6	DESVH Uncharacterized protein	0	0	0	508948	0	0	0.2451	NA	Null
Q72FZ7	DESVH Cytidine/deoxycytidylate deaminase domain protein	0	0	0	144684	182642	232610	0.0140	NA	Increase
Q72FZ9	DESVH Uncharacterized protein	0	0	0	1216744	34785	1196474	0.0012	NA	Increase
Q72G01	DESVH RND efflux system, outer membrane protein, NodT family	0	0	63959	231973	318822	190794	0.1539	11.6	Null

Q72G02	DESVH Efflux pump membrane transporter	0	0	0	0	0	31637	NA	NA	Null
Q72G03	DESVH Efflux transporter, RND family, MFP subunit	2825603	3354994	3224623	4160934	4231618	10511971	0.2371	2.0	Null
Q72G04	DESVH AcrB/AcrD/AcrF family protein	0	0	664789	0	0	145883	0.8009	4.6	Null
Q72G05	DESVH Efflux transporter, RND family, MFP subunit	0	0	0	0	0	42455	NA	NA	Null
Q72G07	DESVH Chemotaxis protein CheV	0	0	0	972726	463404	153330	0.0004	NA	Increase
Q72G11	DESVH GTPase Era	0	0	216742	4203649	2491133	2391788	0.0166	41.9	Increase
Q72G12	DESVH Pyridoxal phosphate homeostasis protein	0	76008	66838	91161	0	487169	0.5396	4.0	Null
Q72G17	DESVH Flagellar motor switch protein FliN	0	418573	544489	1023560	726552	1030442	0.4192	2.9	Null
Q72G19	DESVH RNA methyltransferase, TrmH family, group 3	0	71038	0	74006	145256	77081	0.5078	4.2	Null
Q72G24	DESVH DUF523 domain-containing protein	0	0	0	0	382708	0	0.2664	NA	Null
Q72G25	DESVH Uncharacterized protein	0	0	2047158	1837532	2035678	2233027	0.6338	3.0	Null
Q72G27	DESVH DSBA-like thioredoxin domain protein	1729955	5598076	7922093	7253432	10071575	6743669	0.3935	1.6	Null
Q72G42	DESVH Methyl-accepting chemotaxis protein, putative	0	0	0	751043	0	0	NA	NA	Null
Q72G48	DESVH HTH merR-type domain-containing protein	0	0	0	212540	87948	55177	0.0732	NA	Null
Q72G53	DESVH Asparagine--tRNA ligase	0	0	0	71859	0	249916	0.2715	NA	Null
Q72G54	DESVH Universal stress protein family	0	0	981039	423242	788403	1081374	0.7065	2.3	Null
Q72G56	DESVH DNA gyrase subunit A	0	0	0	679156	422594	1251358	0.0000	NA	Increase
Q72G57	DESVH DNA gyrase subunit B	0	0	0	745005	1602710	529803	0.0000	NA	Increase
Q72G58	DESVH Beta sliding clamp	0	0	0	2938397	3013053	2437262	0.0000	NA	Increase
Q72WE2	DESVH Uncharacterized protein	0	0	0	32474	0	0	NA	NA	Null
Q72WE3	DESVH Uncharacterized protein	0	0	0	0	0	29538	NA	NA	Null
Q72WE4	DESVH Conserved domain protein	0	0	0	75307	133680	78819	0.0978	NA	Null
Q72WF7	DESVH CRISPR-associated protein, TM1801 family	973616	672253	0	0	157129	0	0.4394	10.5	Null
Q72WF8	DESVH CRISPR-associated protein, CT1133 family	164165	0	0	0	0	0	NA	NA	Null
Q72WG3	DESVH Uncharacterized protein	0	0	0	10760325	2718883	3070317	0.0000	NA	Increase
Q72WH3	DESVH Uncharacterized protein	0	206460	281843	196329	45894	135840	0.9724	1.3	Null
Q72WH7	DESVH Type 3 secretion system secretin	0	0	0	0	0	43483	NA	NA	Null
Q72WH8	DESVH Type III secretion system protein, IpaC family, putative	248536	110908	180360	550779	648217	574154	0.0843	3.3	Null
Q72WH9	DESVH Type III secretion system target, YopB family	0	0	0	0	0	128356	NA	NA	Null
Q72WI1	DESVH Uncharacterized protein	0	127623	303073	112744	269783	78109	0.9320	1.1	Null
Q72WI4	DESVH Uncharacterized protein	0	0	0	57976	30947	0	NA	NA	Null
Q72WJ7	DESVH Uncharacterized protein	0	243240	2969867	8487543	65573	21747	NA	2.7	Null
Q72WJ8	DESVH Catalase	142512	0	0	0	33676	69375	0.9468	1.4	Null
Q72WL0	DESVH Adenylyl-sulfate kinase	0	0	0	21272335	570764	0	NA	NA	Null
Q72WL4	DESVH Radical SAM domain protein	0	0	0	315790	488201	0	0.1415	NA	Null
Q72WL5	DESVH Sulfotransferase family protein	0	0	0	0	96989	0	NA	NA	Null
Q72WL7	DESVH Glycosyl transferase, group 1 family protein	0	0	0	201618	123181	55448	0.0606	NA	Null
Q72WL8	DESVH Glycosyl transferase, group 1/2 family protein	0	126989	0	0	20885	58916	NA	1.6	Null
Q72WL9	DESVH Conserved domain protein	0	0	1515590	0	0	0	NA	NA	Null
Q72WQ3	DESVH TPR domain protein	167074	0	0	6198189	0	119968	NA	37.8	Null
Q72WR7	DESVH ABC transporter, ATP-binding protein	0	0	0	0	34232	0	NA	NA	Null
Q72WT6	DESVH Uncharacterized protein	0	0	0	0	0	252962	0.2917	NA	Null
Q72WT7	DESVH ParA family protein	0	0	0	0	131731	54377	NA	NA	Null
Q9L385	RUMCH Glucose-1-phosphate adenylyltransferase	43377862	118290671	241967285	162293223	113193812	115758907	0.9353	1.0	Null
