# Supplementary figures and images for: Cross-Feedings, Competition, and Positive and Negative Synergies in a Four-Species Synthetic Community for Anaerobic Degradation of Cellulose to Methane
Source: mBio. 2023 Feb 27;14(2):e03189-22. doi: 10.1128/mbio.03189-22 (PMC10128006; doi:10.1128/mbio.03189-22)

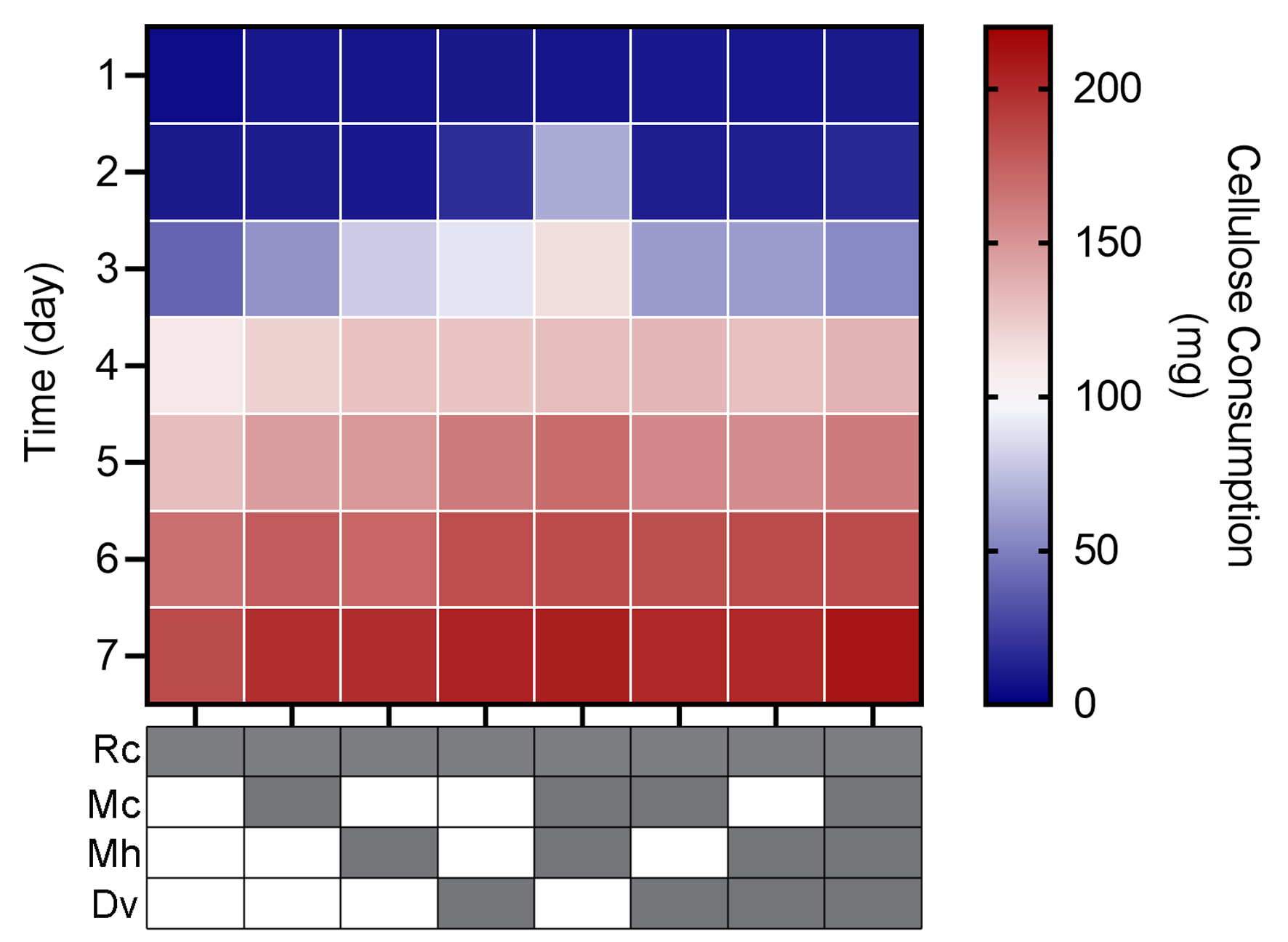

Supplement: FIG S1 [file mbio.03189-22-s0001.tif]

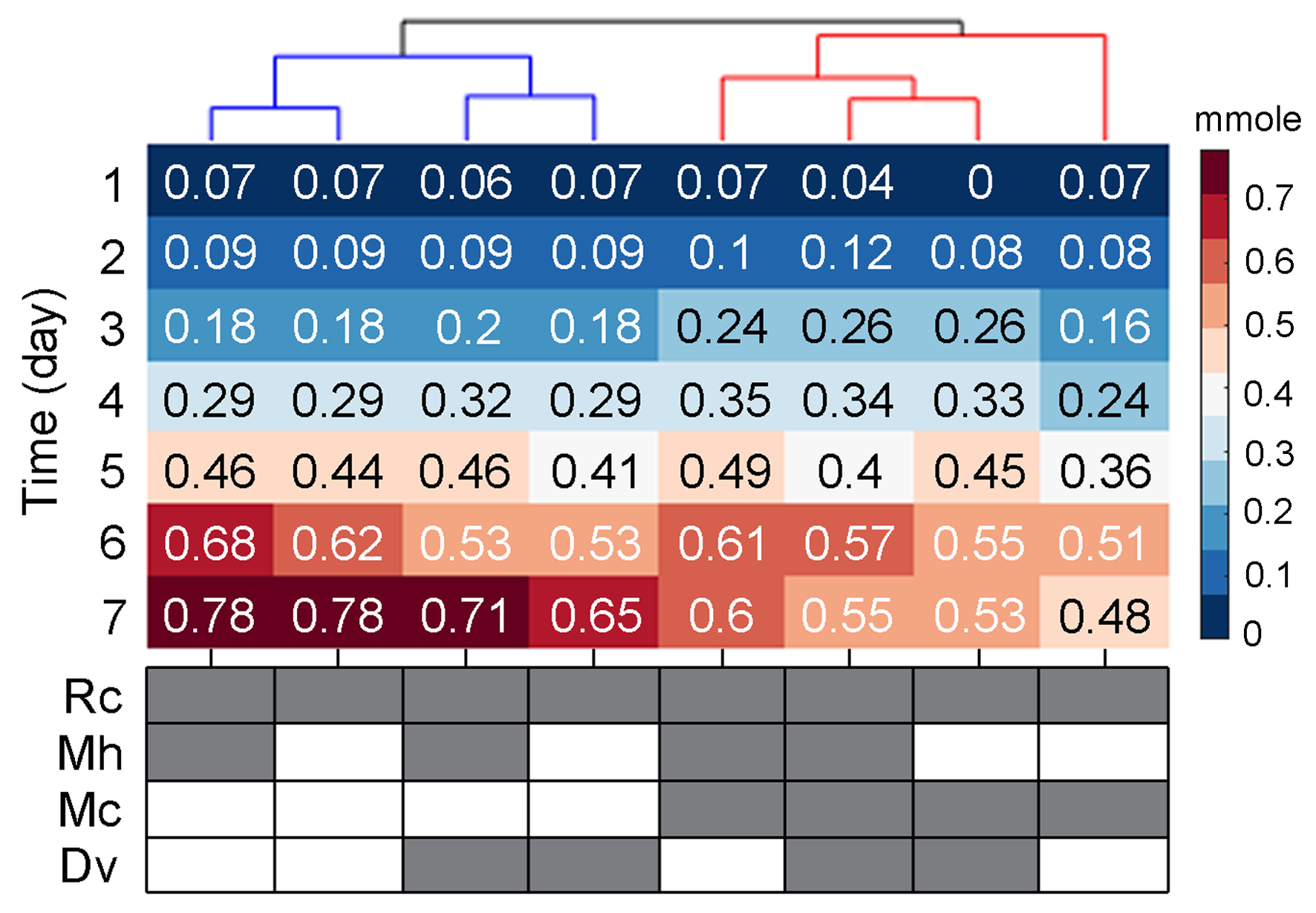

Supplement: FIG S2 [file mbio.03189-22-s0002.tif]
