# Supplementary material for: Cross-Feedings, Competition, and Positive and Negative Synergies in a Four-Species Synthetic Community for Anaerobic Degradation of Cellulose to Methane
Source: mBio. 2023 Feb 27;14(2):e03189-22. doi: 10.1128/mbio.03189-22 (PMC10128006; doi:10.1128/mbio.03189-22)
Supplement: TABLE S3 [file mbio.03189-22-s0005.pdf]

Species	Reaction \ Scenario	Modeling					Proteomics			
		Control Condition		Sulfate addition condition						
		Scenario 1	Scenario 2	Scenario 1	Scenario 2	Scenario 3	Protein Name	Change	Fold Change	*q* -value
R. cellulolyticum	Lactate fermentation	0.90 ± 0.011	0.11 ± 0.002	1.12 ± 0.008	0.87 ± 0.009	0.099 ± 0.001	L-lactate dehydrogenase (Ldh)	Up	3.7	0.04
	Hydrogenic acetogenesis	0 ± 0	0.79 ± 0.009	0 ± 0	0.24 ± 0.001	1.02 ± 0.008	Acetate kinase (Ack)	Null	1.2	0.9
D. vulgaris	Hydrogenic lactate oxidation	1.58 ± 0.018	0 ± 0	2.03 ± 0.016	0 ± 0	0 ± 0	L-lactate dehydrogenase (Ldh)	Null	1.3	NA
	Sulfidogenic lactate oxidation	0 ± 0	0 ± 0	0 ± 0	1.55 ± 0.02	0 ± 0	Sulfide reductase (Srd)	Up	2.0	0.009
	Sulfidogenic hydrogen oxidation	0 ± 0	0 ± 0	1.55 ± 0.02	0 ± 0	1.55 ± 0.02	FeS cluster assembly ATPase	Up	1.8	0.001
M. hungatei	Hydrogenotrophic methanogenesis	0.46 ± 0.005	0.46 ± 0.005	0 ± 0	0 ± 0	0 ± 0	Formate dehydrogenase (Fdh)	Down	27.8	<0.0001
M. concilii	Acetoclastic methanogenesis	0.97 ± 0.02	0.97 ± 0.02	1.38 ± 0.02	1.38 ± 0.02	1.38 ± 0.02	Methyl-coenzyme M reductase (Mcr)	Down	2.2	0.001
